# Pharmacological targeting of the NLRP3 LRR domain with isothiazolinones overcomes CRID3-resistant inflammation

**DOI:** 10.1038/s44321-026-00425-5

**Published:** 2026-04-17

**Authors:** Hawon Woo, Yeonseo Jang, Soyeon Kim, Wonyoung Kim, Fenfen Zhang, Raghvendra Mall, Chirag N Patel, Melan Kurera, Chinh Ngo, Simon H Jiang, Asia Nicotra, Bénédicte F Py, Min Zheng, Si Ming Man, Rajendra Karki

**Affiliations:** 1 Department of Biological Sciences, College of Natural Sciences, Seoul National University, Seoul, Republic of Korea; 2 Institute of Infectious Diseases, Shenzhen Bay Laboratory, Shenzhen, China; 3 Qatar Computing Research Institute, Hamad Bin Khalifa University, Doha, Qatar; 4 Translational Gerontology Branch, National Institute on Aging, NIH, Baltimore, MD USA; 5 Division of Immunology and Infectious Diseases, The John Curtin School of Medical Research, The Australian National University, Canberra, ACT Australia; 6 CIRI, Centre International de Recherche en Infectiologie, Univ Lyon, Inserm, U1111, Université Claude Bernard Lyon 1, CNRS, UMR5308, ENS de Lyon, Lyon, France; 7 The Institute of Molecular Biology and Genetics (IMBG), Seoul National University, Seoul, Republic of Korea

**Keywords:** Immunology, Pharmacology & Drug Discovery

## Abstract

The NLRP3 inflammasome is a key driver in inflammatory, infectious, metabolic, and neurodegenerative diseases. Although the NLRP3 inhibitor CRID3 (also known as MCC950) exhibits potent activity, it cannot inhibit several hyperactive NLRP3 mutations associated with autoinflammatory syndromes and has not progressed clinically, underscoring the need for the development of new NLRP3 inhibitors. Through a high-throughput screening, we identified LOC14, an isothiazolinone-containing small molecule, as a selective NLRP3 inhibitor. Distinct from CRID3, which targets the NACHT domain, LOC14 binds to or near the LRR domain of NLRP3 and inhibits both CRID3-responsive and CRID3-non-responsive hyperactive or gain-of-function NLRP3 variants. Furthermore, we identified that the carbonyl oxygen of the isothiazol-3(2H)-one moiety is critical for inhibitory activity. In vivo, LOC14 exerted anti-inflammatory activity in mouse models of colitis, sepsis, and psoriasis, demonstrating broad physiological and therapeutic relevance. Our findings highlight isothiazolinone-containing compounds as selective NLRP3 inhibitors and provide a promising foundation for developing therapies targeting NLRP3-driven inflammatory diseases.

## Synopsis

LOC14 is identified as a small-molecule inhibitor of the NLRP3 inflammasome that targets the LRR domain, disrupts inflammasome assembly, and reduces inflammation in mouse models of inflammatory diseases.

- LOC14 was identified as a potent inhibitor of the NLRP3 inflammasome activation.
- LOC14 bound to the LRR domain of NLRP3 and disrupted the NLRP3-NEK7 interaction.
- The isothiazolinone moiety of LOC14 was found to be the key structural element mediating NLRP3 inhibition.
- LOC14 also interfered with NLRP3 inflammasome priming by attenuating inflammatory signaling pathways.
- LOC14 reduced inflammation in mouse models of colitis, sepsis, and psoriasis.

## Abstract (web summary)

LOC14 is identified as a small-molecule inhibitor of the NLRP3 inflammasome that targets the LRR domain, disrupts inflammasome assembly, and reduces inflammation in mouse models of inflammatory diseases.

## Graphical abstract

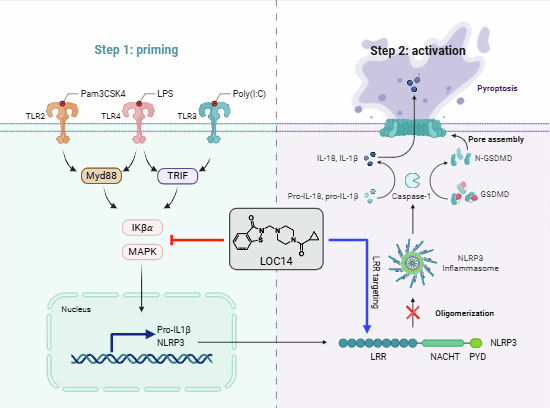

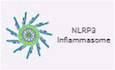

The paper explained **Problem**

The NLRP3 inflammasome contributes to a wide range of human diseases, including autoinflammatory disorders, neurodegenerative diseases, metabolic diseases, and inflammatory bowel disease. Although several NLRP3 inhibitors are currently under clinical investigation, many show limited efficacy or adverse effects. Therefore, the development of NLRP3 inhibitors with distinct mechanisms of action is needed.

**Results**

This study identified LOC14 as a potent inhibitor of the NLRP3 inflammasome. LOC14 binds to or near the LRR domain of NLRP3 and disrupts the interaction between NLRP3 and NEK7, thereby preventing inflammasome assembly. Unlike CRID3, LOC14 inhibits hyperactive CRID3-resistant NLRP3 variants. Mechanistic studies further revealed that the isothiazolinone moiety of LOC14 is essential for its inhibitory activity. In mouse models of inflammatory diseases, including colitis, sepsis, and psoriasis, LOC14 treatment significantly reduced inflammation and disease severity.

**Impact**

These findings identify isothiazolinone-containing compounds as a potential class of NLRP3 inhibitors and suggest that targeting the LRR domain represents an alternative therapeutic strategy to overcome resistance to existing NLRP3 inhibitors. The identification of the isothiazolinone moiety as a critical structural feature also provides a foundation for the rational design of improved NLRP3 inhibitors.

## Introduction

The innate immune system responds to various pathological conditions through the orchestration of highly specialized protein signaling complexes, ensuring host defense and tissue homeostasis. Central to this response is the inflammasome, a multi-protein complex that is assembled in response to pathogen-associated molecular patterns (PAMPs) or damage-associated molecular patterns (DAMPs) (Gurung, 2025; Karki and Kanneganti, 2019; Lamkanfi and Dixit, 2014; Swanson et al, 2019). Once activated, the inflammasome triggers activation of caspase-1, which cleaves the pro-inflammatory cytokines pro-IL-1β and pro-IL-18 into their mature, biologically active forms. Caspase-1 activation also cleaves gasdermin D (GSDMD), allowing its N-terminal fragment to translocate to the plasma membrane (Kayagaki et al, 2015; Shi et al, 2015), where it forms pores and facilitates the release of certain cytokines and cellular contents, ultimately leading to an inflammatory form of cell death called pyroptosis (Karki and Kanneganti, 2021; Man and Kanneganti, 2024).

Among the various inflammasomes, the NOD-like receptor protein 3 (NLRP3) inflammasome has garnered global attention in fundamental and translational science due to its ability to sense a diverse array of PAMPs and DAMPs, triggering inflammation and cell death in multiple disease contexts (Anand, 2025; Hollis and Lukens, 2025; Li et al, 2025; Pandey et al, 2025; Xu and Nunez, 2023). NLRP3 activation is tightly regulated by a sequence of priming and activation events. The priming phase is driven by signals that activate the signaling proteins nuclear factor-kappa B (NF-κB) and extracellular signal-regulated kinase (ERK), leading to the upregulation of NLRP3 and pro-IL-1β (Bauernfeind et al, 2009; Franchi et al, 2009). The subsequent activation phase requires conformational changes in NLRP3 that facilitate the interaction between NLRP3 and the serine/threonine kinase NEK7 (He et al, 2016; Schmid-Burgk et al, 2016; Shi et al, 2016). This interaction is pivotal for the recruitment and oligomerization of the adaptor protein ASC, culminating in the assembly of the NLRP3 inflammasome.

The NLRP3 inflammasome is implicated in various infectious and inflammatory diseases (Vande Walle and Lamkanfi, 2024). Gain-of-function mutations in NLRP3 cause the development of a group of inflammatory diseases known as cryopyrin-associated periodic syndromes (CAPS), characterized by severe localized and systemic inflammation and clinical manifestations owing to aberrant inflammasome activation and excessive IL-1β secretion (Aganna et al, 2002; Feldmann et al, 2002; Hoffman et al, 2001). Beyond genetic predispositions, NLRP3 activation triggered by cholesterol crystals and monosodium urate crystals leads to the progression of atherosclerosis (Duewell et al, 2010) and gout (Martinon et al, 2006), respectively. Moreover, the NLRP3 inflammasome is activated by protein fibrils and aggregates, β-amyloid plaques, and tau fibers, contributing to the pathogenesis of Alzheimer’s disease (Heneka et al, 2013). Despite the therapeutic importance of targeting the NLRP3 inflammasome, the development of effective inhibitors has faced considerable challenges. Several CRID3-based second-generation NLRP3 inhibitors have been developed, but none have received clinical approval (Coll and Schroder, 2025; Mangan et al, 2018), and many cannot inhibit several NLRP3 variants associated with autoinflammatory syndromes (Cosson et al, 2024; Feng et al, 2025; Kim et al, 2025; Vande Walle et al, 2019).

In this study, we used a chemical compound screening to identify the small-molecule LOC14 as an inhibitor of NLRP3. LOC14 bound to the transition LRR domain of NLRP3 and disrupted NLRP3–NEK7 interaction. We identified isothiazolinone as the functional group of LOC14, inhibiting the NLRP3 inflammasome and pyroptosis. Notably, LOC14 did not inhibit the activation of AIM2, NLRP1b, NLRC4, and Pyrin inflammasomes. In mouse models of colitis, sepsis, and psoriasis, administration of LOC14 suppressed inflammatory responses and improved disease outcomes. Overall, our findings highlight isothiazolinone-containing compounds, including LOC14, as promising therapeutic agents for treating NLRP3-associated inflammatory diseases.

## Results

### A chemical library screening reveals LOC14 as an inhibitor of the NLRP3 inflammasome

**Figure 1:**
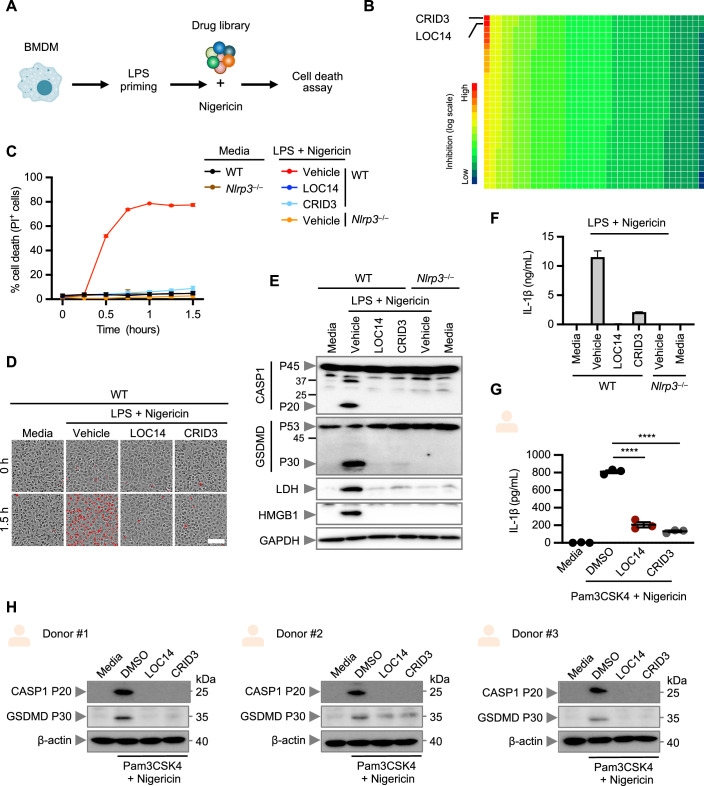
LOC14 inhibits canonical NLRP3 inflammasome activation. (**A**) Schematic representation of the high-throughput screening procedure used to identify potential inhibitors of NLRP3 inflammasome activation. (**B**) Heatmap depicting the efficiency of 1,140 drugs in inhibiting lipopolysaccharide (LPS)- and nigericin-induced cell death, quantified by propidium iodide uptake, normalized to the LPS plus nigericin condition (100%), and visualized as log-transformed values. (**C**–**F**) Real-time analysis (**C**) and representative images of cell death at 0 h and 1.5 h (**D**), and immunoblot analysis of pro- (P45) and cleaved caspase-1 (CASP1; P20), pro- (P53) and cleaved gasdermin D (GSDMD; P30), LDH, and HMGB1 (**E**), and measurement of IL-1β release (**F**) in wild-type (WT) and *Nlrp3*^–/–^ bone marrow-derived macrophages (BMDMs) primed with LPS for 4 h and subsequently stimulated with nigericin for 1 h, with or without LOC14 or CRID3. (**G, H**) IL-1β release (**G**) and immunoblot analysis of cleaved CASP1 (P20) and GSDMD (P30) (**H**) in primary human peripheral blood mononuclear cells (PBMCs) from three healthy donors, primed with Pam3CSK4 for 2.5 h and subsequently stimulated with nigericin for 1.5 h in the presence of DMSO, LOC14, or CRID3. Significance was evaluated using one-way ANOVA followed by Tukey’s multiple comparisons test. ****P < 0.0001. DMSO vs LOC14, *P* = 3.6E-8; DMSO vs CRID3, *P* = 1.8E-8. Each symbol represents an individual donor (**G**, **H**). GAPDH (**E**) and β-actin (**H**) were used as internal controls. Scale bar, 100 μm (**D**). Data are representative of at least three independent experiments (**C**–**H**). Data are shown as mean ± SEM (**C**, **F**, **G**). Source data are available online for this figure.

We screened a chemical library of 1140 compounds in primary bone marrow-derived macrophages (BMDMs) treated with the NLRP3 activator combination of lipopolysaccharide (LPS) plus nigericin, and assessed for cell death to identify potential inhibitors of the NLRP3 inflammasome (Fig. 1A). Among the compounds tested, several inhibitors reduced cell death by more than 70% under the experimental conditions. These included sulfonylurea NLRP3 inhibitor CRID3, LOC14, the steroidal lactone Withaferin A, the caspase-1 dipeptide inhibitor Belnacasan, and the NF-κΒ inhibitor NF-κΒ-ΙΝ-1 (1,6-Heptadiene-3,5-dione) (Fig. 1B). Of these candidates, LOC14 is not known to inhibit NLRP3 previously, whereas CRID3 (Coll et al, 2015), Belnacasan (Wannamaker et al, 2007), NF-κΒ-ΙΝ-1 (Yin et al, 2018), and Withaferin A (Kim et al, 2015) have been reported to inhibit NLRP3 inflammasome activation.

**Figure EV1:**
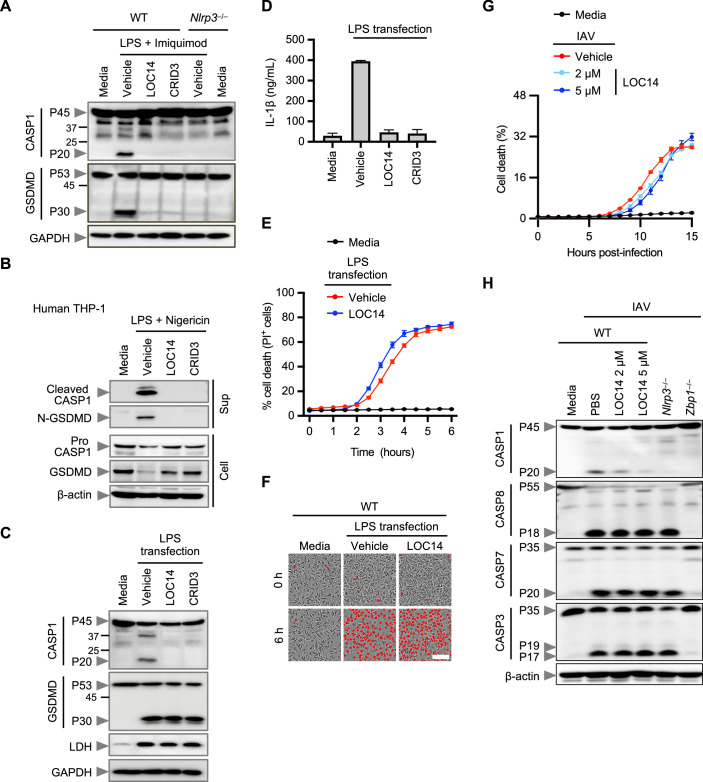
LOC14 suppresses NLRP3 inflammasome activation. (**A**) Immunoblot analysis of pro- (P45) and cleaved caspase-1 (CASP1; P20), and pro- (P53) and cleaved gasdermin D (GSDMD; P30) in wild-type (WT) and *Nlrp3*^–/–^ bone-marrow-derived macrophages (BMDMs) primed with lipopolysaccharide (LPS) for 4 h and subsequently stimulated with imiquimod, with or without LOC14 or CRID3. (**B**) Immunoblot analysis of cleaved CASP1 and GSDMD from supernatants or full-length CASP1 and GSDMD from cell lysates of THP-1 cells primed with LPS and treated with nigericin plus LOC14 or CRID3 for 1 h. (**C**–**F**) Immunoblot analysis of pro- (P45) and cleaved CASP1 (P20), pro- (P53) and cleaved GSDMD (P30), and LDH (**C**), IL-1β release (**D**), real-time analysis (**E**) and representative images of cell death at 0 h and 6 h (**F**) in LPS-transfected WT BMDMs treated with or without LOC14 or CRID3. (**G**) Real-time analysis of cell death in influenza A virus (IAV)-infected WT BMDMs treated with vehicle or LOC14. (**H**) Immunoblot analysis of pro- (P45) and cleaved CASP1 (P20), pro- (P55) and cleaved caspase-8 (CASP8; P18), pro- (P35) and cleaved caspase-7 (CASP7; P20), and pro- (P35) and cleaved caspase-3 (CASP3; P19 and P17) in IAV-infected WT BMDMs treated with LOC14, as well as in *Nlrp3*^–/–^ and *Zbp1*^–/–^ BMDMs. Scale bar, 100 μm (**F**). GAPDH (**A**, **C**) and β-actin (**B**, **H**) were used as internal controls. Data are representative of at least three independent experiments. Data are shown as mean ± SEM (**D**, **E**, **G**).

To validate the role of the new candidate LOC14 in suppressing NLRP3 inflammasome activation, we stimulated wild-type (WT) and *Nlrp3*^–/–^ BMDMs with LPS plus nigericin or LPS plus ATP, in the presence or absence of LOC14 or CRID3. Both LOC14 and CRID3 abolished cell death in WT BMDMs induced by LPS plus nigericin or LPS plus ATP (Fig. 1C,D; Appendix Fig. S1A,B). The cell death inhibitory effect of LOC14 in WT BMDMs was comparable to the outcome achieved in *Nlrp3*^–/–^ BMDMs (Fig. 1C,D; Appendix Fig. S1A,B). Following NLRP3 inflammasome activation, both the cysteine protease caspase-1 and pore-forming protein GSDMD undergo proteolytic cleavage (Kayagaki et al, 2015; Shi et al, 2015), leading to cell death and the release of inflammatory cytosolic contents such as lactate dehydrogenase (LDH) and high mobility group box 1 (HMGB1). Consistent with its inhibitory effect on cell death, LOC14 inhibited the proteolytic cleavage of caspase-1 and GSDMD, and the release of LDH and HMGB1 in BMDMs stimulated with LPS plus nigericin or LPS plus ATP (Fig. 1E; Appendix Fig. S1C). In addition, IL-1β release, which accompanies caspase-1 and GSDMD activation, was abolished in these BMDMs following LOC14 treatment (Fig. 1F; Appendix Fig. S1D). Under LPS plus nigericin stimulation, the IC_50_ values of LOC14 were 1.551 μM for cell death and 0.9808 μM for IL-1β release, whereas those of CRID3 were 0.6307 μM and 0.3538 μM, respectively (Appendix Fig. S1E). Expanding on the activators LPS plus nigericin or LPS plus ATP, which induce NLRP3 activation via a K^+^ efflux-dependent manner, we tested LPS plus imiquimod, which triggers NLRP3 activation independently of K^+^ efflux (Gross et al, 2016). LOC14 also inhibited NLRP3 inflammasome activation induced by LPS plus imiquimod, indicating that its suppressive effect is not restricted to K^+^ efflux-dependent pathways (Fig. EV1A).

To assess the role of LOC14 in human cells, we stimulated the human monocytic cell line THP-1 with LPS plus nigericin in the presence or absence of LOC14. Treatment of LOC14 inhibited the proteolytic cleavage of caspase-1 and GSDMD (Fig. EV1B). Further, in primary human peripheral blood mononuclear cells (PBMCs) from healthy donors, LOC14 attenuated caspase-1 and GSDMD activation, and IL-1β release triggered by Pam3CSK4 plus nigericin (Fig. 1G,H). Although LOC14 is also known as an allosteric inhibitor of protein disulfide isomerase A3 (PDIA3) (Kaplan et al, 2015), gene silencing of PDIA3 in BMDMs did not affect NLRP3 inflammasome activation, suggesting that the inhibitory effect of LOC14 on NLRP3 is independent of its action on PDIA3 (Appendix Fig. S2A–F). Collectively, these data indicate that LOC14 acts as an inhibitor of the NLRP3 inflammasome in both human and mouse cells.

### LOC14 blocks caspase-11- and ZBP1-mediated NLRP3 inflammasome activation

The NLRP3 inflammasome can also be activated by cytoplasmic LPS or infection with Gram-negative bacteria, through a pathway known as the non-canonical activation pathway owing to the requirement for the protease caspase-11 that binds LPS to initiate this process (Karki et al, 2020; Kayagaki et al, 2015; Kayagaki et al, 2011; Shi et al, 2014). To determine whether LOC14 affects non-canonical NLRP3 inflammasome activation, we transfected LPS into BMDMs in the presence or absence of LOC14. Caspase-1 activation and IL-1β release were reduced in LOC14-treated BMDMs, similar to CRID3-treated BMDMs (Fig. EV1C,D). In this case, LOC14 did not inhibit GSDMD cleavage (Fig. EV1C), which is due to caspase-11-dependent proteolytic cleavage of GSDMD that occurs prior to NLRP3 activation (Kayagaki et al, 2011). Moreover, cell death analysis revealed similar kinetics of pyroptosis and the release of LDH in LPS-transfected BMDMs, in the presence or absence of LOC14 (Fig. EV1C,E,F). We next examined a distinct upstream pathway in which influenza A virus (IAV) infection activates the NLRP3 inflammasome through the nucleic acid sensor Z-DNA binding protein 1 (ZBP1), while cell death proceeds independently of NLRP3 (Zheng and Kanneganti, 2020). LOC14 inhibited IAV-induced caspase-1 cleavage without affecting activation of other caspases or overall cell death (Fig. EV1G,H). Together, these results demonstrate that LOC14 does not inhibit caspase-11- or ZBP1-dependent cell death signaling, but instead specifically blocks NLRP3 inflammasome activation downstream of these initiating events.

### LOC14 does not inhibit AIM2, NLRP1b, NLRC4, and Pyrin inflammasomes

**Figure 2:**
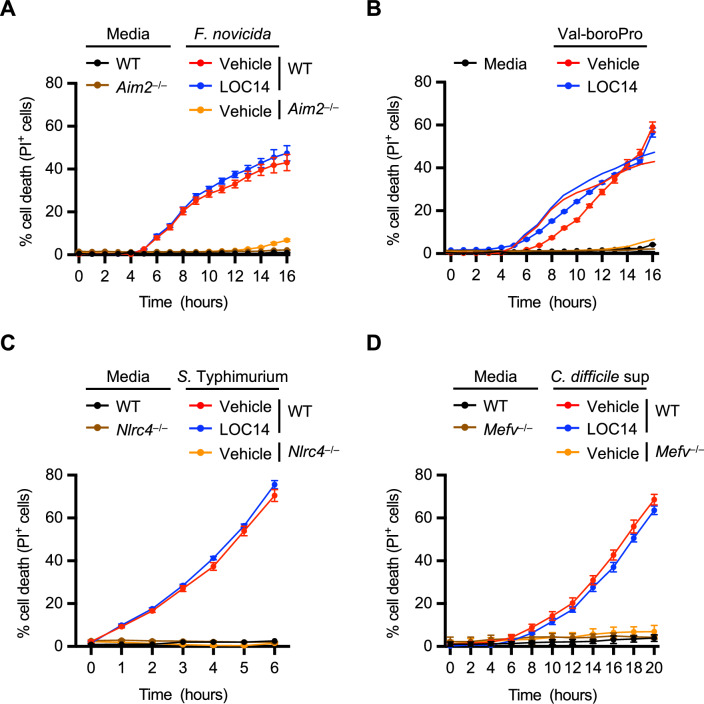
LOC14 does not inhibit AIM2, NLRP1b, NLRC4, and Pyrin inflammasome activation. (**A**) Real-time analysis of cell death in wild-type (WT) and *Aim2*^–/–^ bone marrow-derived macrophages (BMDMs) infected with *Francisella novicida* for 16 h, with or without LOC14. (**B**) Real-time analysis of cell death in WT BMDMs treated with Val-boroPro for 16 h, with or without LOC14. (**C**) Real-time analysis of cell death in WT and *Nlrc4*^–/–^ BMDMs infected with 1 MOI of *Salmonella enterica* subspecies *enterica* serovar Typhimurium for 6 h, with or without LOC14. (**D**) Real-time analysis of cell death in WT and *Mefv*^–/–^ BMDMs incubated with *Clostridioides difficile* supernatant for 20 h, with or without LOC14. Data are representative of at least three independent experiments. Data are shown as mean ± SEM. Source data are available online for this figure.

**Figure EV2:**
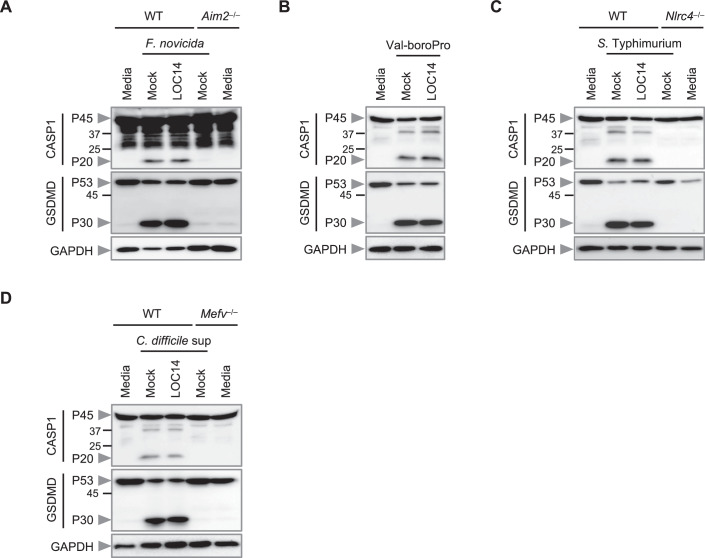
LOC14 does not block caspase-1 activation or GSDMD cleavage downstream of AIM2, NLRP1b, NLRC4, or Pyrin inflammasomes. (**A**–**D**) Immunoblot analysis of pro- (P45) and cleaved caspase-1 (CASP1; P20), and pro- (P53) and cleaved gasdermin D (GSDMD; P30) in bone marrow-derived macrophages (BMDMs) treated with or without LOC14 under the indicated inflammasome-activating conditions: wild-type (WT) and *Aim2*^–/–^ BMDMs infected with *Francisella novicida* (50 MOI) for 16 h (**A**); WT BMDMs treated with Val-boroPro for 16 h (**B**); WT and *Nlrc4*^–/–^ BMDMs infected with *Salmonella enterica* subspecies *enterica* serovar Typhimurium (1 MOI) for 6 h (**C**); and WT and *Mefv*^–/–^ BMDMs incubated with *Clostridioides difficile* supernatant for 20 h (**D**). GAPDH was used as an internal control (**A**–**D**). Data are representative of at least three independent experiments.

Next, we evaluated whether LOC14 functions against other inflammasomes. The DNA-sensing AIM2 inflammasome can be activated either in an IRF1-dependent manner during infection with the bacterium *Francisella tularensis* subspecies *novicida* (*F. novicida*) or independently of IRF1 upon cytosolic delivery of dsDNA such as poly(dA:dT) (Fernandes-Alnemri et al, 2010; Man et al, 2015; Rathinam et al, 2010). In the presence or absence of LOC14, comparable levels of caspase-1 and GSDMD proteolytic cleavage and cell death were observed in BMDMs infected with *F. novicida* or transfected with poly(dA:dT) (Figs. 2A and EV2A; Appendix Fig. S3A–B). Similarly, IRF1 induction was observed in *F. novicida*-infected or IFN-β-treated BMDMs, conditions known to potentiate AIM2 inflammasome activation (Appendix Fig. S3C,D) (Man et al, 2015; Man et al, 2016). These data suggest that LOC14 does not impair IRF1-dependent and IRF1-independent AIM2 inflammasome activation. The NLRP1b inflammasome is activated following the inhibition of cytosolic serine dipeptidases DPP8 and DPP9 (Okondo et al, 2018). However, LOC14 treatment did not impede the activation of caspase-1 and GSDMD or cell death following DPP8/9 inhibitor Val-boroPro stimulation (Figs. 2B and EV2B). NLRC4 inflammasome is activated by bacterial flagellin, or rod and needle proteins of the Type III secretion systems found in *Salmonella enterica* subspecies *enterica* serovar Typhimurium (*S*. Typhimurium) (Karki et al, 2018). LOC14 treatment did not affect caspase-1 and GSDMD activation and the rate of cell death in BMDMs following infection with *S*. Typhimurium (Figs. 2C and EV2C). Finally, Pyrin inflammasome activation occurs as a result of the inactivation of host Rho guanosine triphosphatases caused by infection with the bacterium *Clostridioides difficile* (Xu et al, 2014). We observed that LOC14 treatment did not hinder Pyrin inflammasome activation induced by the supernatant of *Clostridioides difficile* (Figs. 2D and EV2D). These results indicate that LOC14 does not interfere with the activation of the AIM2, NLRP1b, NLRC4, and Pyrin inflammasomes.

### LOC14 inhibits inflammasome oligomerization via binding to NLRP3

**Figure 3:**
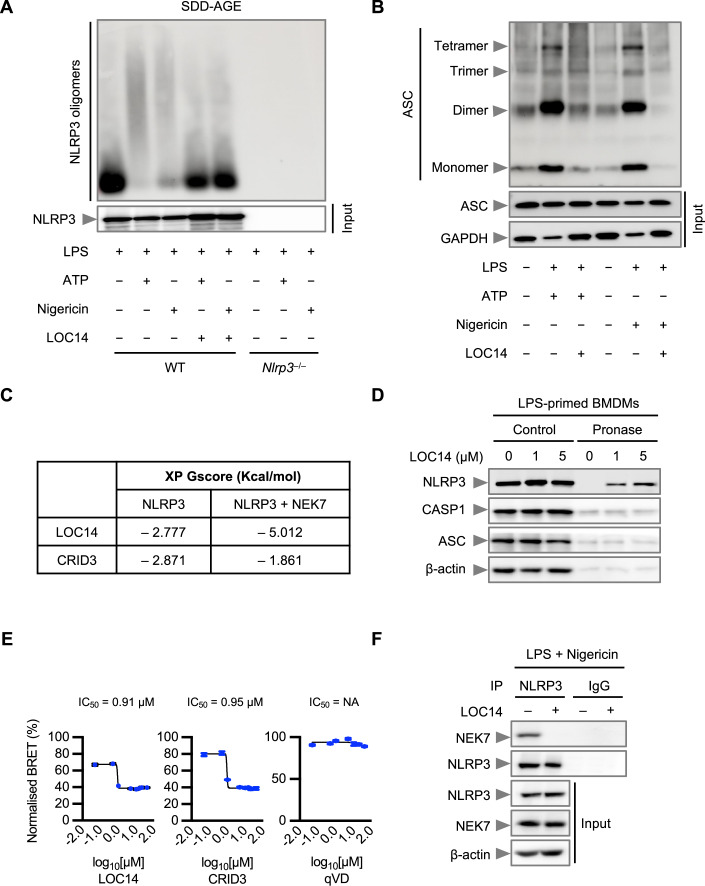
LOC14 suppresses inflammasome activation by targeting NLRP3 and NEK7. (**A**, **B**) Semi-denaturing detergent agarose gel electrophoresis (SDD-AGE) indicating NLRP3 oligomers (**A**) and sodium dodecyl-sulfate polyacrylamide gel electrophoresis (SDS-PAGE) indicating ASC oligomers (**B**) in LOC14-treated wild-type (WT) or *Nlrp3*^–/–^ bone marrow-derived macrophages (BMDMs) primed with lipopolysaccharide (LPS) for 4 h and subsequently stimulated with ATP for 30 min or nigericin for 1 h in the presence of the indicated compounds. (**C**) Glide extra precision (XP) Gscore analysis from molecular docking of LOC14 and CRID3 binding to NLRP3, with or without NEK7. (**D**) Immunoblot analysis of NLRP3, caspase-1 (CASP1), and ASC in LPS-primed BMDMs treated with or without LOC14 and pronase. (**E**) An NLRP3 target engagement assay using HEK293T cells treated with a concentration of 0.05, 0.5, 1, 5, 10, 15, 20, and 40 µM of LOC14, CRID3, or qVD. The normalized Bioluminescence Resonance Energy Transfer (BRET) signal indicates the % of the signal from the NanoBRET tracer on NLRP3 that was being displaced by the inhibitors. The half maximal inhibitory concentration (IC_50_) indicates the concentration of inhibitor required to reduce the NanoBRET tracer signal by 50%. Each symbol represents an individual concentration. (**F**) Immunoblot analysis of NEK7 and NLRP3 following immunoprecipitation (IP) with anti-NLRP3 or IgG control antibodies in LPS-primed THP-1 cells treated with nigericin, with or without LOC14 (5 µM). GAPDH (**B**) and β-actin (**D**, **F**) were used as internal controls. Data are representative of at least two independent experiments. Data are shown as mean ± SEM (**E**). Source data are available online for this figure.

During inflammasome assembly, NLRP3 proteins form active oligomers in a disk-shaped structure, where the C-terminal lobe of NEK7 nestles against both the LRR and NACHT of NLRP3, which is then required to form a larger complex with the inflammasome adaptor protein ASC (Xiao et al, 2023; Yu et al, 2024) (Data ref: Sharif et al, 2019). Indeed, analysis using semi-denaturing detergent agarose-gel electrophoresis (SDD-AGE) revealed that NLRP3 oligomerization induced by LPS plus ATP or LPS plus nigericin was impaired in BMDMs treated with LOC14 (Fig. 3A). Further, ASC oligomerization in BMDMs or the formation of ASC specks in human THP-1 cells stimulated with LPS plus ATP or LPS plus nigericin were impaired in the presence of LOC14 (Fig. 3B; Appendix Fig. S4). Taken together, these results indicate that LOC14 interferes with inflammasome complex assembly at an early stage by simultaneously impairing both NLRP3 oligomerization and subsequent ASC speck formation. Based on this observation, we hypothesized that LOC14 may exert its inhibitory effect by binding to NLRP3.

**Figure EV3:**
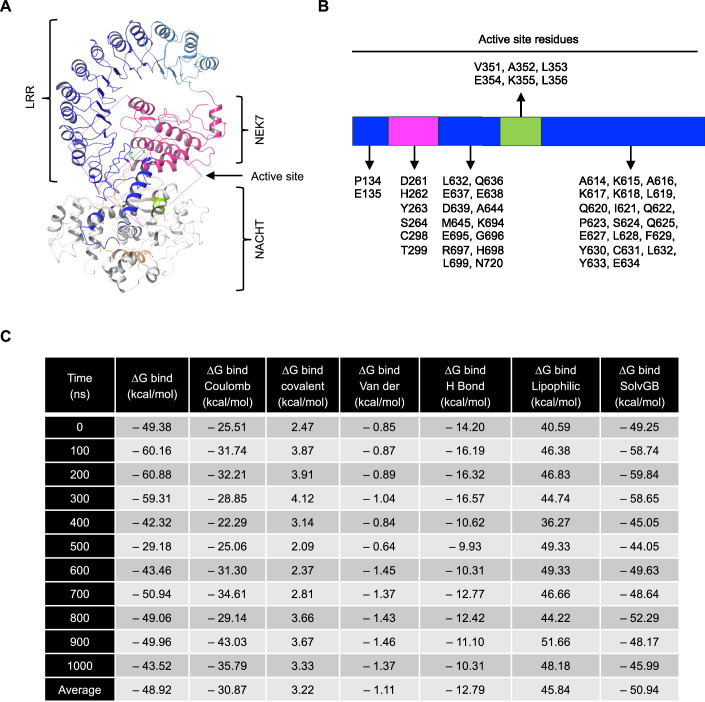
LOC14 exhibits a higher degree of spontaneity in its interaction with NLRP3–NEK7. (**A**, **B**) Structural analysis of NLRP3–NEK7 active site. (**C**) MM/GBSA profiles of LOC14 in interaction with NLRP3 bound to NEK7.

To test this possibility, we first used extra precision XP docking to investigate the interaction between LOC14 and human NLRP3 (Fig. EV3A,B). LOC14 bound to human NLRP3 with a similar affinity at −2.777 kcal/mol compared to that between CRID3 and NLRP3 at −2.871 kcal/mol (Fig. 3C). Notably, in the presence of the kinase NEK7, LOC14 bound to human NLRP3 with an increased affinity at −5.012 kcal/mol (Fig. 3C). The presence of NEK7 did not increase the binding affinity between CRID3 and NLRP3 (Fig. 3C). We then used the Molecular Mechanics Generalized Born Surface Area method to assess the interaction between LOC14 and the NLRP3–NEK7 complex (Genheden and Ryde, 2015). The Gibbs free energy change (∆G) value for LOC14 in the NLRP3–NEK7 complex was −48.92 kcal/mol (Fig. EV3C), indicating that NEK7 binding induces a conformation change on the surface architecture of NLRP3, generating an additional binding interface that enhances LOC14 affinity. To experimentally validate these in silico data, we used the drug affinity responsive target stability (DARTS) technique, which detects drug-protein interactions based on reduced protease susceptibility upon ligand binding (Lomenick et al, 2009). LOC14 protected endogenous NLRP3, but not ASC, caspase-1, or Pyrin, from protease-mediated proteolysis in BMDMs (Fig. 3D; Appendix Fig. S5A). Further, in HEK293T cells expressing full-length human NLRP3 or mouse NLRP3, these proteins were resistant to protease-induced degradation in the presence of LOC14 (Appendix Fig. S5B,C). However, full-length human NLRP6 expressed in HEK293T cells was not resistant to protease-induced degradation (Appendix Fig. S5D). In addition, we used a NanoBRET probe to quantify the engagement between NLRP3 and LOC14 or CRID3 (Teske et al, 2024). In this assay, an NLRP3-Nluc protein is expressed in HEK293T cells that binds to a fluorescent NanoBRET tracer. We found that the addition of either LOC14 or CRID3, but not the pan-caspase inhibitor qVD, to HEK293T cells dose-dependently displaced the NanoBRET tracer from NLRP3 (Fig. 3E), suggesting that LOC14, similar to CRID3, can occupy NLRP3. To further examine the stability of this interaction, we performed washout experiments. Following removal of the compounds, cell death was partially restored in both treatments, with a tendency toward greater recovery in CRID3-treated cells compared with LOC14-treated cells (Appendix Fig. S6). Consistent with the high binding affinity of LOC14 to NLRP3, we found that LOC14 abolished the endogenous interaction between NLRP3 and NEK7 in THP-1 cells (Fig. 3F). Together, these results demonstrate that LOC14 binds to NLRP3, disrupts the interaction between NLRP3 and NEK7, and thereby blocking inflammasome oligomerization at an early stage.

### LOC14 binds to or near the LRR domain of NLRP3

**Figure 4:**
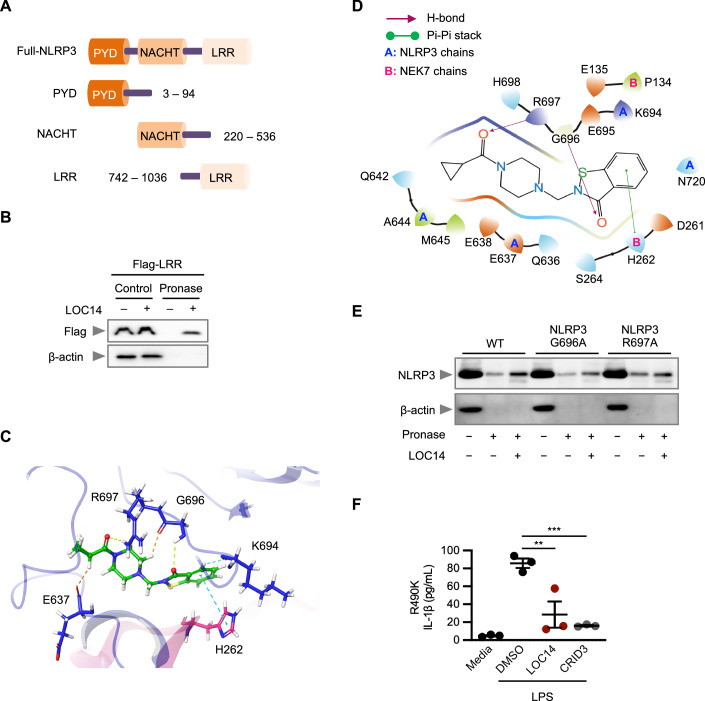
LOC14 binds the LRR domain of NLRP3. (**A**) Schematic of the different domains of NLRP3 overexpressed in HEK293T cells. (**B**) Immunoblot analysis of Flag in HEK293T cells expressing LRR domain of NLRP3, treated with or without LOC14 and pronase. (**C**, **D**) Molecular docking of LOC14 with NLRP3–NEK7 (6NPY): 3D representation (**C**) and 2D representation (**D**) showing ligand interactions via Hydrogen bonds and hydrophobic interactions with the active site of the protein. (**E**) Immunoblot analysis of NLRP3 in HEK293T cells expressing wild-type (WT), G696A, or R697A NLRP3, treated with or without LOC14 and pronase. (**F**) IL-1β release in primary peripheral blood mononuclear cells (PBMCs) from a human patient with cryopyrin-associated periodic syndrome and the NLRP3 gain-of-function mutant R490K, left untreated (media) or treated with LPS for 6 h in the presence of LOC14 or CRID3. Significance was evaluated using one-way ANOVA followed by Dunnett’s multiple comparisons test. **P < 0.01, ***P < 0.001. DMSO vs LOC14, *P* = 0.0021; DMSO vs CRID3, *P* = 0.0006. β-actin was used as an internal control (**B**, **E**). Data are representative of at least three independent experiments (**B**, **E**) or were pooled from three independent fresh blood samples obtained from the same human donor (**F**). Data are shown as mean ± SEM (**F**). Source data are available online for this figure.

**Figure EV4:**
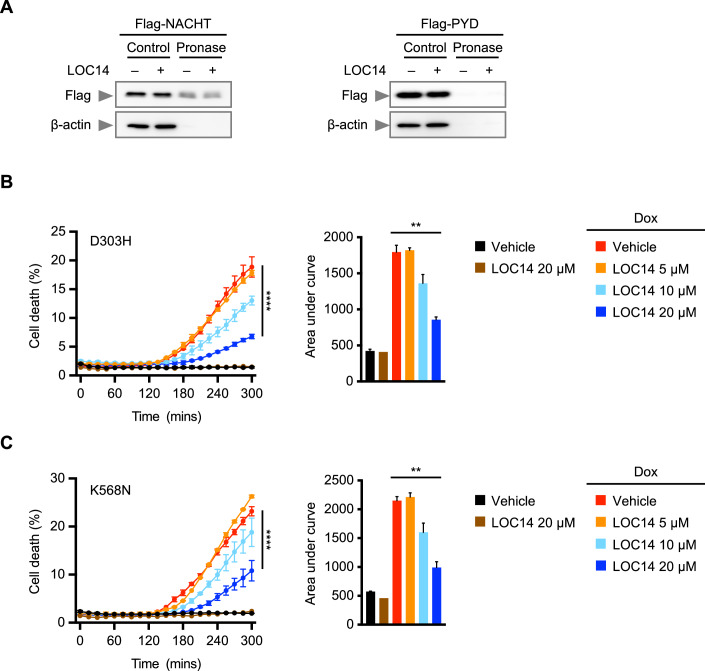
LOC14 suppresses both MCC950-sensitive and -resistant inflammation. (**A**) Immunoblot analysis of Flag in HEK293T cells expressing either NACHT or PYD domains of NLRP3, treated with or without LOC14 and pronase. (**B**, **C**) Human monocytic cell line U937 expressing doxycycline-inducible NLRP3 gain-of-function mutants associated with cryopyrin-associated periodic syndrome, D303H (**B**) and K568N (**C**), were treated with the vehicle control DMSO or LOC14. For the time-course data, Significance was evaluated using two-way ANOVA followed by Tukey’s multiple comparisons test. ****P < 0.0001. Vehicle + Dox vs LOC14 20 μM + Dox, *P* = 4E-15. For the AUC quantification, Significance was evaluated using one-way ANOVA followed by Tukey’s multiple comparisons test. **P < 0.01. Vehicle + Dox vs LOC14 20 μM + Dox, *P* = 0.0014 (**B**, **C**). β-actin was used as an internal control (**A**). Data are representative of at least three independent experiments (**A**). Data are from two independent experiments (*n* = 2) (**B**, **C**).

To identify the specific domains of NLRP3 that bind LOC14, we expressed the LRR, NACHT, and PYD of NLRP3 in HEK293T cells and applied our DARTS assays (Fig. 4A). LOC14 protected the LRR, but not the NACHT or PYD, of NLRP3 against protease-induced degradation (Figs. 4B and EV4A). Detailed analysis of stable molecular interactions between NLRP3, NEK7, and LOC14 revealed that the amino acid residue position R697 of NLRP3, located within the transition LRR domain (amino acid residue positions 640-742) (Hochheiser et al, 2022), formed a hydrogen bond with the carbonyl oxygen bridge found between the piperazine and cyclopropane chemical groups of LOC14. Further, the amino acid G696 of NLRP3 formed a hydrogen bond with the carbonyl oxygen of the benzisothiazolinone moiety of LOC14 (Fig. 4C,D). To experimentally validate the contribution of these residues, alanine substitution mutants of NLRP3 (G696A and R697A) were generated and analyzed by DARTS assay. Compared with WT NLRP3, both mutants retained partial protection against protease-induced degradation (Fig. 4E), suggesting that these residues contribute to, but are not solely responsible for, LOC14 binding. Together, these data support a model in which LOC14 engages the transitional LRR domain of NLRP3.

Given that CAPS patients carry gain-of-function mutations primarily in the NACHT domain of NLRP3, which causes hyperactive inflammasome assembly, we next asked whether LOC14 can suppress inflammasome hyperactivation driven by these NACHT domain mutations (Cosson et al, 2024). To test this, we induced the expression of two well-characterized constitutively active NLRP3 mutants, D303H and K568N, in the human monocytic cell line U937 using doxycycline, leading to inflammasome-dependent cell death (Cosson et al, 2024). Both mutants are non-responsive to CRID3 (Cosson et al, 2024). LOC14 treatment inhibited cell death dose-dependently against both NLRP3 mutants (Fig. EV4B,C). Further, LOC14 blocked LPS-induced IL-1β in primary PBMCs from a patient with CAPS carrying the CRID3-responsive NLRP3 variant R490K (Fig. 4F). These results suggest that LOC14 exerts its inhibitory effects by targeting near the LRR domain, thereby suppressing hyperactivity caused by NLRP3 gain-of-function mutations.

### The isothiazolinone moiety is the functional group inhibiting the NLRP3 inflammasome

**Figure 5:**
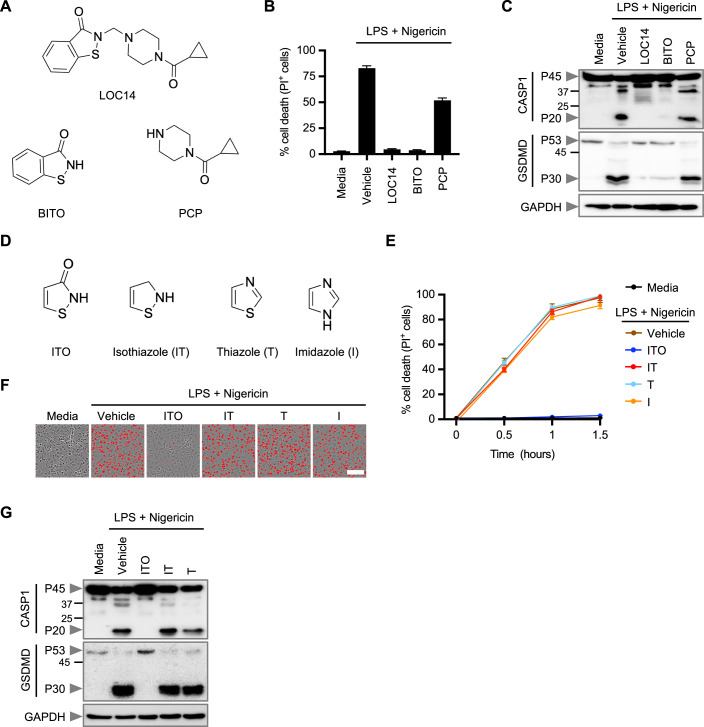
Isothiazol-3(2H)-one moiety is the inhibitory component of LOC14. (**A**) Chemical structures of LOC14, 1,2-Benisothiazol-3(2H)-one (BITO) and 1-(cyclopropylcarbonyl)piperazine-3(2H)-one (PCP). (**B**, **C**) Percentage of cell death (**B**) and immunoblot analysis of pro- (P45) and cleaved caspase-1 (CASP1; P20), and pro- (P53) and cleaved gasdermin D (GSDMD; P30) (**C**) in wild-type (WT) bone marrow-derived macrophages (BMDMs) primed with lipopolysaccharide (LPS) for 4 h and subsequently stimulated with nigericin for 1 h in the presence of the indicated compounds. (**D**) Chemical structures of isothiazol-3(2H)-one (ITO), isothiazole (IT), thiazole (T), or imidazole (I). (**E**–**G**) Real-time analysis (**E**), representative images of cell death (**F**), and immunoblot analysis of pro- (P45) and cleaved CASP1 (P20), and pro- (P53) and cleaved GSDMD (P30) (**G**) in WT BMDMs primed with LPS for 4 h and subsequently stimulated with nigericin for 1 h in the presence of the indicated compounds. Scale bar, 100 μm (**F**). GAPDH was used as an internal control (**C**, **G**). Data are representative of at least three independent experiments. Data are shown as mean ± SEM (**B**, **E**). Source data are available online for this figure.

LOC14 consists of two major chemical moieties: 1,2-Benzisothiazol-3(2H)-one (BITO) and 1-(cyclopropylcarbonyl)piperazine-3(2H)-one (PCP) (Fig. 5A), both of which are precursors for LOC14 synthesis (Kaplan et al, 2015). We sought to determine the relative contributions of these chemical moieties in inhibiting NLRP3. BITO substantially reduced the cleavage of caspase-1 and GSDMD and inhibited cell death in BMDMs stimulated with LPS plus nigericin, similar to the levels achieved by LOC14, whereas little inhibitory effects were observed for PCP (Fig. 5B,C).

From this inhibitory BITO moiety, we further removed the benzene ring within BITO to generate isothiazol-3(2H)-one (ITO), and then systematically removed the carbonyl oxygen from ITO to generate isothiazole (IT), then altered the position of the nitrogen atom IT to generate thiazole (T), and finally replaced the sulfur atom in T with a nitrogen to generate imidazole (I) (Fig. 5D). From these chemical subgroups, we observed that ITO, but not IT, T, or I, inhibited NLRP3 inflammasome activation driven by LPS plus nigericin (Fig. 5D–G). ITO also inhibited NLRP3 inflammasome activation in BMDMs infected with influenza A virus (Appendix Fig. S7). These results indicate that the carbonyl oxygen within the isothiazole ring of LOC14, BITO, and ITO is critical for NLRP3 inhibition. To test whether the isothiazolinone core structure remains essential when additional substituents are present, we next evaluated 4,5-dichloro-2-n-octyl-4-isothiazoline-3-one (DCOIT) and methylisothiazolinone (M-ITO). Both DCOIT and M-ITO exhibited a dose-dependent suppression of LPS plus ATP-induced cell death (Appendix Fig. S8A–D), supporting the functional importance of the isothiazolinone moiety in these chemical derivatives.

### LOC14 inhibits priming of the NLRP3 inflammasome

**Figure 6:**
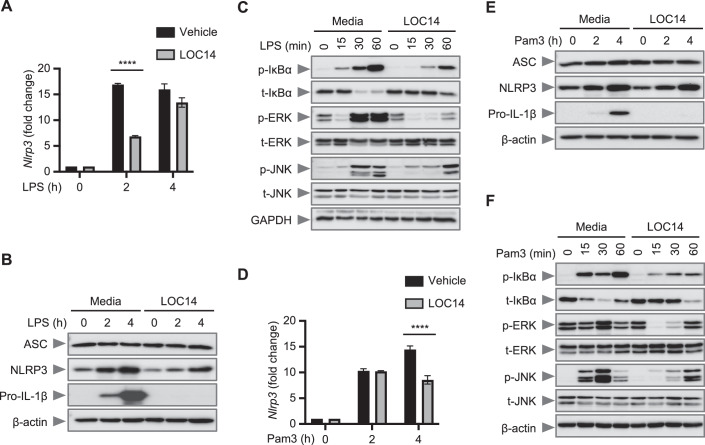
LOC14 inhibits the priming step of the NLRP3 inflammasome. (**A**) Real-time PCR analysis of *Nlrp3* expression in wild-type (WT) bone marrow-derived macrophages (BMDMs) stimulated with lipopolysaccharide (LPS) in the presence of LOC14 for the indicated times. Significance was evaluated using two-way ANOVA followed by Sidak’s multiple comparisons test. ****P < 0.0001. Vehicle vs LOC14 at 2 h, *P* = 1.4E-7. (**B**, **C**) Immunoblot analysis of ASC, NLRP3, and pro-IL-1β (**B**), and phosphorylated IκBα (p-IκBα), total IκBα (t-IκBα), phosphorylated ERK1/2 (p-ERK), total ERK1/2 (t-ERK), phosphorylated JNK (p-JNK), and total JNK (t-JNK) (**C**) in LPS-stimulated WT BMDMs treated with LOC14 for the indicated times. (**D**) Real-time PCR analysis of *Nlrp3* expression in WT BMDMs stimulated with Pam3CSK4 (Pam3) in the presence of LOC14 for the indicated times. Significance was evaluated using two-way ANOVA followed by Sidak’s multiple comparisons test. ****P < 0.0001. Vehicle vs LOC14 at 4 h, *P* = 3.6E-6. (**E**, **F**) Immunoblot analysis of ASC, NLRP3, and pro-IL-1β (**E**), and p-IκBα, t-IκBα, p-ERK, t-ERK, p-JNK, and t-JNK (**F**) in Pam3-stimulated WT BMDMs treated with LOC14 for the indicated times. *Gapdh* (**A**, **D**), β-actin (**B**, **E**, **F**), and GAPDH (**C**) were used as internal controls. Data are representative of at least three independent experiments. Data are shown as mean ± SEM (**A**, **D**). Source data are available online for this figure.

NLRP3 inflammasome activation requires priming, which is regulated by both transcriptional upregulation of *Nlrp3* and *Il1b* expressions via Toll-like receptor (TLR) signaling and post-translational modifications (Bauernfeind et al, 2009; Franchi et al, 2009). To elucidate whether LOC14 also affects priming, BMDMs were treated with LOC14 during the LPS priming phase or activation phase in the presence of nigericin or ATP (Appendix Fig. S9A). LOC14 consistently abolished NLRP3 activation when used in the priming or activation phase (Fig. 1C–F; Appendix Fig. S1A–D and S9A–G). In addition to inhibiting the activation phase, LOC14 delayed the LPS-induced upregulation of NLRP3 mRNA and protein, and markedly attenuated pro-IL-1β protein expression in BMDMs (Fig. 6A,B). These results suggest an inhibition of TLR4-NF-κΒ-dependent transcriptional upregulation of NLRP3 components. Furthermore, LOC14 delayed the kinetics of LPS-induced phosphorylation of priming signaling proteins inhibitor of κB alpha (IκBα), ERK, and c-Jun N-terminal kinase (JNK) (Fig. 6C). Similarly, LOC14 suppressed activation of these signaling pathways and reduced pro-IL-1β upregulation in response to the TLR2 agonist Pam3CSK4 or TLR3 agonist Poly(I:C) (Fig. 6D–F; Appendix Fig. S10A–C). Together, these data indicate that LOC14 interferes with NLRP3 inflammasome priming, with a more pronounced effect on pro-IL-1β expression than on NLRP3 expression itself.

### LOC14 attenuates colitis, sepsis, and psoriasis

**Figure 7:**
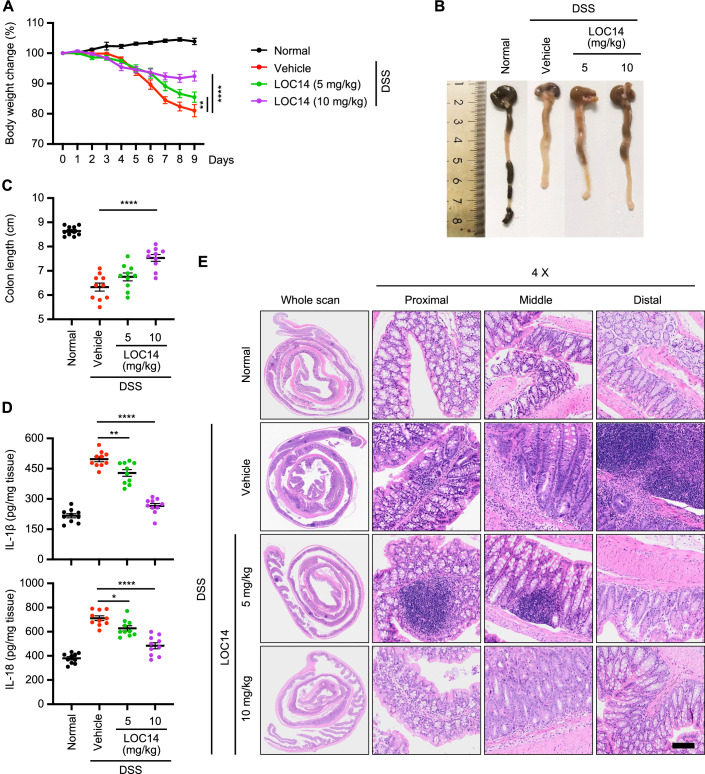
LOC14 ameliorates DSS-induced colonic inflammation in mice. (**A**) Body weight change of wild-type (WT) mice administered with vehicle or LOC14 during dextran sulfate sodium (DSS) treatment. Significance was evaluated using two-way ANOVA followed by Tukey’s multiple comparisons test. **P < 0.01, ****P < 0.0001. Vehicle vs LOC14 (5 mg/kg), *P* = 0.0054; Vehicle vs LOC14 (10 mg/kg), *P* = 6.4E-10. (**B**) Representative images of the colon from WT mice administered with vehicle or LOC14, 9 days after DSS administration. (**C**, **D**) Colon length (**C**) and levels of IL-1β and IL-18 in the colon (**D**) of WT mice administered with vehicle or LOC14, assessed 9 days after DSS administration. Significance was evaluated using one-way ANOVA followed by Tukey’s multiple comparisons test. ****P < 0.0001. Vehicle vs LOC14 (10 mg/kg), *P* = 3.2E-6 (**C**). Significance was evaluated using one-way ANOVA followed by Tukey’s multiple comparisons test. *P < 0.05, **P < 0.01, ****P < 0.0001. Vehicle vs LOC14 (5 mg/kg) in IL-1β, *P* = 0.0040; Vehicle vs LOC14 (10 mg/kg) in IL-1β, *P* = 7.2E-14; Vehicle vs LOC14 (5 mg/kg) in IL-18, *P* = 0.0308; Vehicle vs LOC14 (10 mg/kg) in IL-18, *P* = 1.2E-8 (**D**). (**E**) Representative hematoxylin and eosin (H&E) staining of colon sections. Sections include a whole scan and specific regions (proximal, middle, and distal colon). Each symbol represents an individual mouse (**C**, **D**). Scale bar, 100 μm (**E**). Data are from one experiment representative of two independent experiments (**A**–**E**). Data are shown as mean ± SEM (**A**, **C**, **D**). Source data are available online for this figure.

The NLRP3 inflammasome contributes to inflammatory disorders, including colitis, sepsis, and psoriasis (Sharma and Kanneganti, 2021; Swanson et al, 2019). To evaluate the therapeutic potential of LOC14, we assessed the efficacy of LOC14 across three mouse models of inflammatory diseases. In dextran sulfate sodium (DSS)-induced colitis, WT mice given DSS were treated orally with LOC14 or vehicle once daily. Body weight changes were monitored for 9 days post-DSS administration. LOC14-treated mice exhibited less body weight loss and reduced colon shortening compared with vehicle-treated controls (Fig. 7A–C). The levels of IL-1β and IL-18 were significantly reduced in the colons of mice treated with LOC14 compared with mice treated with a vehicle control (Fig. 7D). Hematoxylin and eosin (H&E) staining revealed diminished cellular infiltration and reduced colon damage in mice treated with LOC14 (Fig. 7E). Histologic parameters, including inflammation, ulceration, and hyperplasia, were markedly attenuated in the proximal, middle, and distal regions of the colon of mice treated with LOC14 (Fig. 7E).

**Figure 8:**
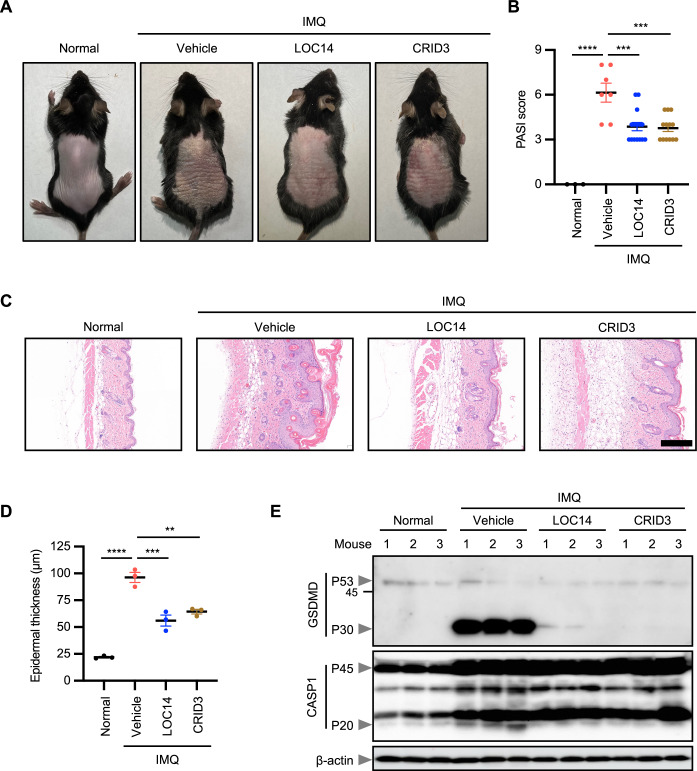
LOC14 alleviates IMQ-induced skin inflammation in mice. (**A**) Representative images of mice developing psoriasis-like skin inflammation, 6 days after Aldara application and 5 days after administration of LOC14 or CRID3. (**B**) Psoriasis area and severity index (PASI) in mice, 6 days after application of imiquimod cream (IMQ). Each symbol represents an individual mouse. Significance was evaluated using one-way ANOVA followed by Dunnett’s multiple comparisons test. ***P < 0.001, ****P < 0.0001. Vehicle vs Normal, *P* = 5.4E-9; Vehicle vs LOC14, *P* = 0.0002; Vehicle vs CRID3, *P* = 0.0002. (**C**) Representative hematoxylin and eosin (H&E) staining of skin sections. Scale bar, 100 μm. (**D**) Measurement of epidermal thickness of skin sections in (**C**). Significance was evaluated using one-way ANOVA followed by Tukey’s multiple comparisons test. **P < 0.01, ***P < 0.001, ****P < 0.0001. Vehicle vs Normal, *P* = 2.5E-6; Vehicle vs LOC14, *P* = 0.0002; Vehicle vs CRID3, *P* = 0.0012. (**E**) Immunoblot analysis of pro- (P53) and cleaved gasdermin D (GSDMD; P30) and pro- (P45) and cleaved caspase-1 (CASP1; P20) in the skin of mice, 6 days after Aldara application. β-actin was used as an internal control (**E**). Data are from one experiment representative of two independent experiments (**A**–**E**). Data are shown as mean ± SEM (**B**, **D**). Source data are available online for this figure.

In our second model of LPS-induced sepsis, mice that were orally administered with LOC14 had reduced circulating levels of IL-1β compared to mice treated with a vehicle control (Appendix Fig. S11). Finally, in a mouse model of psoriasis driven by topical application of imiquimod cream (IMQ) on the back skin for 5 days, we tested the role of LOC14 in inhibiting inflammasome activation and inflammation. Mice treated daily with oral LOC14 showed reduced psoriasis severity defined by the psoriasis area and severity index (PASI) score, similar to that observed in mice treated with CRID3 (Fig. 8A,B). Moreover, mice treated with LOC14 had reduced psoriatic lesions, such as erythema, scaling, and thickening of the skin, compared with mice treated with a vehicle control (Fig. 8A,B). We observed reduced cellular infiltration and epidermal thickness, along with decreased cleavage of GSDMD and caspase-1, in the skin of mice treated with LOC14 compared with mice treated with a vehicle control (Fig. 8C–E). These findings indicate that LOC14 exhibits anti-inflammatory physiological effects across multiple models of inflammatory diseases.

## Discussion

Inflammation is initiated by the innate immune system through a complex network of signaling pathways. Among these pathways, the NLRP3 inflammasome is a key player that has been implicated in a spectrum of inflammatory conditions, from autoinflammatory diseases to metabolic disorders and neurodegenerative conditions (Dubey et al, 2025; Karki et al, 2017; Sharma and Kanneganti, 2021; Swanson et al, 2019). In this study, we identified isothiazolinone-containing drugs as potent inhibitors of NLRP3 and elucidated a distinct mechanism of action for LOC14.

Unlike conventional NLRP3 inhibitors such as CRID3, which stabilize the ADP-bound closed conformation of the NACHT domain to block ATP-dependent activation (Vande Walle and Lamkanfi, 2024), LOC14 acts by binding to or near the LRR domain of NLRP3. This binding disrupts critical interactions between NLRP3 and NEK7, thereby blocking subsequent protein complex assembly and oligomerization. Consistent with this mechanistic distinction, washout experiments showed that LOC14 exhibits a more sustained inhibitory effect than CRID3. This prolonged activity does not only imply irreversible inhibition, but may instead reflect differences in binding mode, which will require further investigation. Docking analysis suggested that LOC14 engages residues G696 and R697 located near the NLRP3–NEK7 binding surface (Data ref: Sharif et al, 2019). However, alanine substitution of either residue resulted in only a modest reduction in LOC14-mediated protease resistance in DARTS assays, indicating that interaction with individual residues is insufficient to disrupt LOC14 engagement. This suggests that LOC14 may interact with NLRP3 through a distributed binding interface involving multiple residues, similar to CRID3 (Ohto et al, 2022). Accordingly, further structural studies will be required to define the precise binding mode of LOC14 within the LRR region. Importantly, this distinct mechanism of action may explain the ability of LOC14 to inhibit CRID3-non-responsive NLRP3 mutants, highlighting the therapeutic potential of LRR-targeting NLRP3 inhibitors in treating CAPS and other NLRP3-driven inflammatory disorders involving gain-of-function NLRP3 mutations resistant to current therapies.

Beyond inflammasome activation, LOC14 also impeded the upregulation of NLRP3 inflammasome components. LOC14 has a less inhibitory effect on NF-κB activation and NLRP3 upregulation compared to ERK activation and pro-IL-1β induction. This potential differential inhibition may imply that NLRP3 upregulation is more reliant on NF-κB signaling, whereas pro-IL-1β induction is more dependent on ERK activation. The inhibitory effects of LOC14 on both priming and activation steps of NLRP3 may offer a broader applicability across different inflammatory diseases. Indeed, our in vivo results demonstrated the efficacy of LOC14 in three preclinical models of inflammatory diseases.

Importantly, LOC14 possesses favorable pharmacokinetic properties, including cell permeability, the ability to cross the blood-brain barrier, and oral bioavailability, with no reported toxicity (Kaplan et al, 2015). The neuroprotective effects of LOC14 in a preclinical model of Huntington’s disease are attributed to its role in inhibiting PDIA3 (Kaplan et al, 2015; Zhou et al, 2018). It is conceivable that the PDIA3-independent, NLRP3-dependent effects of LOC14 might also contribute to the previously observed improvements in cognitive function following LOC14 treatment. Given the implication of inflammasomes in various neurological diseases, our findings potentially broaden the therapeutic application of isothiazolinone-containing drugs to neurological conditions. In conclusion, our findings report LOC14 and isothiazolinone compounds as selective inhibitors of the NLRP3 inflammasome with broad therapeutic effects for inflammatory diseases. Further clinical development of isothiazolinone derivatives as next-generation NLRP3 inhibitors will empower and advance anti-inflammatory therapeutics.

## Methods

**Table:** Reagents and tools table

Reagent/resource	Reference or source	Identifier or catalog number
**Experimental models: organisms/strains**		
C57BL/6J	Raonbio (Yongin, Korea)	N/A
*Nlrp3*^–/–^ C57BL/6J	Dr. SangJoon Lee	N/A
*Aim2*^*–/–*^ C57BL/6J	Dr. SangJoon Lee	N/A
*Nlrc4*^*–/–*^ C57BL/6J	Dr. SangJoon Lee	N/A
*Mefv*^*–/–*^ C57BL/6J	Dr. SangJoon Lee	N/A
*Zbp1*^*–/–*^ C57BL/6J	Dr. SangJoon Lee	N/A
C57BL/6N	Vital River Laboratory Animal Technology Co., Ltd (Beijing)	N/A
*Francisella novicida* strain U112	Man et al, 2015	N/A
*Salmonella Typhimurium* strain SL1344	Dr. Yeong-Jae Seok	N/A
*Clostridioides difficile* strain R20291 (ribotype 027, toxin A⁺/B⁺)	Korean Collection for Type Cultures	KCTC 5009
influenza A virus (A/Puerto Rico/8/34, H1N1 [PR8])	Zheng et al, 2015	N/A
*Escherichia coli* DH5α competent cells	Dr. Jae-Hong Seol	N/A
**Experimental models: cell lines**		
THP1	Kim et al, 2025	N/A
HEK293T	Kim et al, 2025	N/A
U937	Dr. Henry Thomas	N/A
U937 NLRP3 KO	Cosson et al, 2024	N/A
U937 NLRP3^K568N^	Cosson et al, 2024	N/A
U937 NLRP3^D303H^	Cosson et al, 2024	N/A
**Recombinant DNA**		
pNLRP3	Kim et al, 2025	N/A
pNLRP3 G696A	This paper	N/A
pNLRP3 R697A	This paper	N/A
**Antibodies**		
Anti-caspase-1	AdipoGen	Cat# AG-20B-0042
Anti-human cleaved caspase-1	Cell Signaling	Cat# 4199
Anti-human caspase-1	Cell Signaling	Cat# 2225
Anti-caspase-3	Cell Signaling	Cat# 9662
Anti-caspase-3 cleaved	Cell Signaling	Cat# 9661
Anti-caspase-7	Cell Signaling	Cat# 9492
Anti-caspase-7 cleaved	Cell Signaling	Cat# 9491
Anti-caspase-8	AdipoGen	Cat# AG-20T-0137-C100
Anti-caspase-8 cleaved	Cell Signaling	Cat# 8592S
Anti-NEK7	Abcam	Cat# ab133514
Anti-FLAG	Sigma	Cat# F1804
Anti-GSDMD	Abcam	Cat# ab209845
Anti-cleaved GSDMD	Abcam	Cat# ab215203
Anti-LDHA	Proteintech	Cat# 19987-1-AP
Anti-ASC	AdipoGen	Cat# AG-25B-0006
Anti-NLRP3/NALP3	AdipoGen	Cat# AG-20B-0014
Anti-pyrin	Abcam	Cat# AB195975
Anti-HMGB1	Abcam	Cat# ab18256
Anti-SAPK/JNK	Cell Signaling	Cat# 9252
Anti-phospho-SAPK/JNK	Cell Signaling	Cat# 9251
Anti-IκB-alpha	Cell Signaling	Cat# 9242
Anti-phospho-IκB-alpha	Cell Signaling	Cat# 2859
Anti-MAPK p44/42	Cell Signaling	Cat# 9102
Anti-phospho-MAPK p44/42	Cell Signaling	Cat# 9101
Anti-IRF1	Cell Signaling	Cat# 8478
Anti-PDIA3	Abclonal	Cat# A1085
Anti-IL-1β	Abclonal	Cat# A16288
Anti-GAPDH	Cell Signaling	Cat# 5174
Anti-β-actin	Cell Signaling	Cat# 8457
Anti-HRP-conjugated anti-rabbit	Thermo Fisher Scientific	Cat# 31460
Anti-HRP-conjugated anti-mouse	Cellnest	Cat# CNG004-0005
Anti-mouse IgG	Cell Signaling	Cat# 5415
**Oligonucleotides and other sequence-based reagents**		
*Pdia3* siRNA sense 5’-GUGAAGGAGUACGAUGAUA-3’	Bionics, Korea	N/A
*Pdia3* siRNA antisense 5’-UAUCAUCGUACUCCUUCAC-3’	Bionics, Korea	N/A
*Gapdh* forward primer 5’-CGT CCC GTA GAC AAA ATG GT-3’	Bionics, Korea	N/A
*Gapdh* reverse primer 5’-TTG ATG GCA ACA ATC TCC AC-3’	Bionics, Korea	N/A
*Nlrp3* forward primer 5’-TCA GAT TGC TGT GTG TGG GAC TGA-3’	Bionics, Korea	N/A
*Nlrp3* reverse primer 5’-AGC TCA GAA CCA ATG CGA GAT CCT-3’	Bionics, Korea	N/A
*Nlrp3* G696A forward primer 5’-GAAAAGGAAGCCCGACAC-3’	Bionics, Korea	N/A
*Nlrp3* G696A reverse primer 5’-TCGGGCTTCCTTTTCCT-3’	Bionics, Korea	N/A
*Nlrp3* R697A forward primer 5’-GAAAAGGAAGGC GCA CACC-3’	Bionics, Korea	N/A
*Nlrp3* R697A reverse primer 5’-CCATATCAAGGTG TGC GCCT-3’	Bionics, Korea	N/A
**Chemicals, enzymes and other reagents**		
DMEM	Biowest	Cat# L0103-500
Fetal bovine serum	Thermo Fischer Scientific	Cat# 16000044
Penicillin and streptomycin	Biowest	Cat# L0022-100
Non-essential amino acids	Thermo Fisher Scientific	Cat# 11140-050
Human serum	Biowest	Cat# S4190
RPMI 1640	Biowest	Cat# L0498
Tryptic Soy Broth	Formedium	Cat# TSB0110
L-cysteine	ThermoFisher Scientific	Cat# BP376-100
Luria-Bertani (LB) broth	Formedium	Cat# LMM0104
Brain Heart Infusion Agar	BD Biosciences	Cat# 211065
Sheep blood	Kisanbio	Cat# MB-S1876
Vitamin K1-hemin solution	Kisanbio	Cat# MB-V0761
LPS	Sigma	Cat# L2630
LPS	Invivogen	Cat# tlrl-smlps
ATP	Roche	Cat# 10127531001
Nigericin	Cayman	Cat# 11437
LOC14	Selleckchem	Cat# S0321
CRID3	Selleckchem	Cat# S7809
1,2-Benzisothiazol-3(2H)-one	Tokyo Chemical Industry	Cat# B3767
1-(cyclopropylcarbonyl)piperazine	Tokyo Chemical Industry	Cat# C3112
Isothiazol-3(2H)-one	Tokyo Chemical Industry	Cat# I1172
Isothiazol	Tokyo Chemical Industry	Cat# I0982
Thiazole	Tokyo Chemical Industry	Cat# T0185
Imidazole	Tokyo Chemical Industry	Cat# I001
4,5-dichloro-2-n-octyl-4-isothiazoline-3-one	MedChemExpress	Cat# HY-W041308
Methylisothiazolinone	TargetMol	Cat# T19774
Phorbol myristate acetate	Sigma-Aldrich	Cat# P1585
Imiquimod	Invivogen	Cat# tlrl-imqs
Poly(dA:dT)	Invivogen	Cat# tlrl-patn
Opti-MEM	ThermoFisher Scientific	Cat# 31985070
Val-boroPro	Selleckchem	Cat# S8455
Pam3CSK4	InvivoGen	Cat# tlrl-pms
Poly(I:C) HMW	InvivoGen	Cat# tlrl-pic
Gentamicin	ThermoFisher Scientific	Cat# 15750060
Lymphoprep™	STEMCELL Technologies	Cat# 07851
Custom compound library	TargetMol	N/A
Propidium iodide	Invitrogen	Cat# P3566
Immobilon Forte Western HRP Substrate	Millipore	Cat# WBLUF0500
TRIzol reagent	Invitrogen	Cat# 15596026
Protease pronase	AbMole Bioscience	
qVD	Selleckchem	Cat# S7311
Doxycycline	Sigma	D3447
Suberic acid bis	Sigma	Cat# S1885
Dextran sodium sulfate	Yeason	Cat# 60316ES25
Aldara cream	Dong-A Science	N/A
PVDF membrane	Millipore	IPVH00010
4% formaldehyde	Cellnest	Cat# CNP015-1000
IFN- β	PBL Assay	Cat# 12400-1
**Software**		
Incucyte® Cell-by-Cell Analysis	Satorius Software	Cat# 9600-0031 https://www.sartorius.com/en/products/live-cell-imaging-analysis/live-cell-analysis-software/incucyte-cell-by-cell-analysis-software
GraphPad Prism 10.0 software	GraphPad Software, Inc.	https://www.graphpad.com/
ImageJ	National Institutes of Health	https://imagej.nih.gov/ij/
EndNote X8	EndNote Software	https://endnote.com/
LigPrep program	Schrödinger, Inc.	https://www.schrodinger.com/platform/products/ligprep/
The Optimized Potentials for Liquid Simulations (OPLS) 2005 force field	Schrödinger, Inc.	https://www.schrodinger.com/platform/products/force-field-bundle/
**Other**		
NanoBRET TE NLRP3 Kit	Promega	N/A
Xfect	Takara	Cat# 631318
ELISA kits for mouse IL-18	Invitrogen	Cat# BMS618-3
ELISA kits for mouse IL-1β	Invitrogen	Cat# 88-7013-88
ELISA for human IL-1β	ThermoFisher	BMS224-2TEN
Herculase II Fusion Enzyme with dNTPs Combo	Agilent	Cat# 600677
In-Fusion®Snap Assembly Master Mix	Takara	Cat# 638948
Lipofectamine 3000	Invitrogen	Cat# L3000015
Neon^TM^ Transfection System	Invitrogen	MPK5000
M-MLV cDNA synthesis kit	Enzynomics	EZ006S
TB green premix ex Taq	Takara	RR420
ROX reference dye	Takara	AM21069A
BCA Protein Assay Kit	Beyotime Biotechnology	
protein A/G Agarose Resin	Yeason	36403ES
Cryo-EM structure of NLRP3 and NEK7	Sharif et al, 2019	PDB: 6NPYhttps://www.rcsb.org/structure/6NPY
0.22 μm filter	Corning	Cat# 431219
Amersham ImageQuant system	Cytiva	Cat# 29399481
SimpliAmp thermal cyclers	Thermo Fisher Scientific	
QuantStudio 3 Real-Time PCR System	Thermo Fisher Scientific	
CQ1 high content screening microscope	Yokogawa	

### Methods and protocols

#### Mice

C57BL/6 J (wild-type) mice were purchased from Raonbio (Yongin, Korea), and *Nlrp3*^–/–^, *Aim2*^–/–^, *Nlrc4*^–/–^, *Mefv*^–/–^, and *Zbp1*^–/–^ mice were kindly provided by Dr. SangJoon Lee (Ulsan National Institute of Science and Technology) (Oh et al, 2023). Mice were housed and bred under protocols approved by the Seoul National University Committee on the Use and Care of Animals. C57BL/6N mice were purchased from the Vital River Laboratory Animal Technology Co., Ltd (Beijing) and kept in a specific pathogen-free facility at the animal resource center at Shenzhen Bay Laboratory. Mice were maintained with a 12 h light/dark cycle and were fed standard chow. Both male and female age- and sex-matched 6- to 9-week-old mice were used in this study. Animal studies were conducted under protocols approved by the Seoul National University committee and the Regional Ethics Committee for Animal Experiments at Shenzhen Bay Laboratory.

#### Cell culture

Primary bone marrow-derived macrophages (BMDMs) were obtained from the bone marrow of mice. Cells were cultured for 7 days in DMEM (Biowest, L0103-500) with 30% L929 conditioned media, 10% heat-inactivated fetal bovine serum (HI-FBS; Thermo Fischer Scientific, 16000044), 1% penicillin and streptomycin (Biowest, L0022-100), and 1% non-essential amino acids (Thermo Fisher Scientific, 11140-050). BMDMs were then seeded into DMEM media supplemented with 1% non-essential amino acids, 1% penicillin and streptomycin, and 10% HI-FBS, at a density of 1 × 10^6^ cells into 12-well plates and incubated at 37 °C overnight unless otherwise described. In the indicated experiments, DMEM media supplemented with 10% human serum (Biowest, S4190), 1% penicillin and streptomycin, and 1% non-essential amino acids were used during the stimulation procedure. THP1-ASC-GFP cells were established by transducing lentivirus expressing ASC-GFP and then selected via flow cytometry. The cells were grown in RPMI 1640 (Biowest, L0498) with 10% FBS. All cultures were routinely tested for mycoplasma contamination.

#### Bacterial culture

*F. novicida* strain U112 was grown overnight under aerobic conditions at 37 °C in Tryptic Soy Broth (Formedium, TSB0110) supplemented with 0.2% L-cysteine (ThermoFisher Scientific, BP376-100). Bacteria were subcultured (1:10) for 4 h at 37 °C in fresh Tryptic Soy Broth supplemented with 0.2% L-cysteine. *Salmonella* Typhimurium strain SL1344 was inoculated into Luria-Bertani (LB) broth (Formedium, LMM0104) and incubated overnight under aerobic conditions at 37 °C. *S*. Typhimurium SL1344 was subcultured (1:10) for 4 h at 37 °C in fresh LB broth to generate bacteria grown to log phase. *C. difficile* ATCC 9689 (Korean Collection for Type Cultures, 5009) was streaked onto brain heart infusion agar (BD Biosciences, 211065) and incubated overnight at 37 °C in an anaerobic chamber. Single colonies were inoculated into tryptic soy broth with 5% sheep blood (Kisanbio, MB-S1876), 1% vitamin K1-hemin solution (Kisanbio, MB-V0761), 0.05% L-cysteine (ThermoFisher Scientific, BP376-100) at 37 °C anaerobically. The *C. difficile* supernatant was prepared by centrifugation to obtain the culture supernatant, followed by filtration of this supernatant through a 0.22 μm filter (Corning, 431219).

#### Influenza A virus culture

The influenza A virus (A/Puerto Rico/8/34, H1N1 [PR8]) was generated by reverse genetics as previously described (Zheng et al, 2015). Virus stocks were propagated by inoculation of seed virus into the allantoic cavity of 9- to 11-day-old embryonated chicken eggs. Virus titer was measured by plaque assay in MDCK cells. For IAV infection, BMDMs were infected at an MOI of 20 in DMEM media. After absorption for 2 h, cells were supplemented with 10% FBS and then incubated for the indicated time.

#### Cell stimulation

Seeded BMDMs were first gently washed with PBS before stimulation. For activation of the canonical NLRP3 inflammasome, cells were primed for 4 h with 100 ng/mL LPS (InvivoGen, tlrl-smlps) and were stimulated with 5 mM ATP (Roche, 10127531001) or 20 μM nigericin (Cayman, 11437) for indicated time with or without 5 μM LOC14 (Selleckchem, S0321), 1 μM CRID3 (Selleckchem, S7809), 5 μM 1,2-Benzisothiazol-3(2H)-one (Tokyo Chemical Industry, B3767), 5 μM 1-(cyclopropylcarbonyl)piperazine (Tokyo Chemical Industry, C3112), 5 μM Isothiazol-3(2H)-one (Tokyo Chemical Industry, I1172), 5 μM Isothiazol (Tokyo Chemical Industry, I0982), 5 μM Thiazole (Tokyo Chemical Industry, T0185), 5 μM Imidazole (Tokyo Chemical Industry, I001), 1, 5, and 10 μM of 4,5-dichloro-2-n-octyl-4-isothiazoline-3-one (MedChemExpress, HY-W041308), and 1, 5, and 10 μM of methylisothiazolinone (TargetMol, T19774). For NLRP3 inflammasome activation in human monocytes, THP1-ASC-GFP cells were plated in 24-well plates (5 × 10^5^ cells per well) and treated with 100 ng/mL phorbol myristate acetate (Sigma-Aldrich, P1585) for 48 h for differentiation. Then, the cells were washed twice with PBS, followed by a rest for another 24 h. Cells were first primed with 500 ng/mL LPS for 1 h and then treated with 10 μM nigericin plus 5 μM LOC14, or nigericin plus 5 μM CRID3 for 45 min. For activation of the potassium-independent NLRP3 inflammasome, BMDMs were treated with 100 ng/mL LPS plus 20 μg/mL imiquimod (InvivoGen, tlrl-imqs). For transfection of DNA or LPS, each reaction consisted of 2 μg of poly(dA:dT) (InvivoGen, tlrl-patn) or 1 μg of LPS resuspended in PBS and mixed with 0.6 μL of Xfect polymer in Xfect reaction buffer (Clontech Laboratories Inc, 631318). After 10 min, DNA complexes were added to cells that were preincubated in Opti-MEM (ThermoFisher Scientific, 31985070) for 1 h. For activation of the NLRP1 inflammasome, cells were treated with 50 μΜ Val-boroPro (Selleckchem, S8455) for the indicated time. For mRNA expression and signaling, BMDMs were stimulated with 100 ng/mL LPS, 1 μg/mL Pam3CSK4 (InvivoGen, tlrl-pms), and 5 μg/mL Poly(I:C) HMW (InvivoGen, tlrl-pic) for the indicated time. For IRF1 expression, BMDMs were primed with 50 ng/ml IFN-β for 4, 12, or 24 h. For bacterial infection, the following conditions were used: *F. novicida* at an MOI of 100 for 16 h of incubation (for activation of caspase-1 and GSDMD) and an MOI of 50 for 4, 12, or 24 h of incubation (for expression of IRF1); *S*. Typhimurium at an MOI of 0.1 for 3 h of incubation (for activation of caspase-1 and GSDMD). After 4 h of infection, bacteria were washed off with PBS and treated with 50 μg/mL gentamicin (ThermoFisher Scientific, 15750060).

Human blood was obtained with consent from donors in accordance with the WMA Declaration of Helsinki and the Department of Health and Human Services Belmont Report. All procedures were approved by The Australian Capital Territory (ACT) Health Human Research Ethics Committee and The Australian National University Human Research Ethics Committee under protocols ETH.1.16.011, 2022.ETH.00059, 2015/079, and H/2023/1429. Human peripheral blood mononuclear cells (PBMCs) were isolated from blood by density gradient centrifugation over Lymphoprep™ (07851, STEMCELL Technologies) and suspended at 1 × 10^7^ cells/mL in RPMI-1640 supplemented with 10% FBS, 1% penicillin and streptomycin, and 1% L-Glutamine. PBMCs were seeded in antibiotic-free media at a concentration of 1 × 10^6^ cells per well in 24-well plates. PBMCs were primed with Pam3CSK4 for 2.5 h, followed by treatment with either DMSO, 5 μM LOC14, or 10 μM CRID3 for 30 min. PBMCs were then stimulated with 10 μM nigericin for 1.5 h. PBMCs from a human patient with CAPS carrying an NLRP3 R490K mutation were seeded in antibiotic-free media at a concentration of 1 × 10^6^ cells per well in 24-well plates. PBMCs were either left untreated or treated with DMSO, 10 μM LOC14, or 10 μM CRID3 for 30 min, followed by stimulation with LPS for 6 h.

#### Analysis of real-time cell death

Real-time cell death assays were performed on an IncuCyte (Satorius, IncuCyte SX5). BMDMs were seeded in a 12-well plate and stimulated. Propidium iodide (PI; Invitrogen, P3566) was added at the time of stimulation for cell death analysis. Images (4 image fields per well) were acquired at 37 °C and 5% CO_2_, depending on the expected time frame for cell death. Subsequent image analysis was conducted using the software package supplied with the IncuCyte imager; the number of propidium iodide-positive cells (PI^+^ cells) present in each image was counted. Representative images were selected at the indicated time points.

#### High-throughput screening

A high-throughput screening (HTS) based on cell death induced by NLRP3 inflammasome activation was performed. Cell death triggered by LPS plus nigericin was screened against a custom library (TargetMol), which included inhibitors targeting more than 500 molecules across more than 100 signaling pathways, FDA-approved drugs, clinical trial drugs, and natural products. BMDMs were seeded at a density of 2 × 10^5^ cells per well into 96-well plates and treated with each library compound at 5 μM. The compounds were screened in duplicate, and real-time cell death assays were performed using the IncuCyte system. PI was added at the time of stimulation for cell death analysis. Subsequent image analysis was conducted as described in the previous section. Percent cell death was defined as the PI uptake in compound-treated cells normalized to the LPS- and nigericin-treated condition and expressed as a percentage; for visualization, data are shown on a logarithmic scale.

#### Knockdown via small interfering RNAs (siRNAs)

siRNAs specifically targeting *Pdia3* were chemically synthesized by Bionics, Korea. The sequences of si-*Pdia3* were as follows: sense, 5’-GUGAAGGAGUACGAUGAUA-3’; and antisense, 5’-UAUCAUCGUACUCCUUCAC-3’. A non-targeting siRNA was used as a control. BMDMs were transfected with these oligos for 24 h using the Neon^TM^ Transfection System (Invitrogen, MPK5000) according to the manufacturer’s instructions. After transfection, the cells were stimulated with LPS and ATP or LPS and nigericin as previously described.

#### Immunoblot analysis

Immunoblotting was performed as described previously (Karki et al, 2021b). For caspase analysis, cells were lysed along with the supernatant using 50 μL caspase lysis buffer (containing 1× protease inhibitors, 1× phosphatase inhibitors, 10% NP-40, and 25 mM DTT) followed by the addition of 100 μL 4× sample loading buffer (containing SDS and 2-mercaptoethanol). For analysis of LDH and HMGB1, centrifuged (8000 rpm for 4 min) supernatant 180 μL was combined with the sample loading buffer 60 μL. For analysis of signaling proteins, supernatants were removed at the indicated time points, and cells were washed once with PBS, after which cells were lysed with 150 µL RIPA buffer (containing 1× phosphatase inhibitor, protease inhibitor, 1% NP-40, 0.5% sodium deoxycholate) and 50 µL sample loading buffer. Proteins were separated by electrophoresis through 8–12% polyacrylamide gels. Following electrophoretic transfer of proteins onto PVDF membranes (Millipore, IPVH00010), non-specific binding was blocked by incubation with 5% skim milk in TBST; then membranes were incubated at 4 °C for overnight with the following primary antibodies: caspase-1 (AdipoGen, AG-20B-0042, 1:1000), human cleaved caspase-1 (Cell Signaling, 4199, 1:1000), human caspase-1 (Cell Signaling, 2225, 1:1000), caspase-3 (Cell Signaling, 9662, 1:1000), cleaved caspase-3 (Cell Signaling, 9661, 1:1000), caspase-7 (Cell Signaling, 9492, 1:1000), cleaved caspase-7 (Cell Signaling, 9491, 1:1000), caspase-8 (AdipoGen, AG-20T-0137-C100, 1:1000), cleaved caspase-8 (Cell Signaling, 8592, 1:1000), NEK7 (Abcam, ab133514, 1:1000), FLAG (Sigma, F1804, 1:5000), GSDMD (Abcam, ab209845, 1:1000), cleaved GSDMD (abcam, ab215203, 1:1000), LDHA (Proteintech, 19987-1-AP, 1:1000), ASC (AdipoGen, AG-25B-0006-C100, 1:1000), NLRP3/NALP3 (AdipoGen, AG-20B-0014-C100, 1:1000), Pyrin (abcam, AB195975, 1:1000), HMGB1 (abcam, ab18256, 1:1000), SAPK/JNK (Cell Signaling, 9252, 1:1000), phospho-SAPK/JNK (Cell Signaling, 9251, 1:1000), IκB-alpha (Cell Signaling, 9242, 1:1000), phospho-IκB-alpha (Cell Signaling, 2859, 1:1000), MAPK p44/42 (Cell Signaling, 9102, 1:1000), phospho-MAPK p44/42 (Cell Signaling, 9101, 1:1000), IRF1 (Cell Signaling, 8478, 1:1000), PDIA3 (Abclonal, A1085, 1:1000), IL-1β (Abclonal, A16288, 1:1000), GAPDH (CST, 5174S, 1:1000) and β-actin (Cell Signaling, 8457, 1:1000) antibodies. Membranes were then washed with TBST (10 min, 3 times) and incubated with appropriate secondary antibodies: HRP-conjugated anti-rabbit (Thermo Fisher Scientific, 31460, 1:5000) and HRP-conjugated anti-mouse (Cellnest, CNG004-0005, 1:5000) for 1 h, after which they were washed with TBST (10 min, 4 times). Proteins were visualized by using Immobilon Forte Western HRP Substrate (Millipore, WBLUF0500), and the membranes were analyzed using Amersham ImageQuant 800 UV (Bae et al, 2025).

#### Real-time PCR analysis

Total RNA from cells was extracted with TRIzol reagent (Invitrogen, 15596026) following the manufacturer’s instructions. Isolated RNAs were reverse transcribed with the M-MLV cDNA synthesis kit (Enzynomics, EZ006S) using the Applied Biosystems SimpliAmp thermal cycler (A24812) following the manufacturer’s instructions. Real-time qPCR was performed on QuantStudio 3 Real-Time PCR System by using TB green premix ex Taq (Takara, RR420) and ROX reference dye (Takara, AM21069A). Oligonucleotides used were as follows (Bionics): *Gapdh*: 5’-CGT CCC GTA GAC AAA ATG GT-3’(forward), 5’-TTG ATG GCA ACA ATC TCC AC-3’ (reverse); and *Nlrp3*: 5’-TCA GAT TGC TGT GTG TGG GAC TGA-3’ (forward), 5’-AGC TCA GAA CCA ATG CGA GAT CCT-3’ (reverse).

#### Generation of NLRP3 mutant plasmids

To generate NLRP3 plasmids harboring the G696A or R697A single-amino acid substitution, mutation-specific primers were designed. Mutation-specific oligonucleotides used were as follows (Bionics): NLRP3 G696A: 5’-GAA AAG GAA GCC CGA CAC-3’ (forward), 5’-TCG GGC TTC CTT TTC CT-3’ (reverse); NLRP3 R697A: 5’-GAA AAG GAA GGC GCA CAC C-3’ (forward), 5’-C CAT ATC AAG GTG TGC GCC T-3’ (reverse). Using the designed primers, a wild-type NLRP3 plasmid (Kim et al, 2025) as a template, and Herculase II Fusion DNA Polymerase (Agilent, 600677), PCR amplification was performed to generate fragments containing the desired single-point mutation. The resulting PCR products were then assembled into the vector using In-Fusion® Snap Assembly Master Mix (Takara, 638948) to generate recombinant plasmids. The assembly reaction products were transformed into *Escherichia coli* DH5α competent cells, which were subsequently plated on ampicillin-containing selection plates. Individual colonies were picked and subjected to mini-prep, and the presence of the intended mutations was confirmed by Sanger sequencing (Bionics). Through this procedure, NLRP3 plasmids containing the G696A or R697A single mutation were successfully generated.

#### Drug affinity responsive target stability (DARTS) assay

DARTS assay was carried out as described previously (Lomenick et al, 2009). BMDMs were primed with LPS (50 ng/mL) for 3 h. HEK293T cells were harvested 24 h after transfection with the indicated plasmids using Lipofectamine 3000 (Invitrogen, L3000015). Cells were lysed with NP-40 lysis buffer containing protease inhibitors. Lysates were centrifuged at 13,000 × *g* for 10 min at 4 °C, and the protein concentration was measured with a BCA Protein Assay Kit (Beyotime Biotechnology). Lysates were incubated with LOC14 at the indicated concentrations overnight at 4 °C with rotation. Then, the protease pronase (200 ng of enzyme per reaction, AbMole Bioscience) was added to the lysates and incubated for 15 min at room temperature. The reaction was stopped by the addition of 4× SDS loading buffer. The samples were then analyzed by immunoblotting.

#### NLRP3 target engagement assay

A NanoBRET^®^ probe was used to quantify engagement between the NLRP3 and LOC14 or control inhibitors (Teske et al, 2024). HEK293T cells were transiently transfected with plasmids encoding NLRP3-Nluc fusion proteins. After 24 h of transfection, HEK293T cells were treated with serially diluted NLRP3 NanoBRET^®^ tracer in the presence or absence of serially diluted LOC14, CRID3, or qVD (Selleckchem, S7311). After 2 h of equilibration, the NanoBRET^®^ Target Engagement substrate solution and the inhibitor solution were added one after the other, and Bioluminescence Resonance Energy Transfer (BRET) was recorded on a VICTOR Nivo Plate Reader.

#### U937 expressing NLRP3-AID-associated mutant assays

NLRP3-deficient U937 cells reconstituted with the doxycycline-inducible NLRP3 D303H or K568N constitutively active mutant have been previously described (Cosson et al, 2024). In total, 0.1 × 10^6^ cells/mL were plated in RPMI 1640 GlutaMaxTM-I supplemented with 1× penicillin and streptomycin and 10% FBS. The next day, cells were treated with LOC14 at concentrations of 20, 10, or 5 μM in a final DMSO concentration of 0.2% (for 20 μM). 15 min later, cells were treated with 2 μg/mL doxycycline (Sigma, D3447) before time-lapse imaging using a CQ1 high content screening microscope (Yokogawa) for 5 h. PI (1.25 μg/mL) and Hoechst (0.2 μg/mL) were added 1 h before imaging. 2 images per well were taken every 15 min for 5 h. Image analysis was performed as previously described (Cosson et al, 2024).

#### NLRP3 oligomerization assay

NLRP3 oligomerization was determined by using semi-denaturing detergent agarose gel electrophoresis (SDD-AGE). BMDM lysate was mixed with SDD-AGE loading dye (0.5% TAE, 5% glycerol, 2% sarcosyl, 0.1 mg/mL bromophenol blue, and protease inhibitor) without boiling. The capillary transfer method was used to transfer the proteins to the PVDF membrane as described previously (Hanna-Addams and Wang, 2018). Input lysate was boiled in 4× sample loading buffer (containing SDS and 2-mercaptoethanol) and run for SDS-PAGE.

#### ASC oligomerization assay

After stimulation with ATP or nigericin, BMDMs were rinsed in ice-cold PBS and then lysed with NP-40 buffer (20 mM HEPES-KOH, pH 7.5, 10 mM KCl, 1 mM EGTA, 1 mM EDTA, 320 mM sucrose, protease inhibitor) and incubated for 10 min on ice. Collected lysates were passed through a 21-gauge needle at least 10 times to further disrupt the cells and then incubated on ice for 5 min. 30 μL of cell lysate was used for input. The remaining lysates were centrifuged at 3400 × *g* for 15 min at 4 °C. After removing the supernatants, the pellets were washed once with PBS and centrifuged at 3400 × *g* for 15 min at 4 °C. The pellets were resuspended in 500 μL PBS containing 2 mM suberic acid bis (Sigma, S1885). The samples were incubated at RT for 1 h for cross-linking and centrifuged at 10,000 × *g* for 15 min at 4 °C. The supernatants were aspirated, and the pellets were resuspended with 35 μL of 2× loading dye. Both input and cross-linked samples were incubated at 95 °C for 5 min and analyzed by immunoblot (Zangiabadi et al, 2022).

#### ASC specks formation assay

After stimulation with LPS and nigericin, THP1-ASC-GFP cells were rinsed with ice-cold PBS. The cells were then fixed with 4% paraformaldehyde for 10 min at room temperature, followed by three washes with PBS. ASC specks were visualized by immunofluorescence microscopy.

#### Co-immunoprecipitation assay

THP1-ASC-GFP cells (1 × 10^7^ cells) were plated in 10 cm dishes and treated for 45 min as indicated. Following treatment, the cells were collected and washed once with ice-cold PBS. Cells were then lysed using NP-40 lysis buffer containing protease inhibitors. The cell lysates were centrifuged at 13,000 × *g* for 10 min at 4 °C. The supernatant was incubated overnight with 1 μg anti-NLRP3 (AG-20B-0014, Adipogen) or mouse IgG (Cell Signaling, 5415) antibody at 4 °C with rotation. After overnight incubation, protein A/G Agarose Resin (Yeason, 36403ES) was added to the lysates and further incubated for 1 h at 4 °C. The agarose resin was then washed five times with NP-40 lysis buffer. The samples were mixed with 2× SDS loading buffer and boiled at 100 °C for 5 min.

#### Mouse models of inflammatory diseases

##### LPS-induced systemic inflammation

7- to 8-week-old female WT mice were injected intraperitoneally with 20 mg/kg body weight of LPS (Sigma, L2630) (Karki et al, 2021a). Thirty minutes prior to the LPS injection, mice were administered intraperitoneally with either 200 μL of vehicle (10% DMSO in PBS, *n* = 10) or LOC14 (10 mg/kg body weight, *n* = 10). After 4 h, serum was collected, and IL-1β levels were measured by ELISA.

##### DSS-induced colitis

Colitis was induced in 7- to 8-week-old female WT mice using dextran sodium sulfate (DSS) as described previously (Karki et al, 2016). Mice were randomly divided into four groups: Normal (*n* = 7), DSS (*n* = 9), DSS + 5 mg/kg LOC14 (*n* = 9), and DSS + 10 mg/kg LOC14 (*n* = 9). LOC14 was administered via intraperitoneal injection daily for 9 days. Both the normal and DSS-only (Yeason, 60316ES25) groups received the same volume of DMSO. Body weight was monitored daily. On day 9, mice were sacrificed, colon length was measured, serum was collected, and colons were submitted for histological analysis.

##### IMQ-induced skin inflammation (psoriasis)

To induce psoriasis-like skin inflammation, mice were treated topically with Aldara (5% imiquimod cream; IMQ) on the shaved back skin (62.5 mg per mouse, Dong-A Science Technology) for 6 days. 7- to 8-week-old female WT mice were randomly divided into four groups: Normal (Vaseline, *n* = 3), IMQ (*n* = 7), IMQ + LOC14 (*n* = 15), and IMQ + CRID3 (*n* = 13). The normal group received the same amount of Vaseline. Starting one day after the IMQ application, mice were orally administered LOC14 (20 mg/kg) or CRID3 (200 mg/kg) daily. To score the severity of inflammation of the back skin, an objective scoring system was used based on the clinical psoriasis area and severity index (PASI) (van der Fits et al, 2009). Erythema, scaling, and thickening were scored independently on a scale from 0 to 4: 0, none; 1, slight; 2, moderate; 3, marked; 4, very marked. The cumulative score (erythema plus scaling plus thickening) served as a measure of the severity of inflammation (scale 0–12). Mice were sacrificed on day 7, and skin tissues were submitted for histological analysis or homogenized in RIPA lysis buffer.

#### Histological analysis

The back skin and colon were fixed in 4% formaldehyde (Cellnest, CNP015-1000). Tissue processing and hematoxylin and eosin (H&E) staining for skin and colon were performed by DKKorea and Wuhan Servicebio Technology Laboratory, respectively. The stained sections were digitized using a whole slide imaging system (Olympus VS200) at ×20 objective magnification with a consistent scanning setting across the same set of experiments.

#### Cytokine analysis

Cytokines were measured by performing ELISA for mouse IL-18 (Invitrogen, BMS618-3), mouse IL-1β (Invitrogen, 88-7013-88), and human IL-1β (ThermoFisher, BMS224-2TEN) according to the manufacturers’ instructions.

#### Docking analysis

##### Preparation of receptor and ligands

The structure of the NLRP3–NEK7 complex was retrieved from the Protein Data Bank (PDB) with the ID 6NPY (Data ref: (Sharif et al, 2019). The Protein Preparation Wizard tool was utilized to assign hydrogen atoms and charges. The OPLS_2005 force field was used to optimize ionization and tautomeric states (Jorgensen et al, 1996). The 3D structure of LOC14 (PubChem ID: 9117962) was downloaded as a structure data file (SDF). LigPrep (Schrödinger Release 2024-2) was applied to verify chiral centers. The NLRP3 protein consists of 2 chains. Chain A includes the NACHT, LRR, and PYD domains, while Chain B comprises the Serine/threonine-protein kinase NEK7. To investigate the interaction between NLRP3 and NEK7, the Receptor Grid Generation tool was used to define the active site. The active site of NLRP3 included P134, E135, A614, K615, A616, K617, K618, L619, Q620, I621, Q622, P623, S624, Q625, E627, L628, F629, Y630, C631, L632, Q636, E637, E638, D639, A644, M645, K694, E695, G696, R697, H698, L699, N720 residues. For NEK7, the active site included residues D261, H262, Y263, S264, C298, and T299. The receptor-ligand docking study was conducted using the Glide (grid-based ligand docking with extra precision (XP)) tool from the Schrödinger molecular modeling package (Halgren et al, 2004).

##### Molecular dynamic simulation

The Molecular Dynamics (MD) simulation of the NLRP3–NEK7–LOC14 complex was conducted using the Desmond package (Schrödinger Release 2024-2) for a duration of 1000 ns. The complex was prepared using a protein preparation wizard, which involved inserting hydrogen atoms, removing water molecules, assigning bond orders, and completing missing side chains and loops. Hydrogen-bond assignments were optimized at a pH of 7.0, and water orientations were sampled. Energy minimization of the protein-ligand complexes was performed using the OPLS-2005 force field (Price and Brooks, 2004).

The system was assembled using the TIP3P solvent model, creating a 10 Å buffer around the complex in an orthorhombic simulation box. Na^+^ ions and Cl^–^ counter ions were added to achieve a physiological salt concentration of 0.15 M and to neutralize the system. The MD simulation was run under an NPT (constant Number of particles, Pressure, and Temperature) ensemble at a temperature of 300 K and a pressure of 1.013 bar (Patel et al, 2023). Surface tension was calculated using the Smooth Particle Mesh Ewald (PME) method (Essmann et al, 1995) to account for long-range electrostatic interactions, with the RESPA integrator used for potential energy calculations (Wang et al, 2006).

The simulation was performed for 1000 ns, capturing 1000 frames throughout the duration. The resulting trajectories were analyzed using the Simulation Interaction Diagram wizard to calculate Root Mean Square Deviation (RMSD) and Root Mean Square Fluctuation (RMSF) values (Zhang and Lazim, 2017). Protein-ligand contact patterns and the duration of interactions for key amino acid residues were assessed over the simulation period. The MD simulation approach was used to validate the docking postures and the interactions predicted by both ligands with the NLRP3 protein.

##### Binding free energy calculations

The Gibbs free energy change was performed using the Molecular Mechanics Generalized Born Surface Area (MM-GBSA) method to analyze the interaction between the LOC14 and NLRP3–NEK7 protein complex (Hou et al, 2011; Massova and Kollman, 2000). The initial step involved conducting an MD simulation to generate an output file containing the trajectories and conformations of the protein-ligand complex. The MD stimulation output file was input into Schrödinger Maestro’s Prime wizard for further optimization. The optimized structures were used to calculate the binding free energy using the OPLS-2005 force field. The change in free energy upon binding (∆G) was determined by the following equation:

$${\Delta {{\rm{G}}}}_{{{\rm{Bind}}}}={\Delta {{\rm{E}}}}_{{{\rm{MM}}}}+{\Delta {{\rm{G}}}}_{{{\rm{Solv}}}}+{\Delta {{\rm{G}}}}_{{{\rm{SA}}}}$$

Where:

∆G_Bind:_ Molar Gibbs free energy change associated with the binding of the receptor and ligand in solution.

∆E_MM:_ Difference in the molecular mechanics energy between the minimal energy states of the protein-ligand complex.

∆G_Solv:_ Total solvation energy, comprising the solvation energies of the protein and ligand and the change in GBSA solvation energy.

∆G_SA:_ Difference in surface area energies between the free and bound states.

#### Statistical analysis

Formal allocation randomization was not conducted because the study did not involve randomized clinical procedures. Blinding of investigators was not implemented during either data collection or analysis. Experiments were independently repeated for each assay where applicable, and the replicate numbers (*n*) are indicated in the respective figure legends. GraphPad Prism 10.0 software was used for data analysis. The data are presented as the mean ± SEM. Statistical significance was determined using a *t* test (two-tailed) or one-way ANOVA or two-way ANOVA for comparisons among multiple groups. When one-way ANOVA was applied, Dunnett’s or Tukey’s multiple comparisons tests were used for post hoc analysis. When two-way ANOVA was applied, Tukey’s or Sidak’s multiple comparisons tests were used for post hoc analysis. *P* values less than 0.05 were considered statistically significant and are indicated as *P < 0.05, **P < 0.01, ***P < 0.001, and ****P < 0.0001.

#### Graphics

The synopsis image was created with Biorender.com.

## Supplementary information

Appendix

Peer Review File

Source data Fig. 1

Source data Fig. 2

Source data Fig. 3

Source data Fig. 4

Source data Fig. 5

Source data Fig. 6

Source data Fig. 7

Source data Fig. 8

Expanded View Figures

## Data Availability

This study includes no data deposited in external repositories. The source data of this paper are collected in the following database record: biostudies:S-SCDT-10_1038-S44321-026-00425-5.
