# Supplementary material for: Pharmacological targeting of the NLRP3 LRR domain with isothiazolinones overcomes CRID3-resistant inflammation
Source: EMBO Mol Med. 2026 Apr 17;18(6):2124–51. doi: 10.1038/s44321-026-00425-5 (PMC13269794; doi:10.1038/s44321-026-00425-5)
Supplement: Supplementary file 1 — Appendix [file 44321_2026_425_MOESM1_ESM.pdf]

Appendix

Pharmacological Targeting of the NLRP3 LRR Domain with Isothiazolinones Overcomes CRID3-Resistant Inflammation

Hawon Woo[1*], Yeonseo Jang[1*], Soyeon Kim[1], Wonyoung Kim[1], Fenfen Zhang[2], Raghvendra Mall[3], Chirag N. Patel[3,4], Melan Kurera[5], Chinh Ngo[5], Simon H. Jiang[5], Asia Nicotra[6], Bénédicte F. Py[6], Min Zheng[2**], Si Ming Man[5**], Rajendra Karki[1**]

Table of Contents

Appendix Figure S1 2

Appendix Figure S2 3

Appendix Figure S3 4

Appendix Figure S4 5

Appendix Figure S5 6

Appendix Figure S6 7

Appendix Figure S7 8

Appendix Figure S8 9

Appendix Figure S9 10

Appendix Figure S10 11

Appendix Figure S11 12

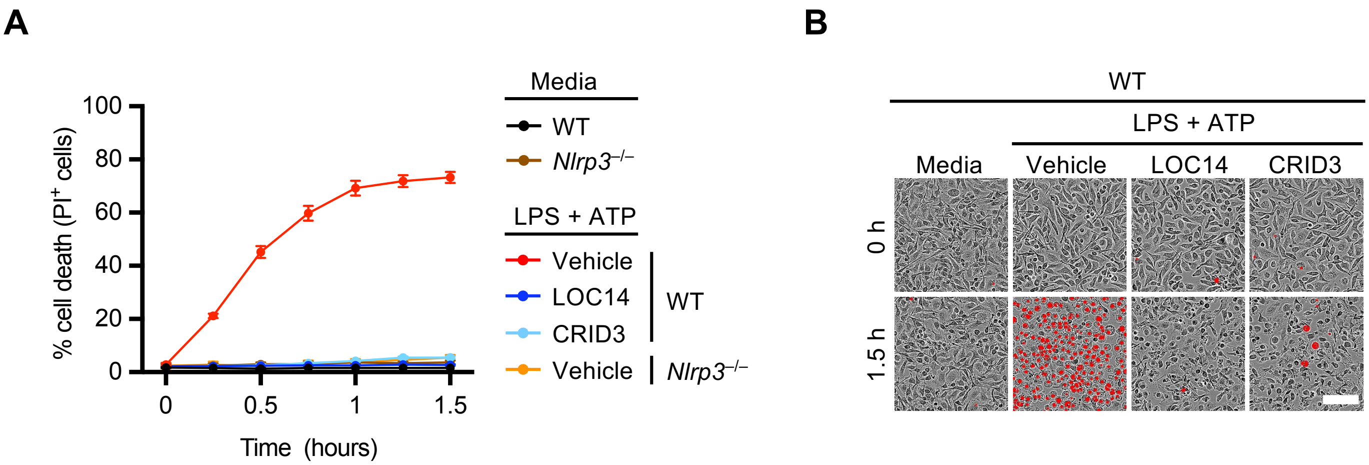

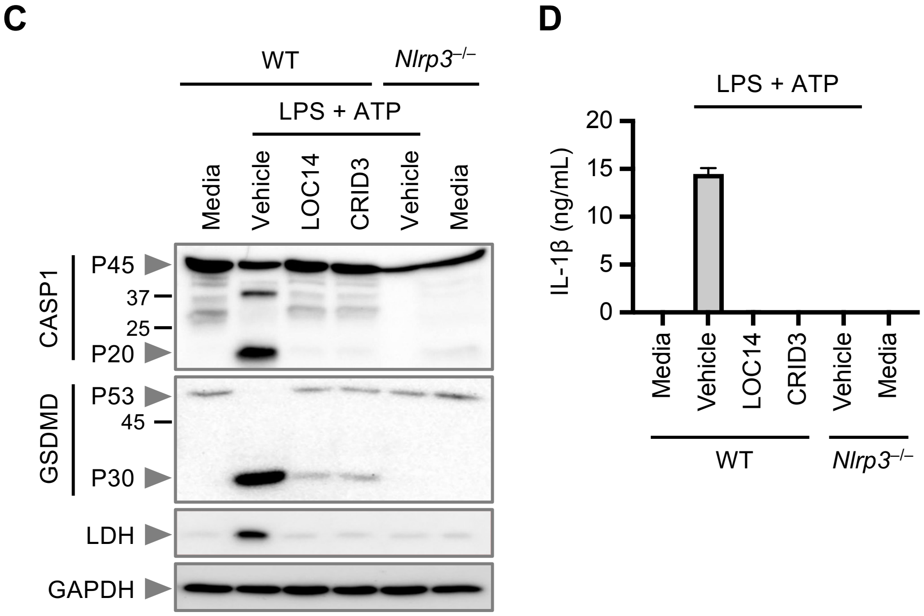

E

IC50 (μM)	LPS + Nigericin	
	Cell death	IL-1β release
LOC14	1.551	0.9808
CRID3	0.6307	0.3538

Appendix Figure S1 – LOC14 inhibits the canonical NLRP3 inflammasome.

A–D Real-time analysis (**A**) and representative images of cell death at 0 h and 1.5 h (**B**), and immunoblot analysis of pro- (P45) and cleaved caspase-1 (CASP1; P20), pro- (P53) and cleaved gasdermin D (GSDMD; P30), and LDH (**C**), and measurement of IL-1β release (**D**) in wild-type (WT) and *Nlrp3*$^{-/-}$ bone marrow-derived macrophages (BMDMs) primed with LPS for 4 h and subsequently stimulated with ATP for 1.5 h, with or without LOC14 or CRID3. **E** IC$_{50}$ values of LOC14 and CRID3 inhibiting cell death and IL-1β release induced by LPS plus nigericin for 1 h. Scale bar, 100 μm (B). GAPDH was used as an internal control (**C**). Data are representative of at least three independent experiments (**A–D**) or two independent experiments (**E**). Data are shown as mean ± SEM (**A, D**).

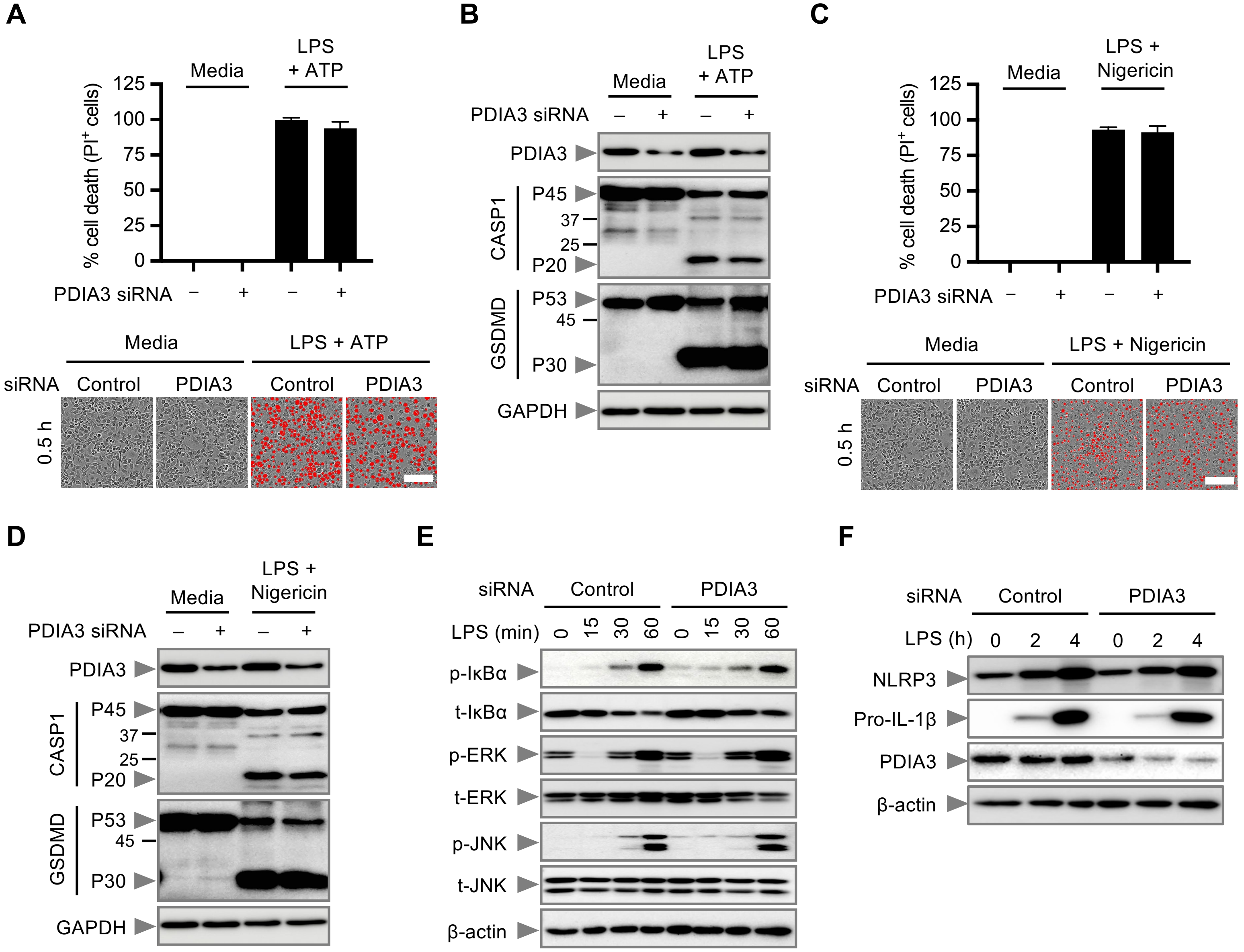

Appendix Figure S2 – PDIA3 is not required for NLRP3 inflammasome activation.

A, B Percentage of cell death and representative images (**A**), and immunoblot analysis of PDIA3, pro- (P45) and cleaved caspase-1 (CASP1; P20), pro- (P53) and cleaved gasdermin D (GSDMD; P30) (**B**) in PDIA3- or control siRNA-transfected wild-type (WT) bone marrow-derived macrophages (BMDMs) primed with LPS for 4 h and subsequently stimulated with ATP for 0.5 h. **C, D** Percentage of cell death and representative images (**C**), and immunoblot analysis of PDIA3, pro- (P45) and cleaved CASP1 (P20), pro- (P53) and cleaved GSDMD (P30) (**D**) in PDIA3- or control siRNA-transfected BMDMs primed with LPS for 4 h and subsequently stimulated with nigericin for 0.5 h. **E, F** Immunoblot analysis of phosphorylated IκBα (p-IκBα), total IκBα (t-IκBα), phosphorylated ERK1/2 (p-ERK), total ERK1/2 (t-ERK), phosphorylated JNK (p-JNK), and total JNK (t-JNK) (**E**) and NLRP3, pro-IL-1β, and PDIA3 (**F**) in LPS-stimulated WT BMDMs upon transfection with PDIA3 or control siRNA. Scale bars, 100 µm (**A, C**). GAPDH (**B, D**) and β-actin (**E, F**) were used as internal controls. Data are representative of at least three independent experiments. Data are shown as mean ± SEM (**A, C**).

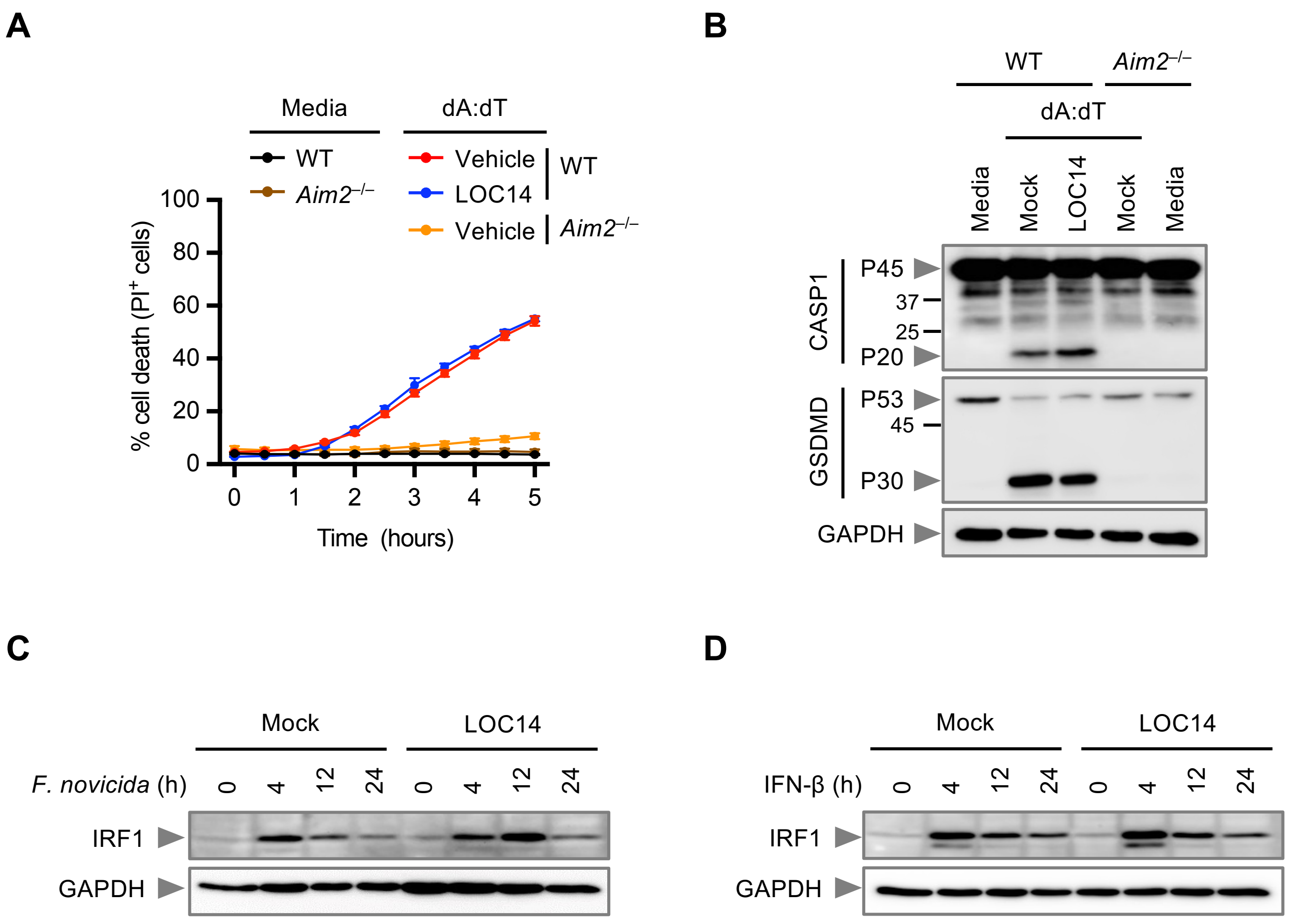

Appendix Figure S3 – LOC14 does not affect AIM2 inflammasome activation or IRF1 induction.

A, B Real-time analysis of cell death (**A**) and immunoblot analysis of pro- (P45) and cleaved caspase-1 (CASP1; P20), pro- (P53) and cleaved gasdermin D (GSDMD; P30) (**B**) in wild-type (WT) and *Aim2*$^{-/-}$ bone marrow-derived macrophages (BMDMs) transfected with poly(dA:dT) for 4 h, with or without LOC14. **C, D** Immunoblot analysis of IRF1 in WT BMDMs infected with 100 MOI of *Francisella novicida* (**C**) and stimulated with IFN-β (**D**) in the presence of LOC14 for the indicated times. GAPDH was used as an internal control (**B–D**). Data are representative of at least three independent experiments. Data are shown as mean ± SEM (**A**).

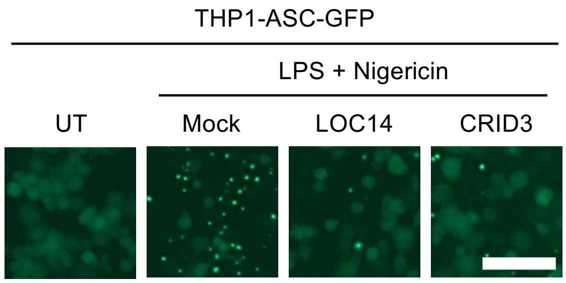

Appendix Figure S4 – LOC14 reduces ASC speck formation in LPS-primed THP1-ASC-GFP cells.

Representative fluorescence microscopy images of THP1-ASC-GFP cells primed with LPS for 1 h and subsequently stimulated with nigericin for 1 h, with or without LOC14 or CRID3. Scale bar, 50 μm. Data are representative of at least three independent experiments.

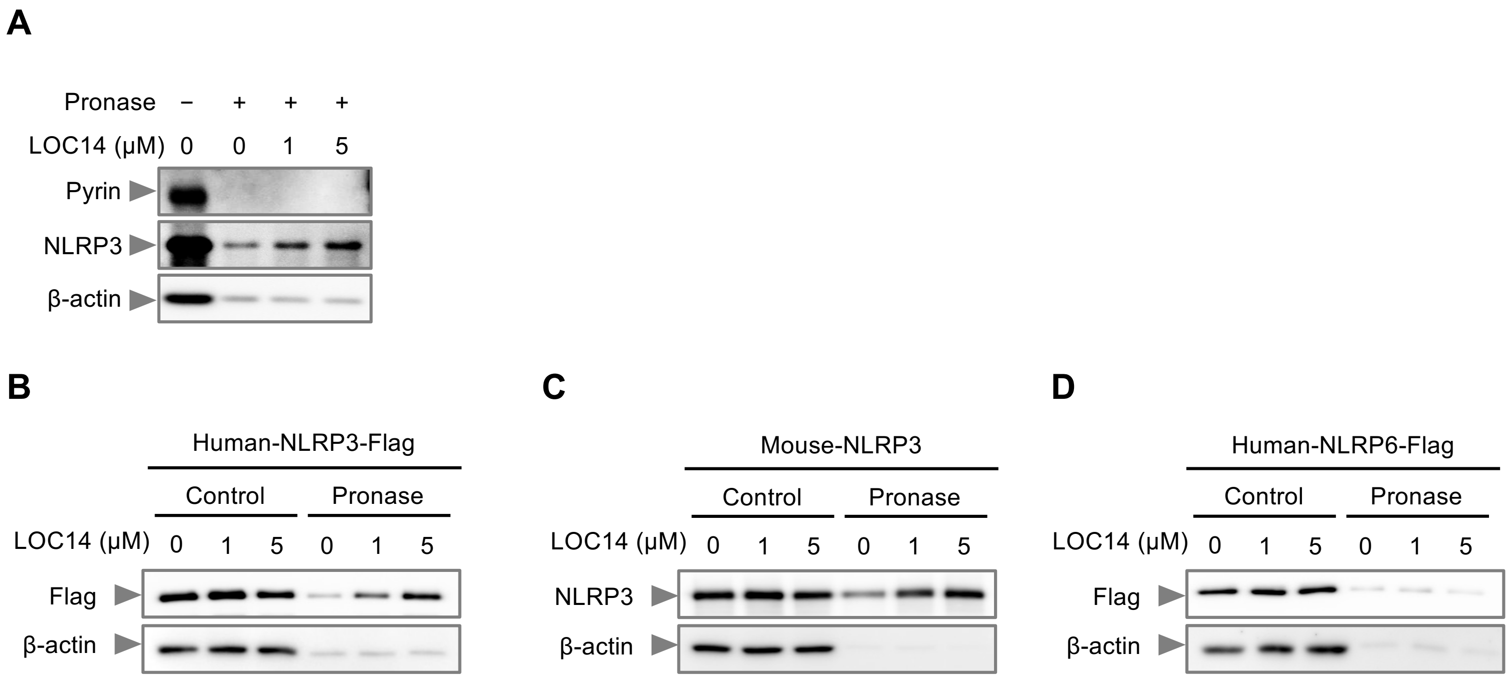

Appendix Figure S5 – LOC14 selectively protects NLRP3 from proteolysis.

A Immunoblot analysis of endogenous NLRP3 and Pyrin in bone marrow–derived macrophages (BMDMs) treated with LOC14 and subjected to pronase digestion. **B–D** Immunoblot analysis of human NLRP3-Flag (**B**), mouse NLRP3 (**C**), or human NLRP6-Flag (**D**) expressed in HEK293T cells, treated with or without LOC14 and pronase. β-actin was used as an internal control. Data are representative of at least two independent experiments.

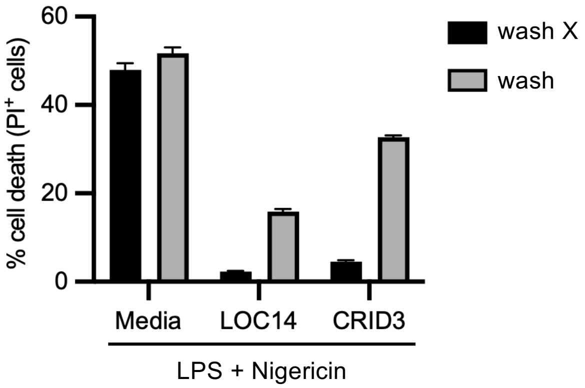

Appendix Figure S6 – Washout analysis of LOC14-mediated inhibition of NLRP3–dependent cell death.

Wild-type (WT) bone marrow–derived macrophages (BMDMs) were primed with LPS for 4 h and treated with the indicated compounds for 30 min. For washout conditions (wash), cells were washed three times at 5 min intervals after compound treatment and then stimulated with nigericin. For non-wash conditions (wash X), cells were not washed after compound treatment and stimulated with nigericin at the same time point as the washout condition. Data are representative of at least three independent experiments. Data are shown as mean ± SEM.

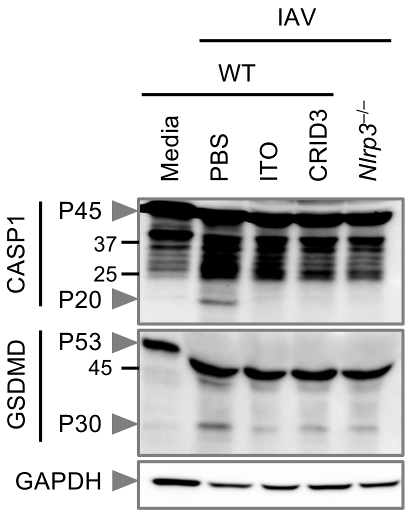

Appendix Figure S7 – ITO suppresses CASP1 activation and GSDMD cleavage in IAV-infected macrophages in an NLRP3–dependent manner

Immunoblot analysis of pro- (P45) and cleaved caspase-1 (CASP1; P20), pro- (P53) and cleaved gasdermin D (GSDMD; P30) in influenza A virus (IAV)-infected wild-type (WT) and *Nlrp3*$^{-/-}$ bone marrow-derived macrophages (BMDMs) treated with or without isothiazol-3(2H)-one (ITO) or CRID3 for 16 h. GAPDH was used as an internal control. Data are representative of at least three independent experiments.

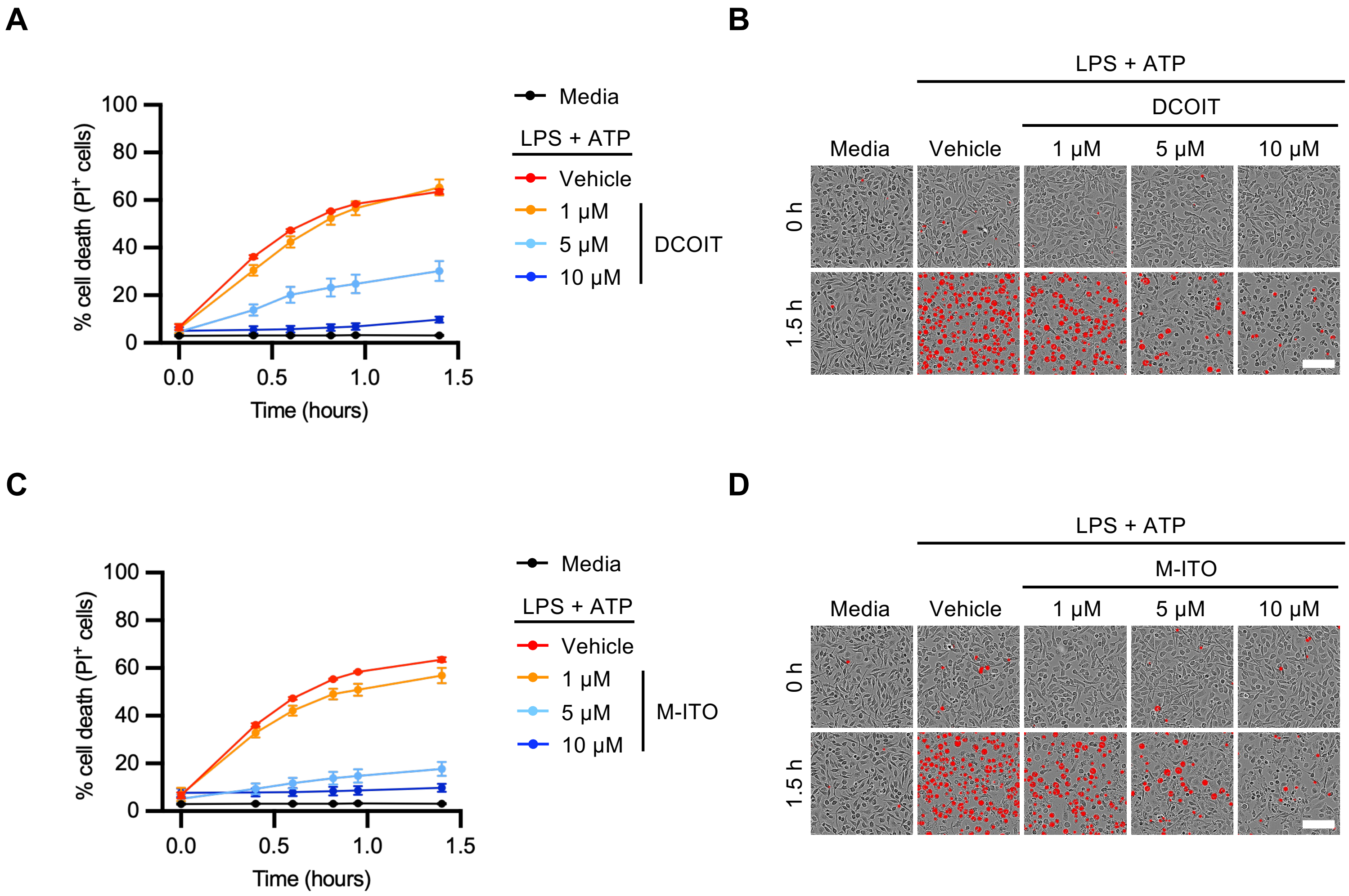

Appendix Figure S8 – Isothiazolinone-containing compounds inhibit NLRP3 inflammasome–mediated cell death

A–D Real-time analysis (**A, C**) and representative images of cell death at 0 h and 1.5 h (**B, D**) in wild-type (WT) bone marrow-derived macrophages (BMDMs) primed with LPS for 4 h and subsequently stimulated with ATP for 1.5 h, with or without 4,5-dichloro-2-n-octyl-4-isothiazoline-3-one (DCOIT) (**A**, **B**) or methylisothiazolinone (M-ITO) (**C**, **D**). Scale bars, 100 μm (**B, D**). Data are representative of at least three independent experiments. Data are shown as mean ± SEM (**A, C**).

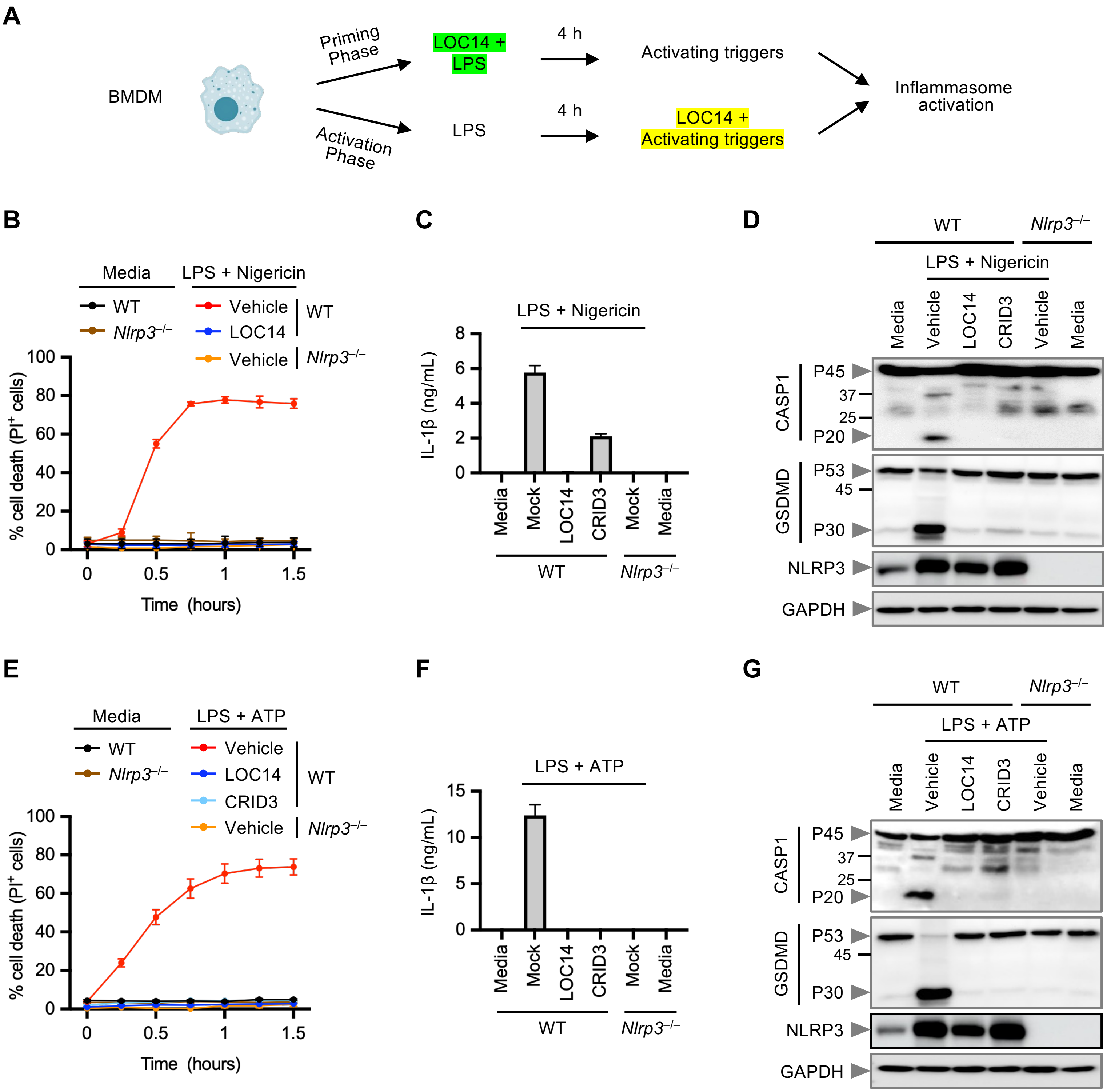

Appendix Figure S9 – LOC14 inhibits NLRP3 inflammasome priming.

A Schematic diagram showing LOC14 treatment during priming or activation events of NLRP3 inflammasome activation. **B–D** Real-time analysis of cell death (**B**), IL-1β release (**C**), and immunoblot analysis of pro- (P45) and cleaved caspase-1 (CASP1; P20), pro- (P53) and cleaved gasdermin D (GSDMD; P30), and NLRP3 (**D**) in wild-type (WT) and *Nlrp3*$^{-/-}$ bone marrow–derived macrophages (BMDMs) co-treated with LPS and LOC14 or CRID3 for 4 h, followed by nigericin stimulation. **E–G** Real-time analysis of cell death (**E**), IL-1β release (**F**), and immunoblot analysis of pro- (P45) and cleaved CASP1 (P20), pro- (P53) and cleaved GSDMD (P30), and NLRP3 (**G**) in WT and *Nlrp3*$^{-/-}$ BMDMs co-treated with LPS and LOC14 or CRID3 for 4 h, followed by ATP stimulation. GAPDH was used as an internal control (**D, G**). Data are representative of at least three independent experiments (**B–G)**. Data are shown as mean ± SEM (**B, C, E, F**).

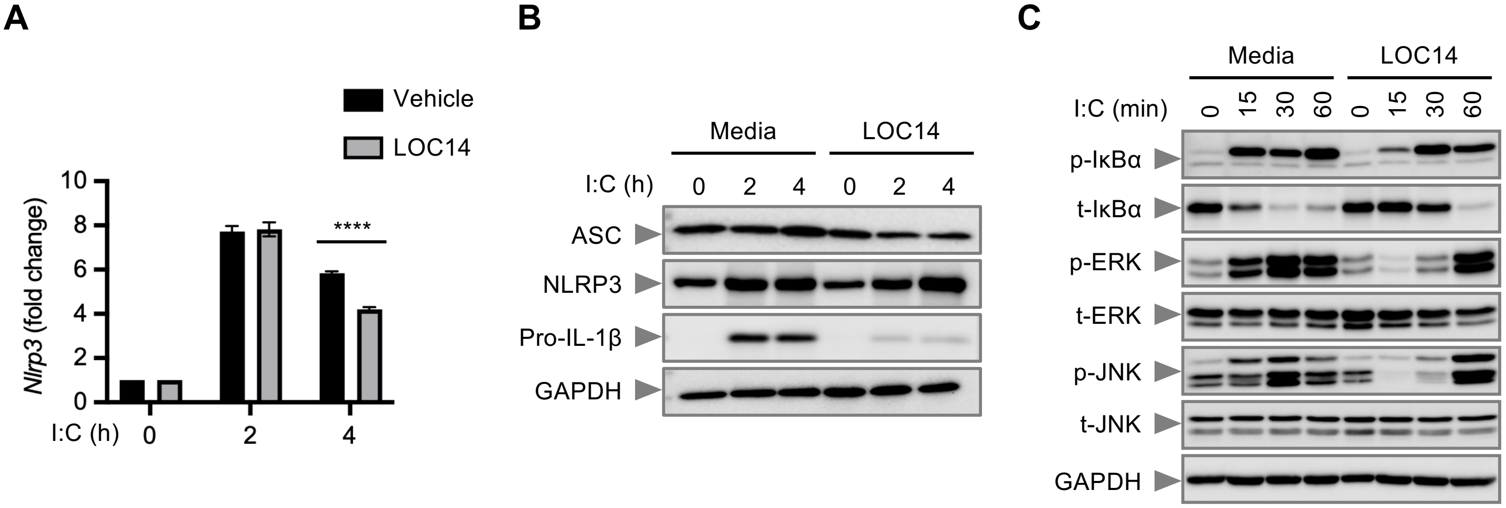

Appendix Figure S10 – LOC14 delays priming of NLRP3.

A Real-time PCR analysis of *Nlrp3* expression in wild-type (WT) bone-marrow-derived macrophages (BMDMs) stimulated with Poly I:C (I:C) in the presence of LOC14 for the indicated times. Significance was evaluated using two-way ANOVA followed by Sidak's multiple comparisons test. ****$P < 0.0001$. Vehicle vs LOC14 at 4 h, $P = 6.8E\text{-}5$. **B, C** Immunoblot analysis of ASC, NLRP3, and pro-IL-1β (**B**) and phosphorylated IκBα (p-IκBα), total IκBα (t-IκBα), phosphorylated ERK1/2 (p-ERK), total ERK1/2 (t-ERK), phosphorylated JNK (p-JNK), and total JNK (t-JNK) (**C**) in WT BMDMs stimulated with I:C in the presence of LOC14 for the indicated times. *Gapdh* (**A**) and GAPDH (**B**, **C**) were used as internal controls. Data are representative of at least three independent experiments. Data are shown as mean ± SEM (**A**).

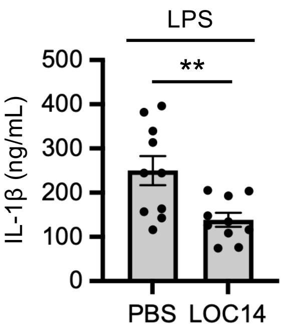

Appendix Figure S11 – LOC14 suppresses LPS-induced IL-1β in mice.

Levels of IL-1β in the serum of wild-type (WT) mice administered with vehicle or LOC14, after 4 h of LPS injection. Each symbol represents an individual mouse. Significance was evaluated using two-tailed *t*-test. $**P < 0.01$. PBS vs LOC14, $P = 0.009$. Data are shown as mean ± SEM. Data are representative of two independent experiments.
