# Supplementary figures and images for: Pharmacological targeting of the NLRP3 LRR domain with isothiazolinones overcomes CRID3-resistant inflammation (part 1 of 2)
Source: EMBO Mol Med. 2026 Apr 17;18(6):2124–51. doi: 10.1038/s44321-026-00425-5 (PMC13269794; doi:10.1038/s44321-026-00425-5)

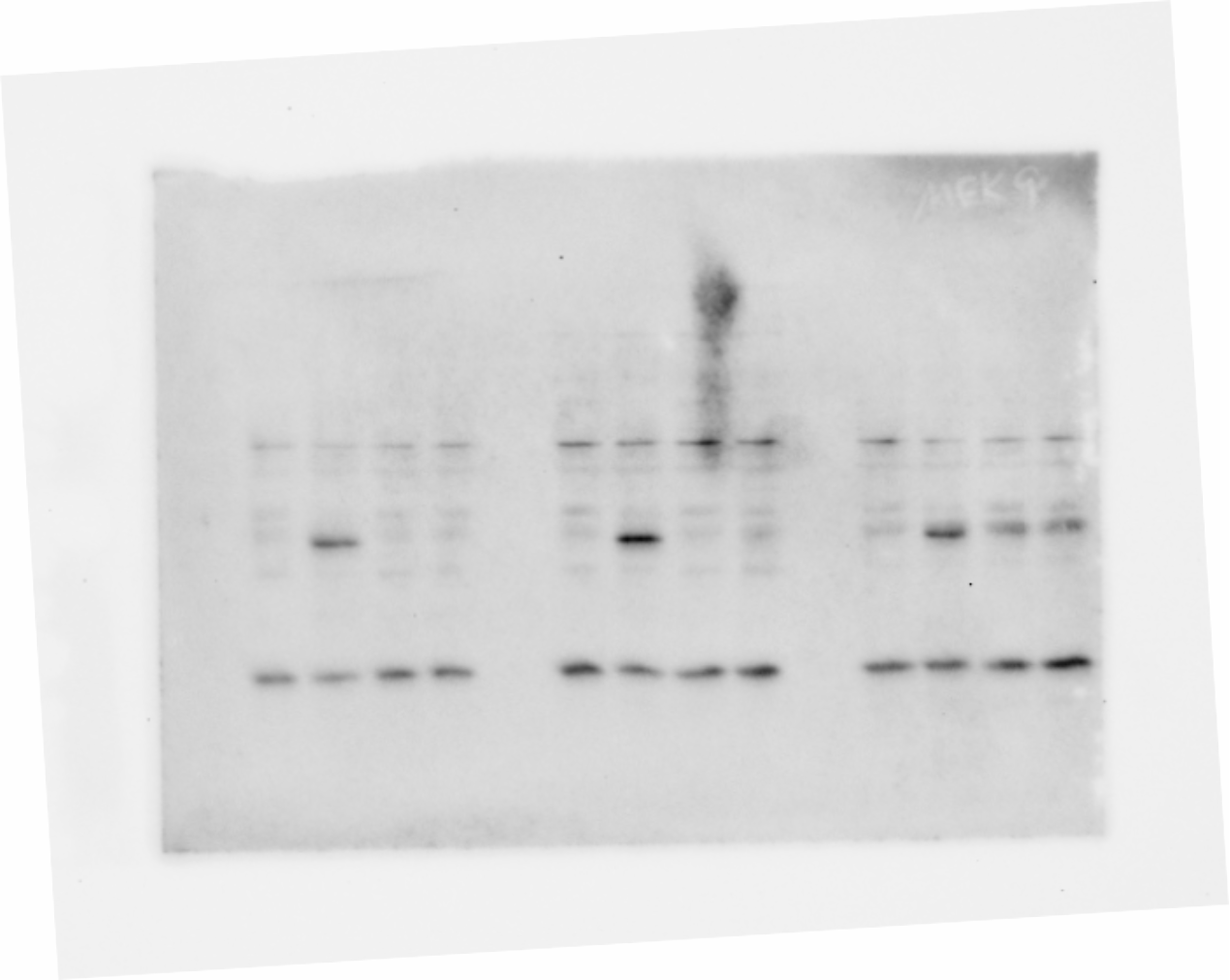

Supplement: Supplementary file 3 — Source data Fig. 1 [file 44321_2026_425_MOESM3_ESM.zip › Figure 1 Source Data/1H/1H_GSDMD.tif]

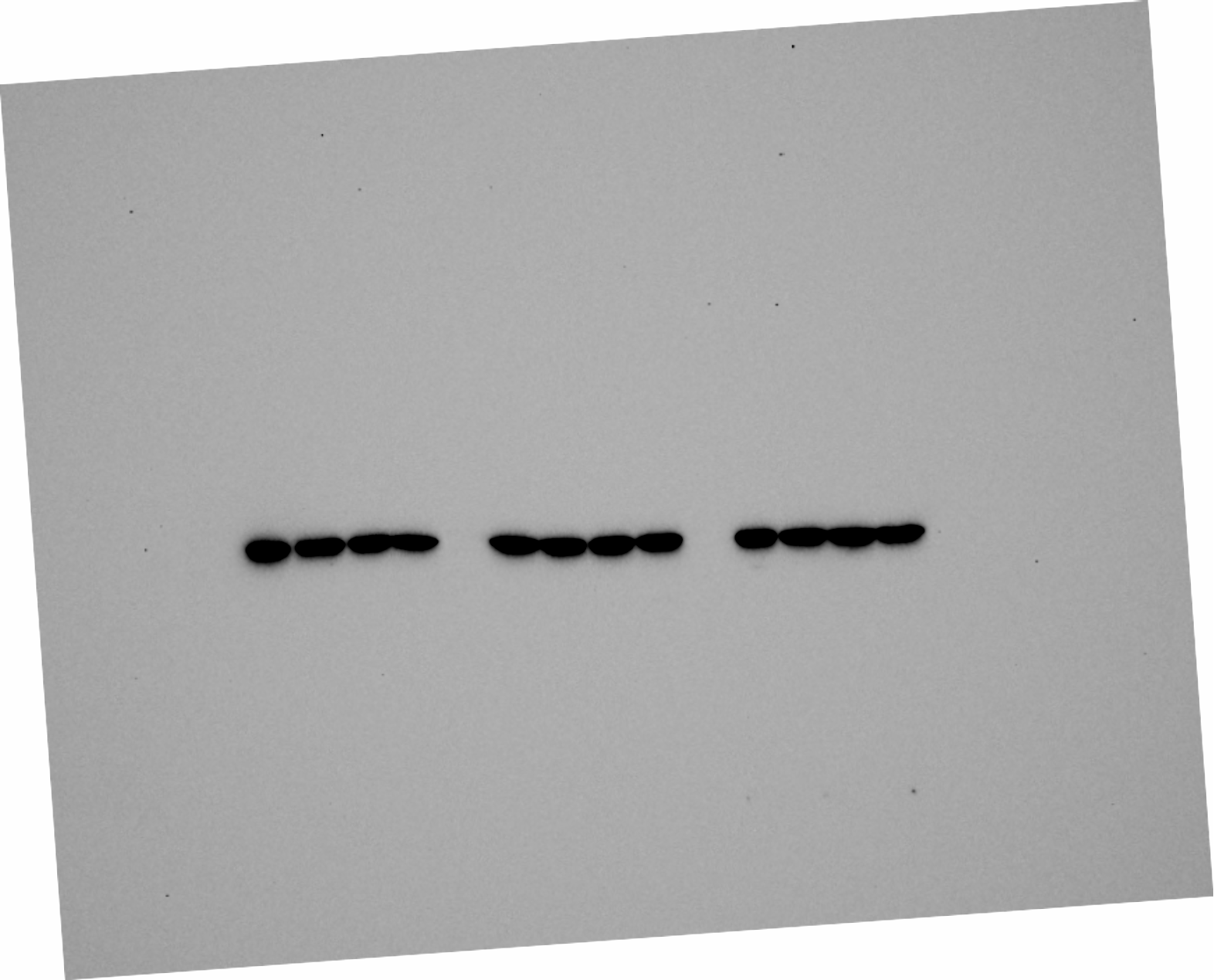

Supplement: Supplementary file 3 — Source data Fig. 1 [file 44321_2026_425_MOESM3_ESM.zip › Figure 1 Source Data/1H/1H_b-actin.tif]

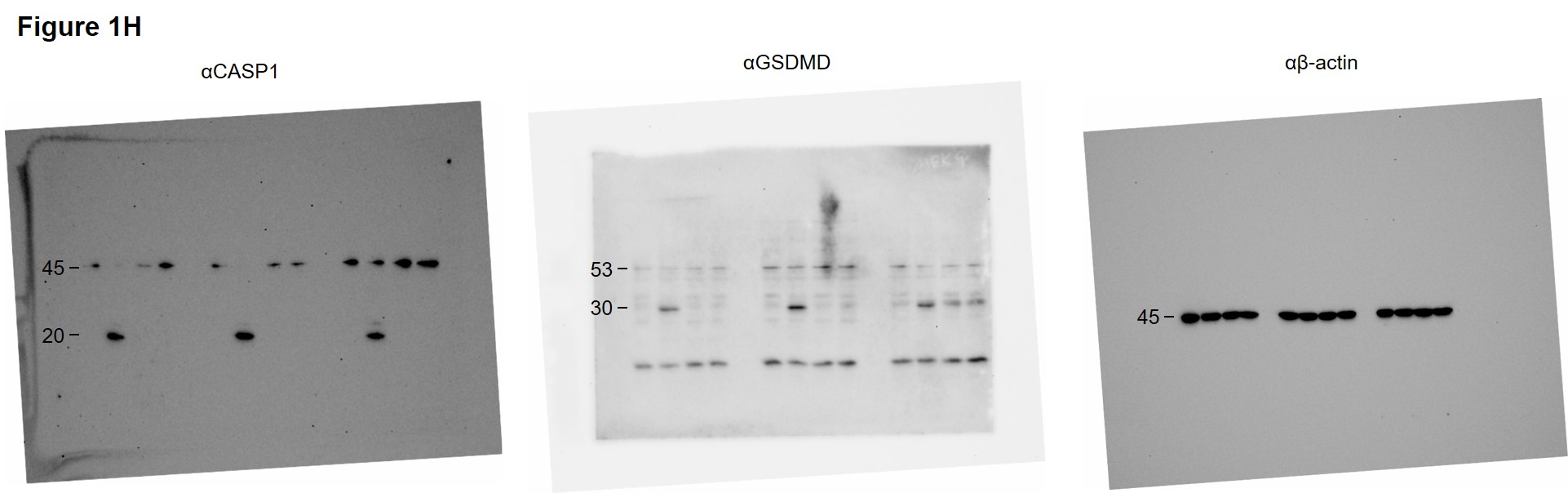

Supplement: Supplementary file 3 — Source data Fig. 1 [file 44321_2026_425_MOESM3_ESM.zip › Figure 1 Source Data/1H/1H.jpg]

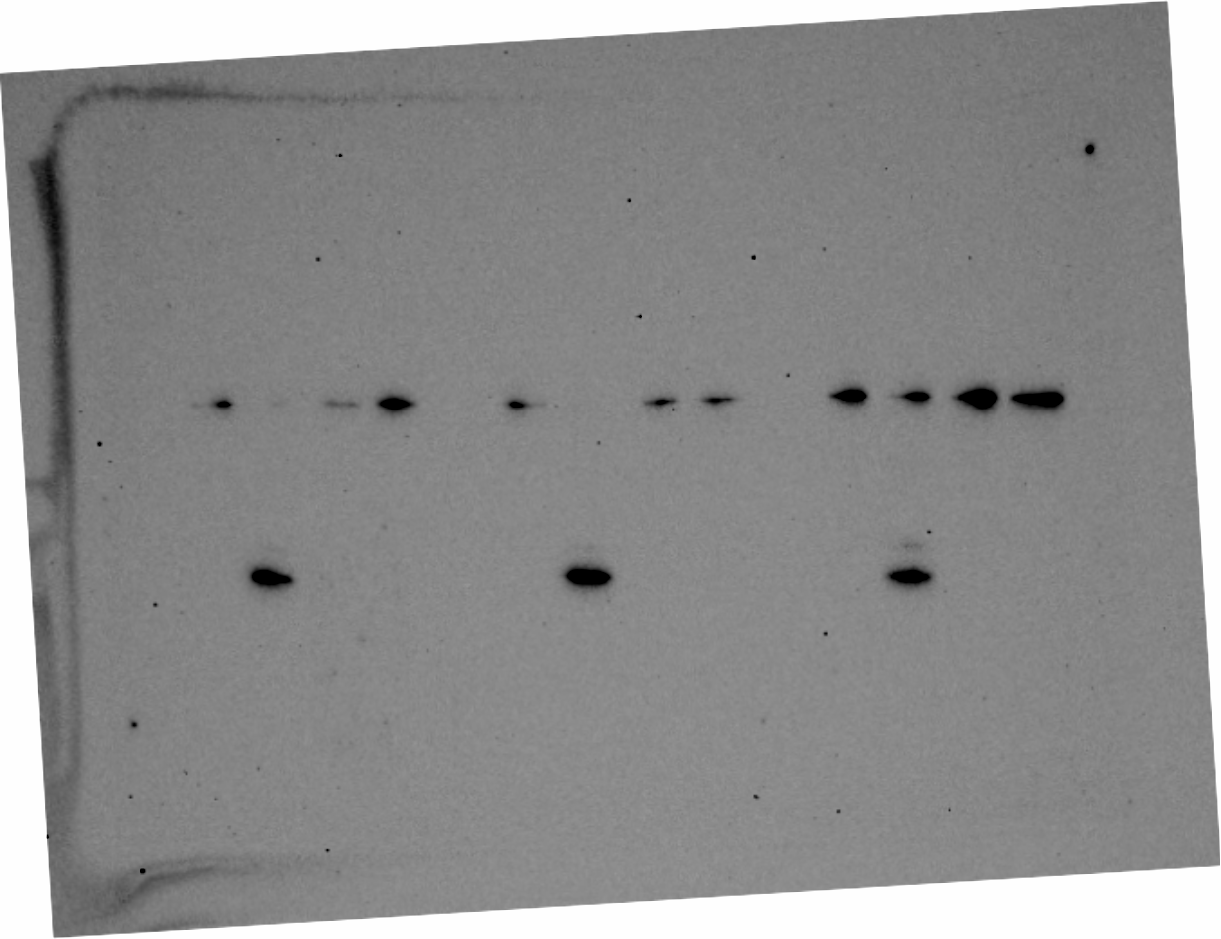

Supplement: Supplementary file 3 — Source data Fig. 1 [file 44321_2026_425_MOESM3_ESM.zip › Figure 1 Source Data/1H/1H_CASP1.tif]

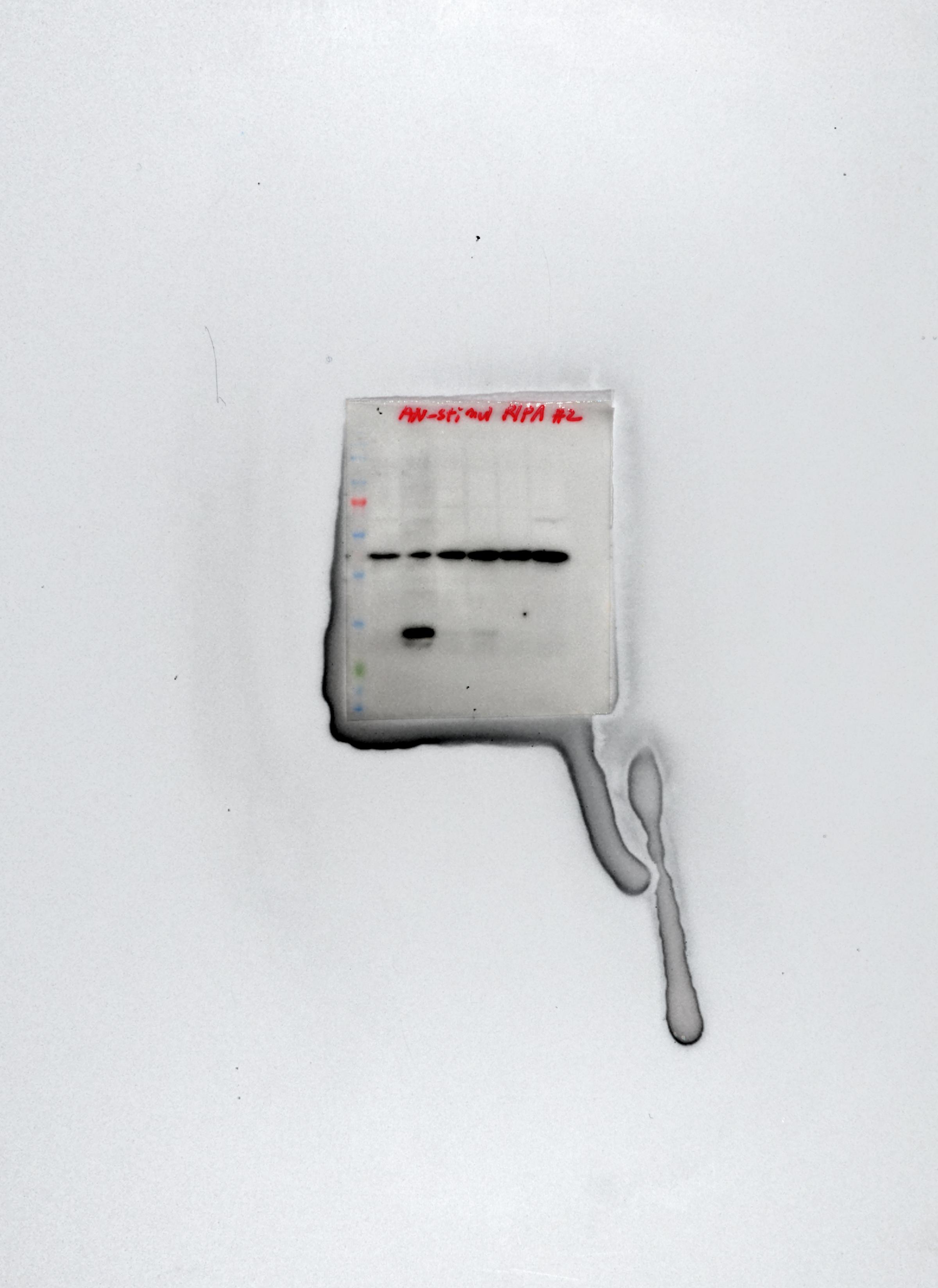

Supplement: Supplementary file 3 — Source data Fig. 1 [file 44321_2026_425_MOESM3_ESM.zip › Figure 1 Source Data/1E/1E_GSDMD.jpg]

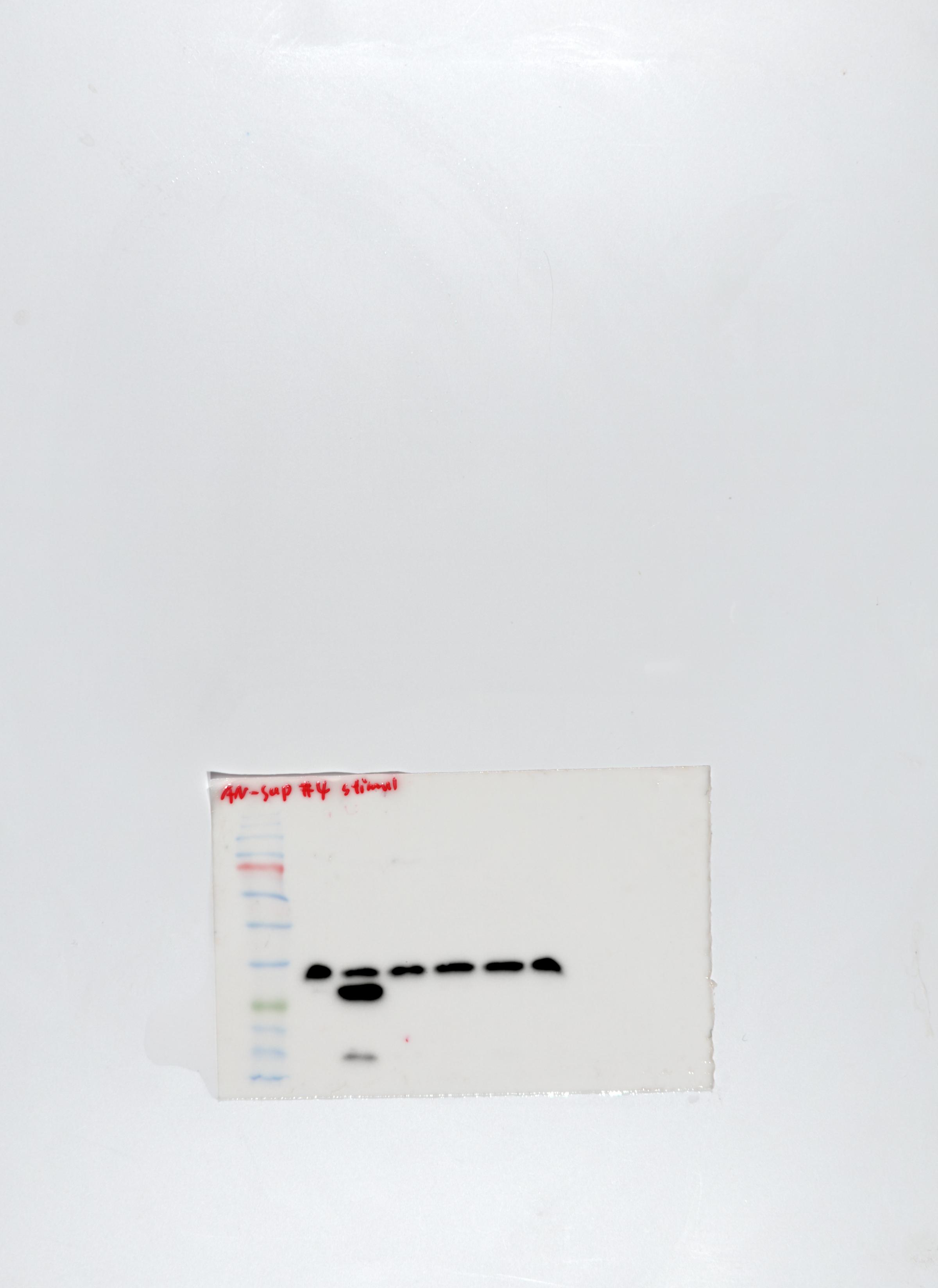

Supplement: Supplementary file 3 — Source data Fig. 1 [file 44321_2026_425_MOESM3_ESM.zip › Figure 1 Source Data/1E/1E_HMGB1.jpg]

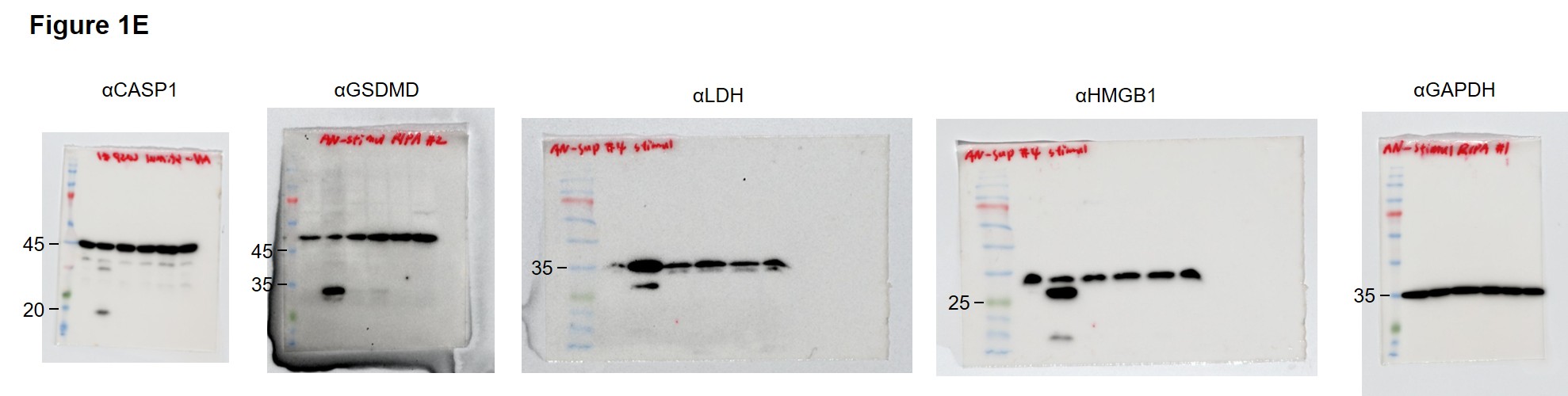

Supplement: Supplementary file 3 — Source data Fig. 1 [file 44321_2026_425_MOESM3_ESM.zip › Figure 1 Source Data/1E/1E.jpg]

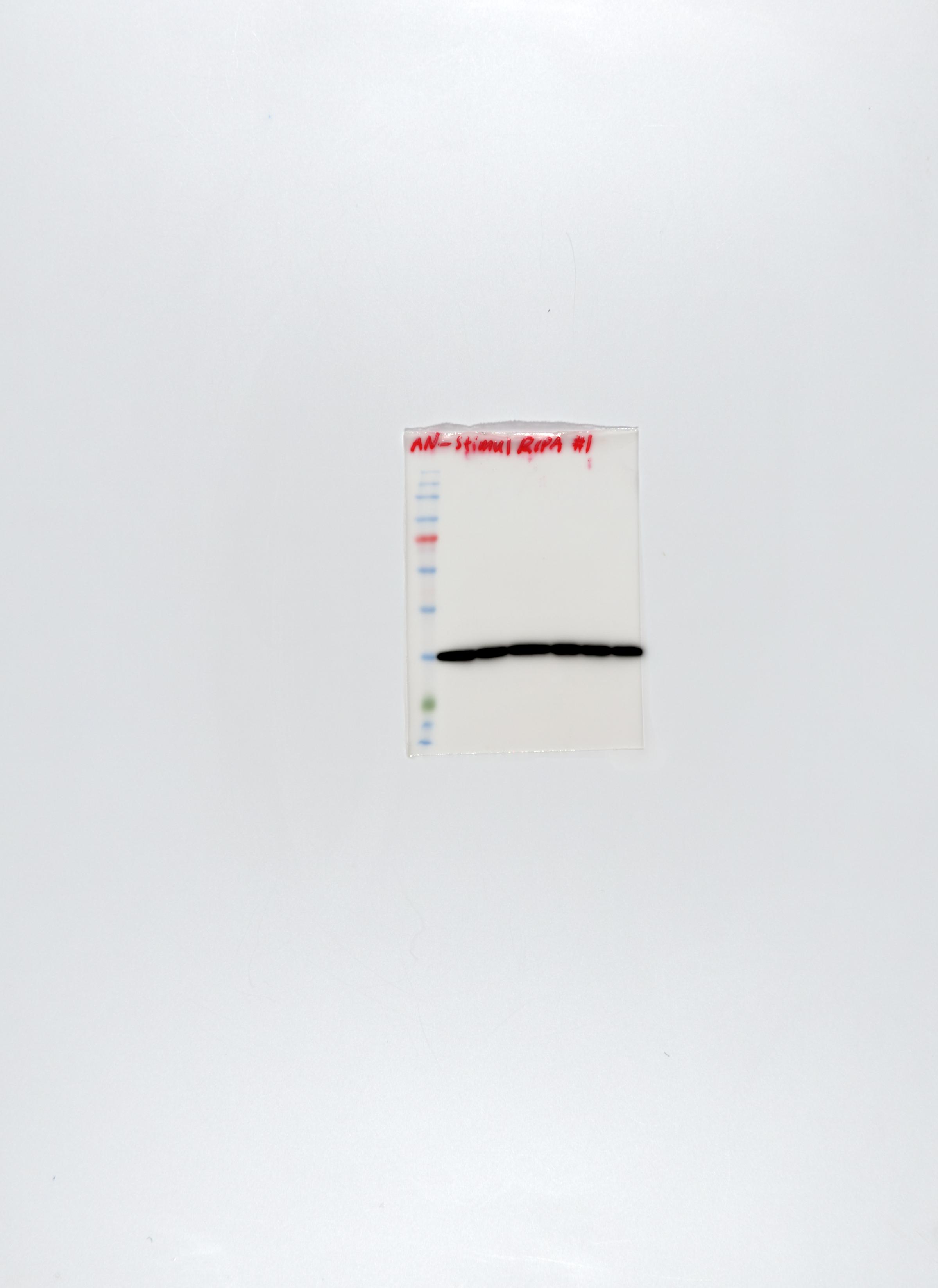

Supplement: Supplementary file 3 — Source data Fig. 1 [file 44321_2026_425_MOESM3_ESM.zip › Figure 1 Source Data/1E/1E_GAPDH.jpg]

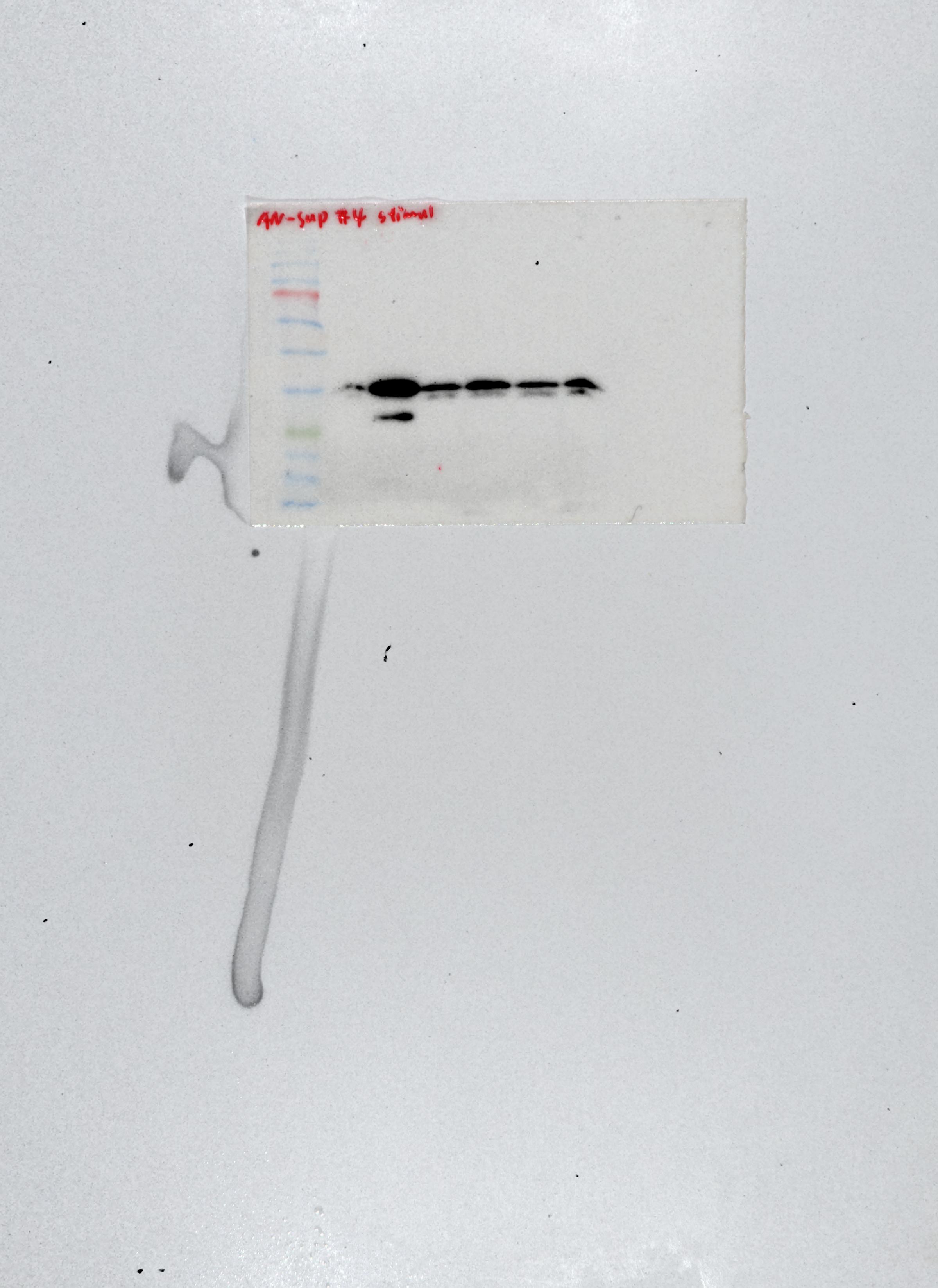

Supplement: Supplementary file 3 — Source data Fig. 1 [file 44321_2026_425_MOESM3_ESM.zip › Figure 1 Source Data/1E/1E_LDH.jpg]

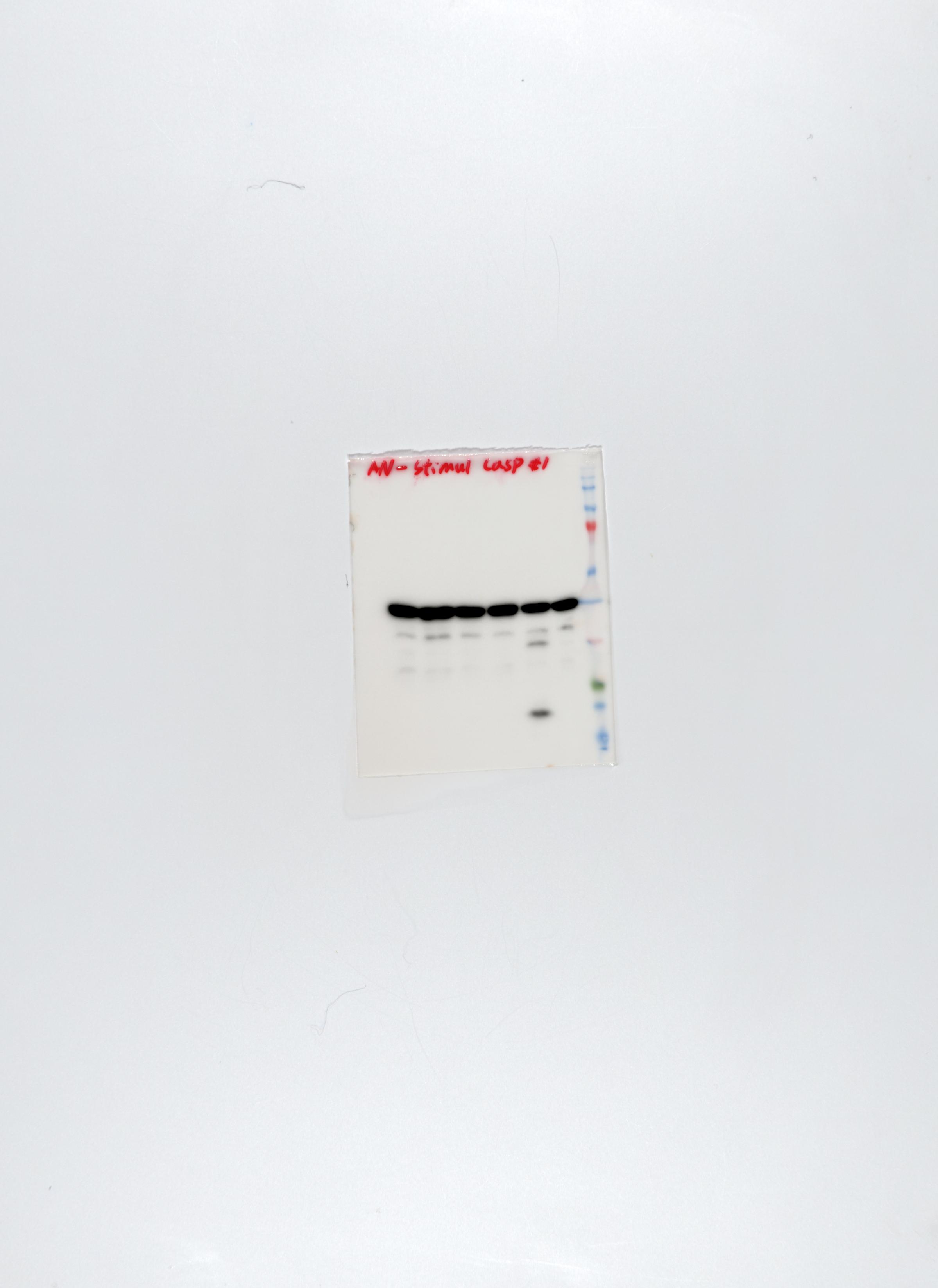

Supplement: Supplementary file 3 — Source data Fig. 1 [file 44321_2026_425_MOESM3_ESM.zip › Figure 1 Source Data/1E/1E_CASP1.jpg]

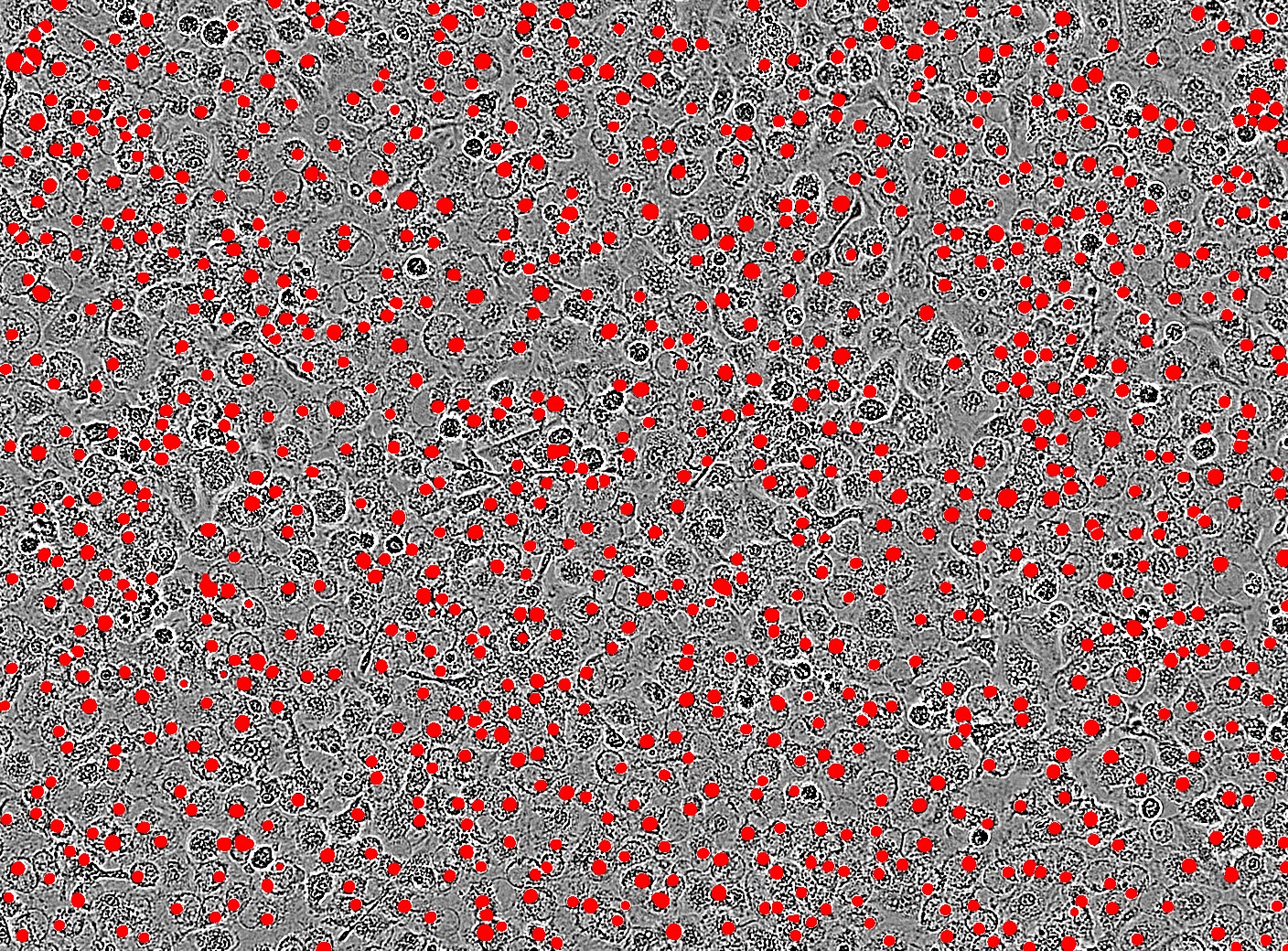

Supplement: Supplementary file 3 — Source data Fig. 1 [file 44321_2026_425_MOESM3_ESM.zip › Figure 1 Source Data/1D/1D_Vehicle_1.5h.jpg]

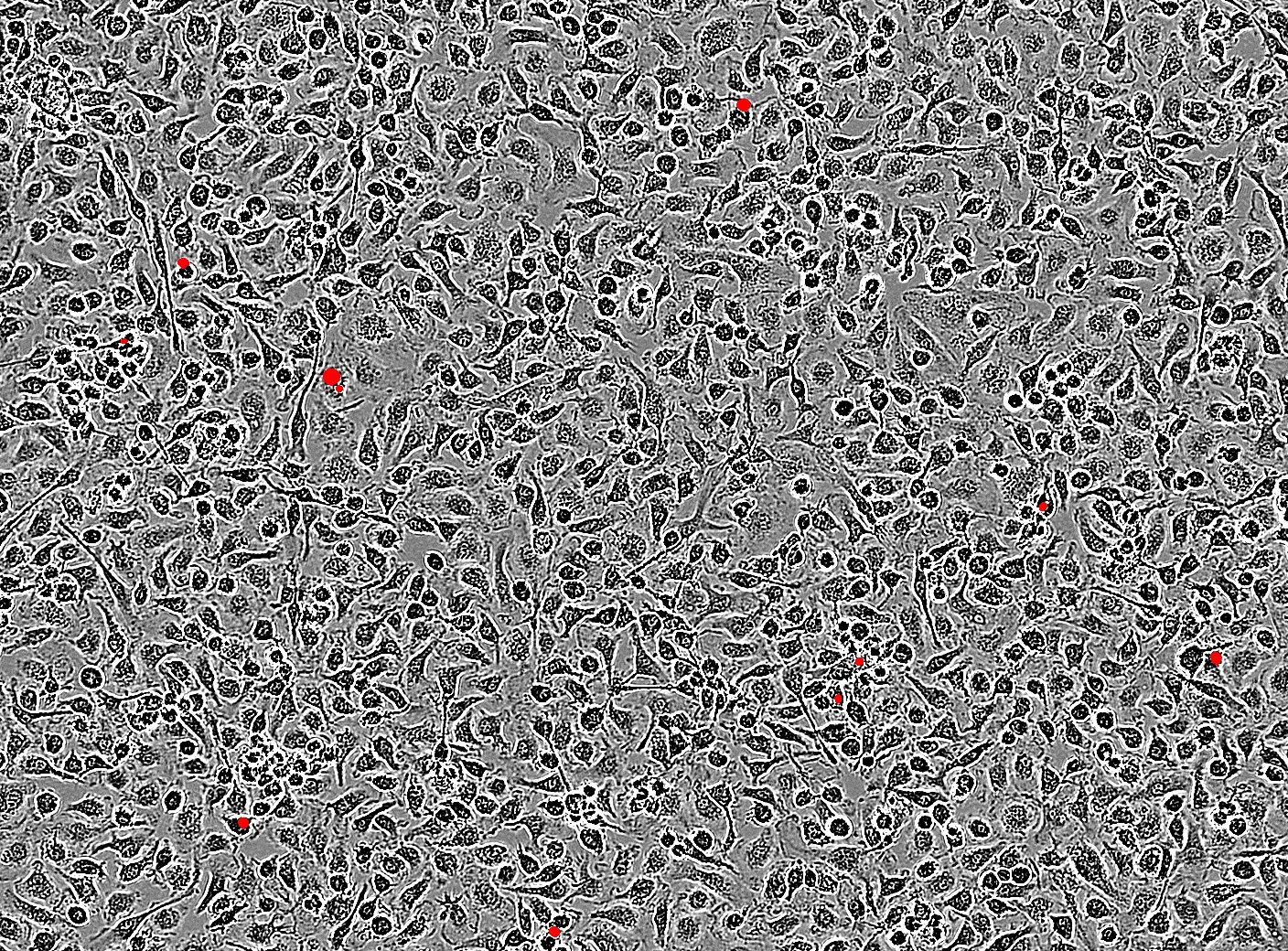

Supplement: Supplementary file 3 — Source data Fig. 1 [file 44321_2026_425_MOESM3_ESM.zip › Figure 1 Source Data/1D/1D_Media_0h.jpg]

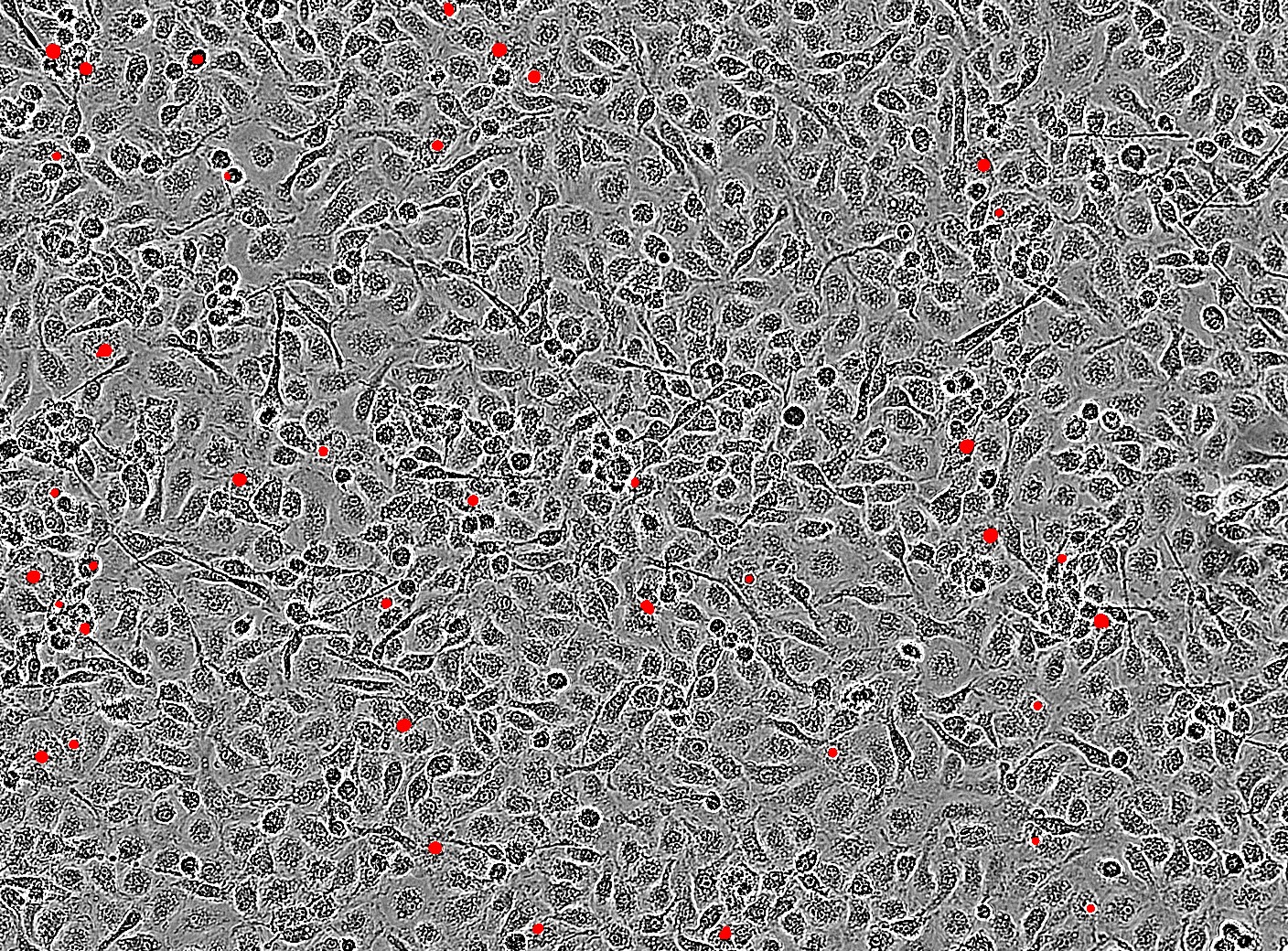

Supplement: Supplementary file 3 — Source data Fig. 1 [file 44321_2026_425_MOESM3_ESM.zip › Figure 1 Source Data/1D/1D_Vehicle_0h.jpg]

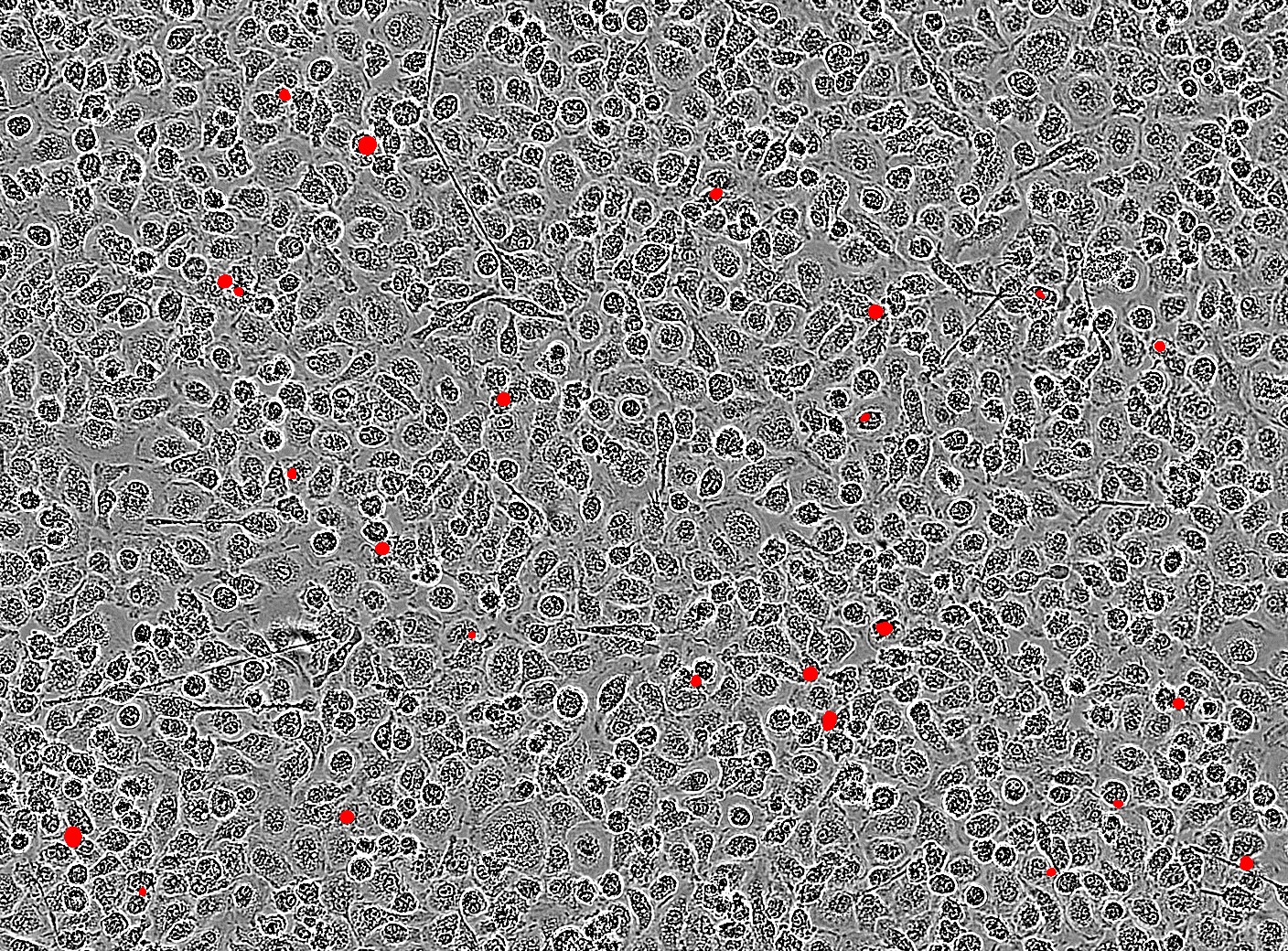

Supplement: Supplementary file 3 — Source data Fig. 1 [file 44321_2026_425_MOESM3_ESM.zip › Figure 1 Source Data/1D/1D_LOC14_1.5h.jpg]

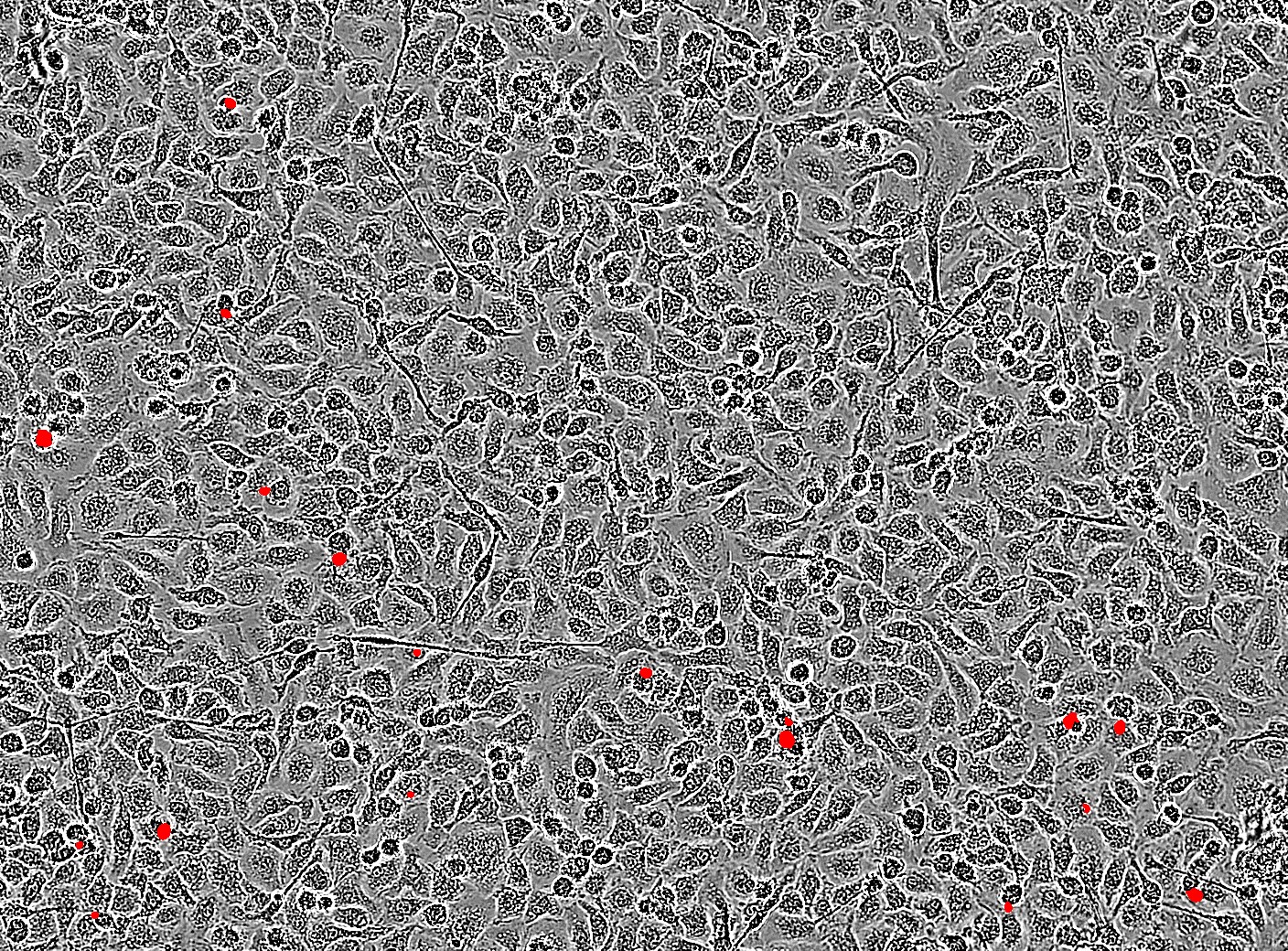

Supplement: Supplementary file 3 — Source data Fig. 1 [file 44321_2026_425_MOESM3_ESM.zip › Figure 1 Source Data/1D/1D_LOC14_0h.jpg]

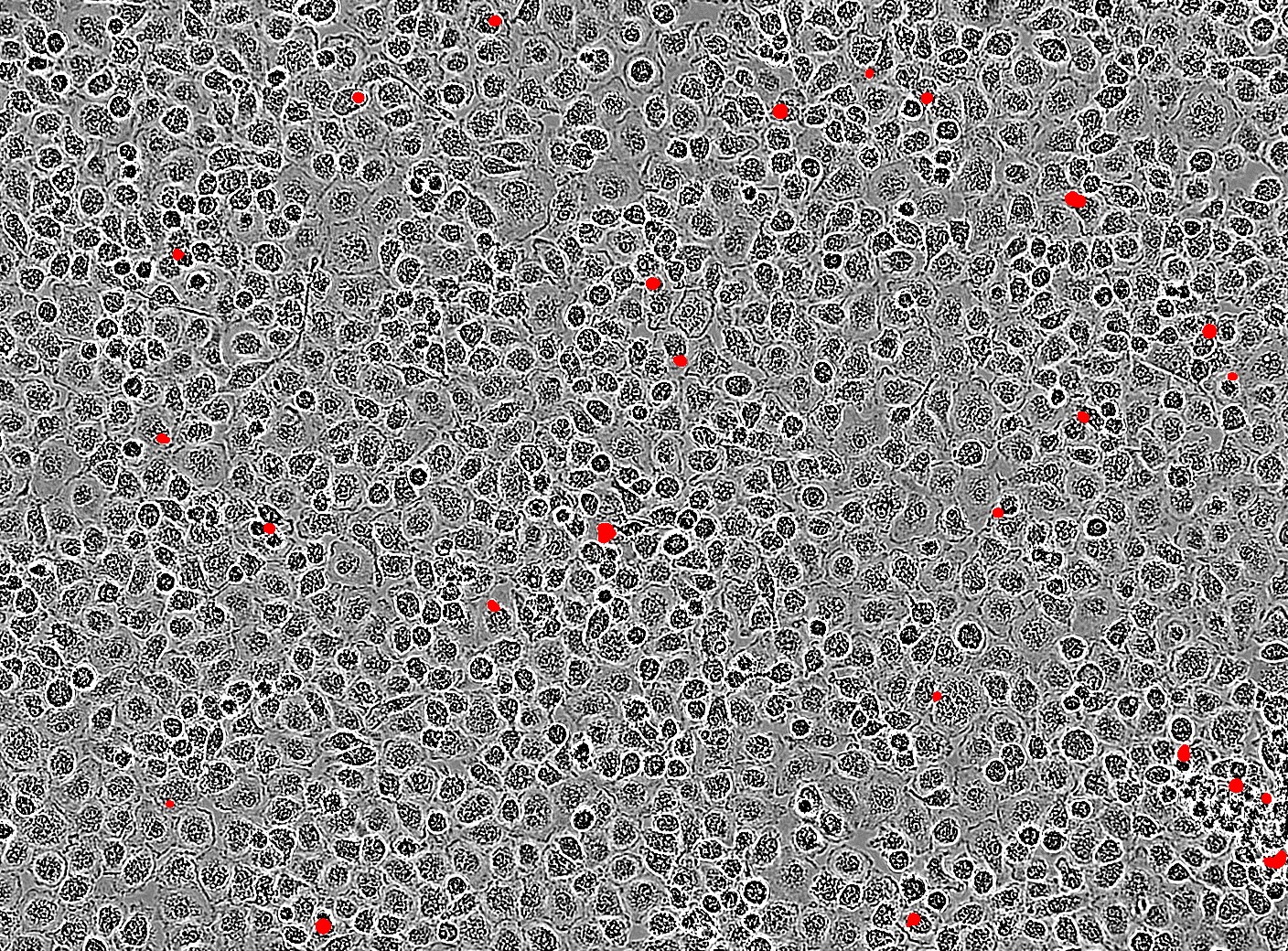

Supplement: Supplementary file 3 — Source data Fig. 1 [file 44321_2026_425_MOESM3_ESM.zip › Figure 1 Source Data/1D/1D_CRID3_1.5h.jpg]

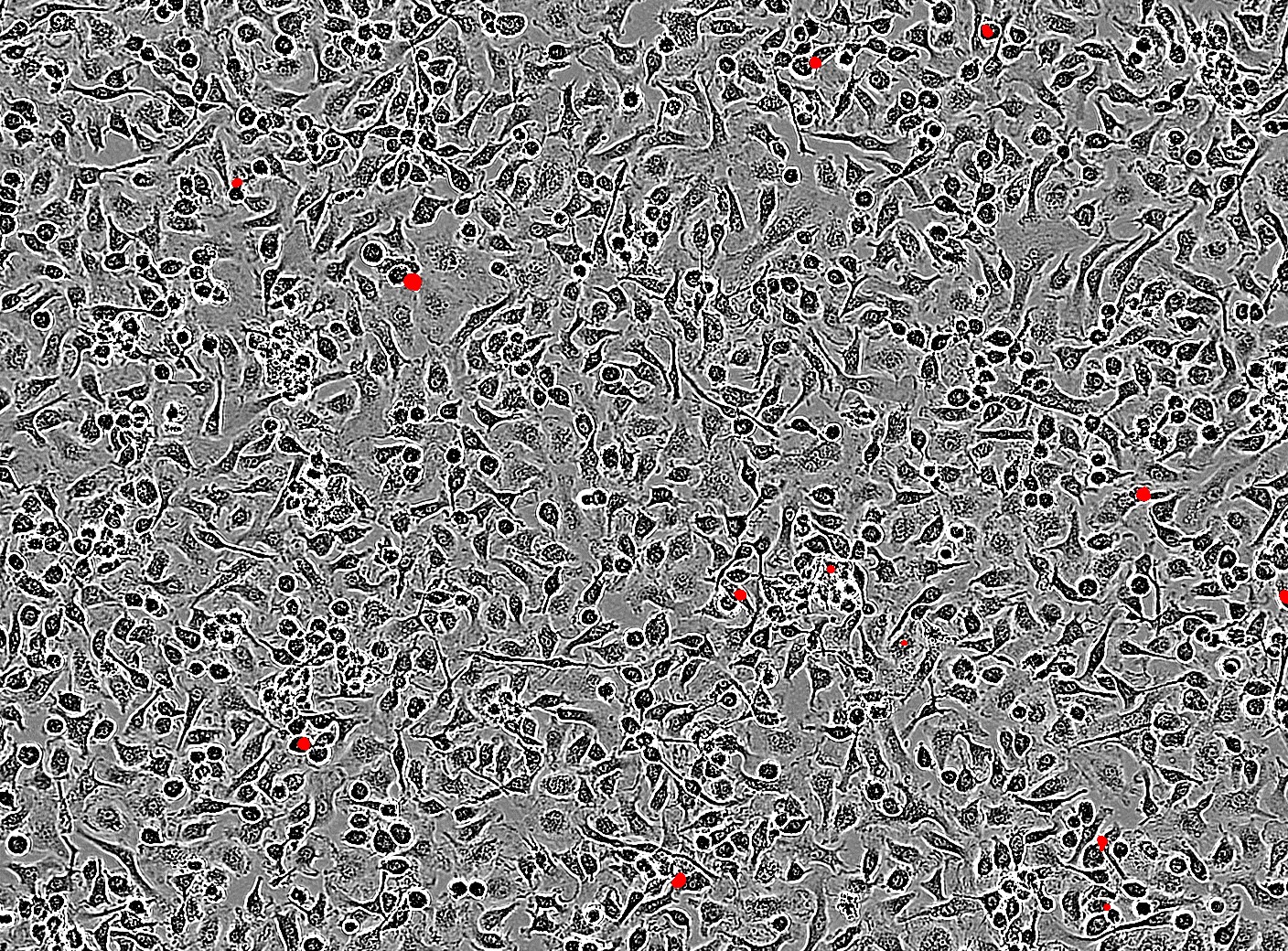

Supplement: Supplementary file 3 — Source data Fig. 1 [file 44321_2026_425_MOESM3_ESM.zip › Figure 1 Source Data/1D/1D_Media_1.5h.jpg]

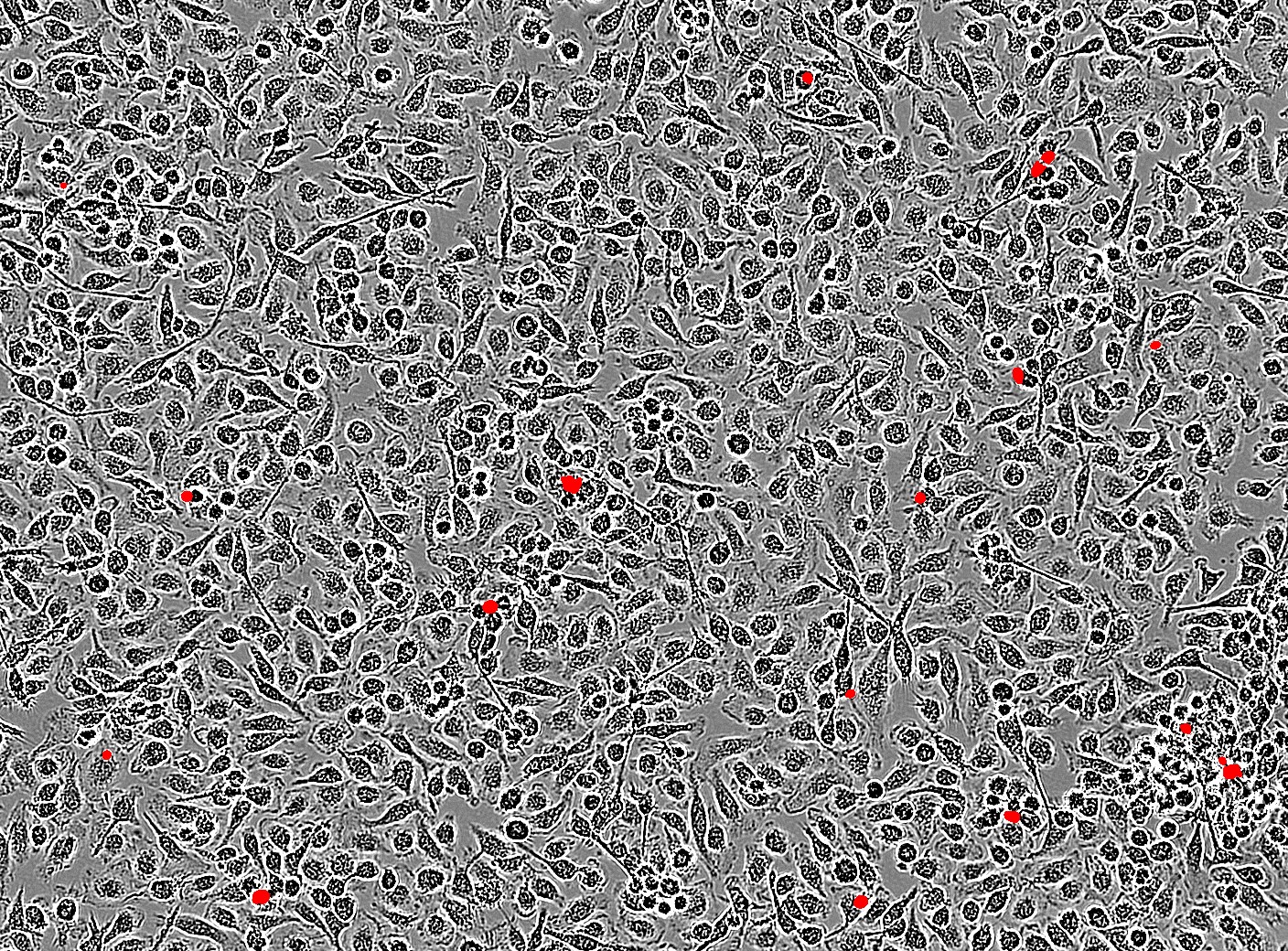

Supplement: Supplementary file 3 — Source data Fig. 1 [file 44321_2026_425_MOESM3_ESM.zip › Figure 1 Source Data/1D/1D_CRID3_0h.jpg]

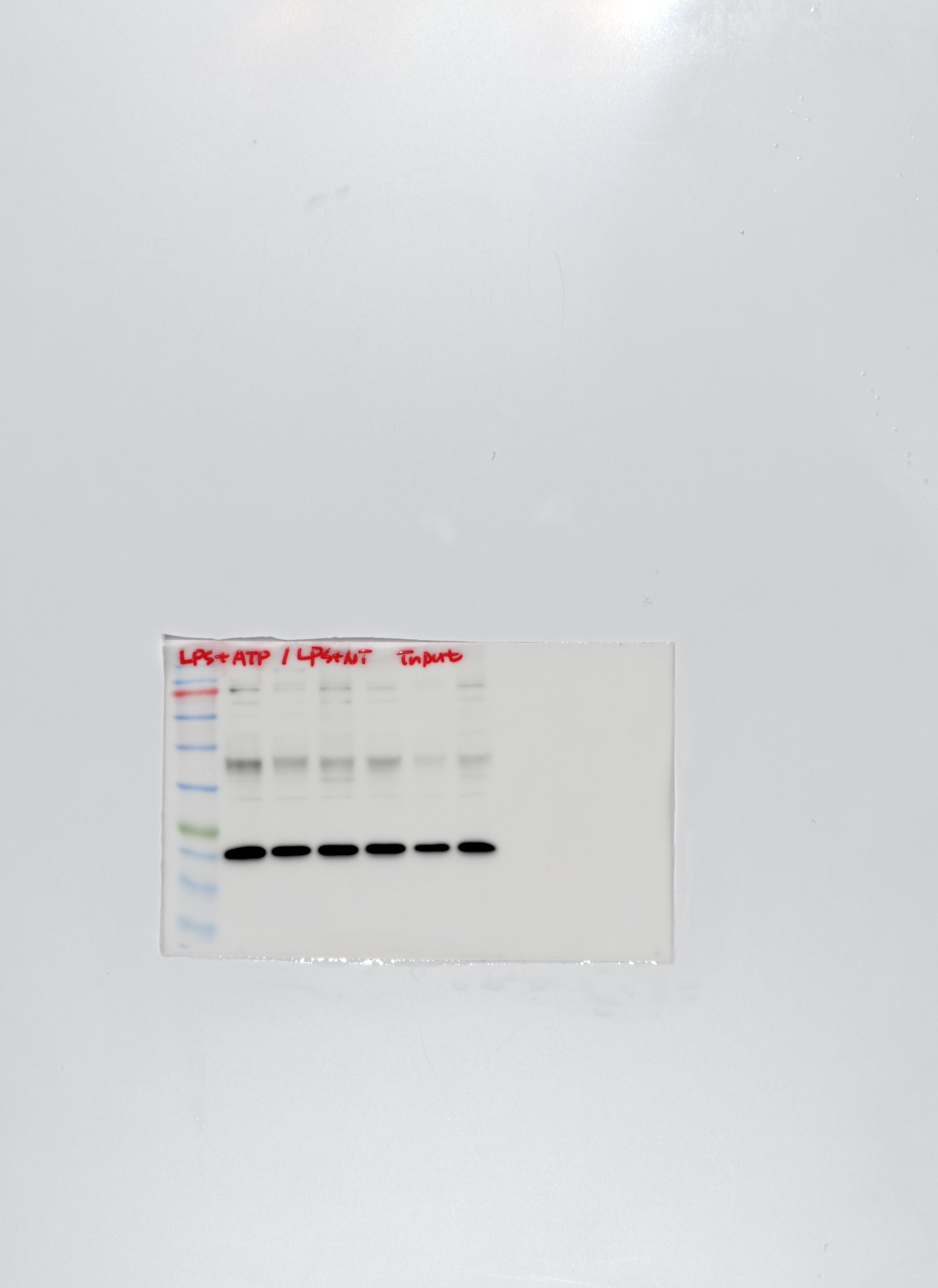

Supplement: Supplementary file 5 — Source data Fig. 3 [file 44321_2026_425_MOESM5_ESM.zip › Figure 3 Source Data/3B/3B_ASC.jpg]

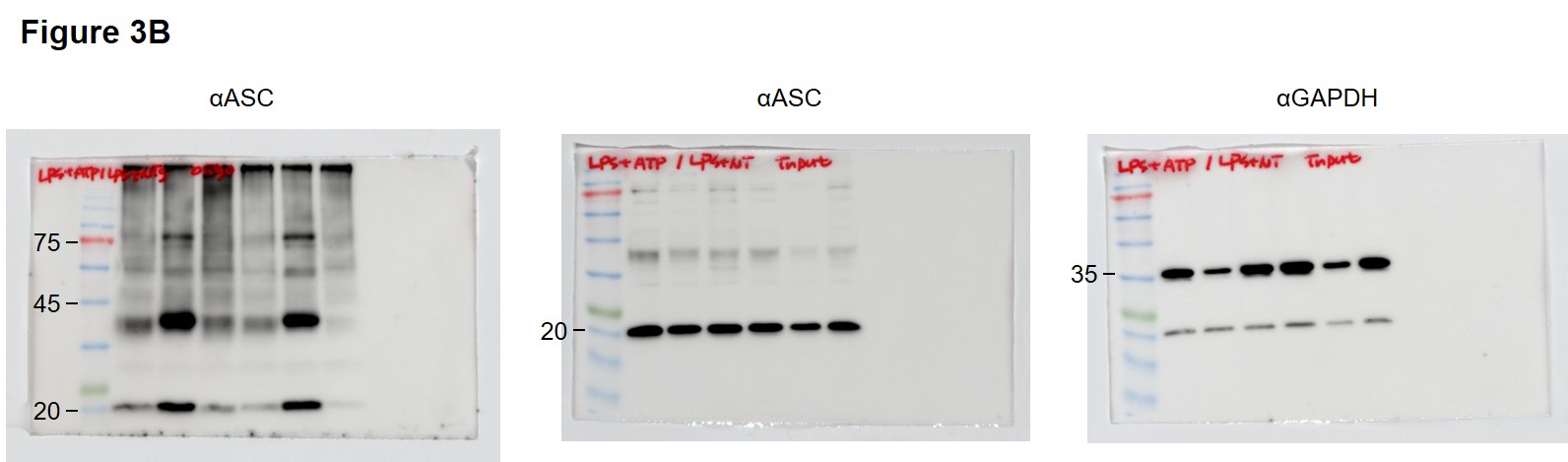

Supplement: Supplementary file 5 — Source data Fig. 3 [file 44321_2026_425_MOESM5_ESM.zip › Figure 3 Source Data/3B/3B.jpg]

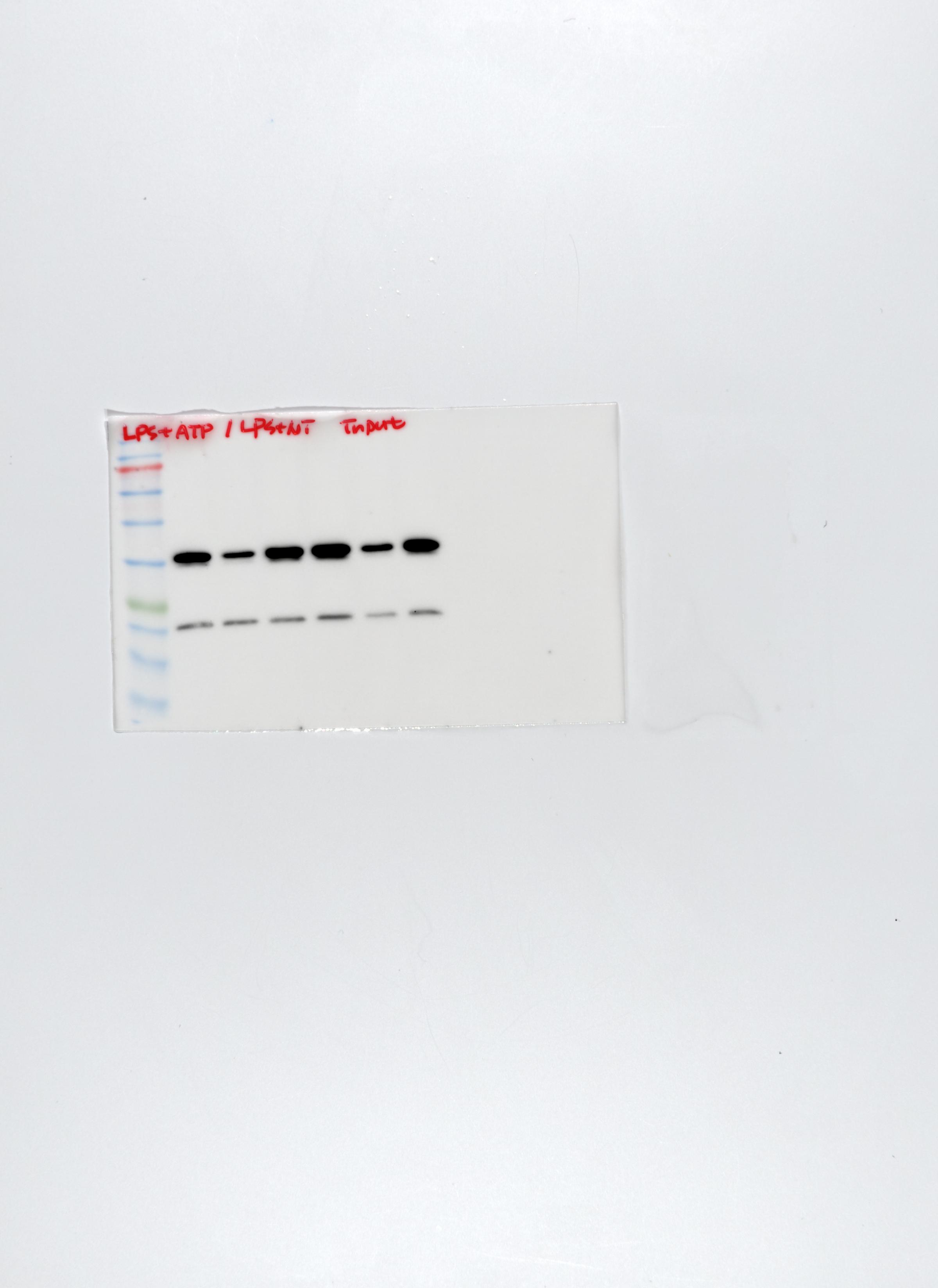

Supplement: Supplementary file 5 — Source data Fig. 3 [file 44321_2026_425_MOESM5_ESM.zip › Figure 3 Source Data/3B/3B_GAPDH.jpg]

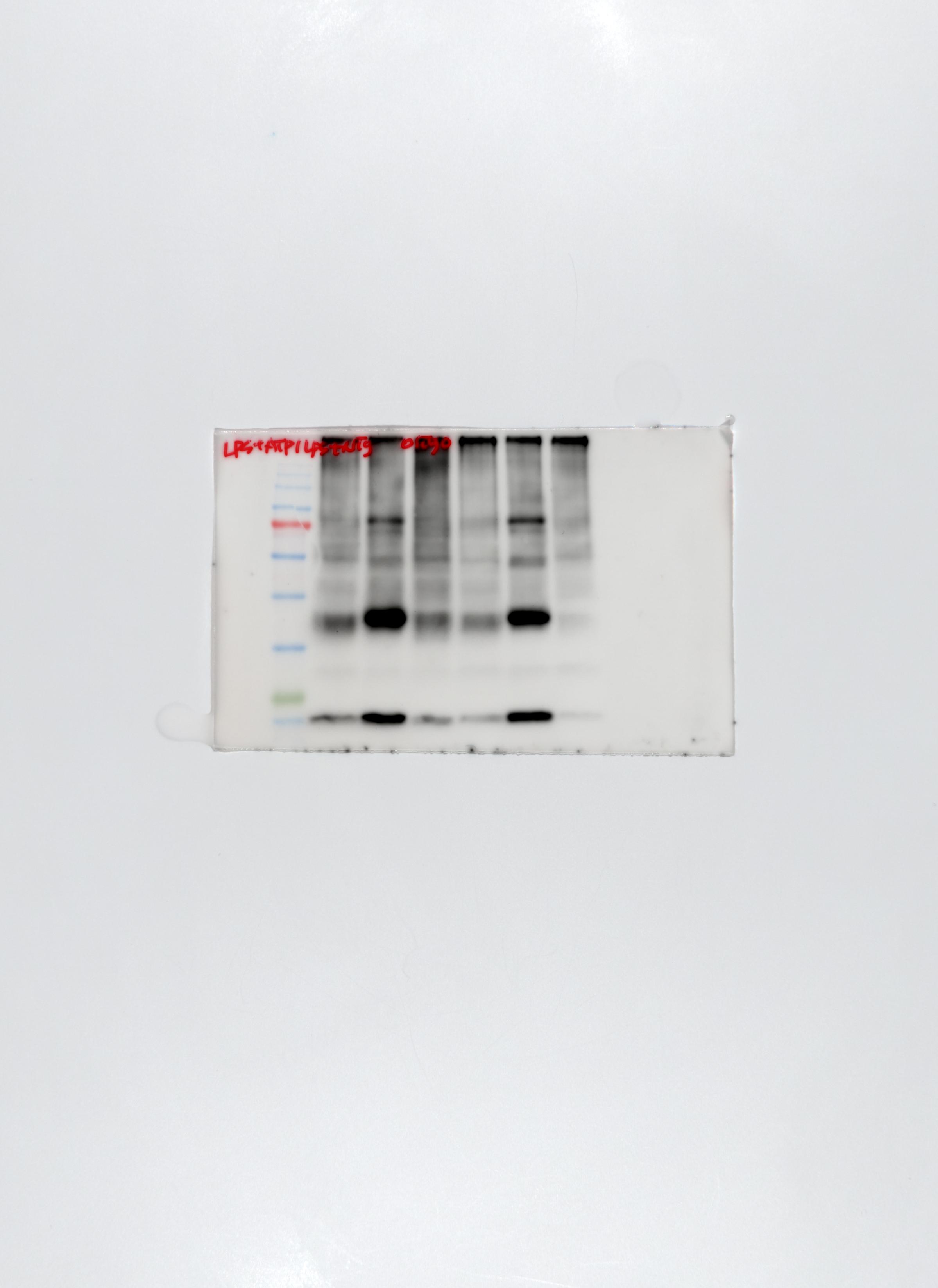

Supplement: Supplementary file 5 — Source data Fig. 3 [file 44321_2026_425_MOESM5_ESM.zip › Figure 3 Source Data/3B/3B_ASC oligomers.jpg]

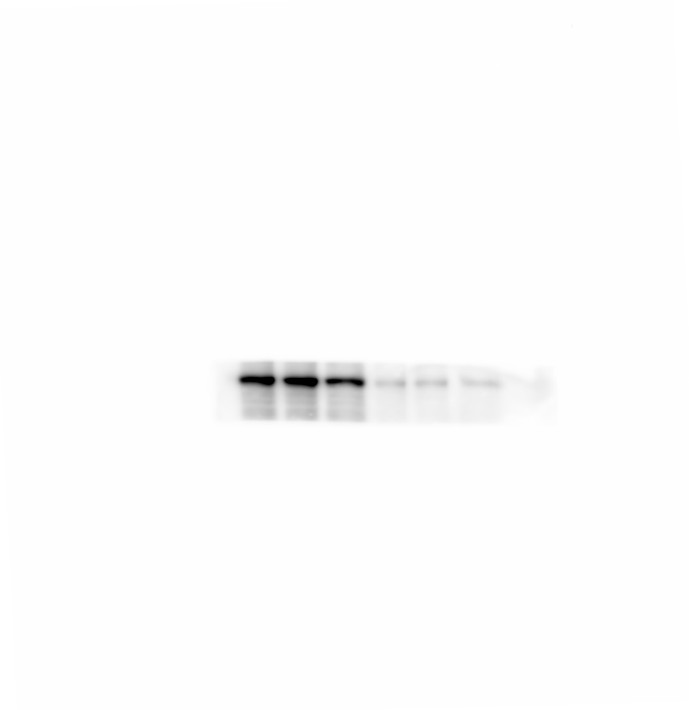

Supplement: Supplementary file 5 — Source data Fig. 3 [file 44321_2026_425_MOESM5_ESM.zip › Figure 3 Source Data/3D/3D_ASC.tiff]

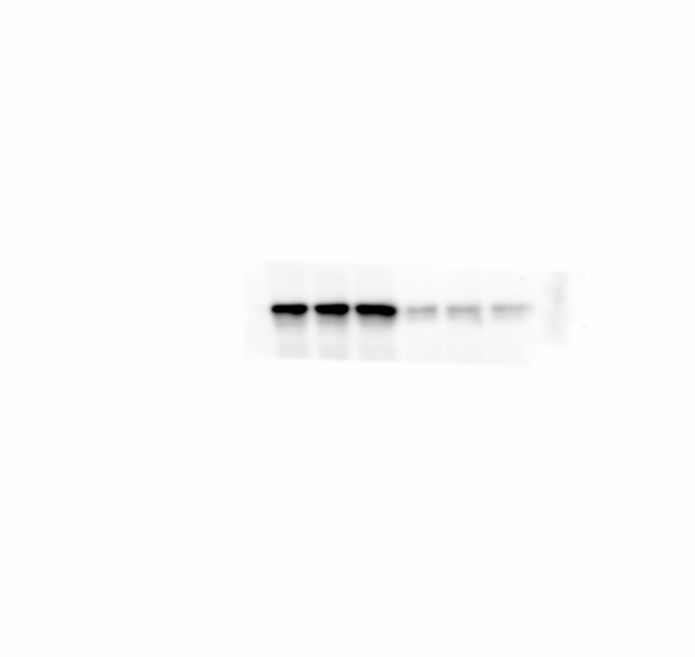

Supplement: Supplementary file 5 — Source data Fig. 3 [file 44321_2026_425_MOESM5_ESM.zip › Figure 3 Source Data/3D/3D_CASP1.tiff]

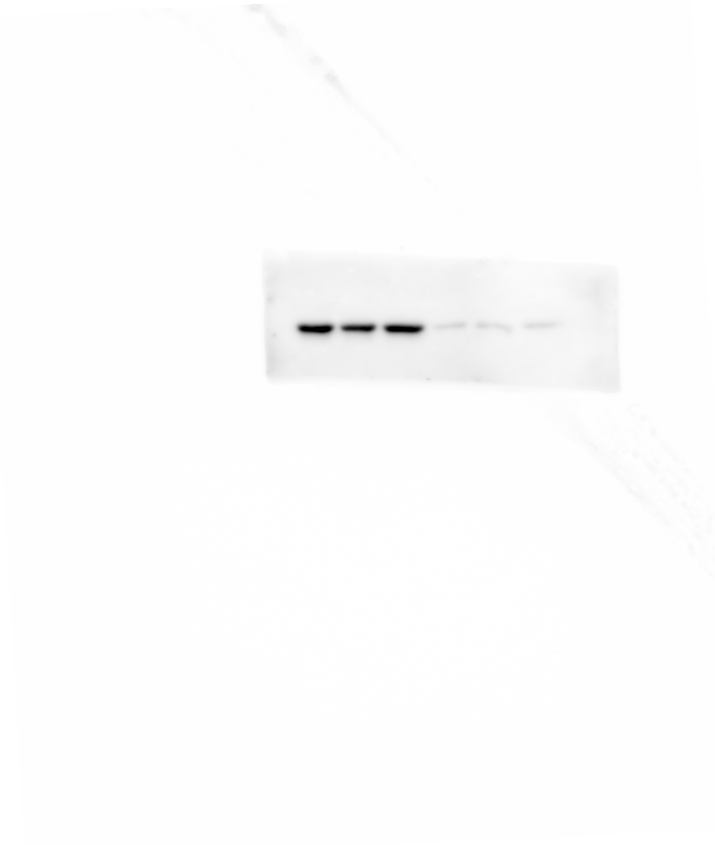

Supplement: Supplementary file 5 — Source data Fig. 3 [file 44321_2026_425_MOESM5_ESM.zip › Figure 3 Source Data/3D/3D_b-actin.tiff]

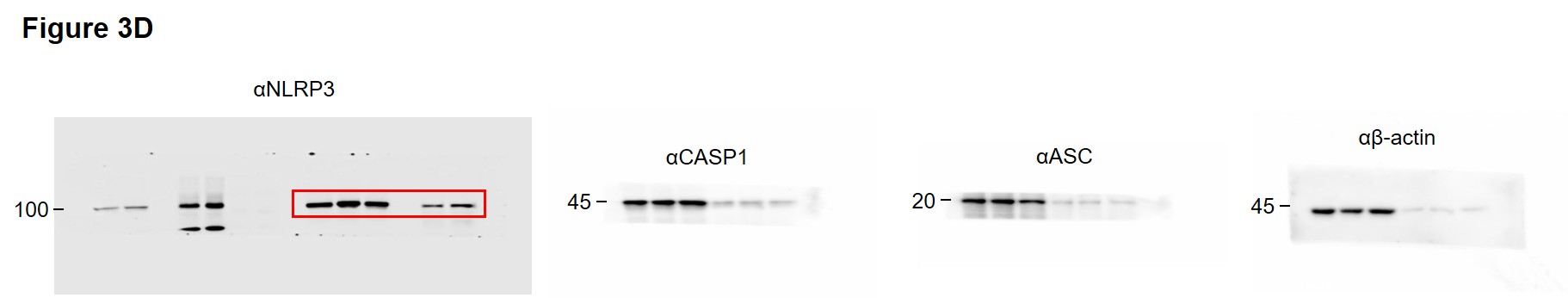

Supplement: Supplementary file 5 — Source data Fig. 3 [file 44321_2026_425_MOESM5_ESM.zip › Figure 3 Source Data/3D/3D.jpg]

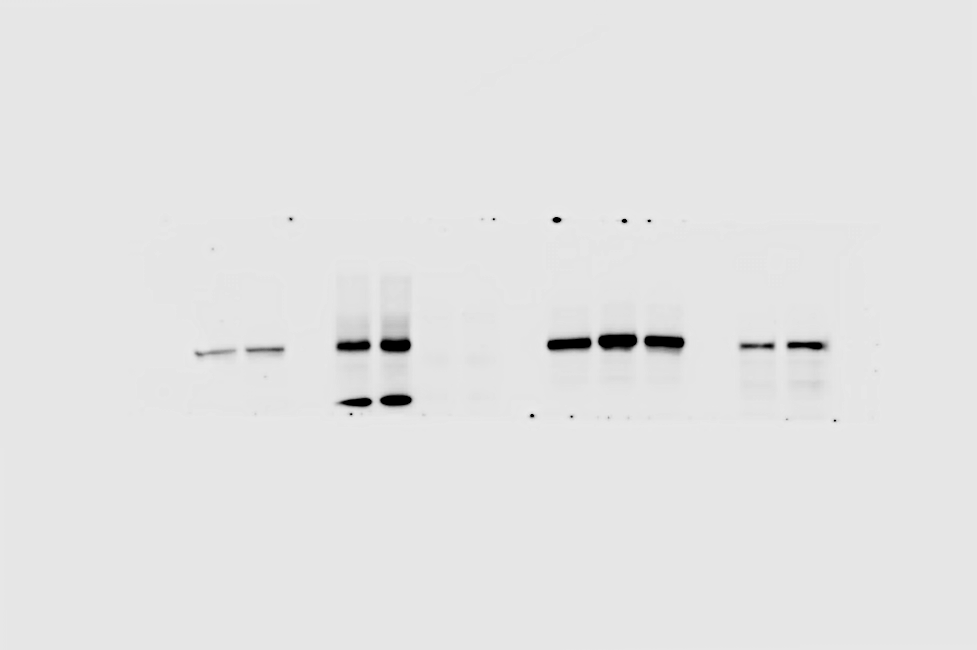

Supplement: Supplementary file 5 — Source data Fig. 3 [file 44321_2026_425_MOESM5_ESM.zip › Figure 3 Source Data/3D/3D_NLRP3.png]

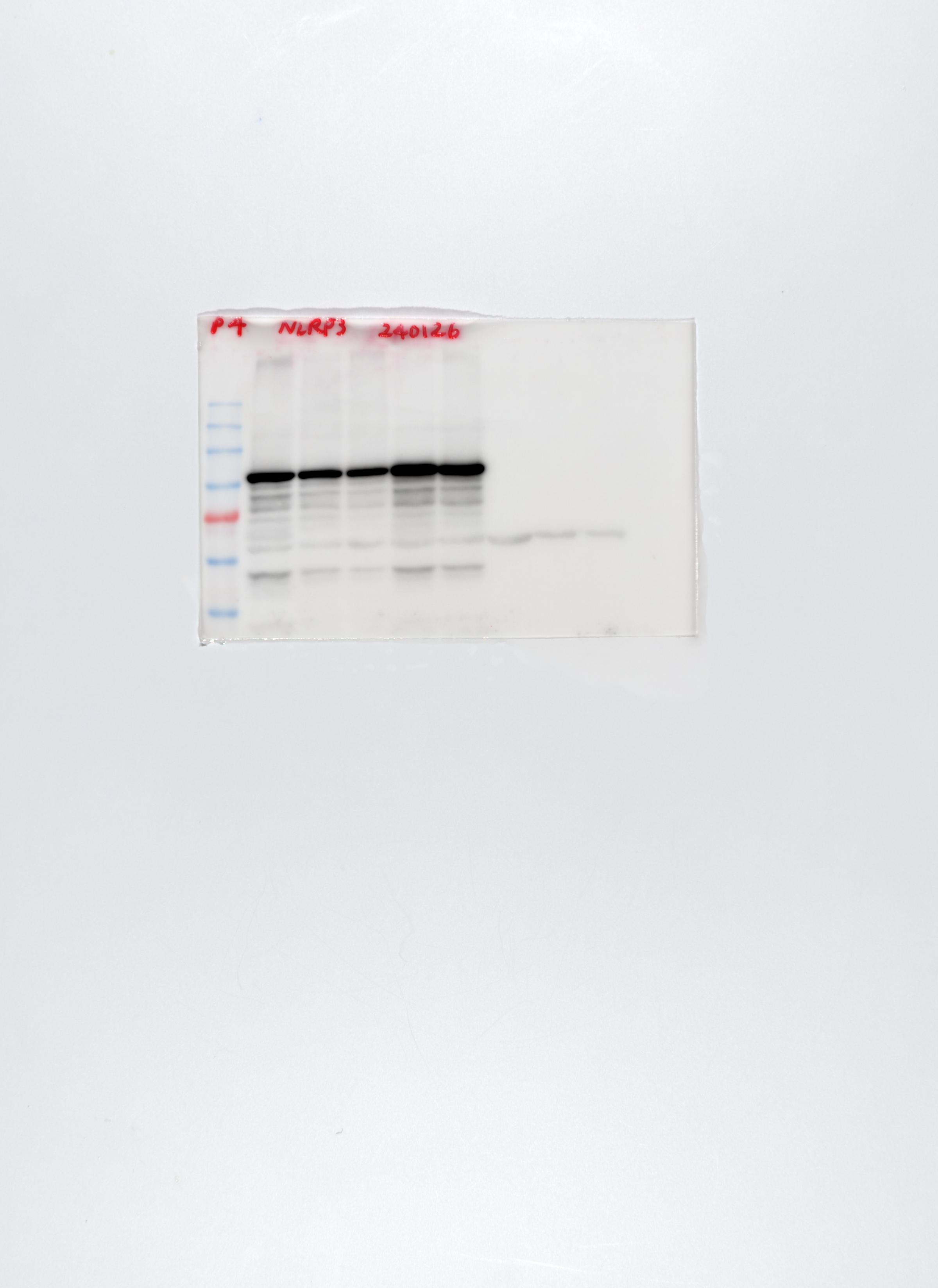

Supplement: Supplementary file 5 — Source data Fig. 3 [file 44321_2026_425_MOESM5_ESM.zip › Figure 3 Source Data/3A/3A_NLRP3.jpg]

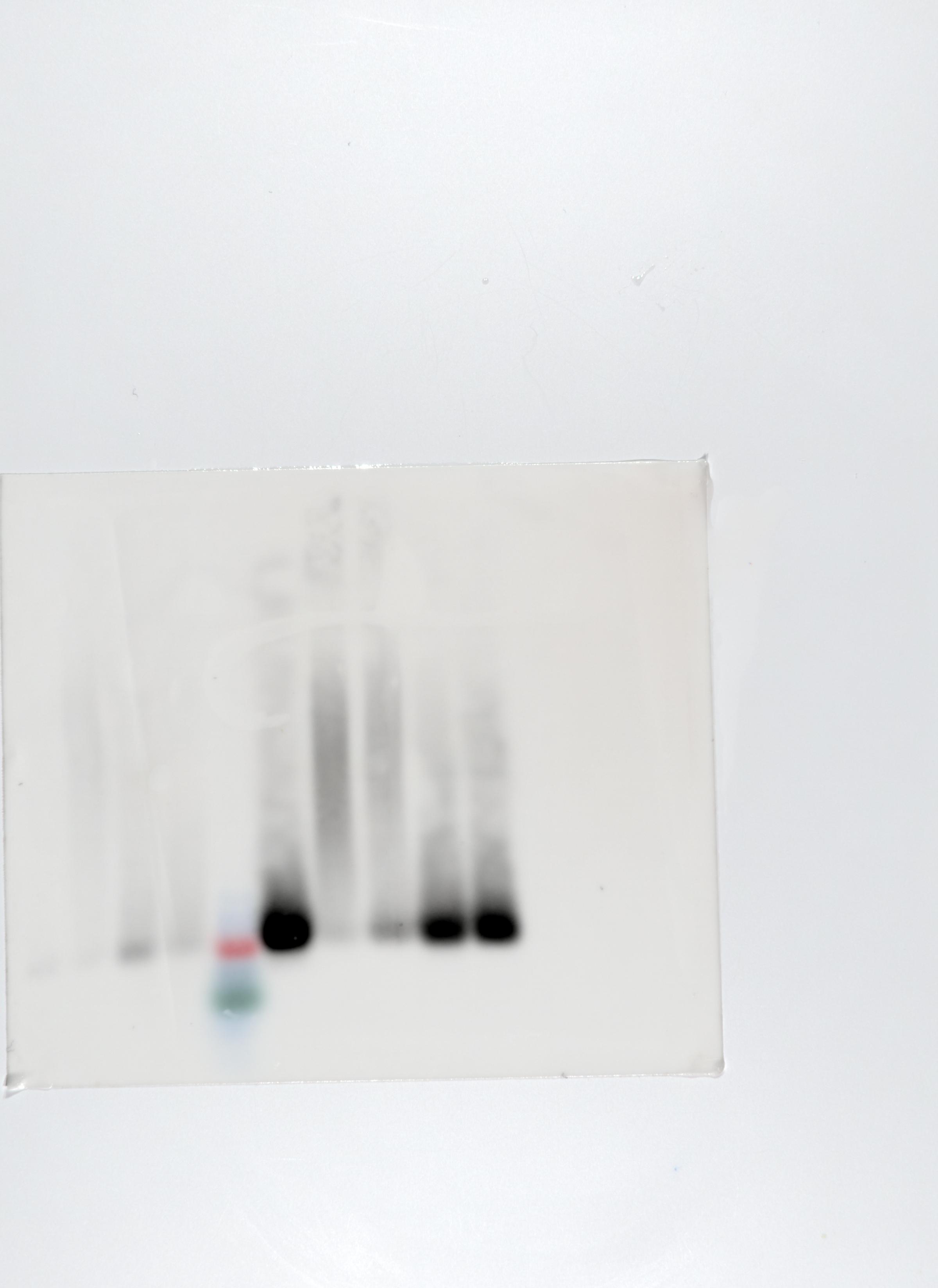

Supplement: Supplementary file 5 — Source data Fig. 3 [file 44321_2026_425_MOESM5_ESM.zip › Figure 3 Source Data/3A/3A_NLRP3 oligomers.jpg]

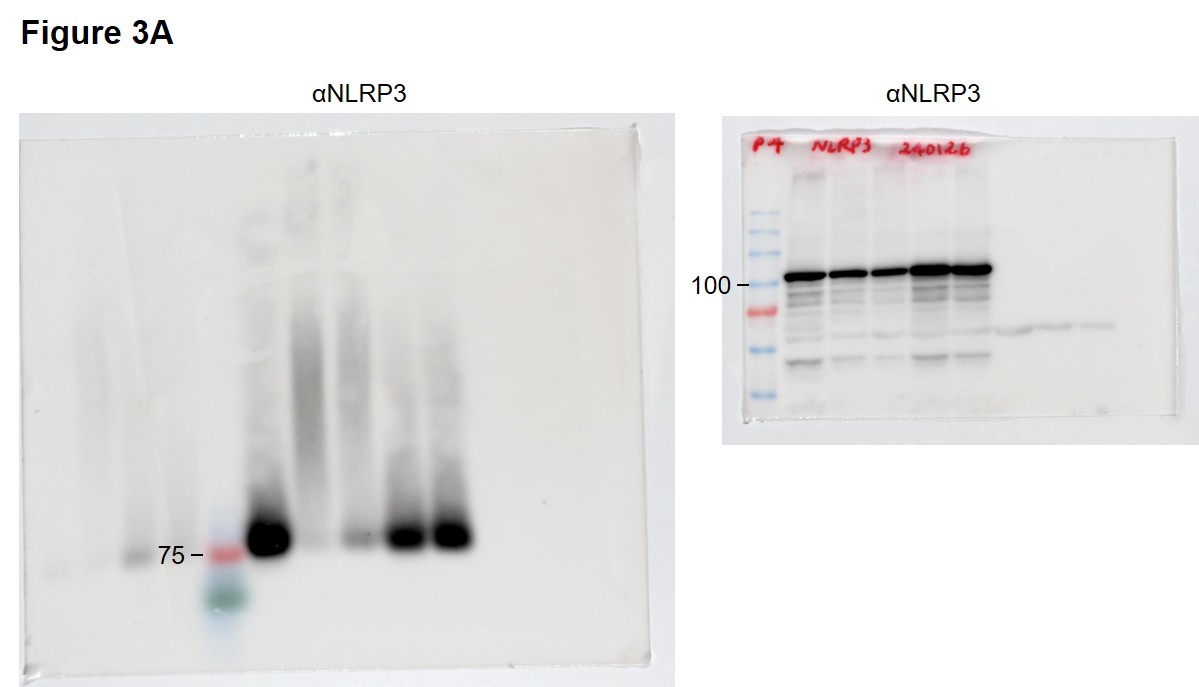

Supplement: Supplementary file 5 — Source data Fig. 3 [file 44321_2026_425_MOESM5_ESM.zip › Figure 3 Source Data/3A/3A.jpg]

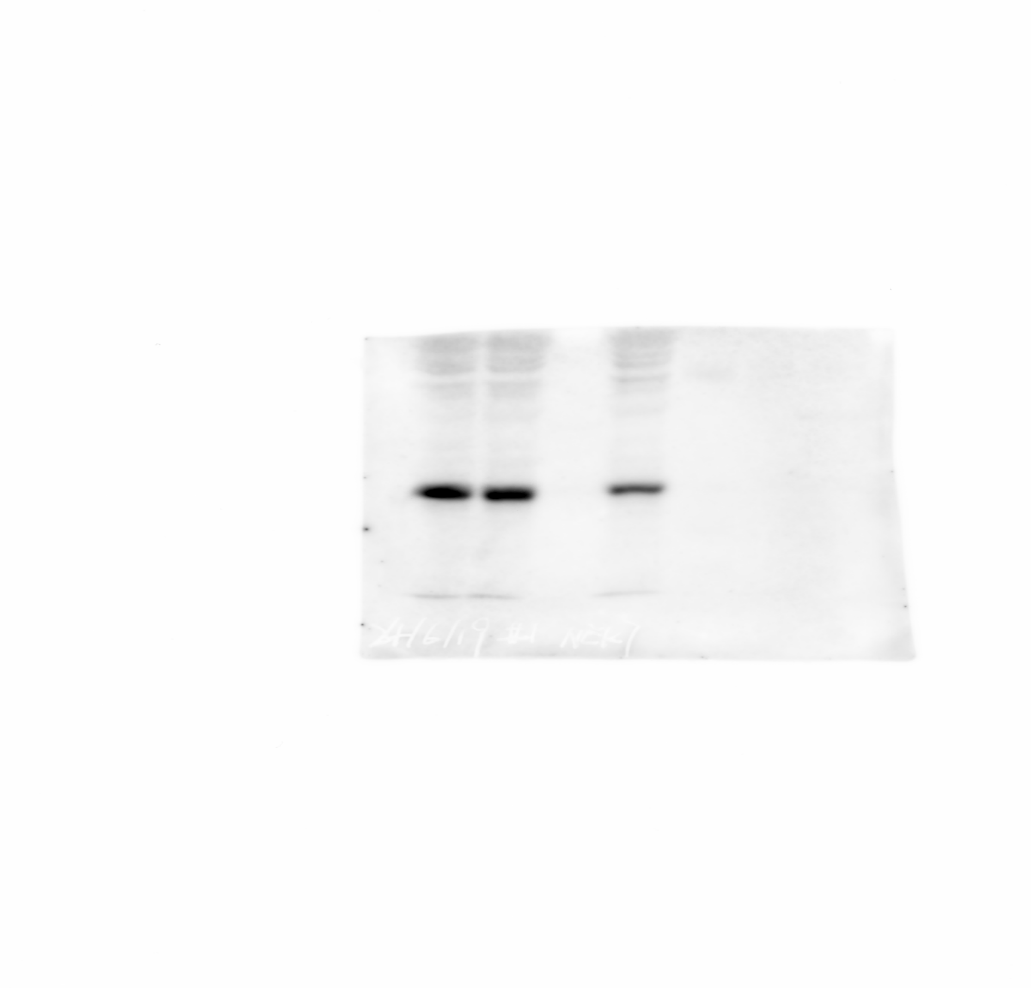

Supplement: Supplementary file 5 — Source data Fig. 3 [file 44321_2026_425_MOESM5_ESM.zip › Figure 3 Source Data/3F/3F_NEK7.tiff]

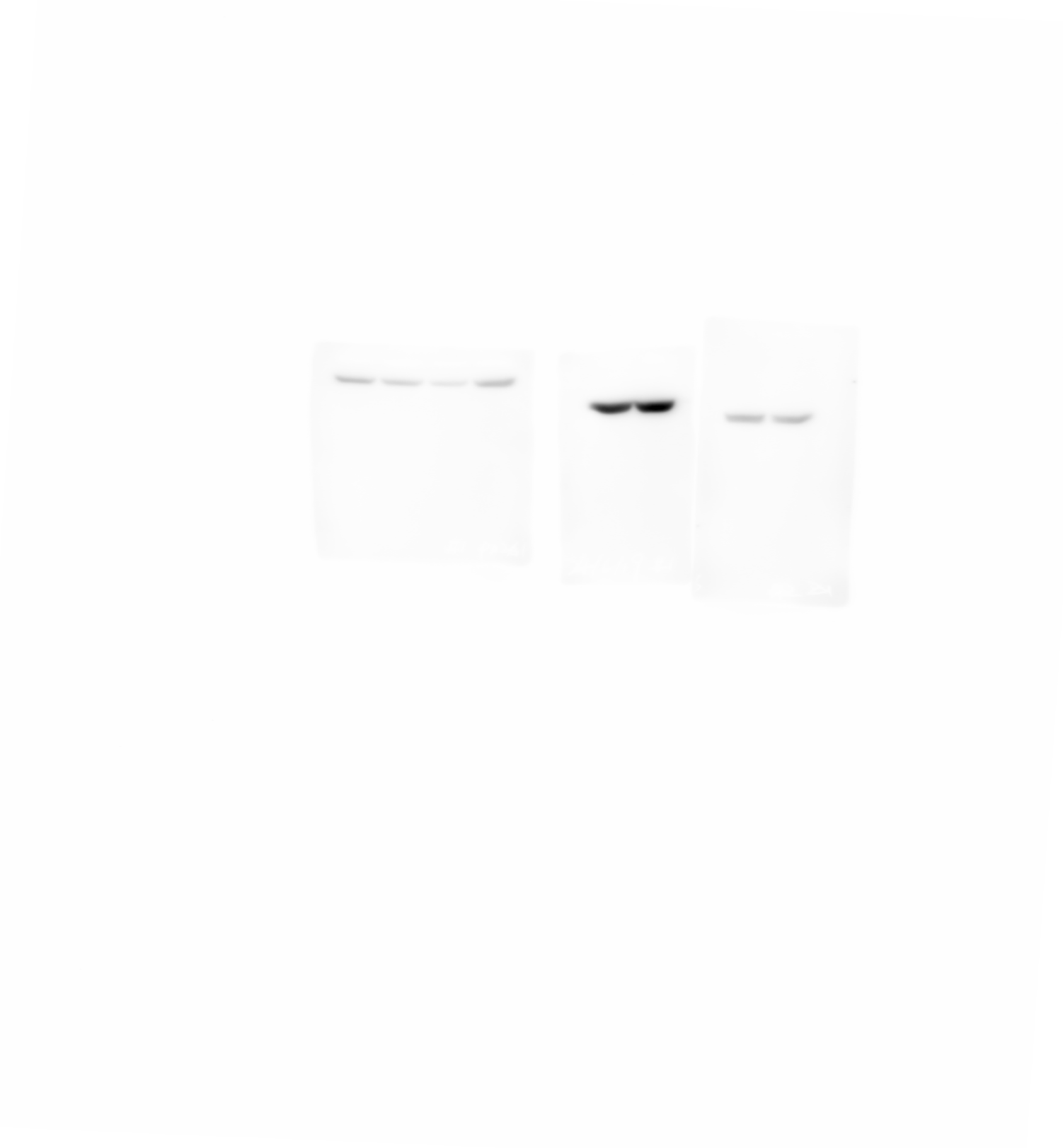

Supplement: Supplementary file 5 — Source data Fig. 3 [file 44321_2026_425_MOESM5_ESM.zip › Figure 3 Source Data/3F/3F_b-actin.tiff]

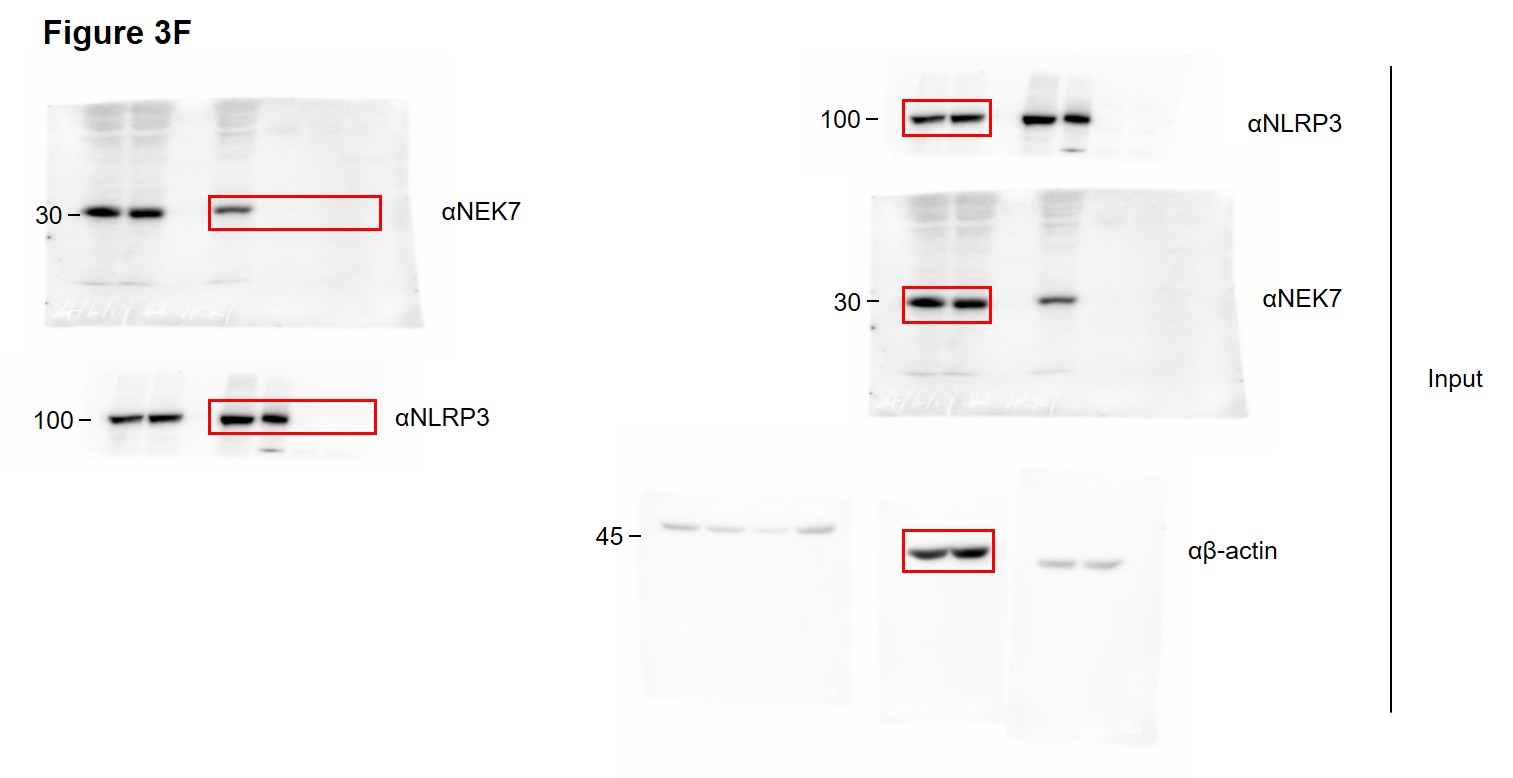

Supplement: Supplementary file 5 — Source data Fig. 3 [file 44321_2026_425_MOESM5_ESM.zip › Figure 3 Source Data/3F/3F.jpg]

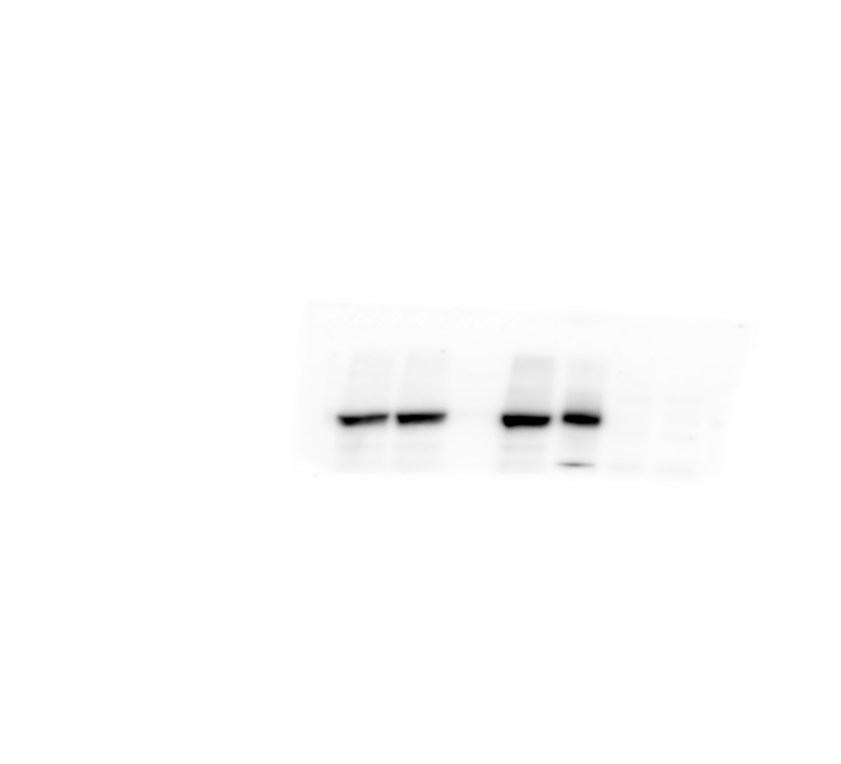

Supplement: Supplementary file 5 — Source data Fig. 3 [file 44321_2026_425_MOESM5_ESM.zip › Figure 3 Source Data/3F/3F_NLRP3.tiff]

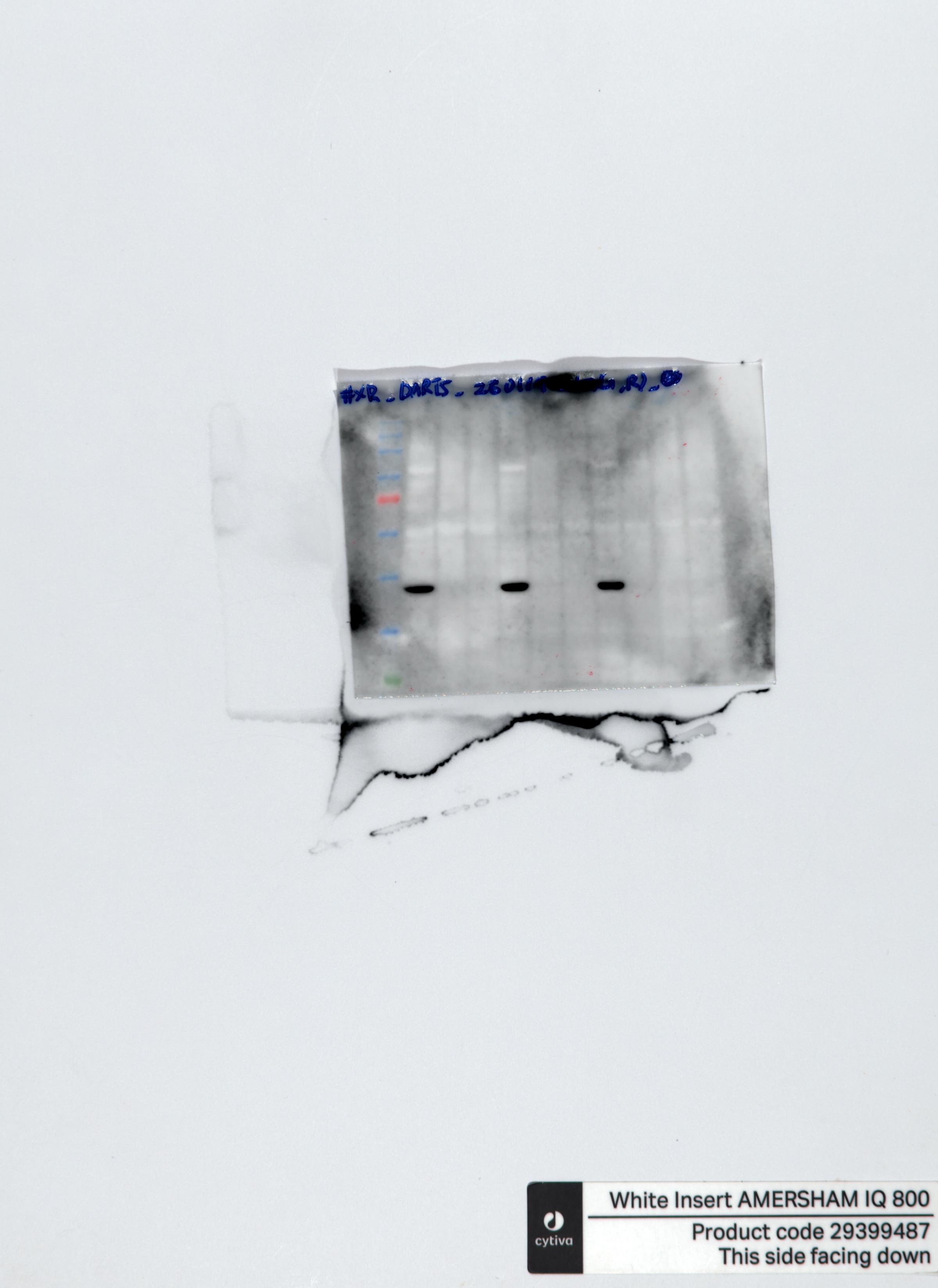

Supplement: Supplementary file 6 — Source data Fig. 4 [file 44321_2026_425_MOESM6_ESM.zip › Figure 4 Source Data/4E/4E_b-actin.jpg]

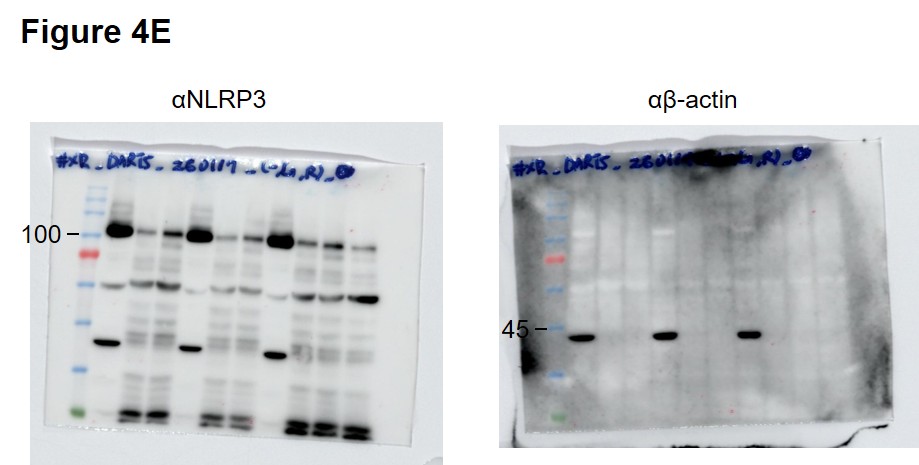

Supplement: Supplementary file 6 — Source data Fig. 4 [file 44321_2026_425_MOESM6_ESM.zip › Figure 4 Source Data/4E/4E.jpg]

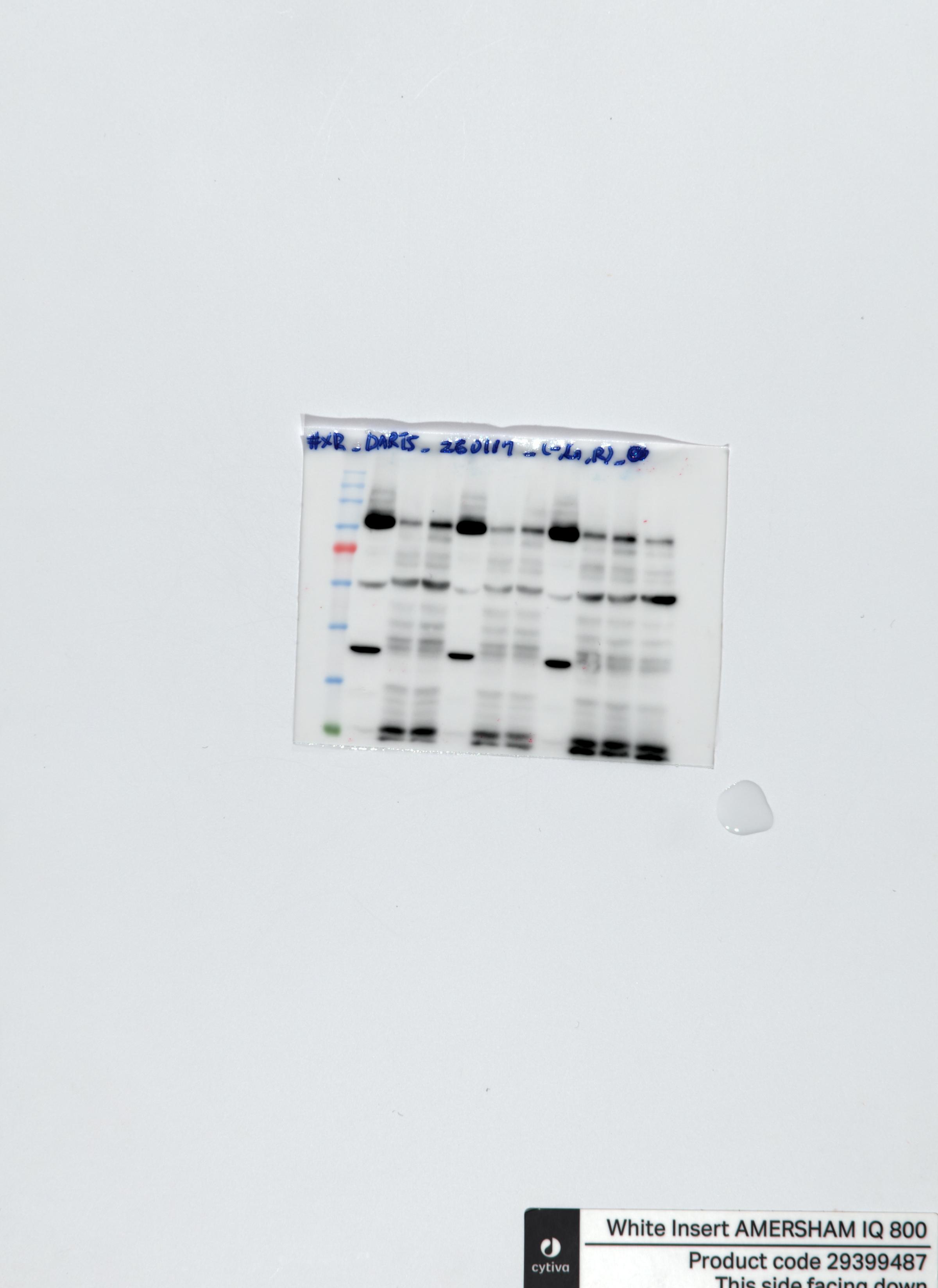

Supplement: Supplementary file 6 — Source data Fig. 4 [file 44321_2026_425_MOESM6_ESM.zip › Figure 4 Source Data/4E/4E_NLRP3.jpg]

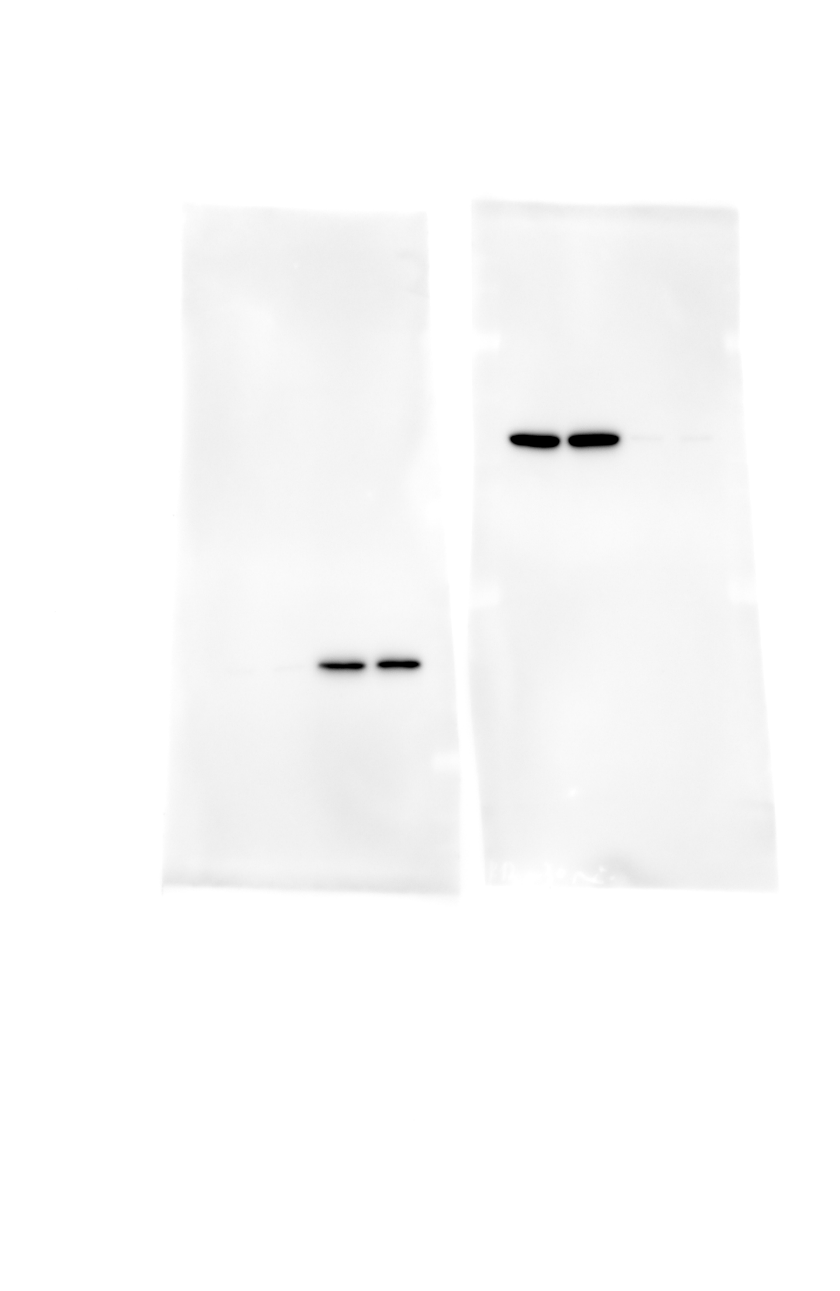

Supplement: Supplementary file 6 — Source data Fig. 4 [file 44321_2026_425_MOESM6_ESM.zip › Figure 4 Source Data/4B/4B_b-actin.tiff]

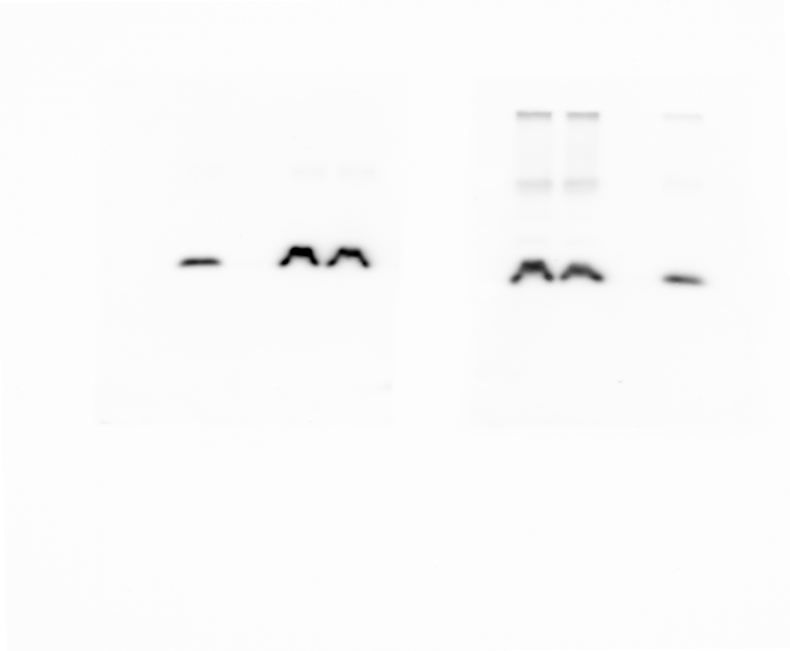

Supplement: Supplementary file 6 — Source data Fig. 4 [file 44321_2026_425_MOESM6_ESM.zip › Figure 4 Source Data/4B/4B_flag.tiff]

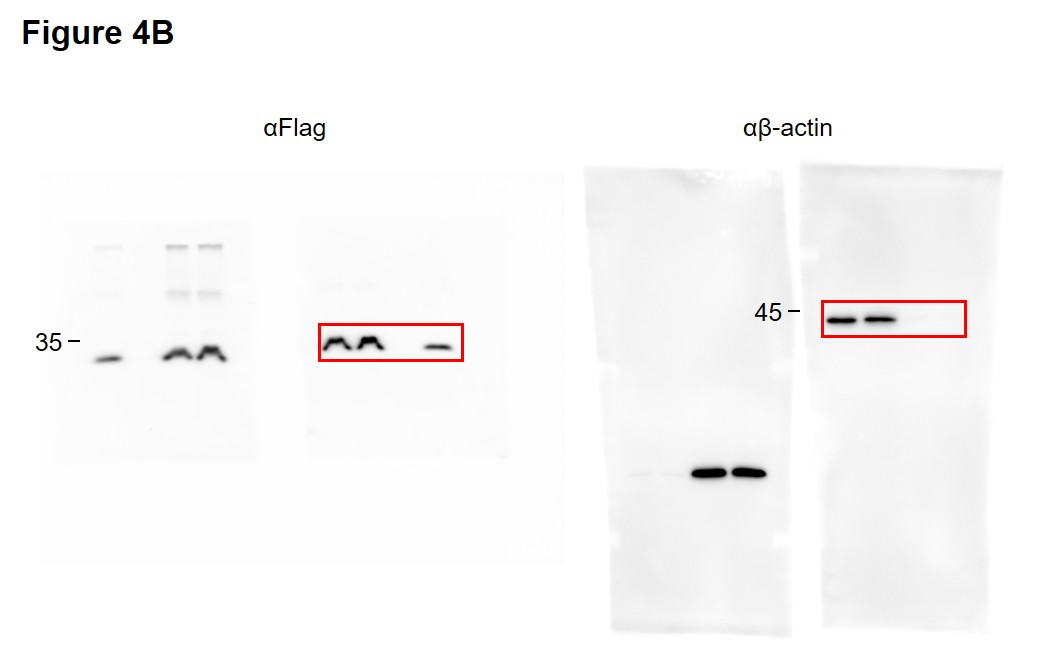

Supplement: Supplementary file 6 — Source data Fig. 4 [file 44321_2026_425_MOESM6_ESM.zip › Figure 4 Source Data/4B/4B.jpg]

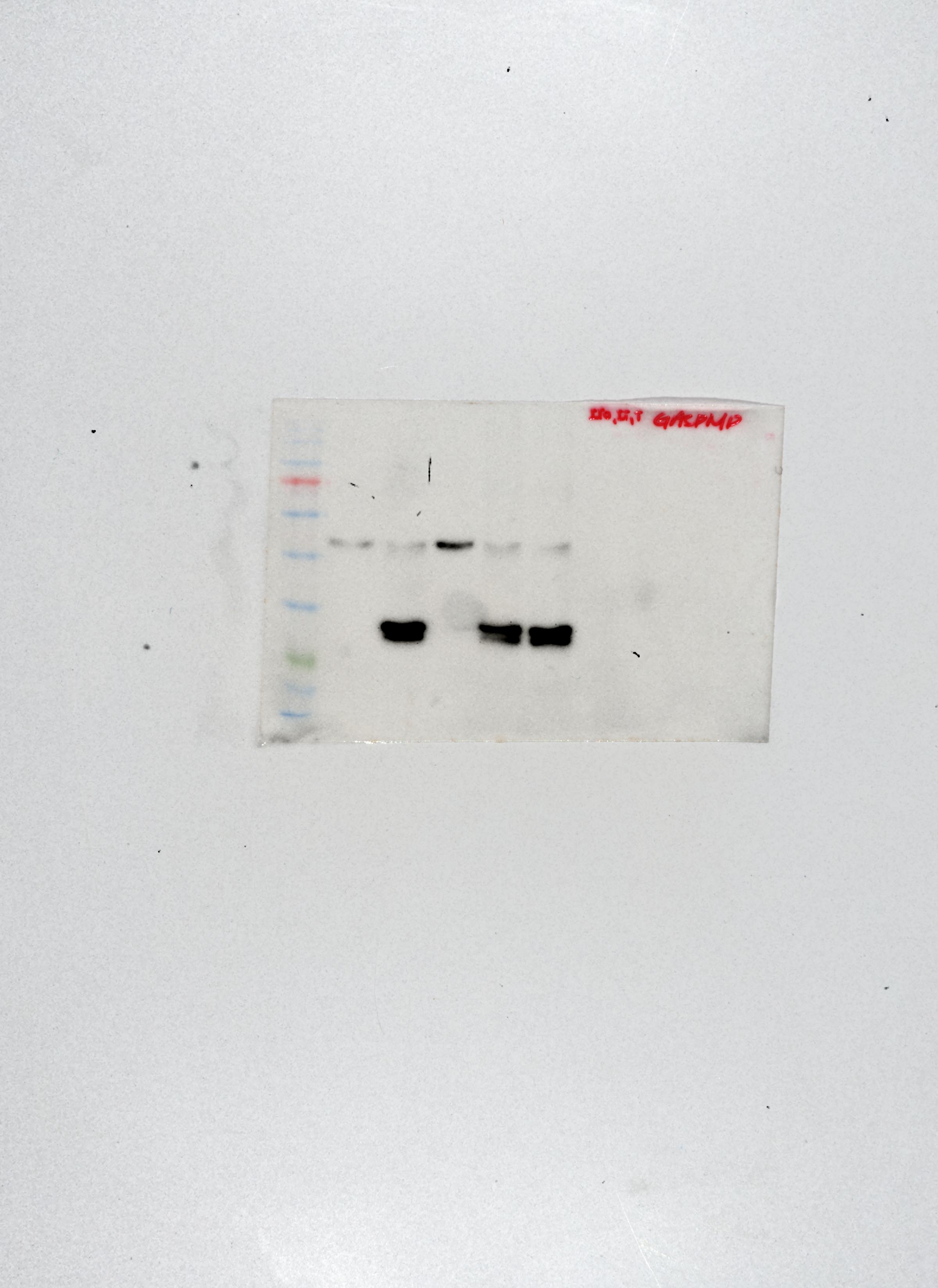

Supplement: Supplementary file 7 — Source data Fig. 5 [file 44321_2026_425_MOESM7_ESM.zip › Figure 5 Source Data/5G/5G_GSDMD.jpg]

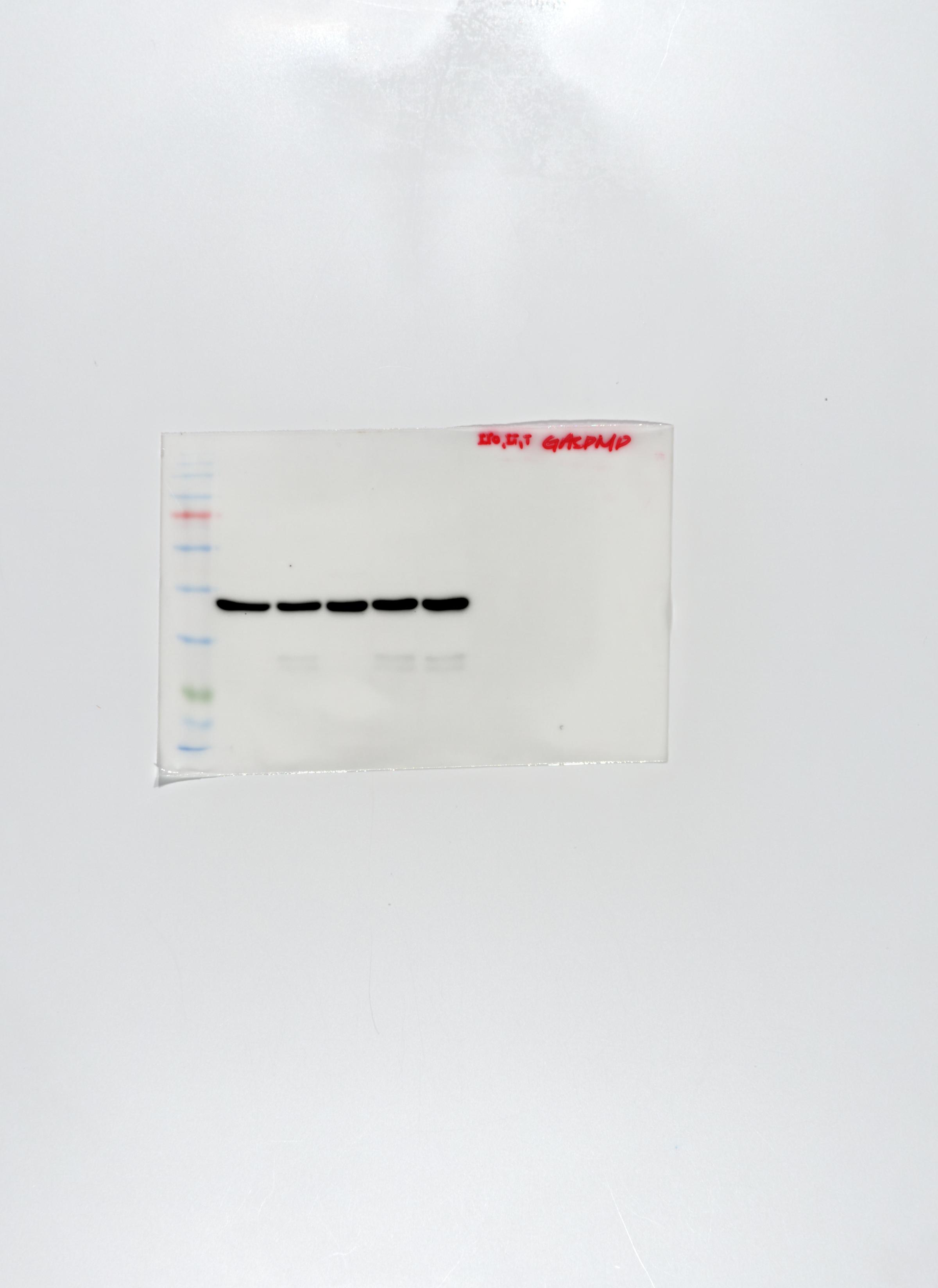

Supplement: Supplementary file 7 — Source data Fig. 5 [file 44321_2026_425_MOESM7_ESM.zip › Figure 5 Source Data/5G/5G_b-actin.jpg]

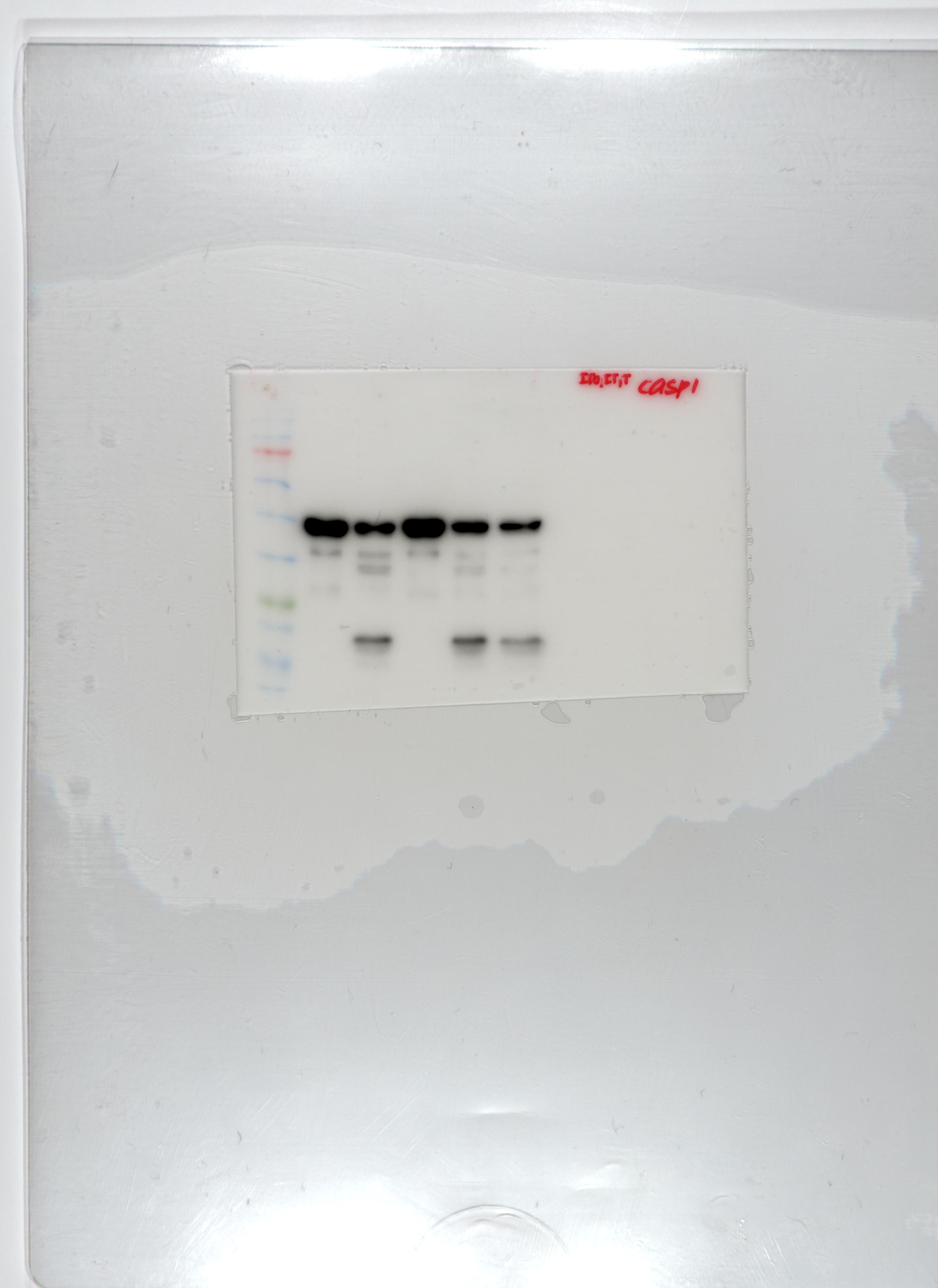

Supplement: Supplementary file 7 — Source data Fig. 5 [file 44321_2026_425_MOESM7_ESM.zip › Figure 5 Source Data/5G/5G_CASP1.jpg]

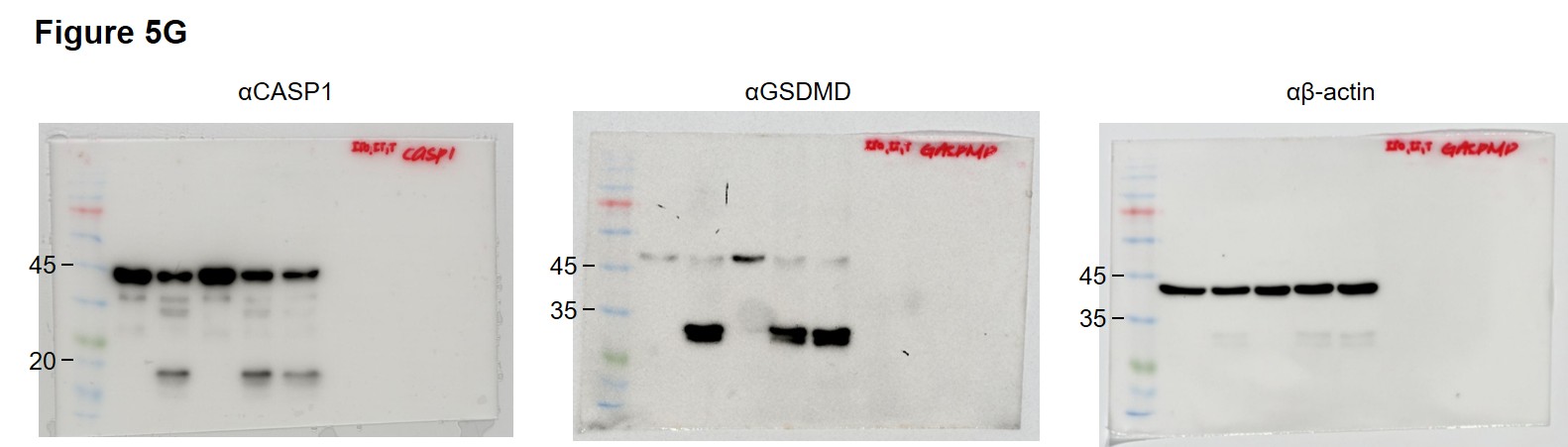

Supplement: Supplementary file 7 — Source data Fig. 5 [file 44321_2026_425_MOESM7_ESM.zip › Figure 5 Source Data/5G/5G.jpg]

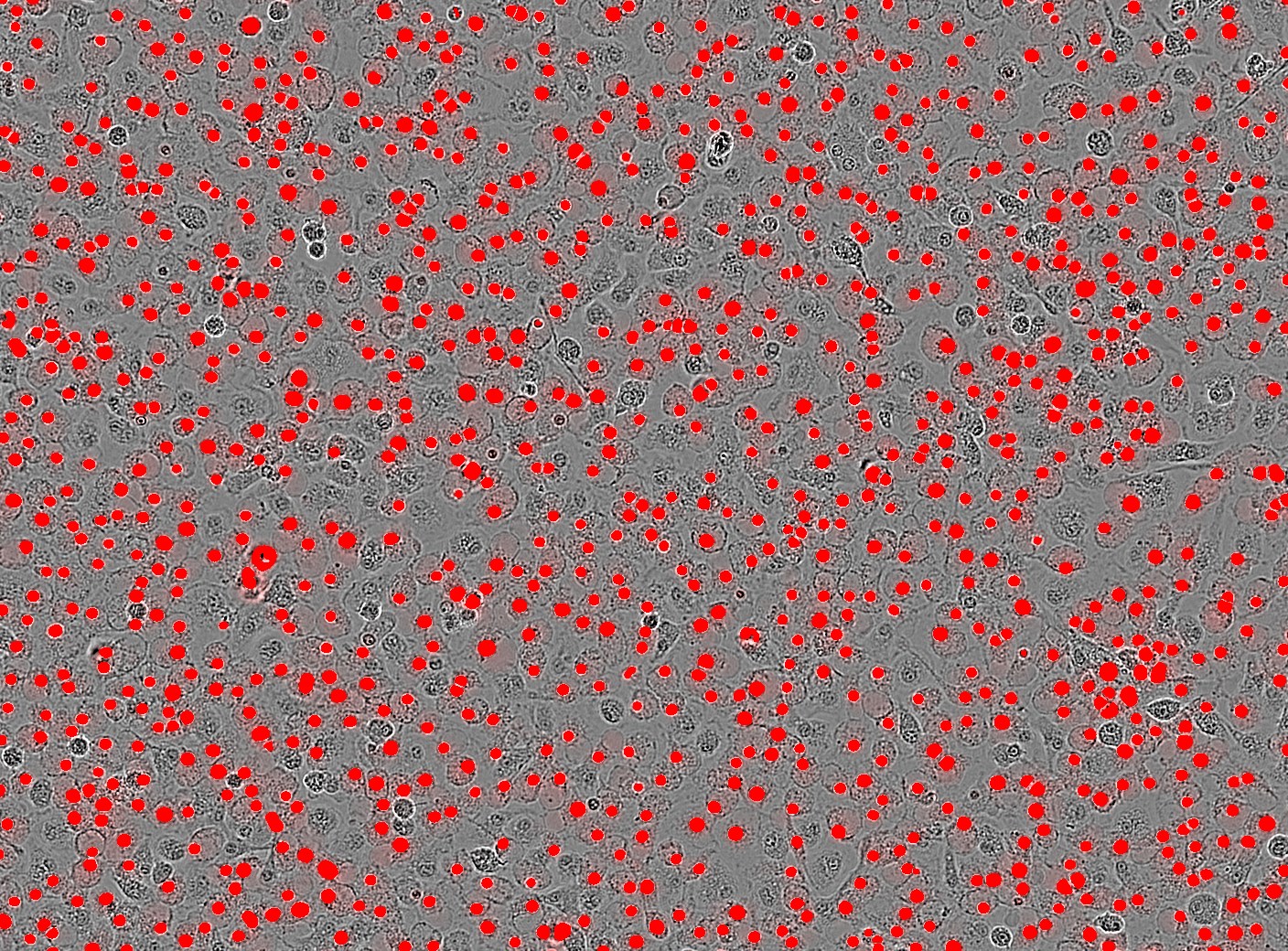

Supplement: Supplementary file 7 — Source data Fig. 5 [file 44321_2026_425_MOESM7_ESM.zip › Figure 5 Source Data/5F/5F_Vehicle.jpg]

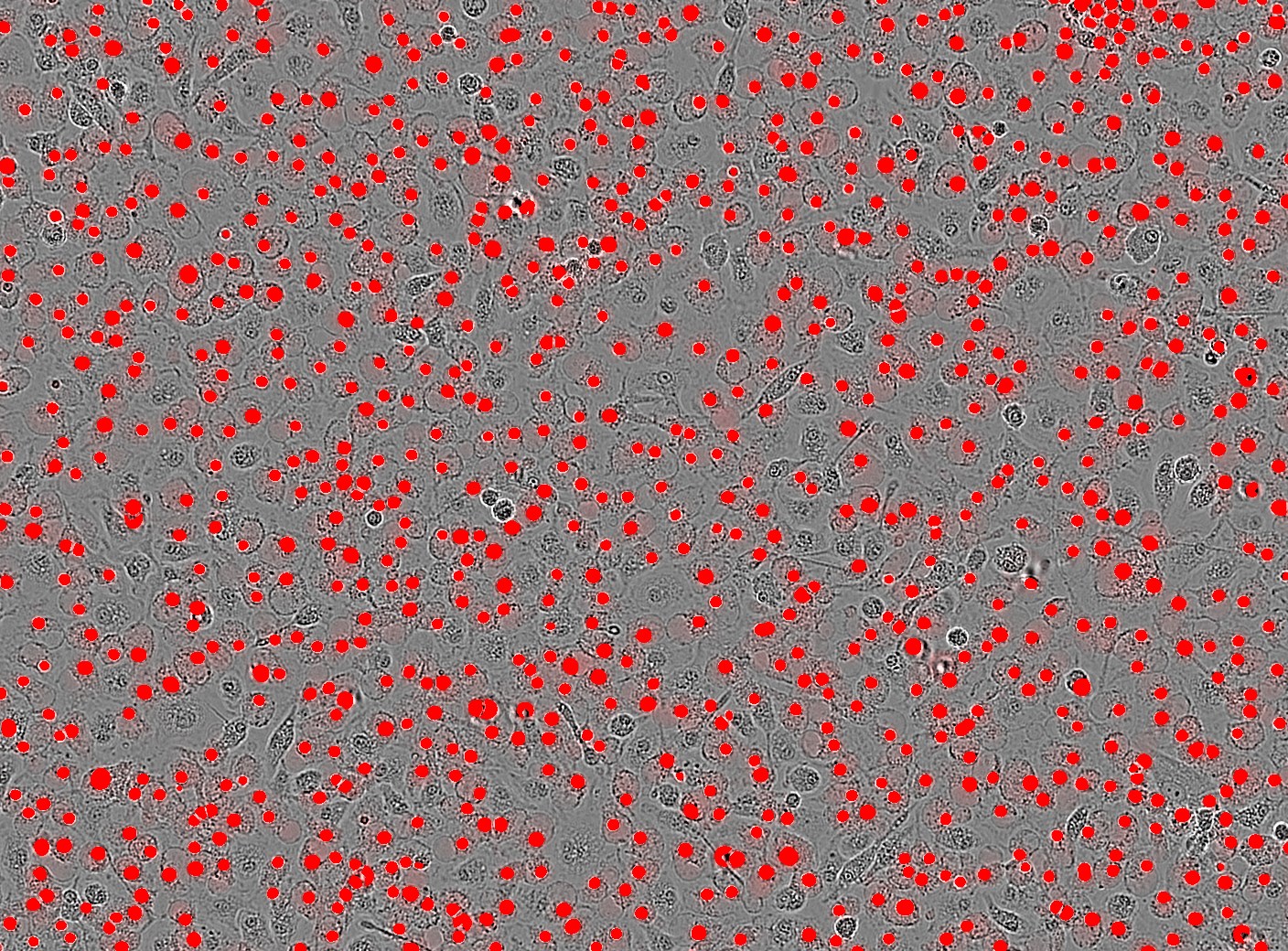

Supplement: Supplementary file 7 — Source data Fig. 5 [file 44321_2026_425_MOESM7_ESM.zip › Figure 5 Source Data/5F/5F_I.jpg]

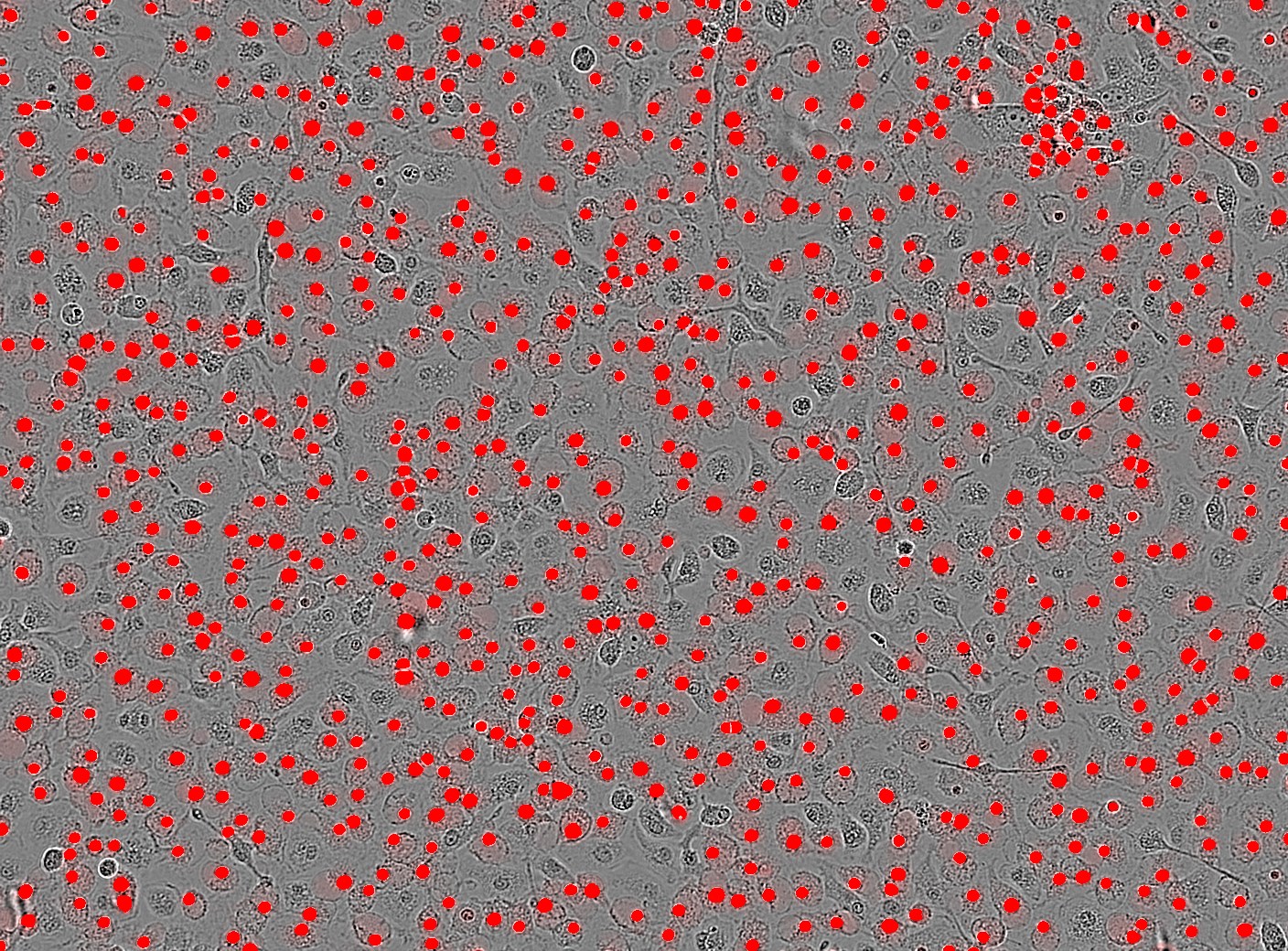

Supplement: Supplementary file 7 — Source data Fig. 5 [file 44321_2026_425_MOESM7_ESM.zip › Figure 5 Source Data/5F/5F_IT.jpg]

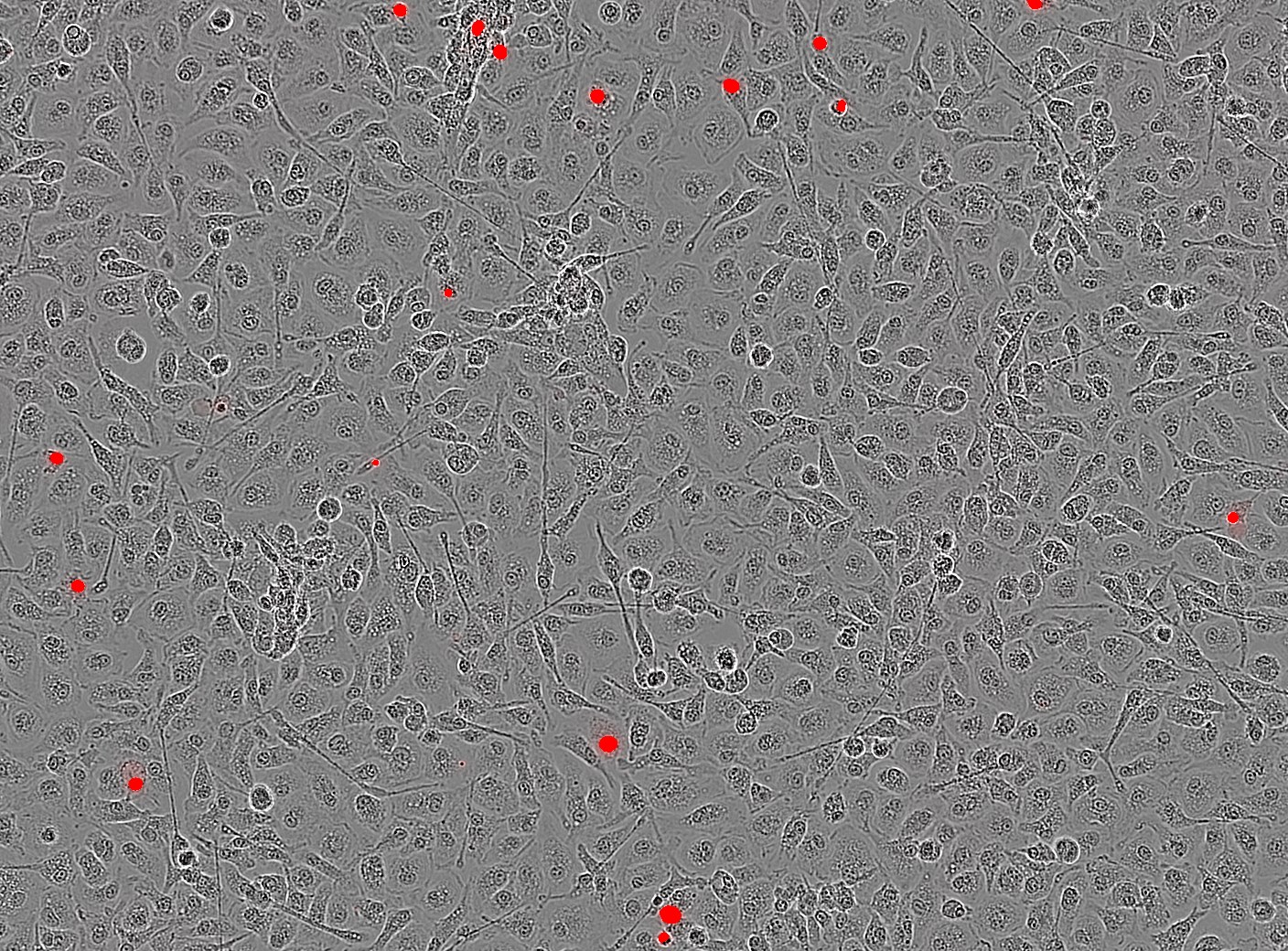

Supplement: Supplementary file 7 — Source data Fig. 5 [file 44321_2026_425_MOESM7_ESM.zip › Figure 5 Source Data/5F/5F_ITO.jpg]

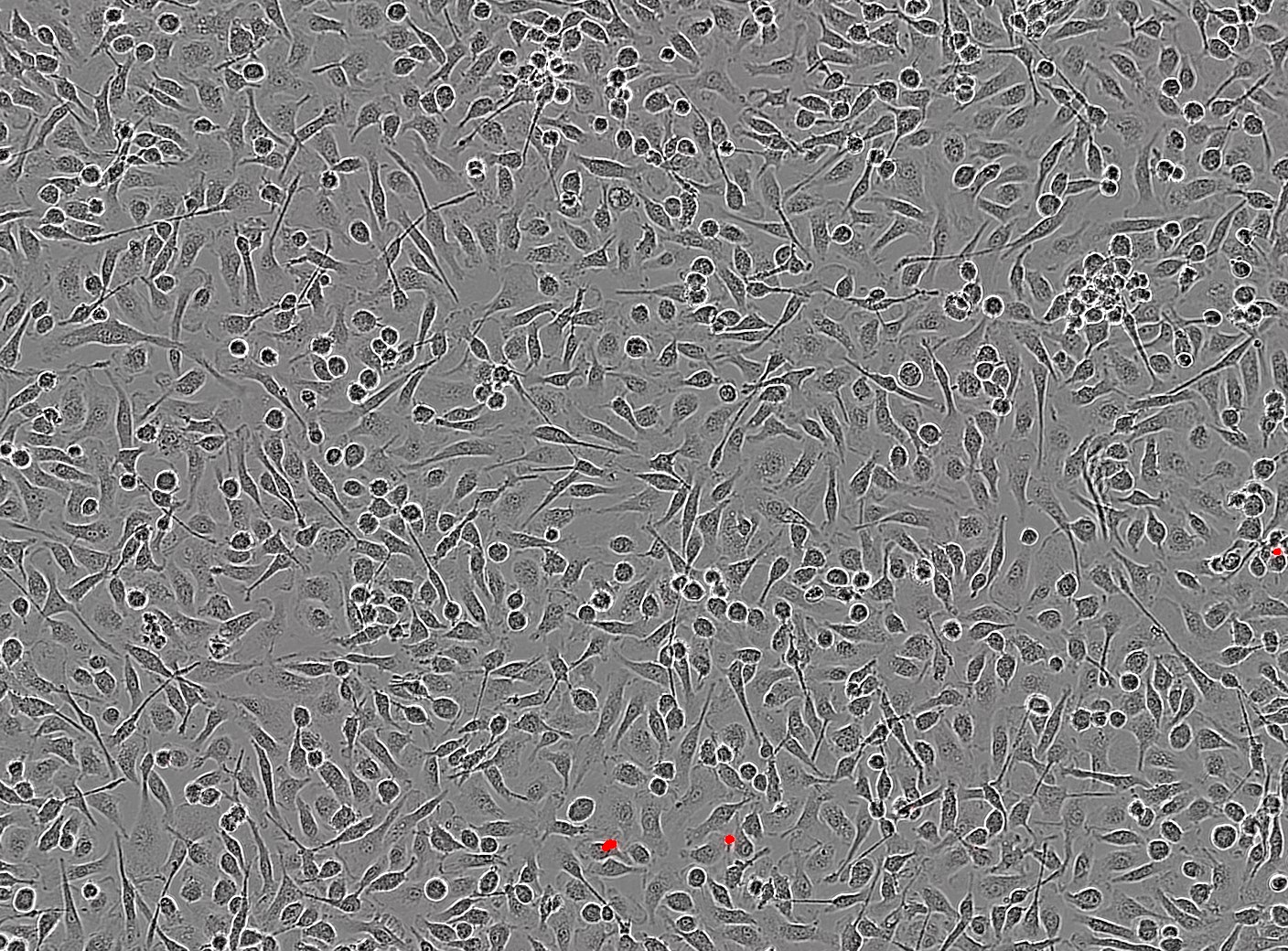

Supplement: Supplementary file 7 — Source data Fig. 5 [file 44321_2026_425_MOESM7_ESM.zip › Figure 5 Source Data/5F/5F_Media.jpg]

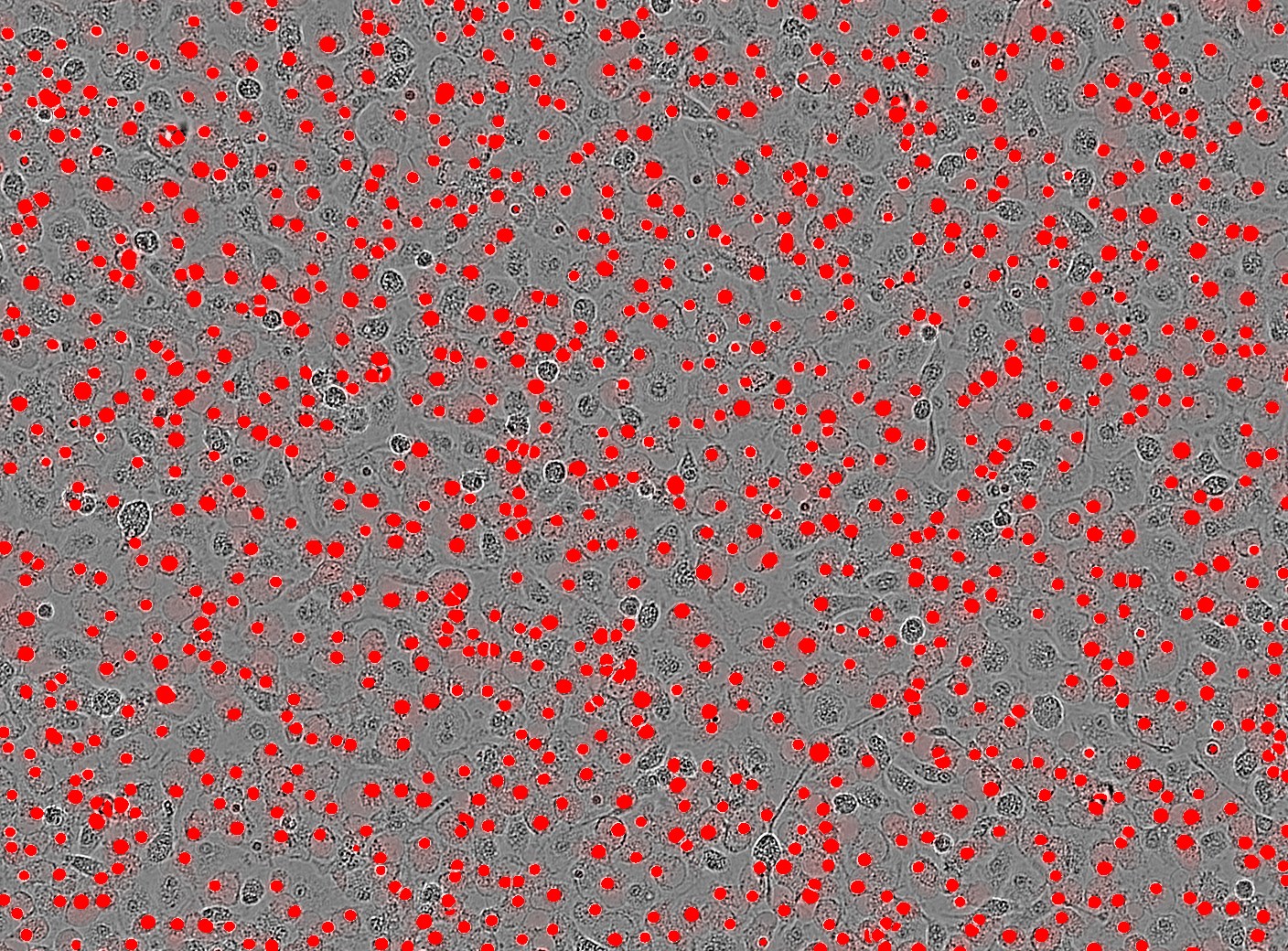

Supplement: Supplementary file 7 — Source data Fig. 5 [file 44321_2026_425_MOESM7_ESM.zip › Figure 5 Source Data/5F/5F_T.jpg]

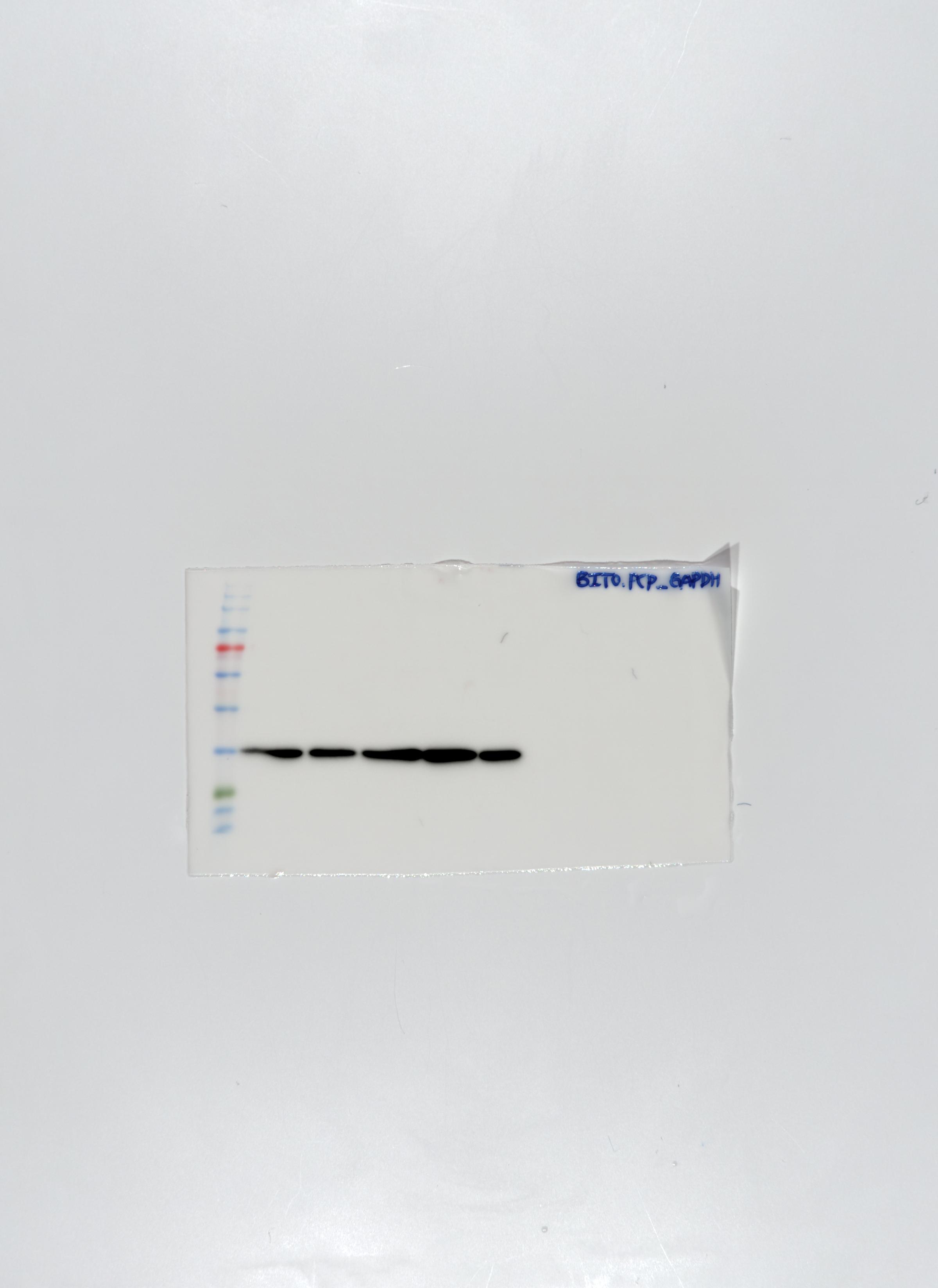

Supplement: Supplementary file 7 — Source data Fig. 5 [file 44321_2026_425_MOESM7_ESM.zip › Figure 5 Source Data/5C/5C_GAPDH.jpg]

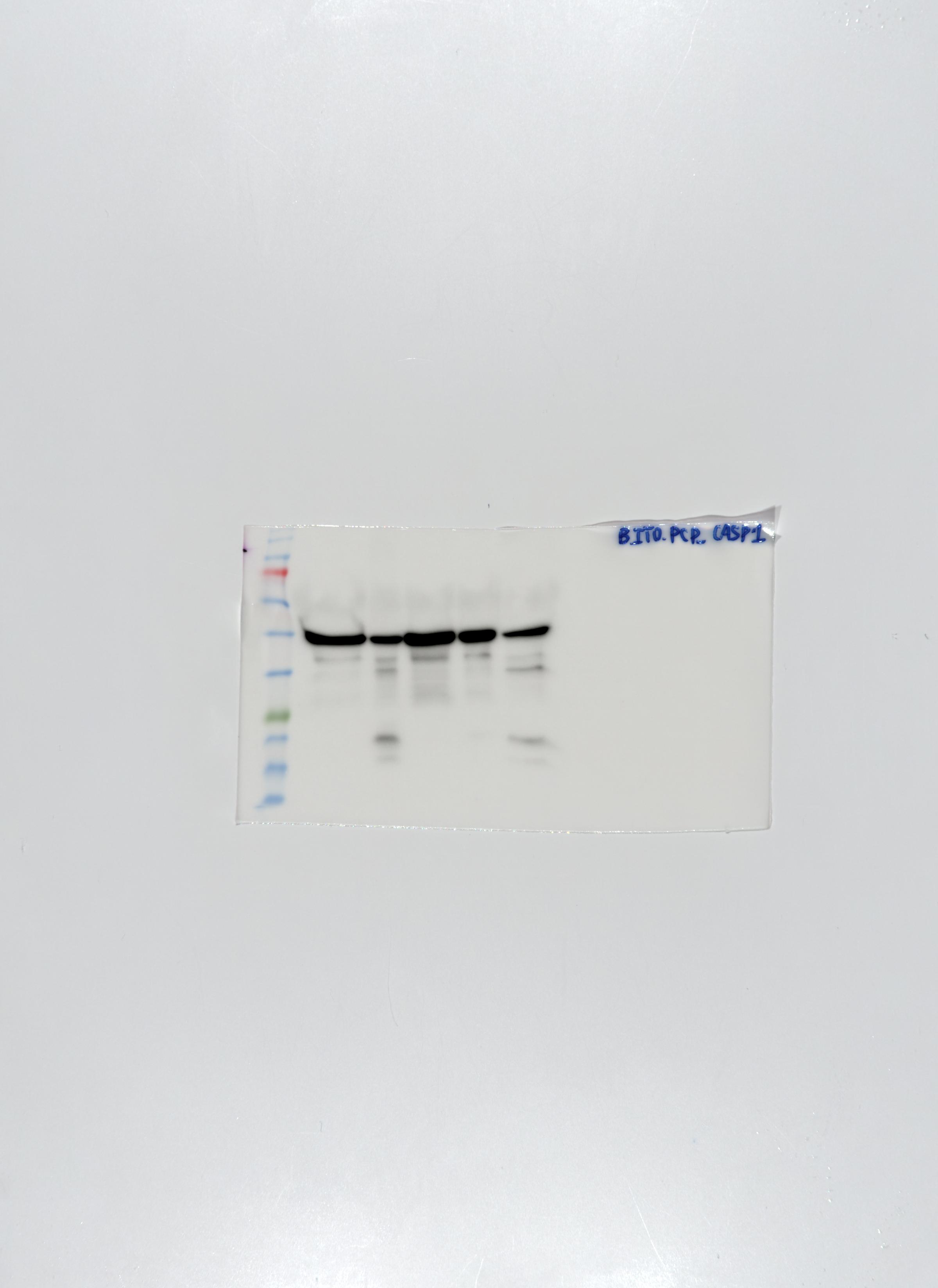

Supplement: Supplementary file 7 — Source data Fig. 5 [file 44321_2026_425_MOESM7_ESM.zip › Figure 5 Source Data/5C/5C_CASP1.jpg]

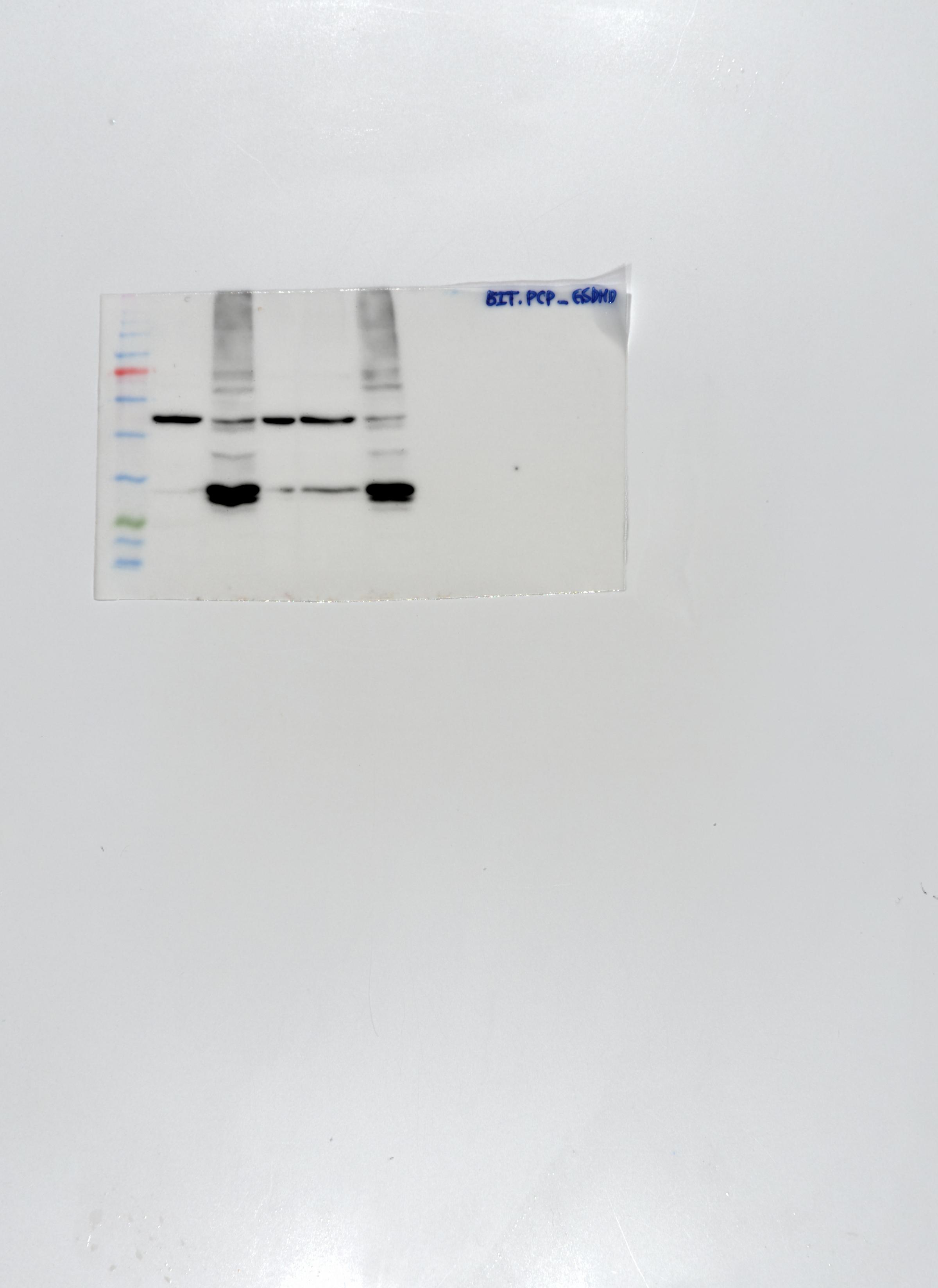

Supplement: Supplementary file 7 — Source data Fig. 5 [file 44321_2026_425_MOESM7_ESM.zip › Figure 5 Source Data/5C/5C_GSDMD.jpg]

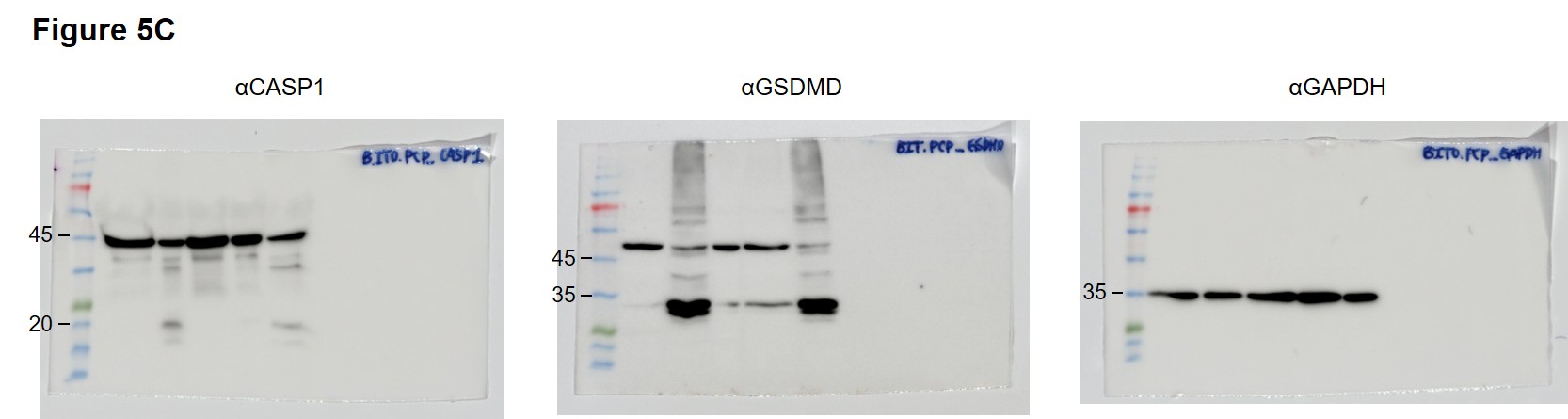

Supplement: Supplementary file 7 — Source data Fig. 5 [file 44321_2026_425_MOESM7_ESM.zip › Figure 5 Source Data/5C/5C.jpg]

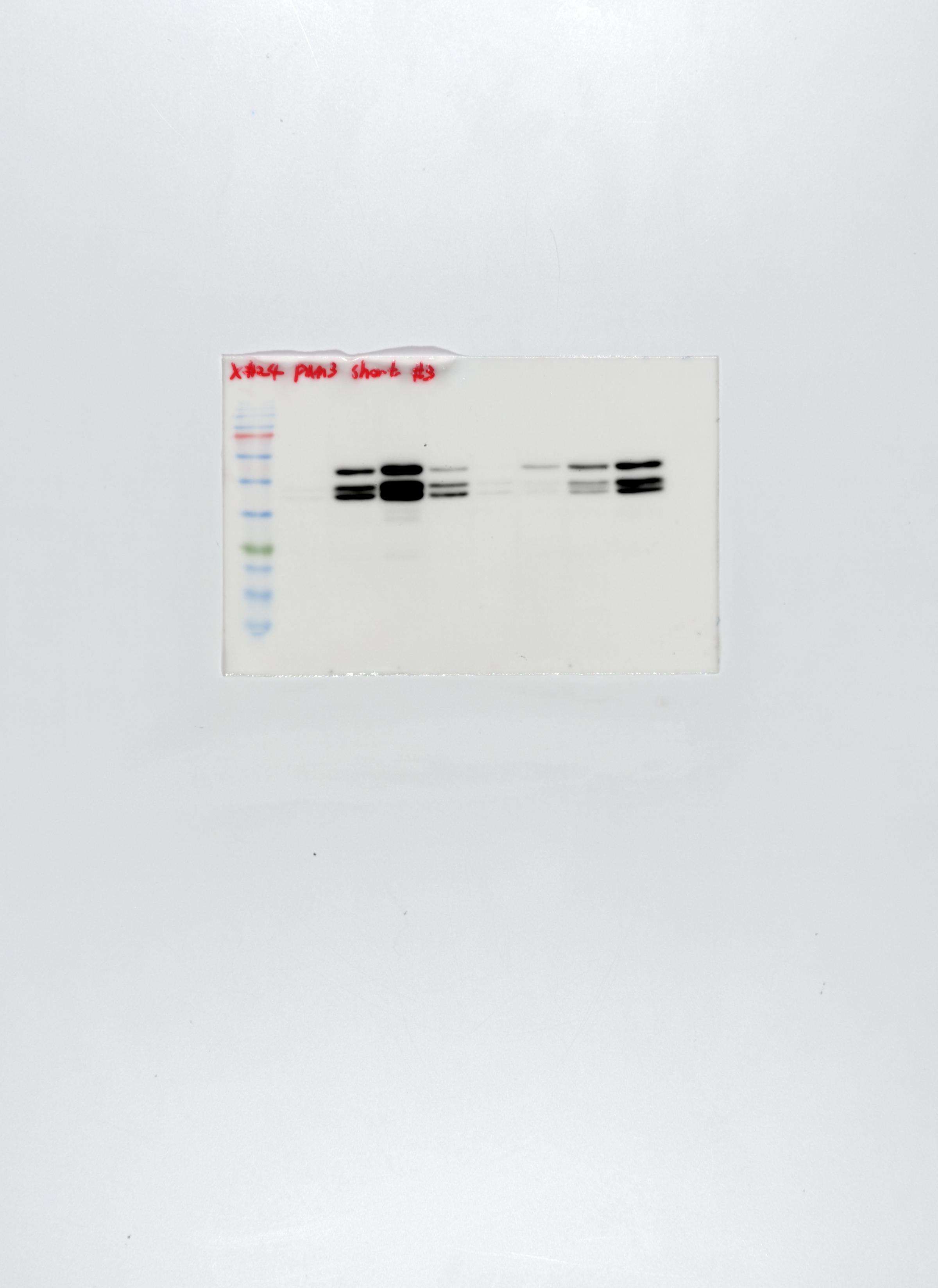

Supplement: Supplementary file 8 — Source data Fig. 6 [file 44321_2026_425_MOESM8_ESM.zip › Figure 6 Source Data/6F/6F_p-JNK.jpg]

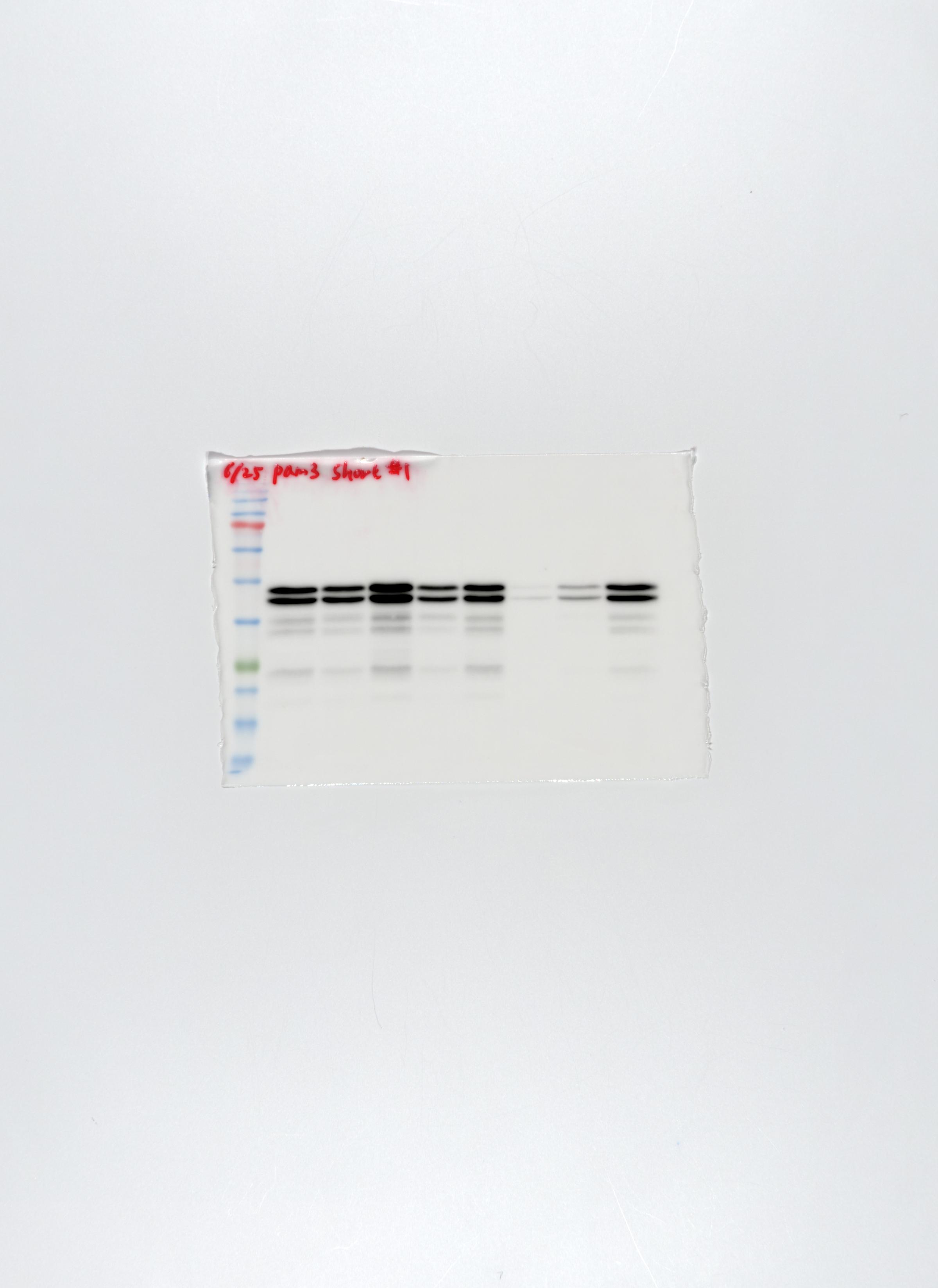

Supplement: Supplementary file 8 — Source data Fig. 6 [file 44321_2026_425_MOESM8_ESM.zip › Figure 6 Source Data/6F/6F_p-ERK.jpg]

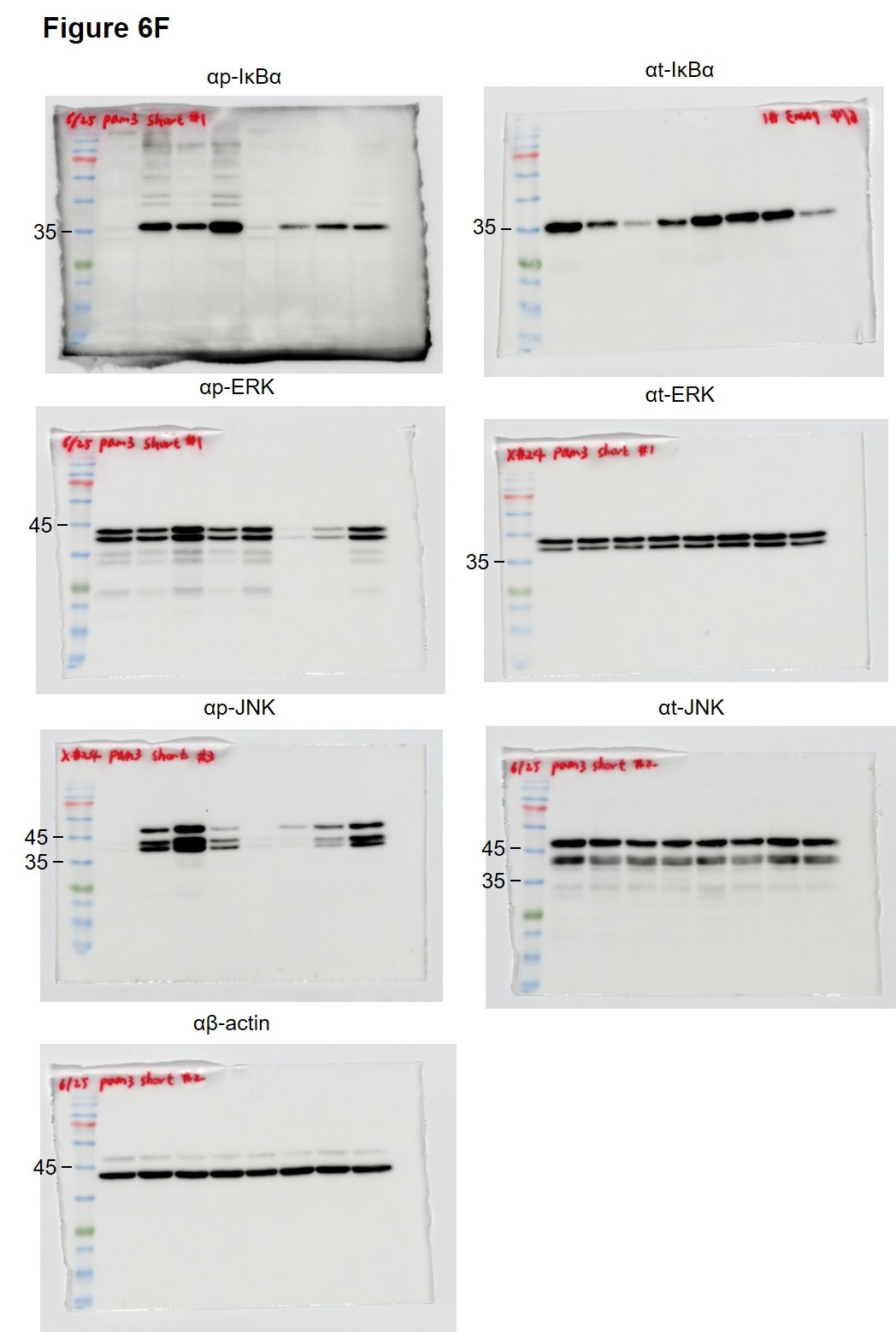

Supplement: Supplementary file 8 — Source data Fig. 6 [file 44321_2026_425_MOESM8_ESM.zip › Figure 6 Source Data/6F/6F.jpg]

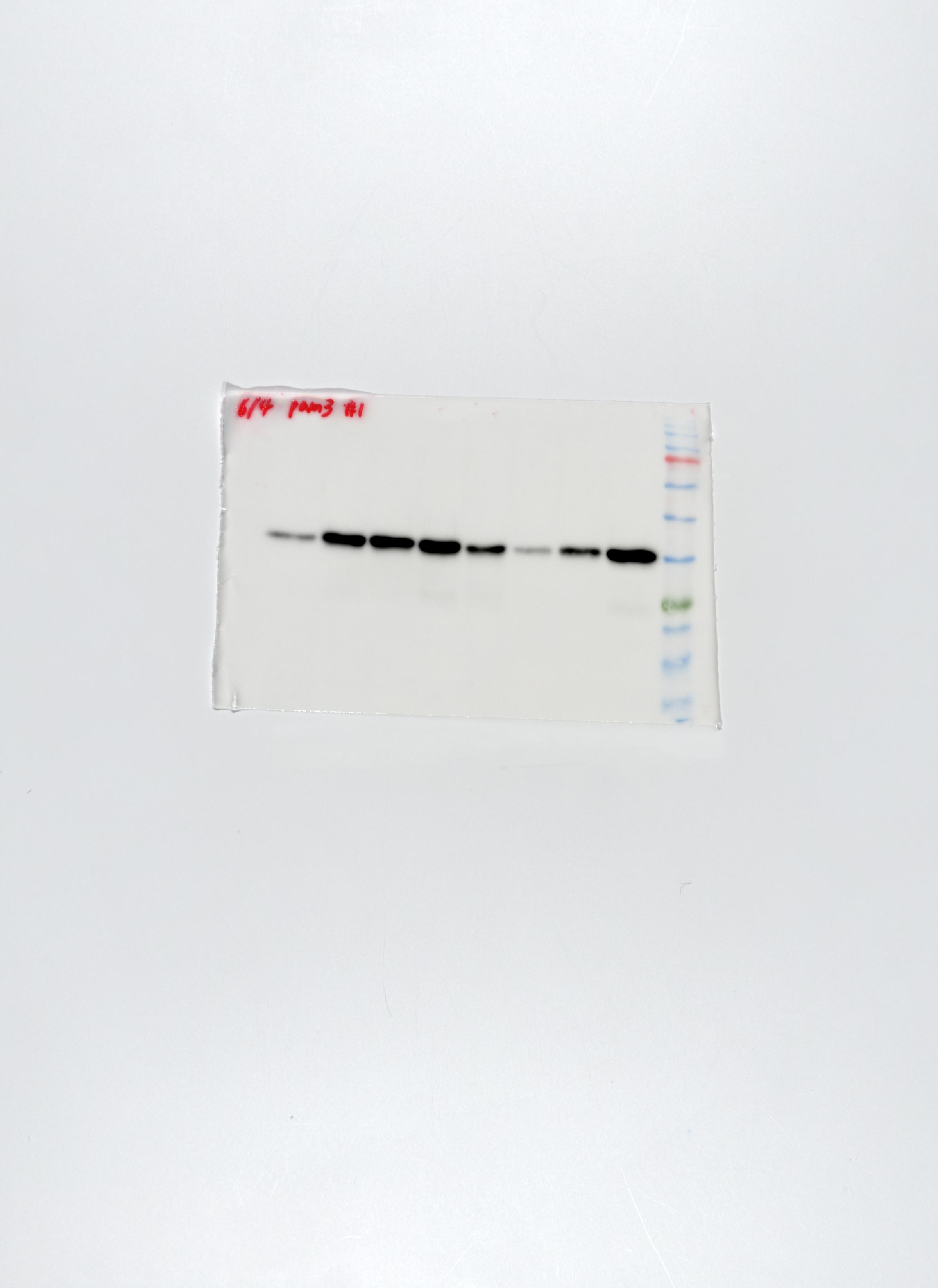

Supplement: Supplementary file 8 — Source data Fig. 6 [file 44321_2026_425_MOESM8_ESM.zip › Figure 6 Source Data/6F/6F_t-IKBa.jpg]

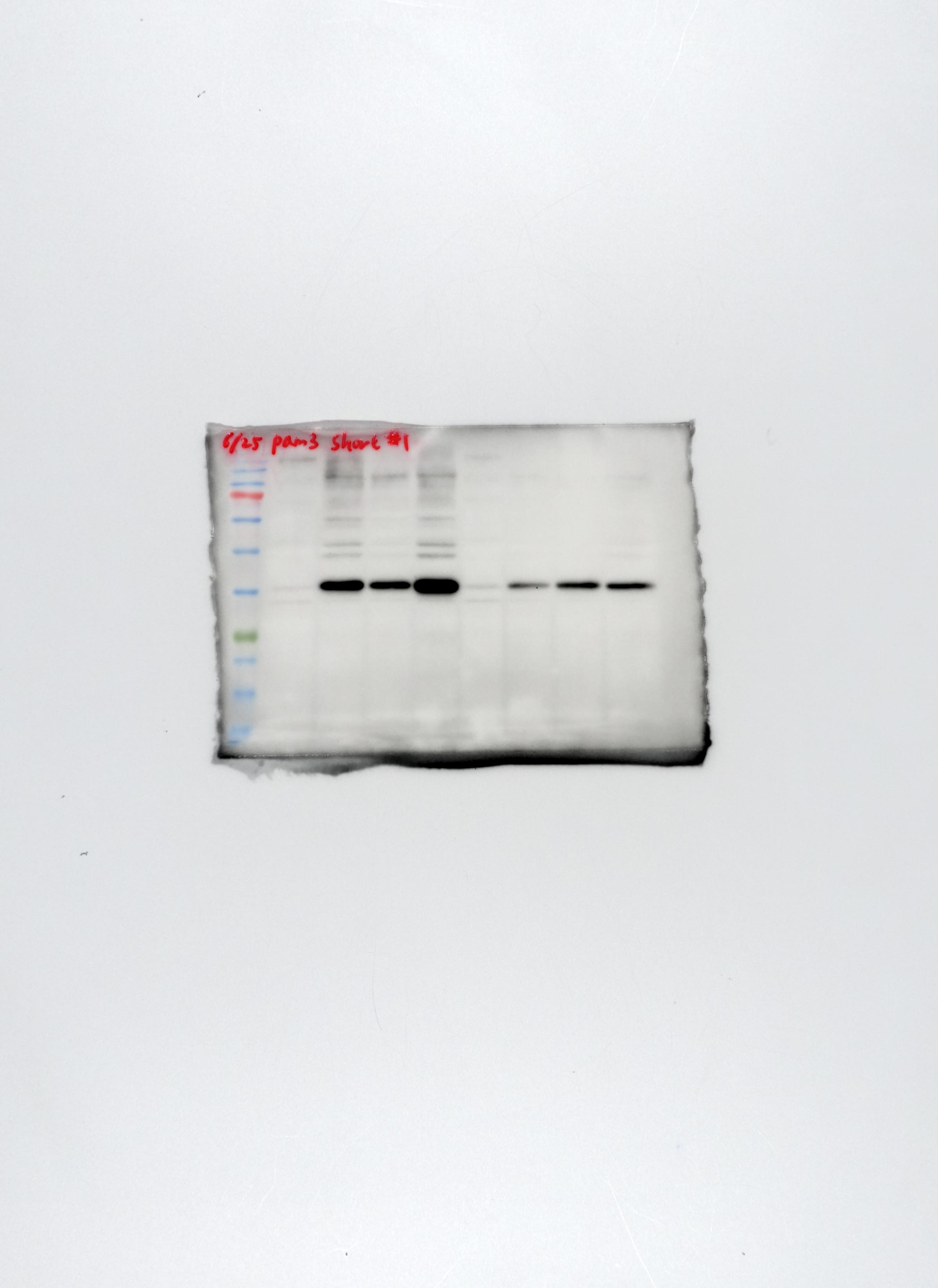

Supplement: Supplementary file 8 — Source data Fig. 6 [file 44321_2026_425_MOESM8_ESM.zip › Figure 6 Source Data/6F/6F_p-IKBa.jpg]

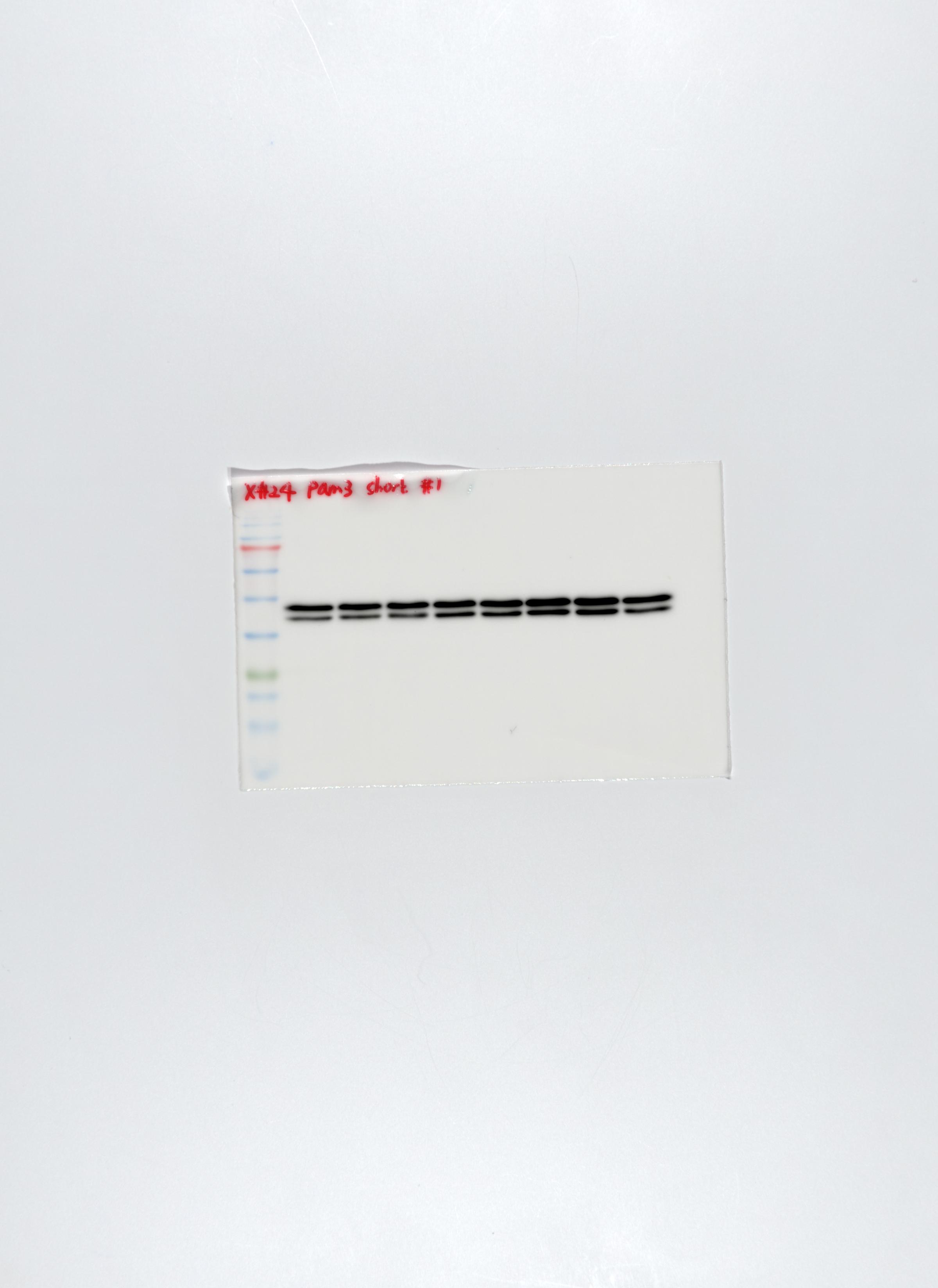

Supplement: Supplementary file 8 — Source data Fig. 6 [file 44321_2026_425_MOESM8_ESM.zip › Figure 6 Source Data/6F/6F_t-ERK.jpg]

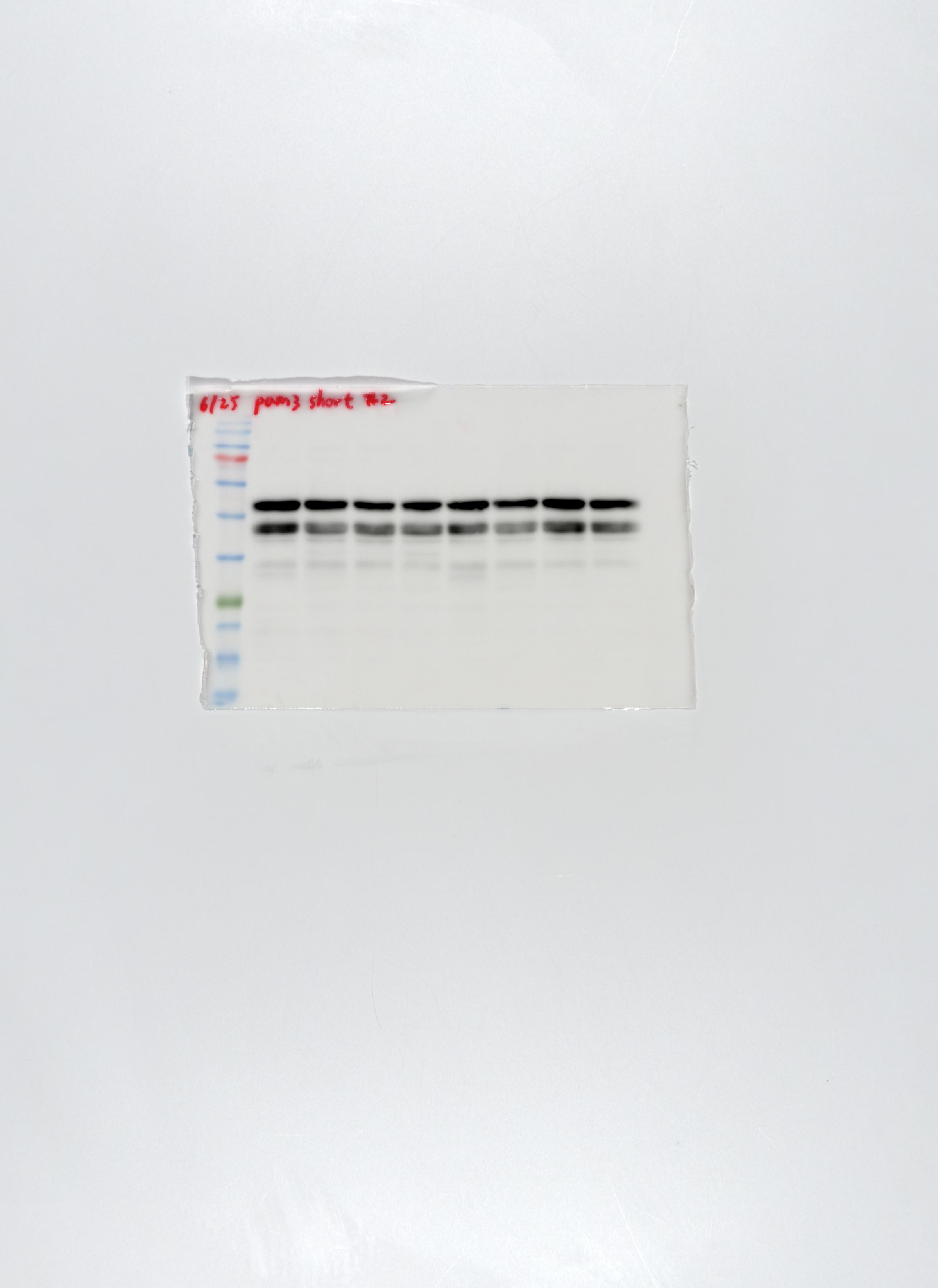

Supplement: Supplementary file 8 — Source data Fig. 6 [file 44321_2026_425_MOESM8_ESM.zip › Figure 6 Source Data/6F/6F_t-JNK.jpg]

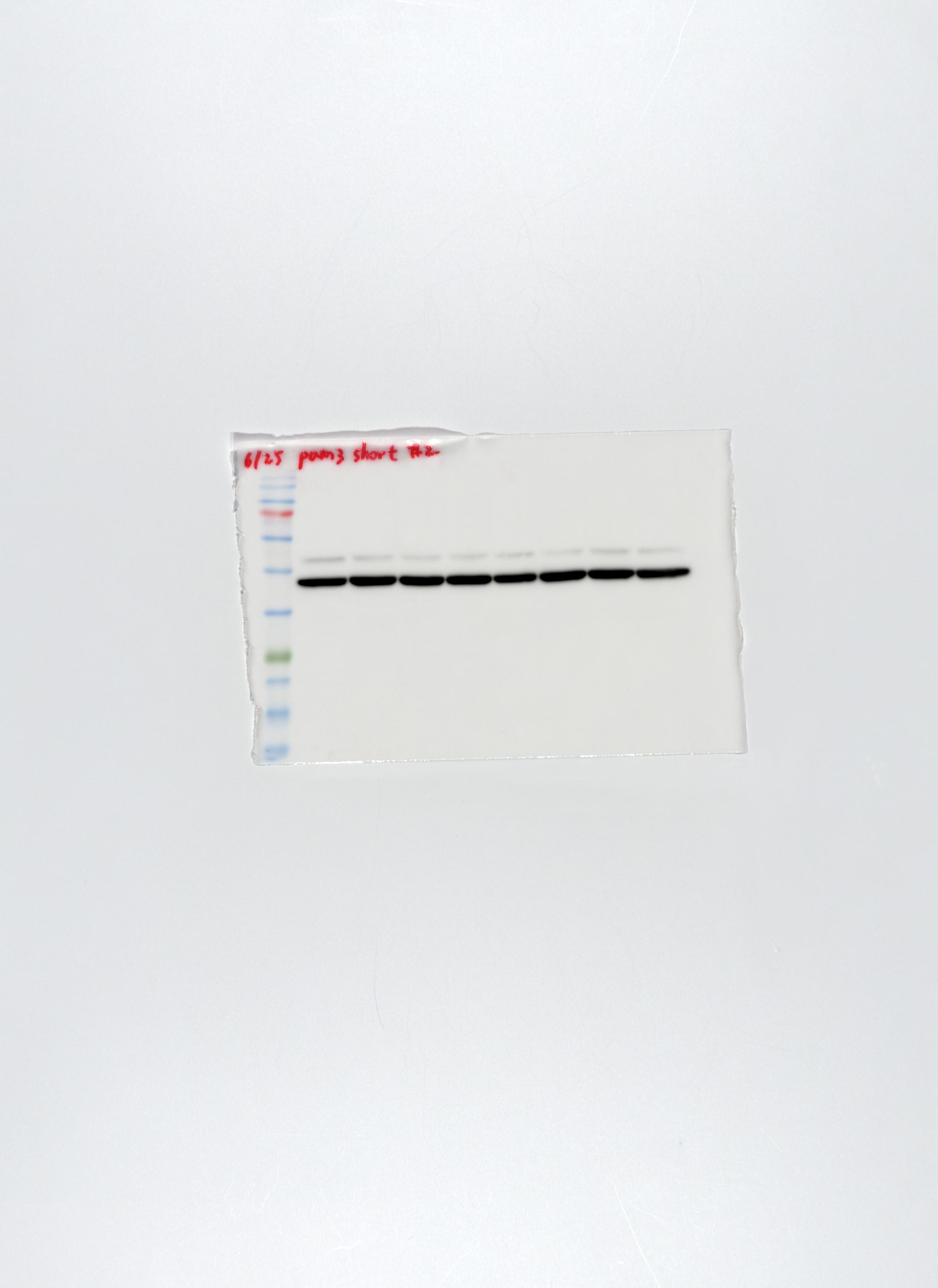

Supplement: Supplementary file 8 — Source data Fig. 6 [file 44321_2026_425_MOESM8_ESM.zip › Figure 6 Source Data/6F/6F_b-actin.jpg]

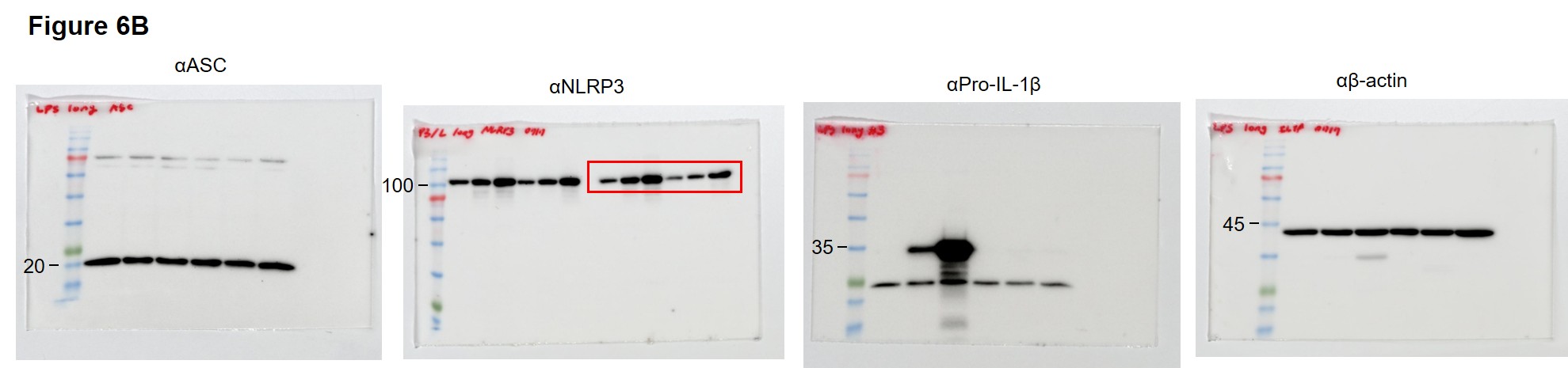

Supplement: Supplementary file 8 — Source data Fig. 6 [file 44321_2026_425_MOESM8_ESM.zip › Figure 6 Source Data/6B/6B.jpg]

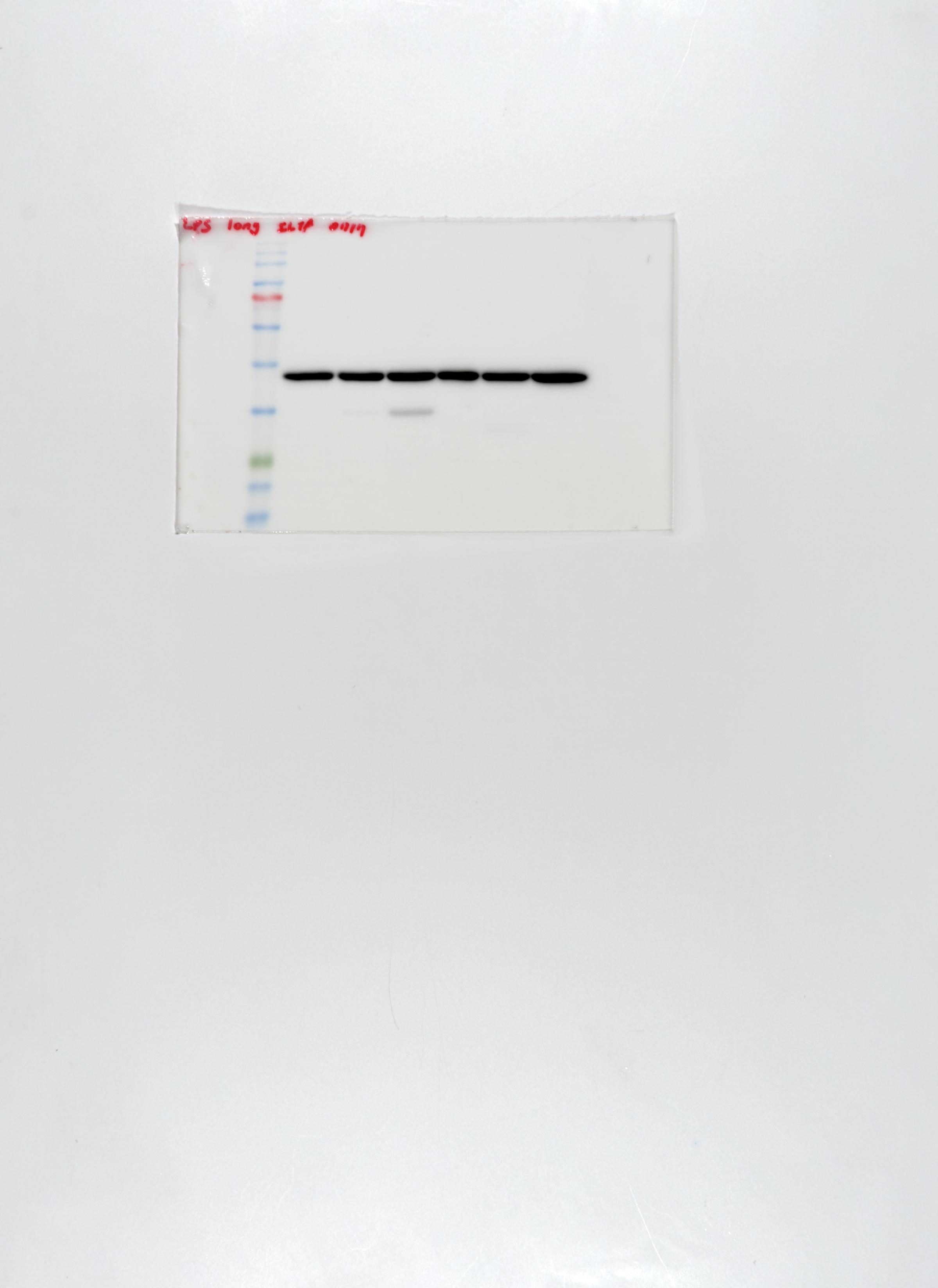

Supplement: Supplementary file 8 — Source data Fig. 6 [file 44321_2026_425_MOESM8_ESM.zip › Figure 6 Source Data/6B/6B_b-actin.jpg]

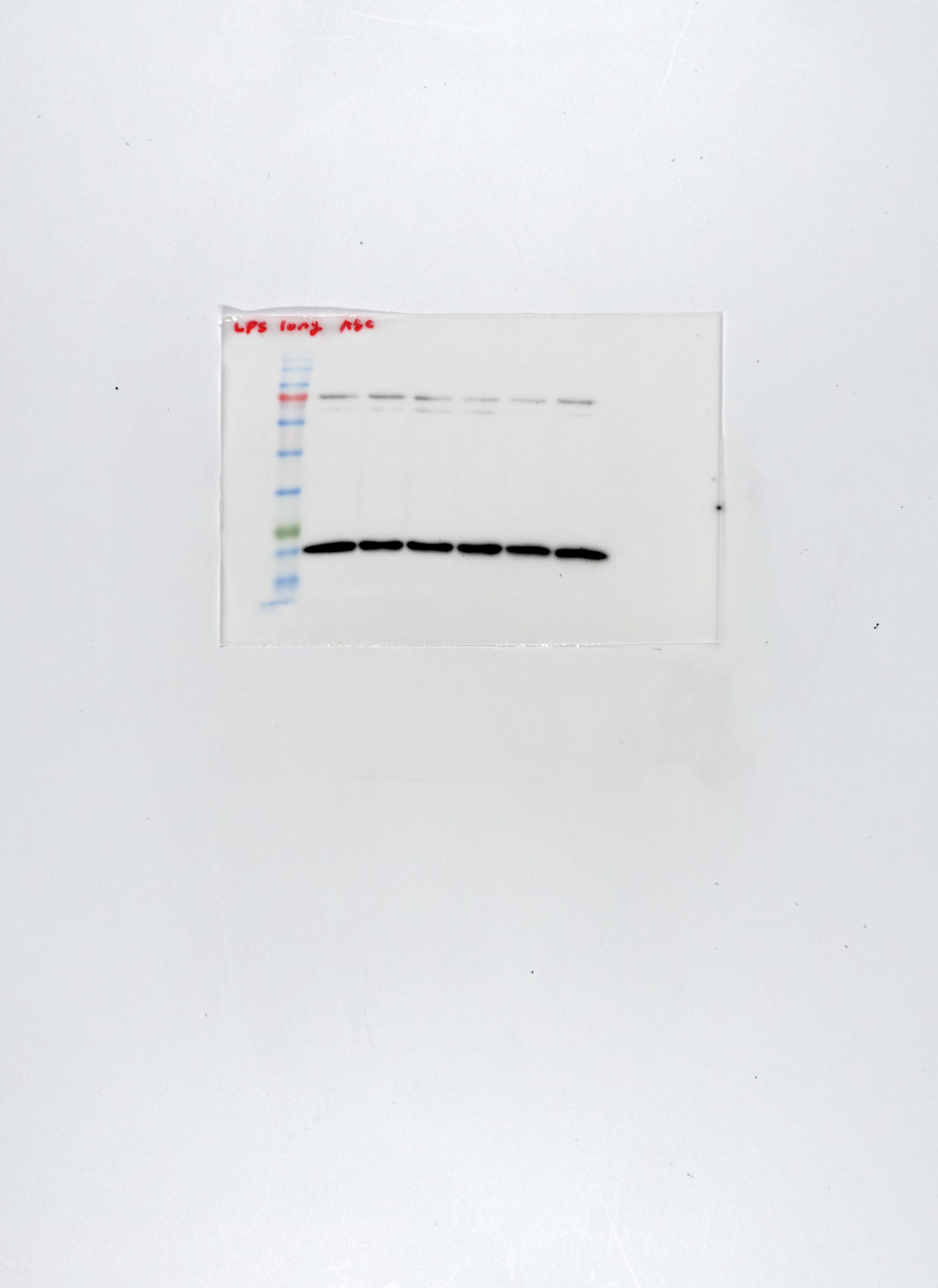

Supplement: Supplementary file 8 — Source data Fig. 6 [file 44321_2026_425_MOESM8_ESM.zip › Figure 6 Source Data/6B/6B_ASC.jpg]

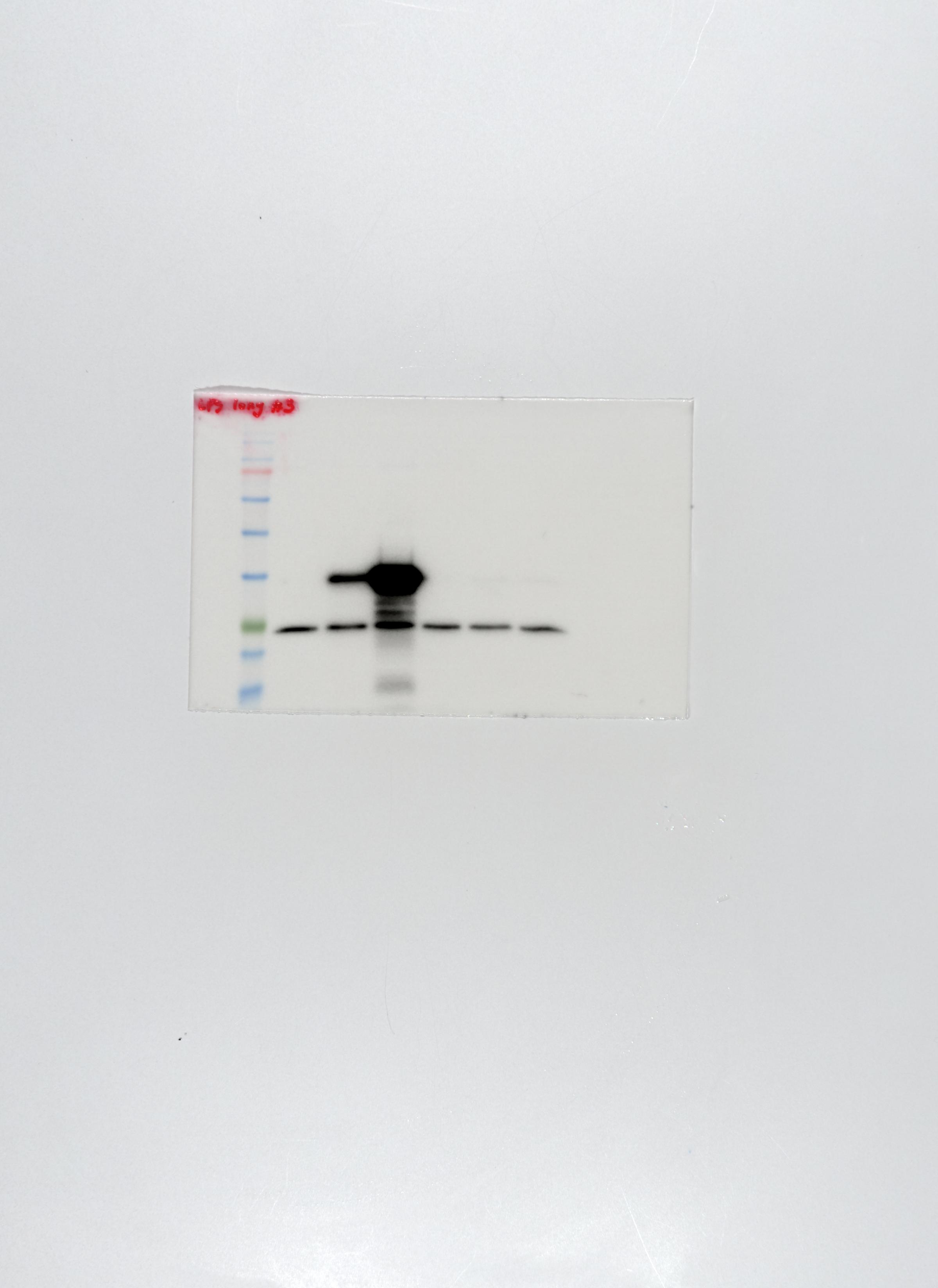

Supplement: Supplementary file 8 — Source data Fig. 6 [file 44321_2026_425_MOESM8_ESM.zip › Figure 6 Source Data/6B/6B_IL-1b.jpg]

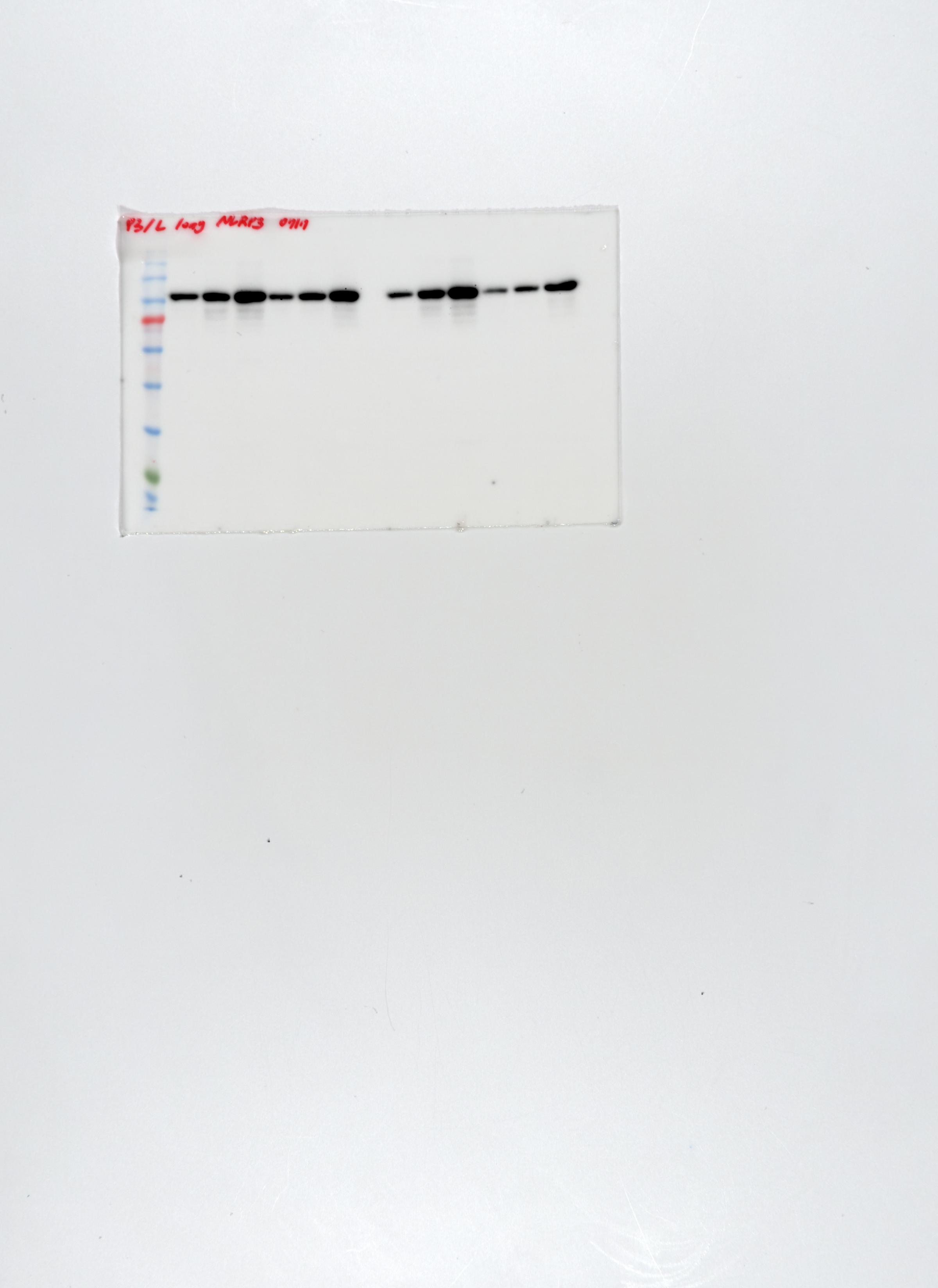

Supplement: Supplementary file 8 — Source data Fig. 6 [file 44321_2026_425_MOESM8_ESM.zip › Figure 6 Source Data/6B/6B_NLRP3.jpg]

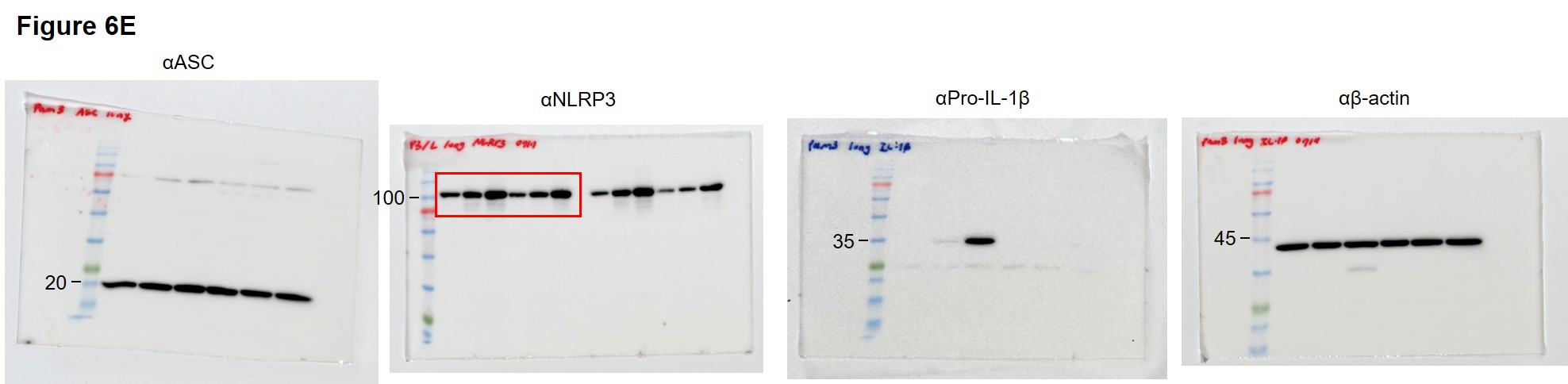

Supplement: Supplementary file 8 — Source data Fig. 6 [file 44321_2026_425_MOESM8_ESM.zip › Figure 6 Source Data/6E/6E.jpg]

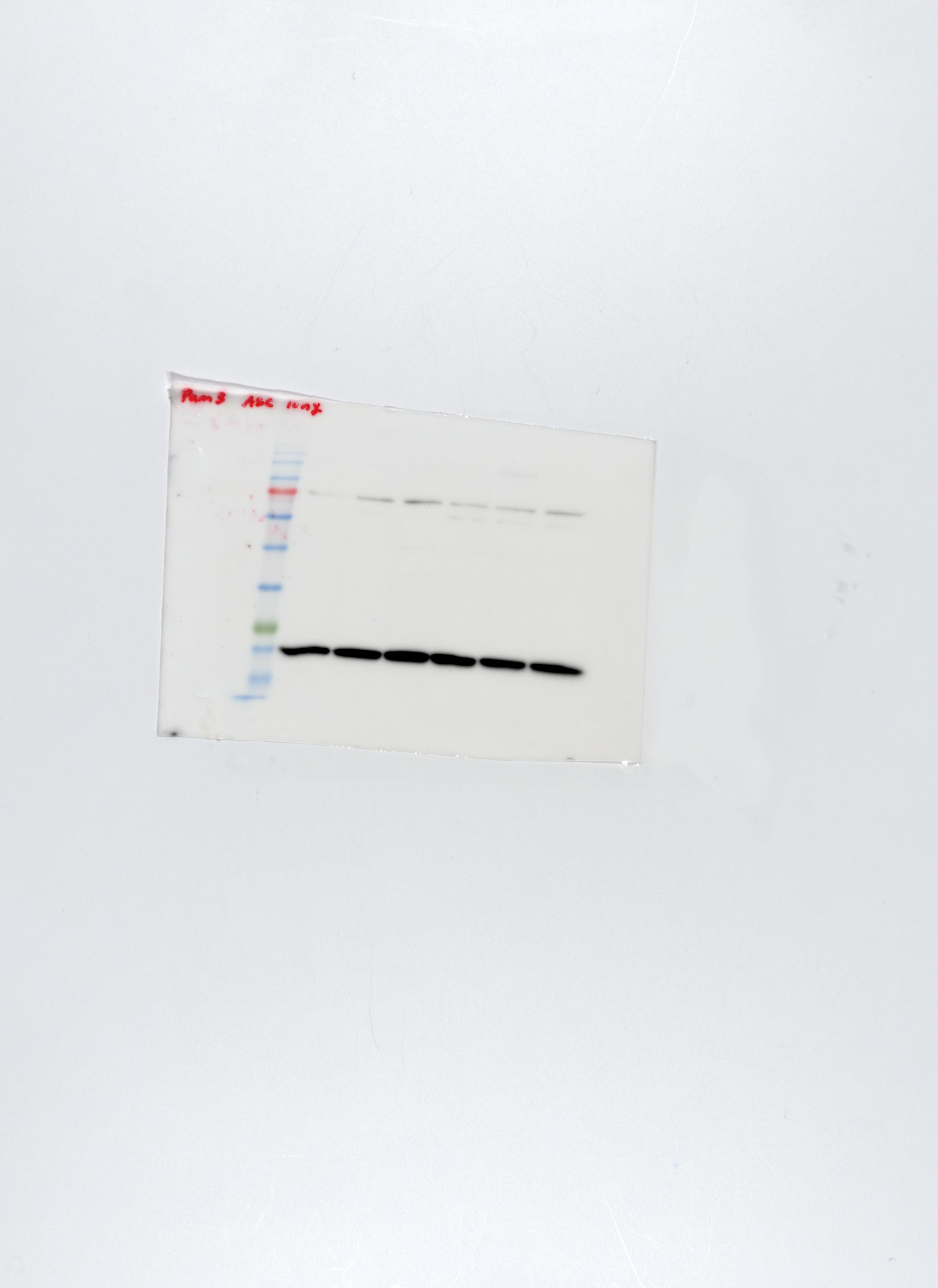

Supplement: Supplementary file 8 — Source data Fig. 6 [file 44321_2026_425_MOESM8_ESM.zip › Figure 6 Source Data/6E/6E_ASC.jpg]

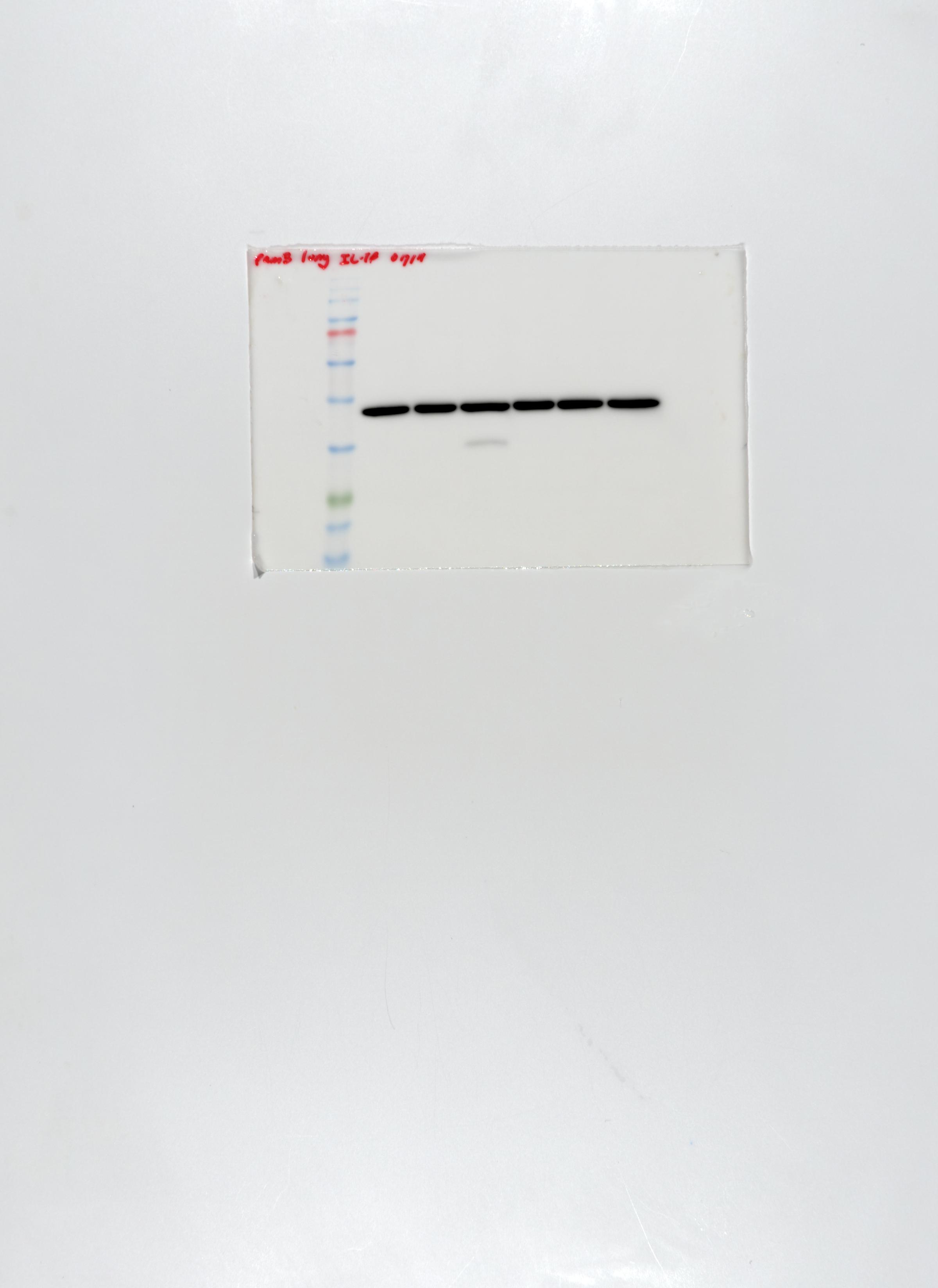

Supplement: Supplementary file 8 — Source data Fig. 6 [file 44321_2026_425_MOESM8_ESM.zip › Figure 6 Source Data/6E/6E_b-actin.jpg]

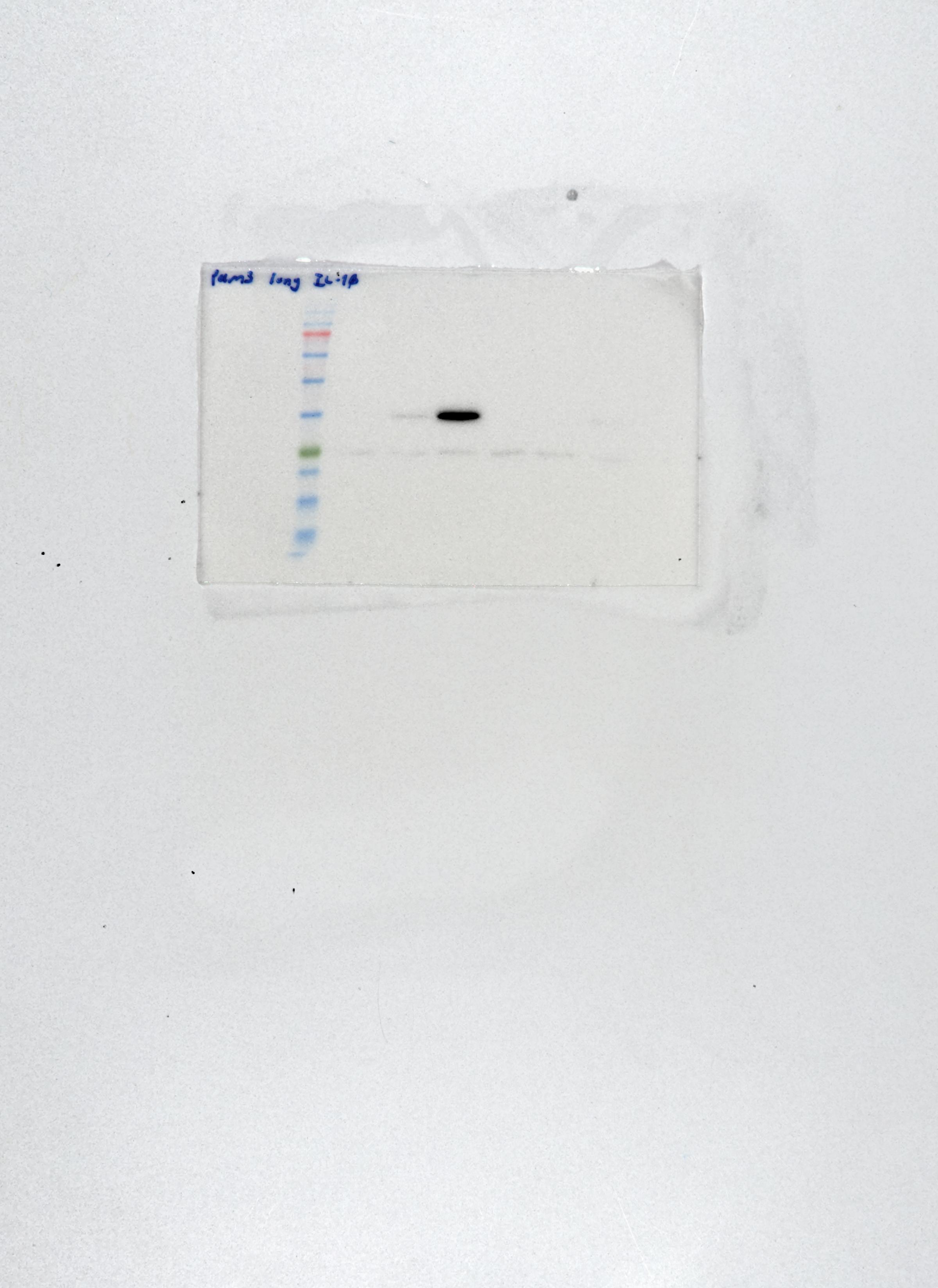

Supplement: Supplementary file 8 — Source data Fig. 6 [file 44321_2026_425_MOESM8_ESM.zip › Figure 6 Source Data/6E/6E_IL-1b.jpg]

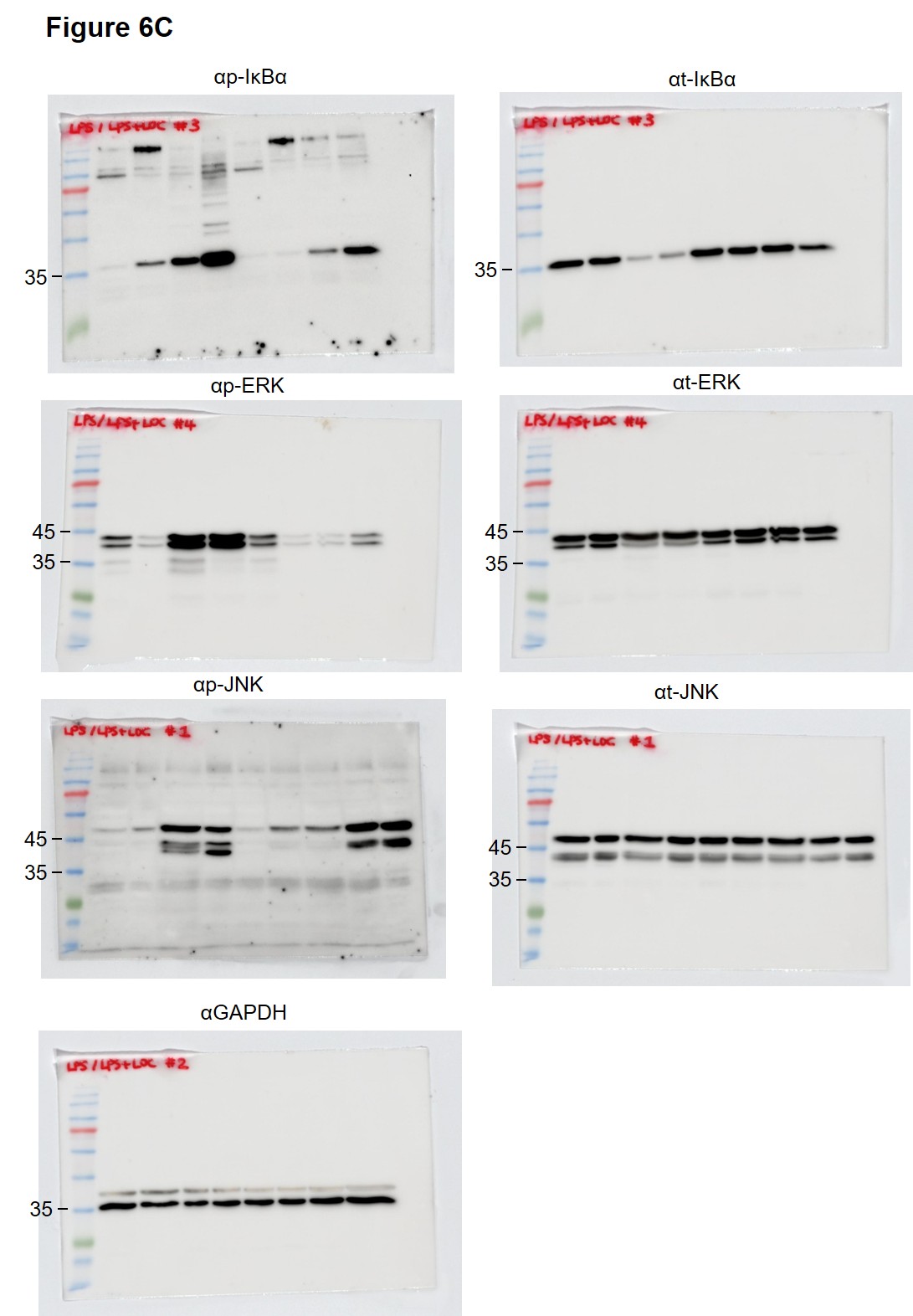

Supplement: Supplementary file 8 — Source data Fig. 6 [file 44321_2026_425_MOESM8_ESM.zip › Figure 6 Source Data/6C/6C.jpg]

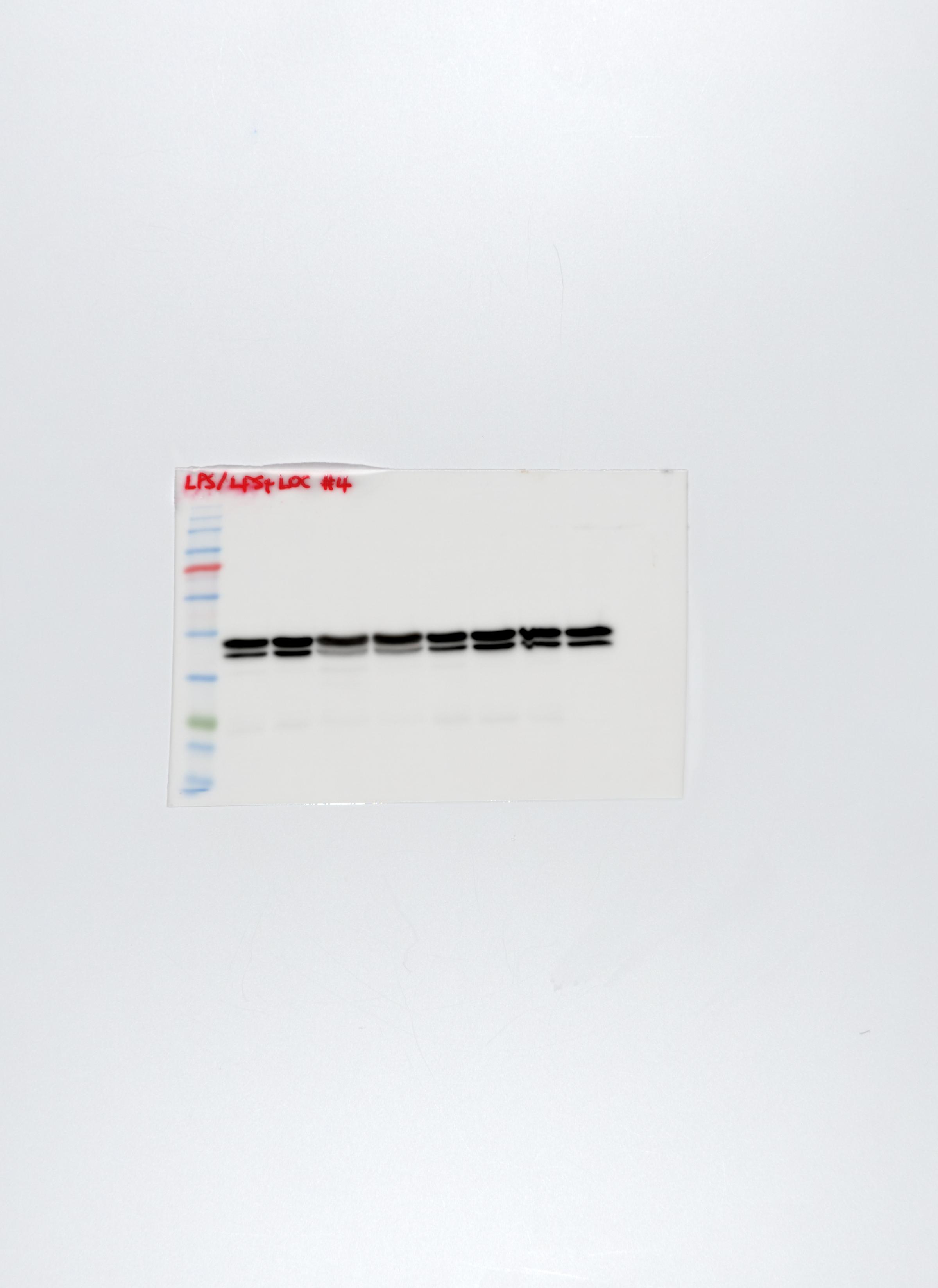

Supplement: Supplementary file 8 — Source data Fig. 6 [file 44321_2026_425_MOESM8_ESM.zip › Figure 6 Source Data/6C/6C_t-ERK.jpg]

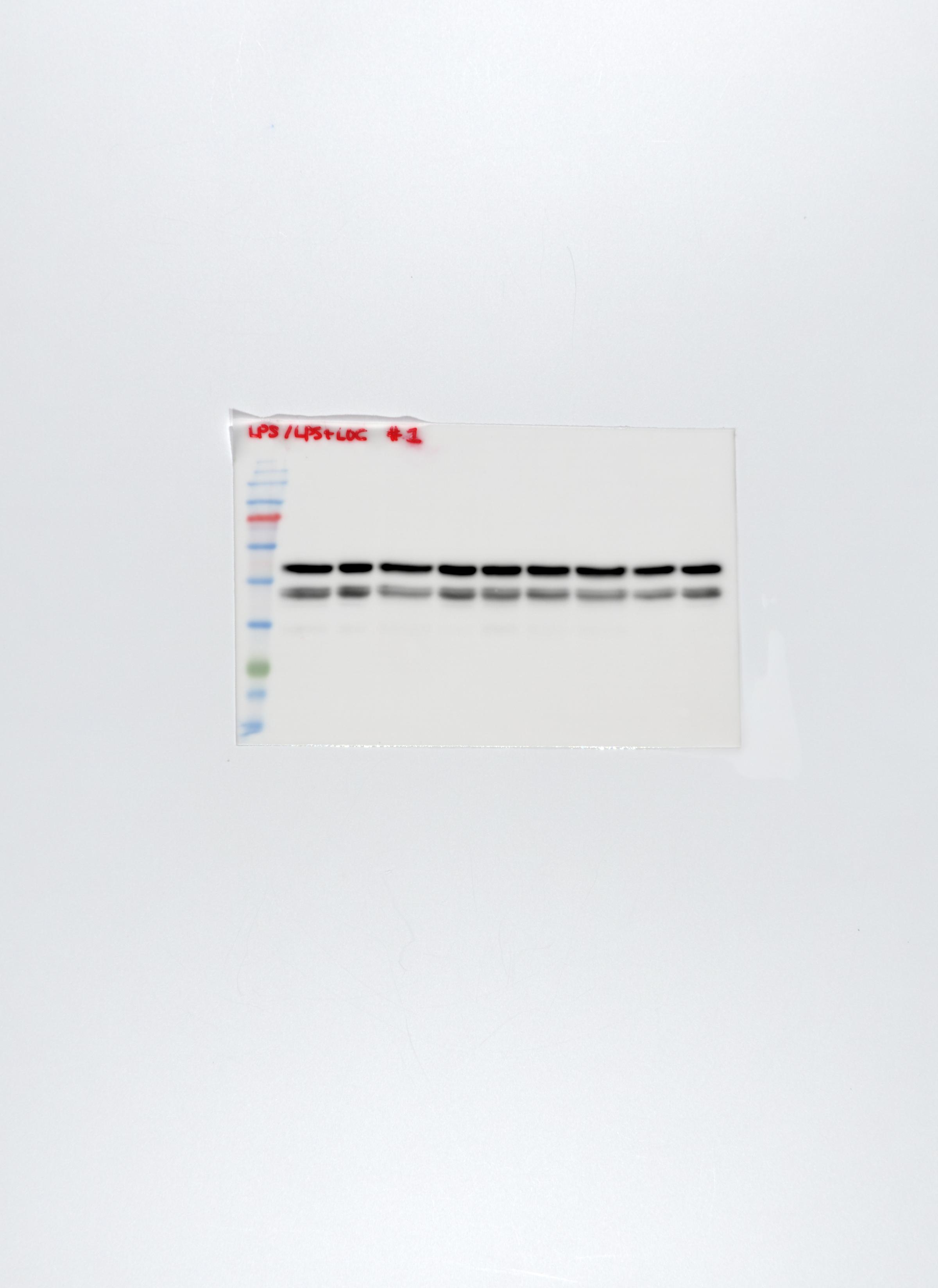

Supplement: Supplementary file 8 — Source data Fig. 6 [file 44321_2026_425_MOESM8_ESM.zip › Figure 6 Source Data/6C/6C_t-JNK.jpg]

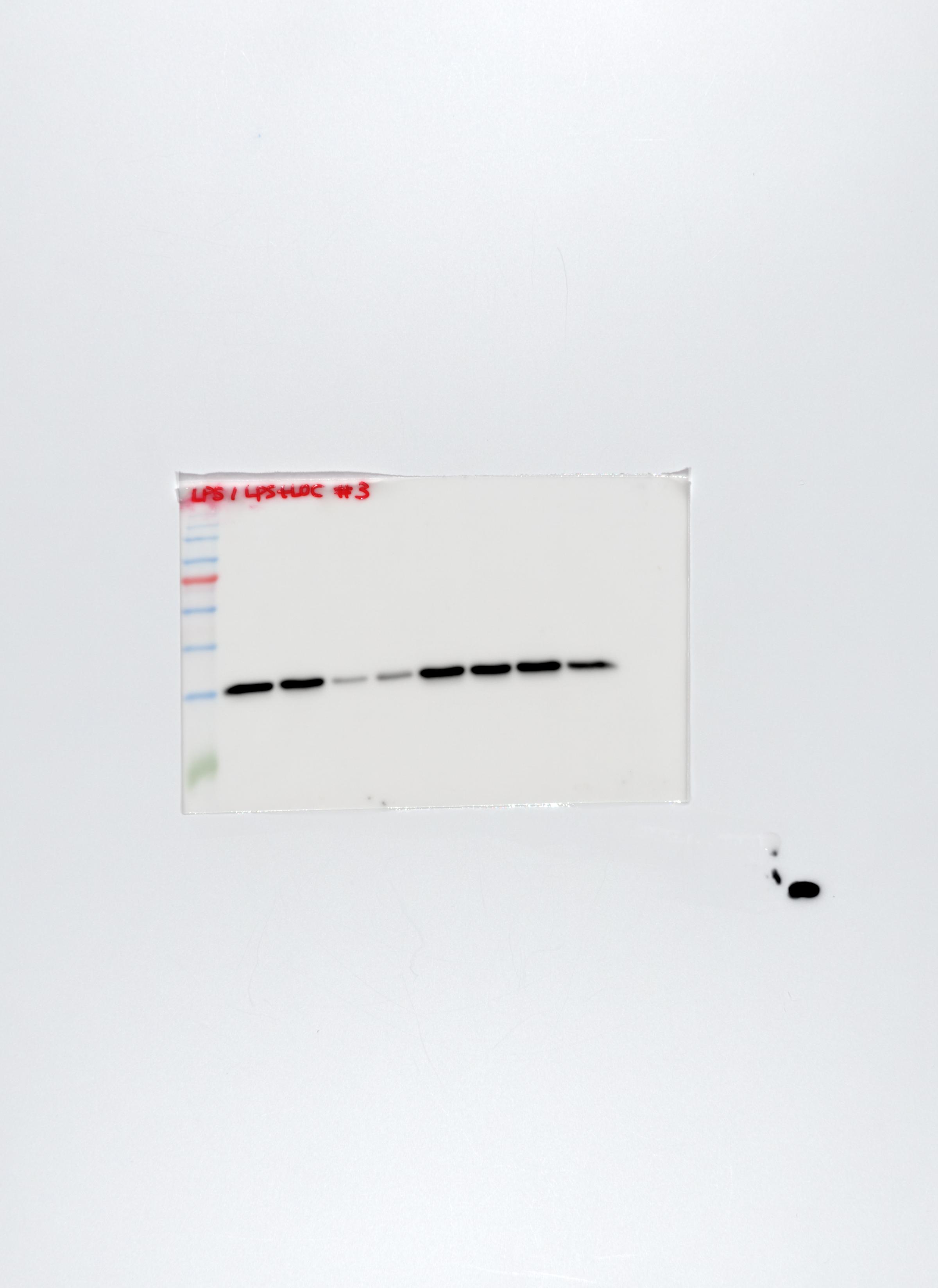

Supplement: Supplementary file 8 — Source data Fig. 6 [file 44321_2026_425_MOESM8_ESM.zip › Figure 6 Source Data/6C/6C_t-IkBa.jpg]

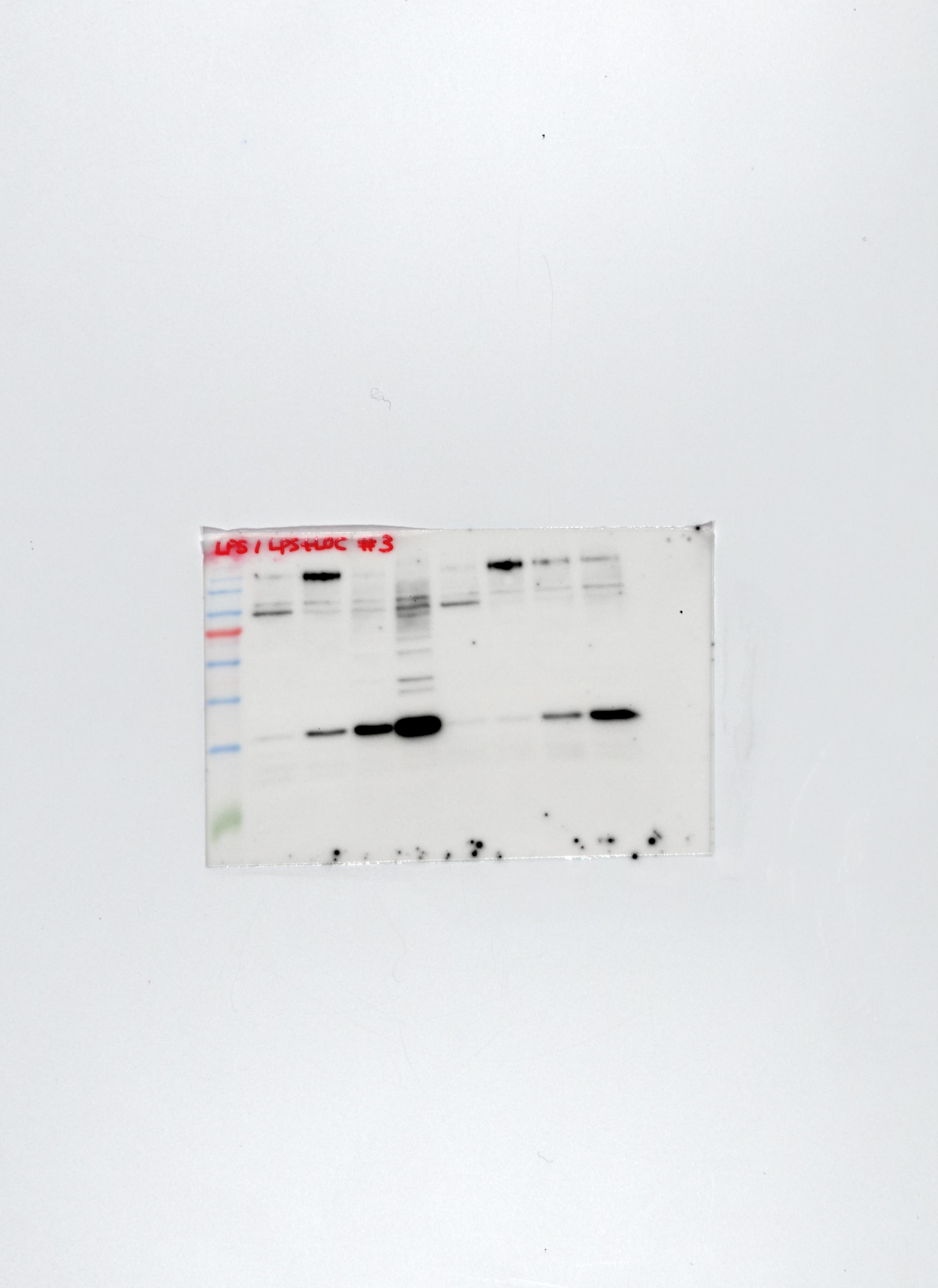

Supplement: Supplementary file 8 — Source data Fig. 6 [file 44321_2026_425_MOESM8_ESM.zip › Figure 6 Source Data/6C/6C_p-IkBa.jpg]

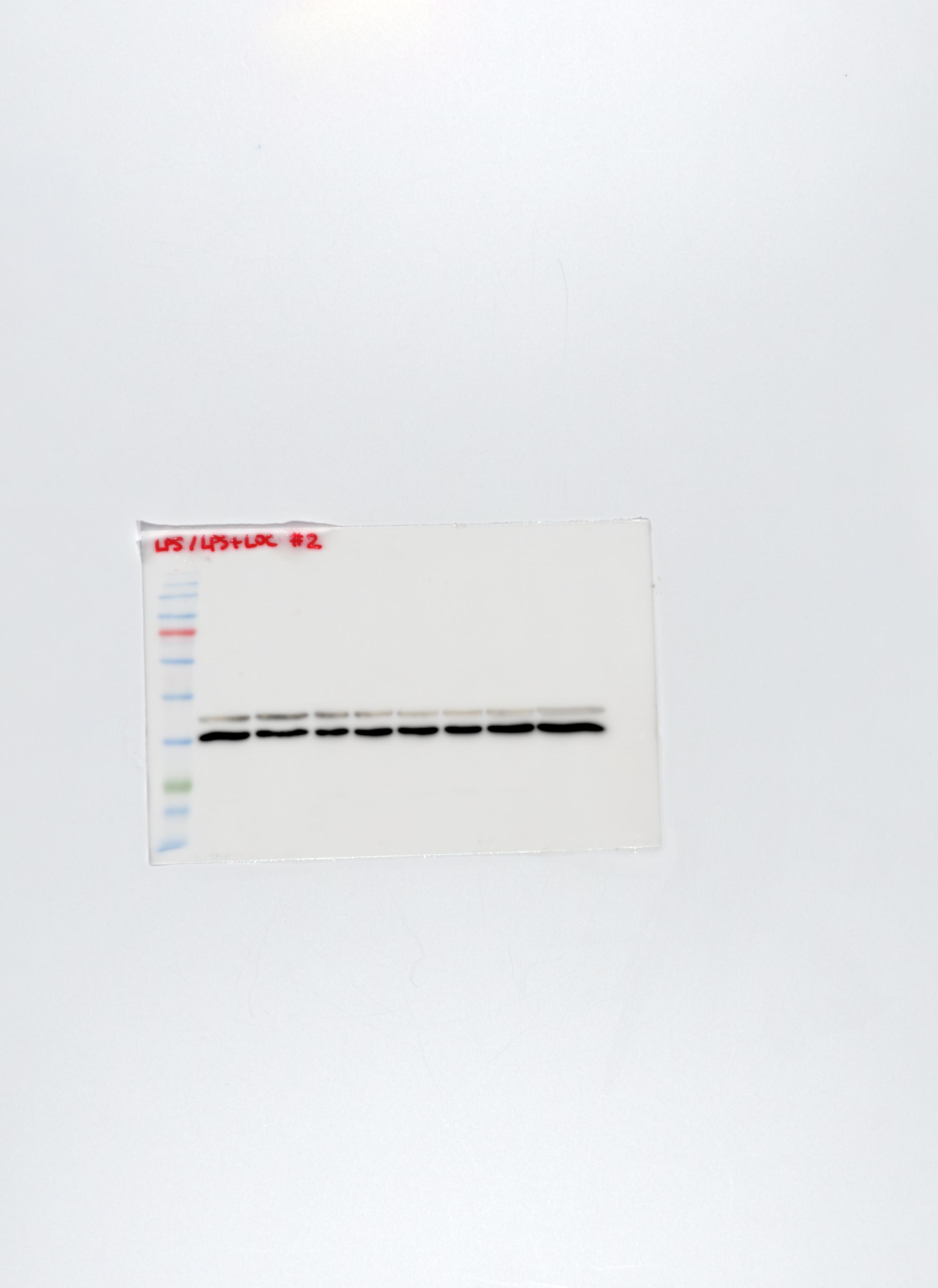

Supplement: Supplementary file 8 — Source data Fig. 6 [file 44321_2026_425_MOESM8_ESM.zip › Figure 6 Source Data/6C/6C_GAPDH.jpg]

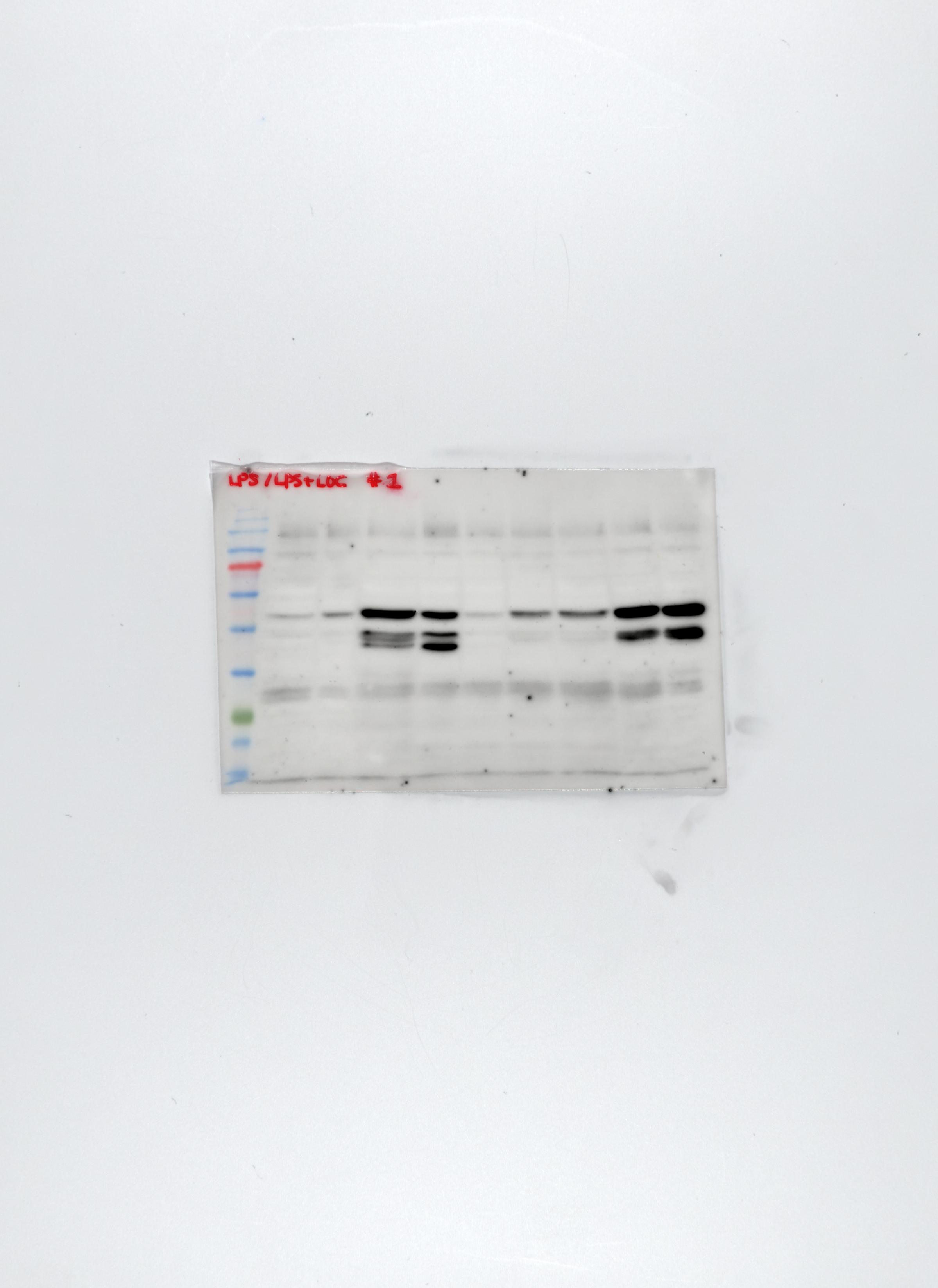

Supplement: Supplementary file 8 — Source data Fig. 6 [file 44321_2026_425_MOESM8_ESM.zip › Figure 6 Source Data/6C/6C_p-JNK.jpg]

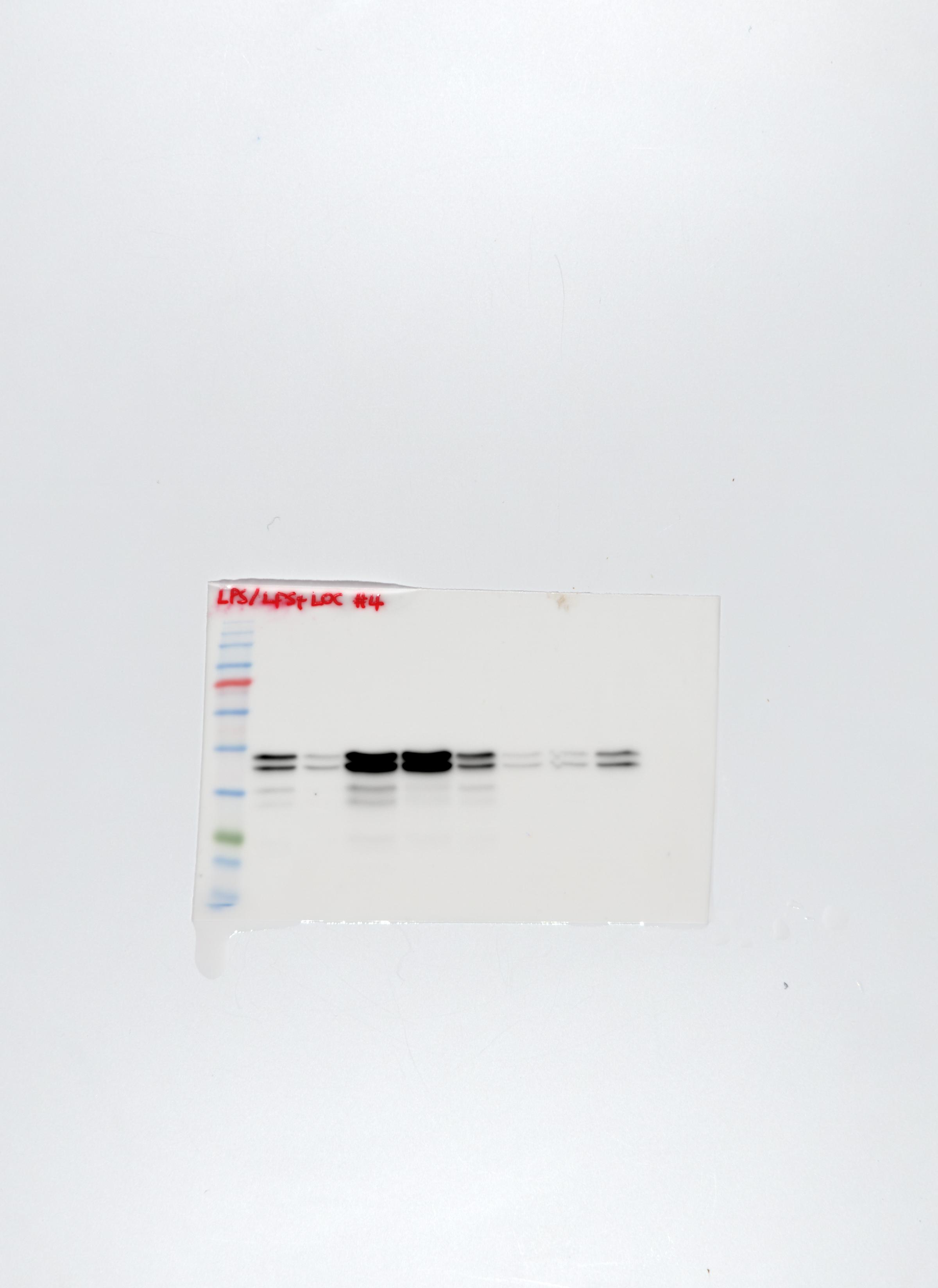

Supplement: Supplementary file 8 — Source data Fig. 6 [file 44321_2026_425_MOESM8_ESM.zip › Figure 6 Source Data/6C/6C_p-ERK.jpg]

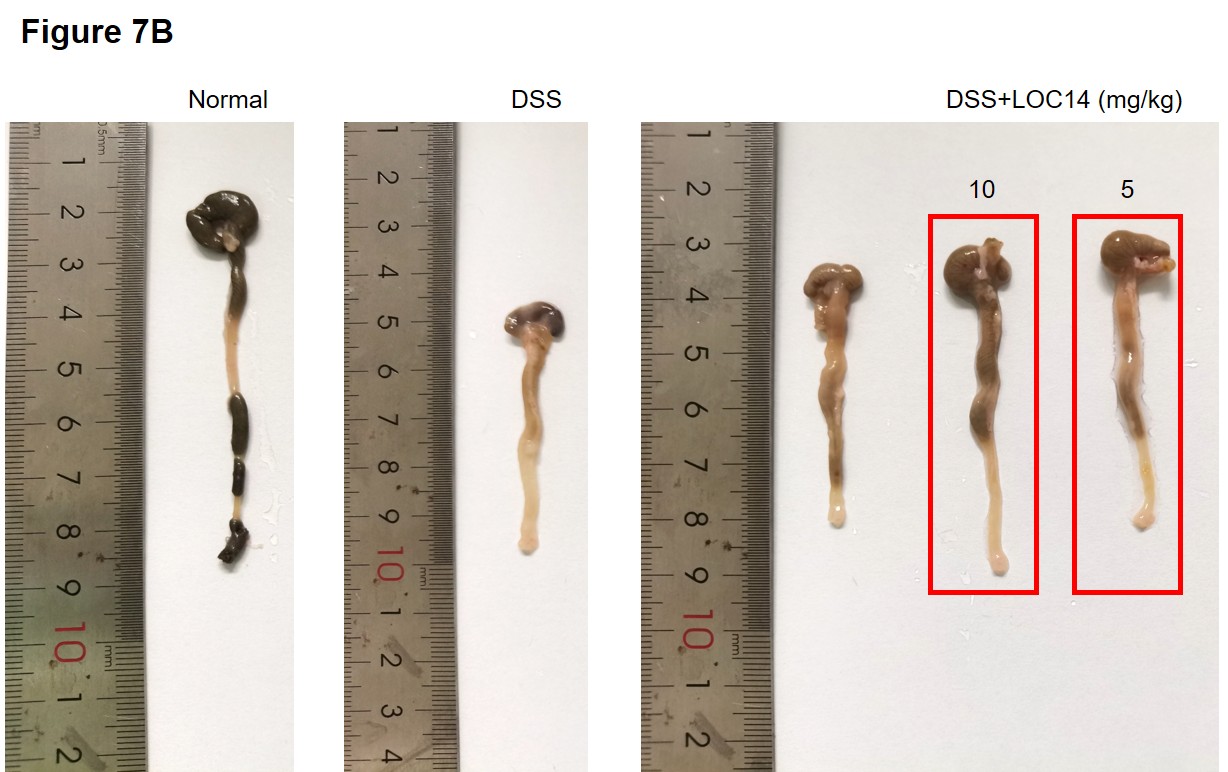

Supplement: Supplementary file 9 — Source data Fig. 7 [file 44321_2026_425_MOESM9_ESM.zip › Figure 7 Source Data/7B/7B.jpg]

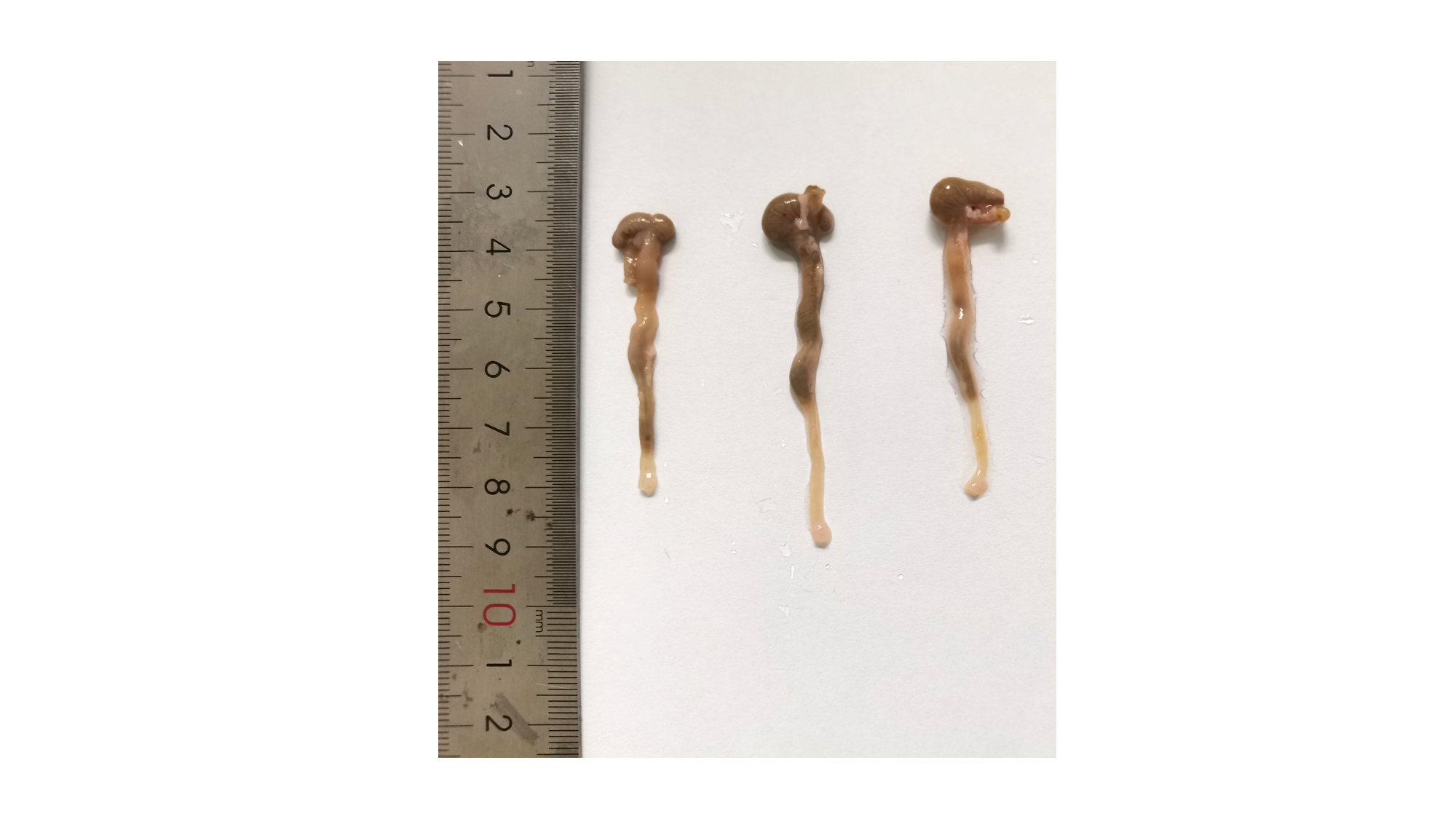

Supplement: Supplementary file 9 — Source data Fig. 7 [file 44321_2026_425_MOESM9_ESM.zip › Figure 7 Source Data/7B/7B_DSSLOC14-5 and DSSLOC14-10.tiff]

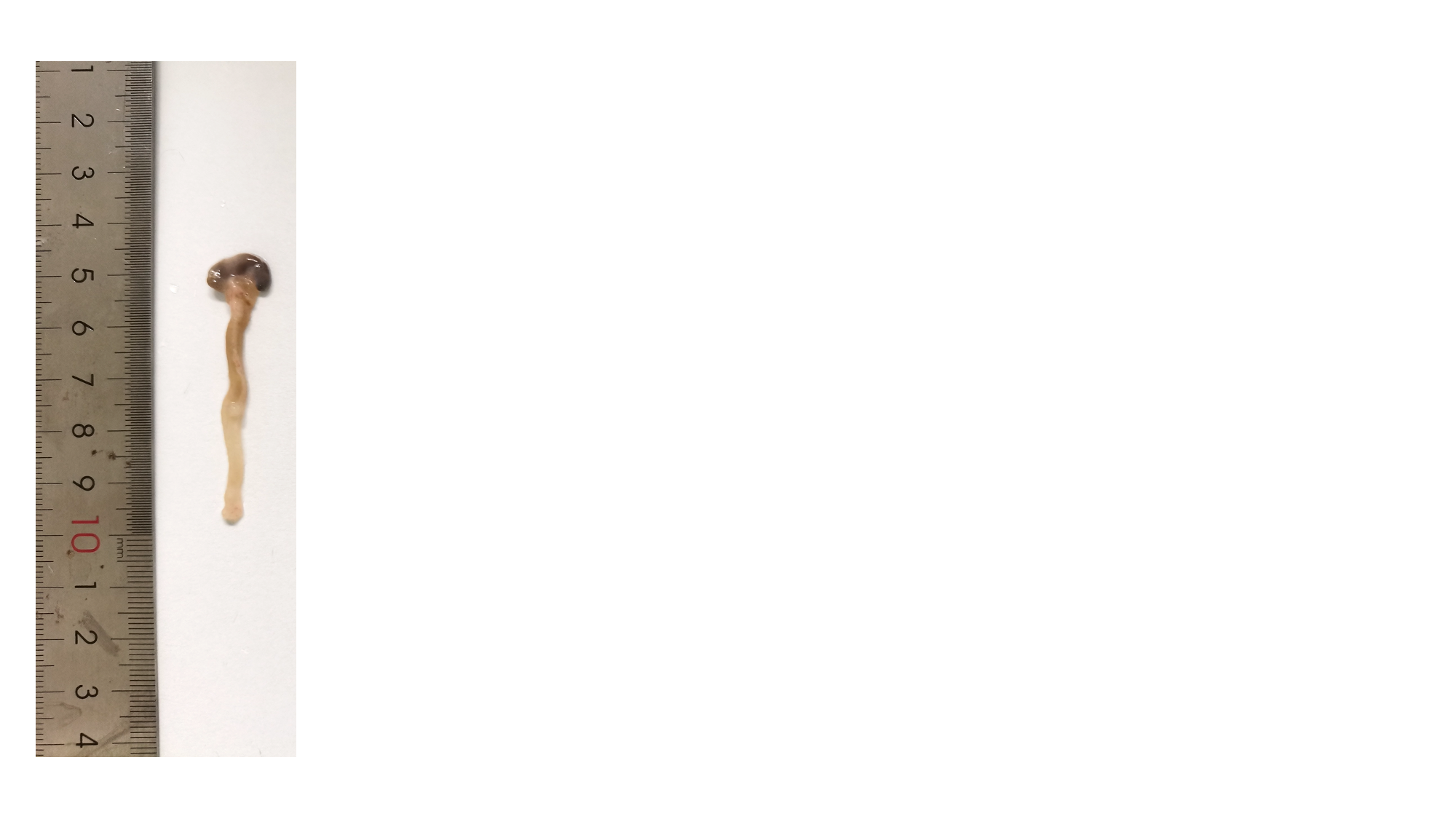

Supplement: Supplementary file 9 — Source data Fig. 7 [file 44321_2026_425_MOESM9_ESM.zip › Figure 7 Source Data/7B/7B_DSS.tiff]

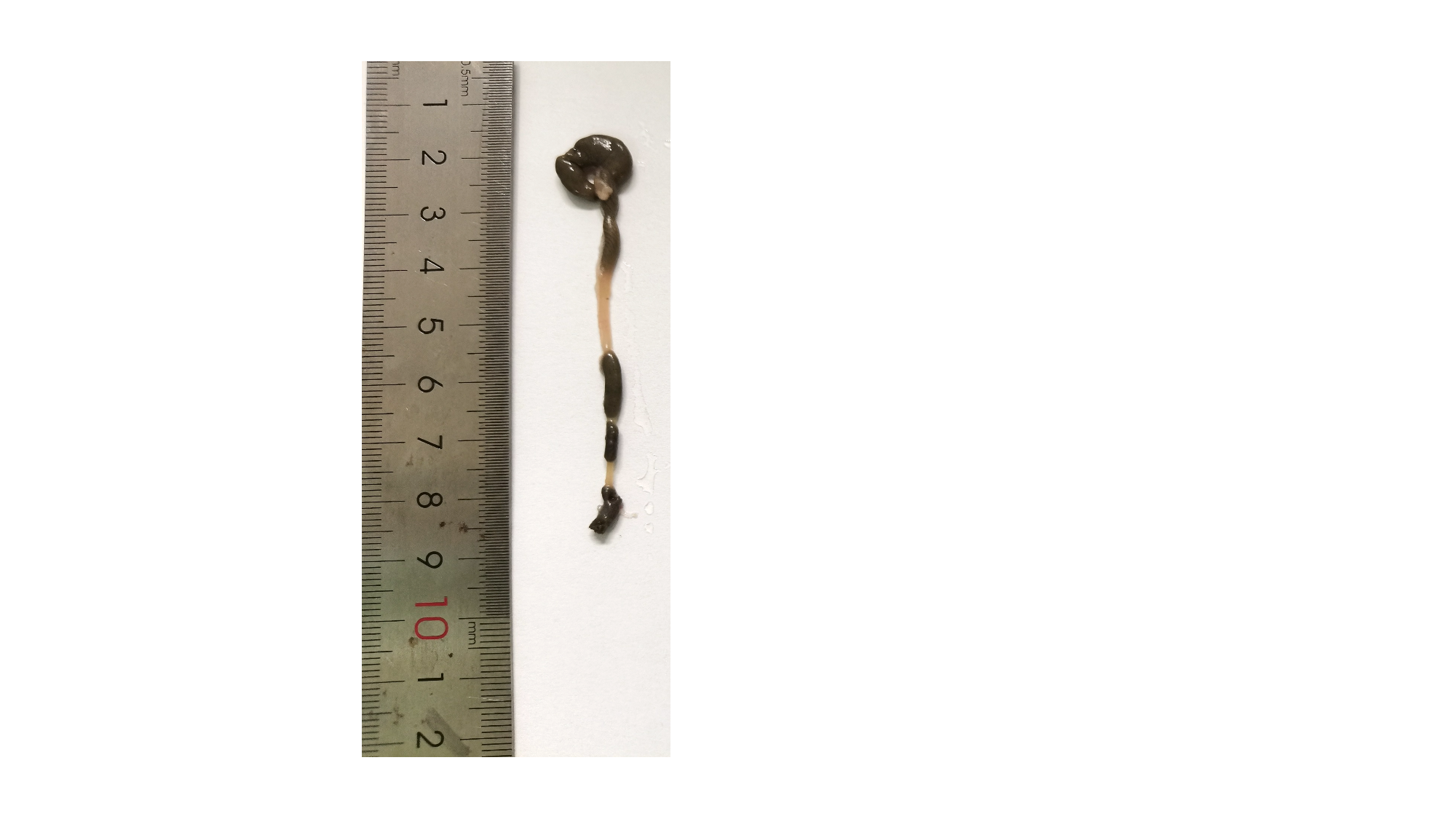

Supplement: Supplementary file 9 — Source data Fig. 7 [file 44321_2026_425_MOESM9_ESM.zip › Figure 7 Source Data/7B/7B_Normal.tiff]

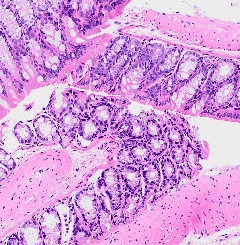

Supplement: Supplementary file 9 — Source data Fig. 7 [file 44321_2026_425_MOESM9_ESM.zip › Figure 7 Source Data/7E/7E_Normal_Middle.jpeg]

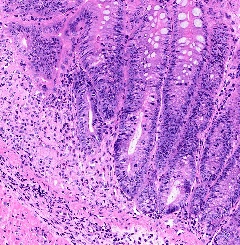

Supplement: Supplementary file 9 — Source data Fig. 7 [file 44321_2026_425_MOESM9_ESM.zip › Figure 7 Source Data/7E/7E_DSS_Middle.jpeg]

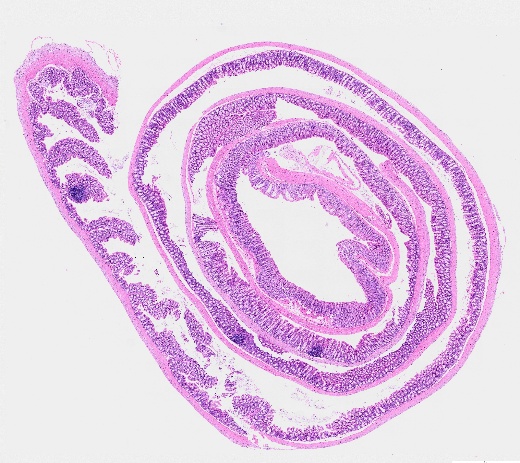

Supplement: Supplementary file 9 — Source data Fig. 7 [file 44321_2026_425_MOESM9_ESM.zip › Figure 7 Source Data/7E/7E_DSS+LOC14(5mgkg).jpeg]

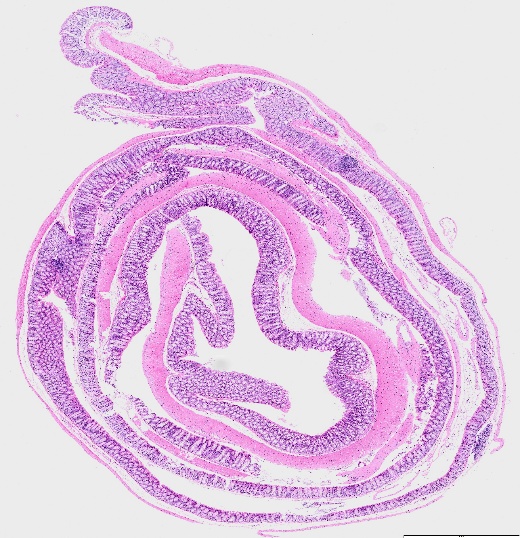

Supplement: Supplementary file 9 — Source data Fig. 7 [file 44321_2026_425_MOESM9_ESM.zip › Figure 7 Source Data/7E/7E_Normal.jpeg]

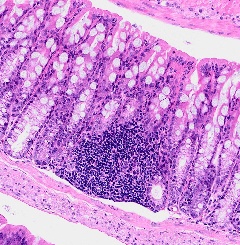

Supplement: Supplementary file 9 — Source data Fig. 7 [file 44321_2026_425_MOESM9_ESM.zip › Figure 7 Source Data/7E/7E_DSS+LOC14(5mgkg)_Middle.jpeg]

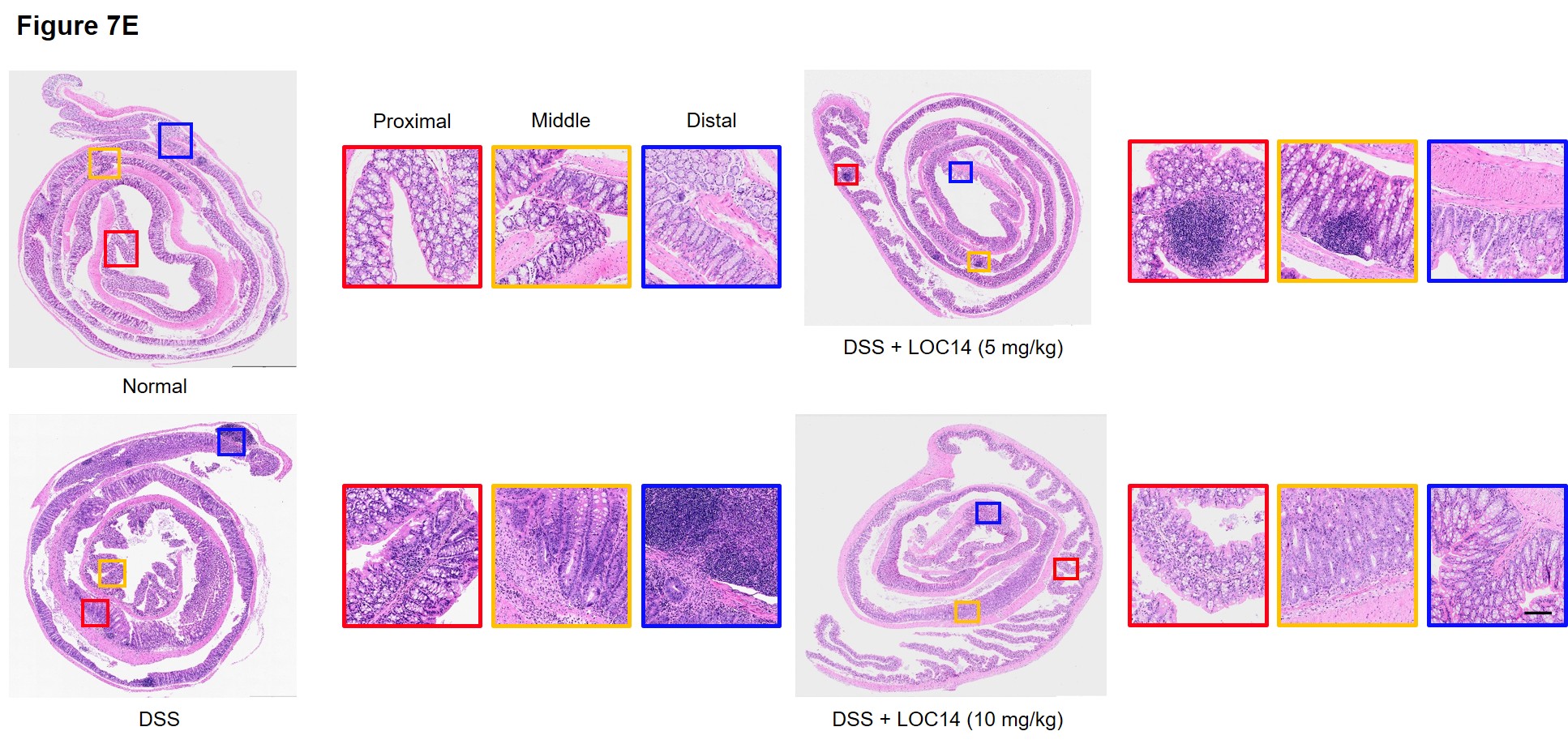

Supplement: Supplementary file 9 — Source data Fig. 7 [file 44321_2026_425_MOESM9_ESM.zip › Figure 7 Source Data/7E/7E.jpg]

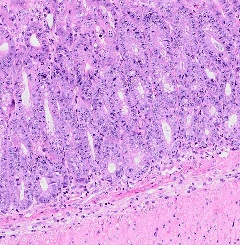

Supplement: Supplementary file 9 — Source data Fig. 7 [file 44321_2026_425_MOESM9_ESM.zip › Figure 7 Source Data/7E/7E_DSS+LOC14(10mgkg)_Middle.jpeg]

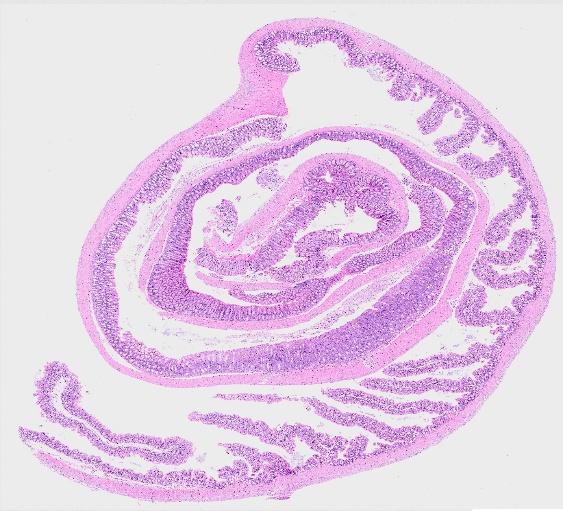

Supplement: Supplementary file 9 — Source data Fig. 7 [file 44321_2026_425_MOESM9_ESM.zip › Figure 7 Source Data/7E/7E_DSS+LOC14(10mgkg).jpeg]

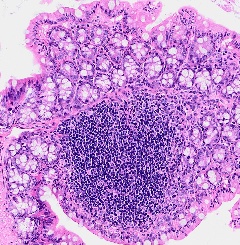

Supplement: Supplementary file 9 — Source data Fig. 7 [file 44321_2026_425_MOESM9_ESM.zip › Figure 7 Source Data/7E/7E_DSS+LOC14(5mgkg)_Proximal.jpeg]

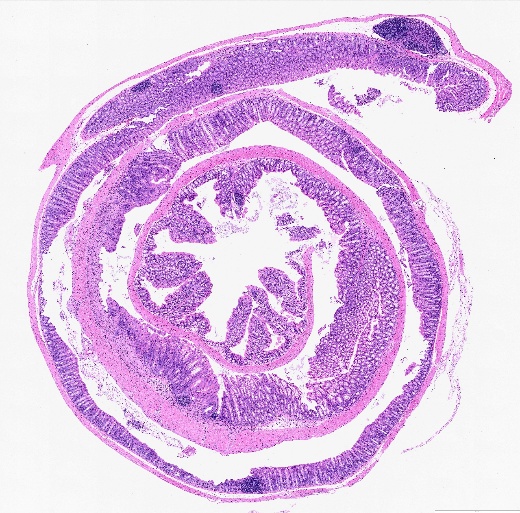

Supplement: Supplementary file 9 — Source data Fig. 7 [file 44321_2026_425_MOESM9_ESM.zip › Figure 7 Source Data/7E/7E_DSS.jpeg]

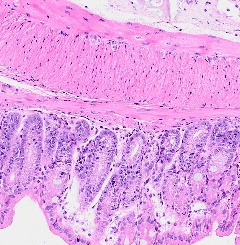

Supplement: Supplementary file 9 — Source data Fig. 7 [file 44321_2026_425_MOESM9_ESM.zip › Figure 7 Source Data/7E/7E_DSS+LOC14(5mgkg)_Distal.jpeg]

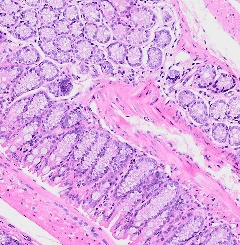

Supplement: Supplementary file 9 — Source data Fig. 7 [file 44321_2026_425_MOESM9_ESM.zip › Figure 7 Source Data/7E/7E_Normal_Distal.jpeg]

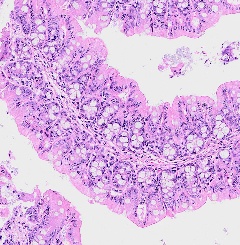

Supplement: Supplementary file 9 — Source data Fig. 7 [file 44321_2026_425_MOESM9_ESM.zip › Figure 7 Source Data/7E/7E_DSS+LOC14(10mgkg)_Proximal.jpeg]

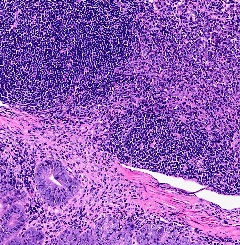

Supplement: Supplementary file 9 — Source data Fig. 7 [file 44321_2026_425_MOESM9_ESM.zip › Figure 7 Source Data/7E/7E_DSS_Distal.jpeg]

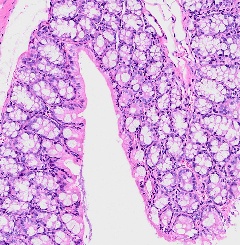

Supplement: Supplementary file 9 — Source data Fig. 7 [file 44321_2026_425_MOESM9_ESM.zip › Figure 7 Source Data/7E/7E_Normal_Proximal.jpeg]

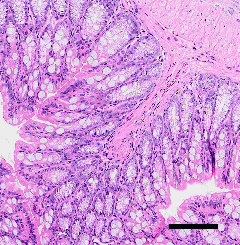

Supplement: Supplementary file 9 — Source data Fig. 7 [file 44321_2026_425_MOESM9_ESM.zip › Figure 7 Source Data/7E/7E_DSS+LOC14(10mgkg)_Distal.tiff]

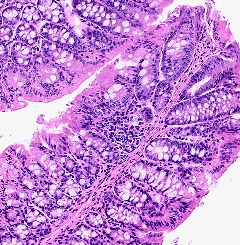

Supplement: Supplementary file 9 — Source data Fig. 7 [file 44321_2026_425_MOESM9_ESM.zip › Figure 7 Source Data/7E/7E_DSS_Proximal.jpeg]
