# Supplementary figures and images for: Pharmacological targeting of the NLRP3 LRR domain with isothiazolinones overcomes CRID3-resistant inflammation (part 2 of 2)
Source: EMBO Mol Med. 2026 Apr 17;18(6):2124–51. doi: 10.1038/s44321-026-00425-5 (PMC13269794; doi:10.1038/s44321-026-00425-5)

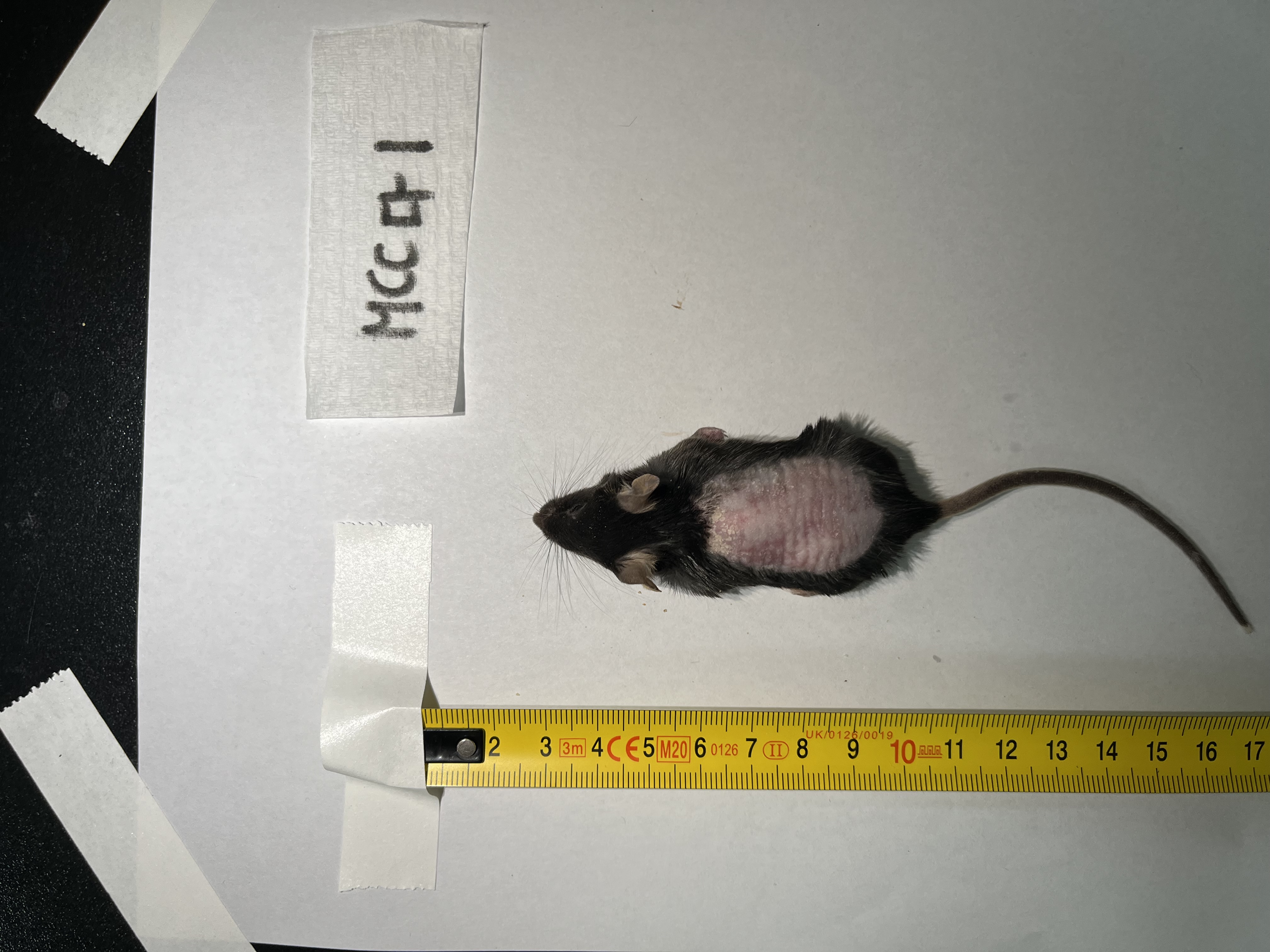

Supplement: Supplementary file 10 — Source data Fig. 8 [file 44321_2026_425_MOESM10_ESM.zip › Figure 8 Source Data/8A/8A_IMQ+CRID3.JPG]

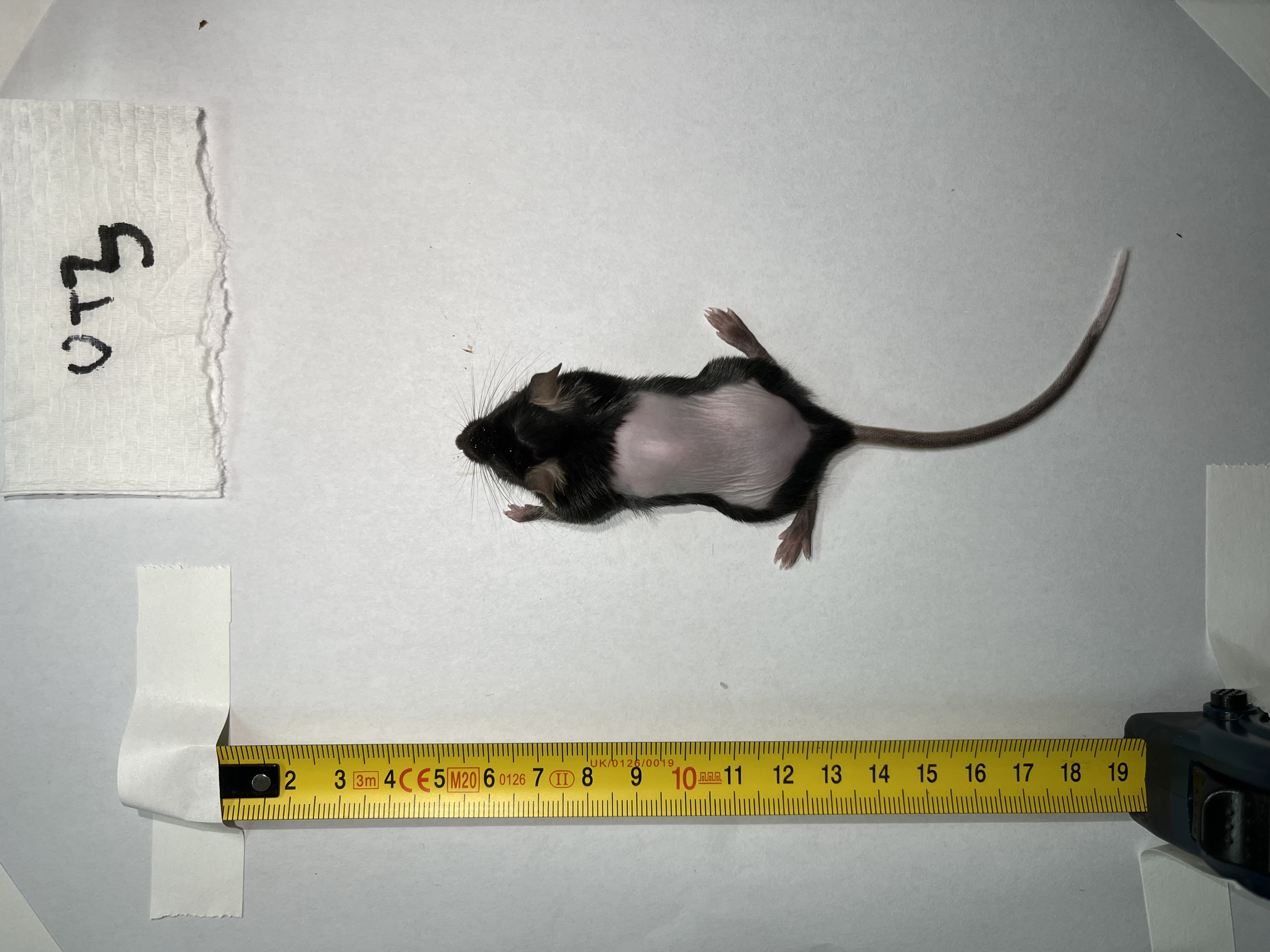

Supplement: Supplementary file 10 — Source data Fig. 8 [file 44321_2026_425_MOESM10_ESM.zip › Figure 8 Source Data/8A/8A_Normal.JPG]

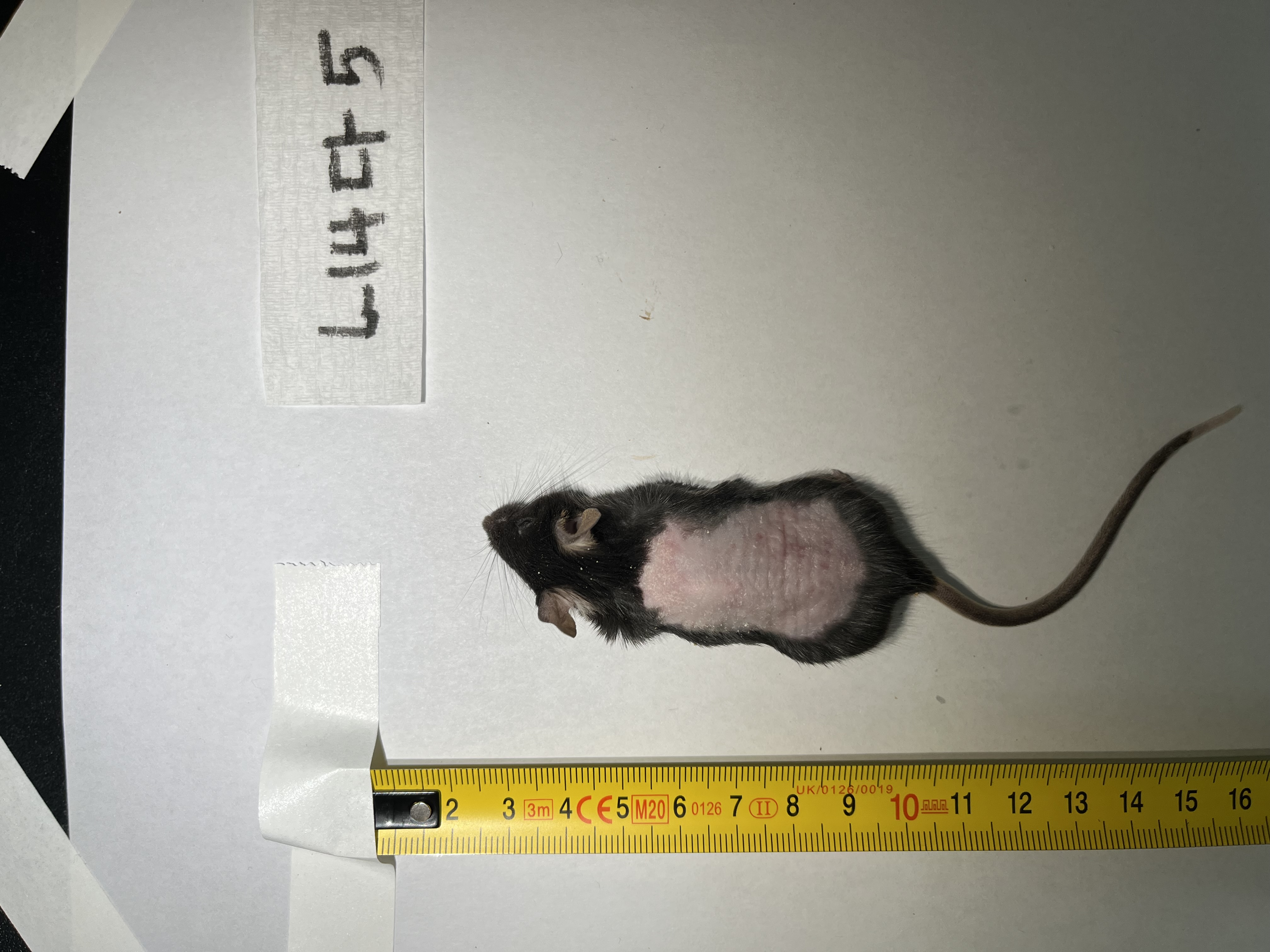

Supplement: Supplementary file 10 — Source data Fig. 8 [file 44321_2026_425_MOESM10_ESM.zip › Figure 8 Source Data/8A/8A_IMQ+LOC14.JPG]

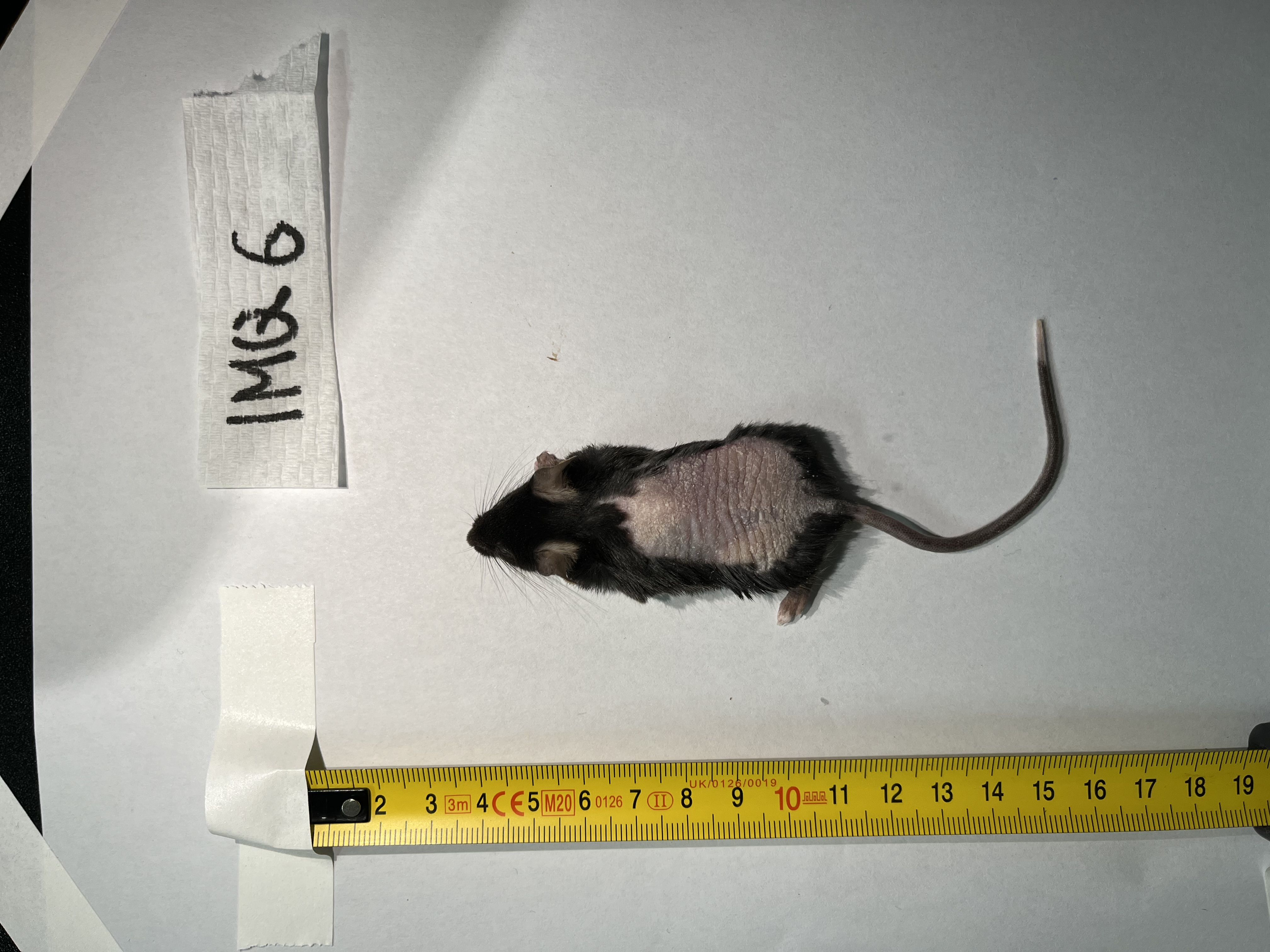

Supplement: Supplementary file 10 — Source data Fig. 8 [file 44321_2026_425_MOESM10_ESM.zip › Figure 8 Source Data/8A/8A_IMQ.JPG]

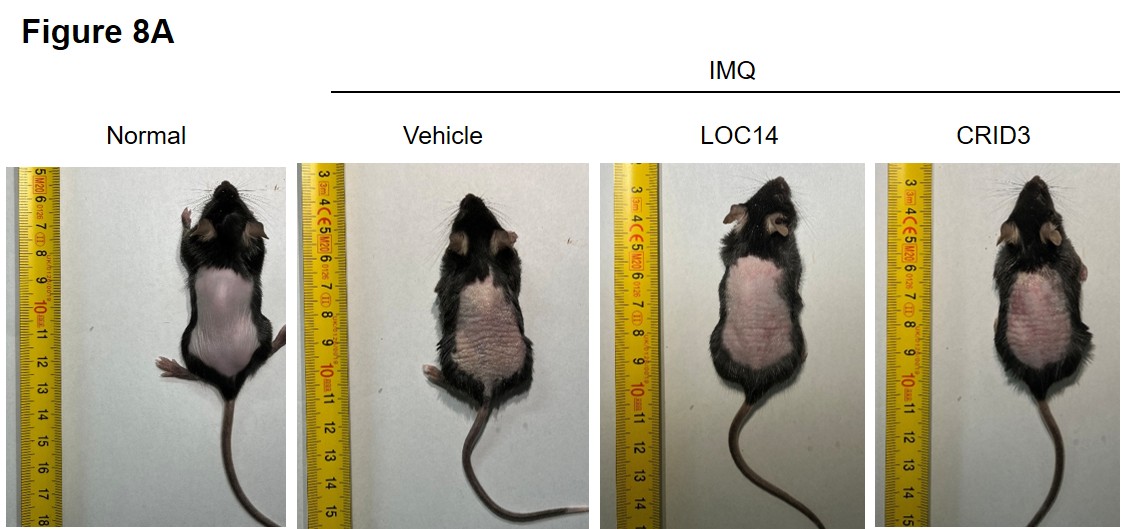

Supplement: Supplementary file 10 — Source data Fig. 8 [file 44321_2026_425_MOESM10_ESM.zip › Figure 8 Source Data/8A/8A.jpg]

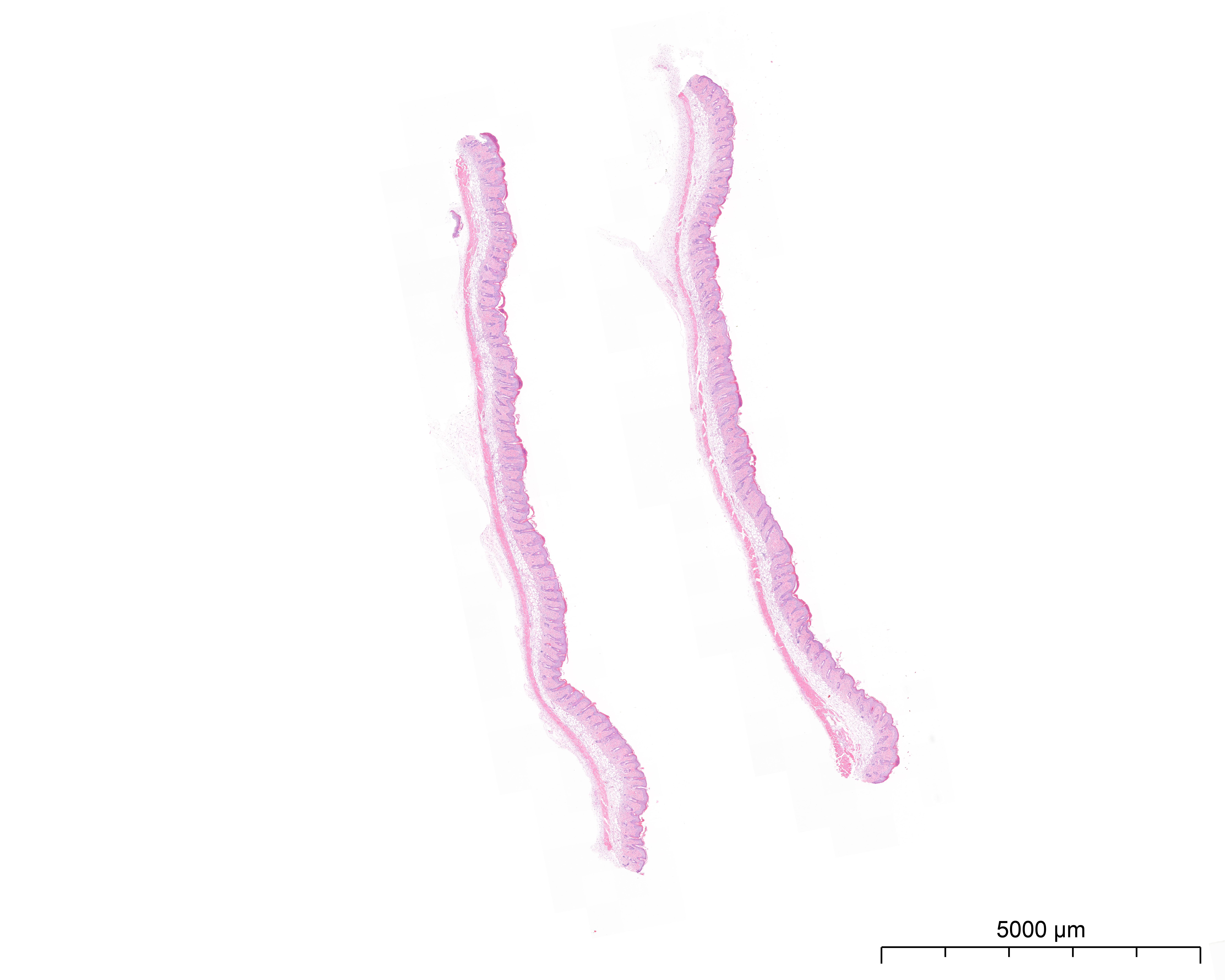

Supplement: Supplementary file 10 — Source data Fig. 8 [file 44321_2026_425_MOESM10_ESM.zip › Figure 8 Source Data/8C/8C_IMQ+CRID3__H&E_1.0x 600ppi.jpg]

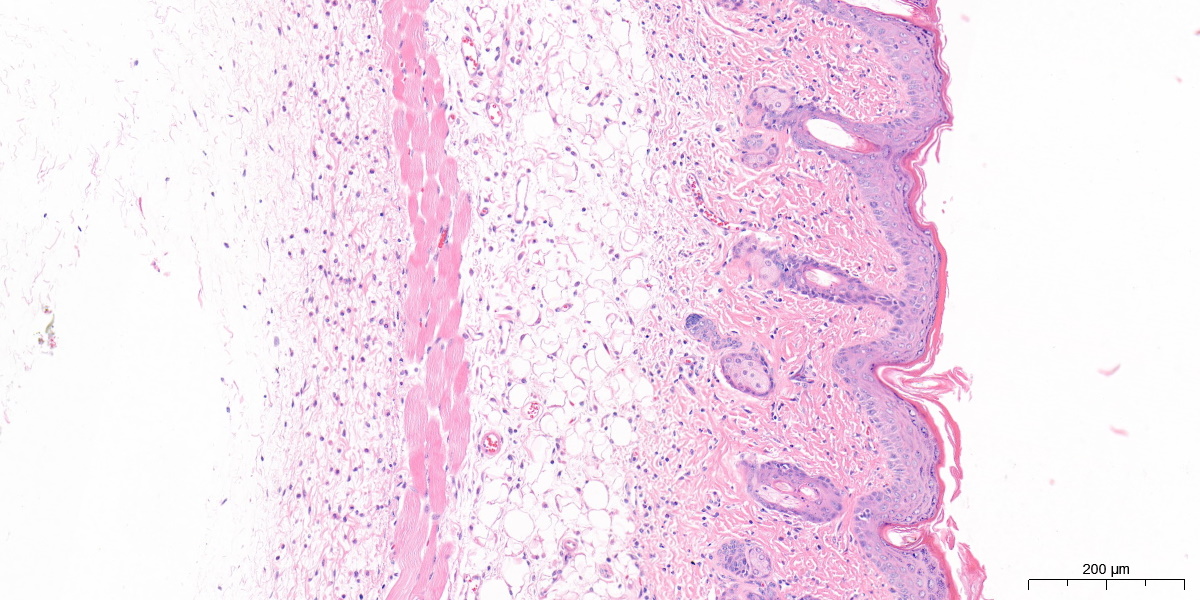

Supplement: Supplementary file 10 — Source data Fig. 8 [file 44321_2026_425_MOESM10_ESM.zip › Figure 8 Source Data/8C/8C_IMQ+CRID3_12.5x.jpg]

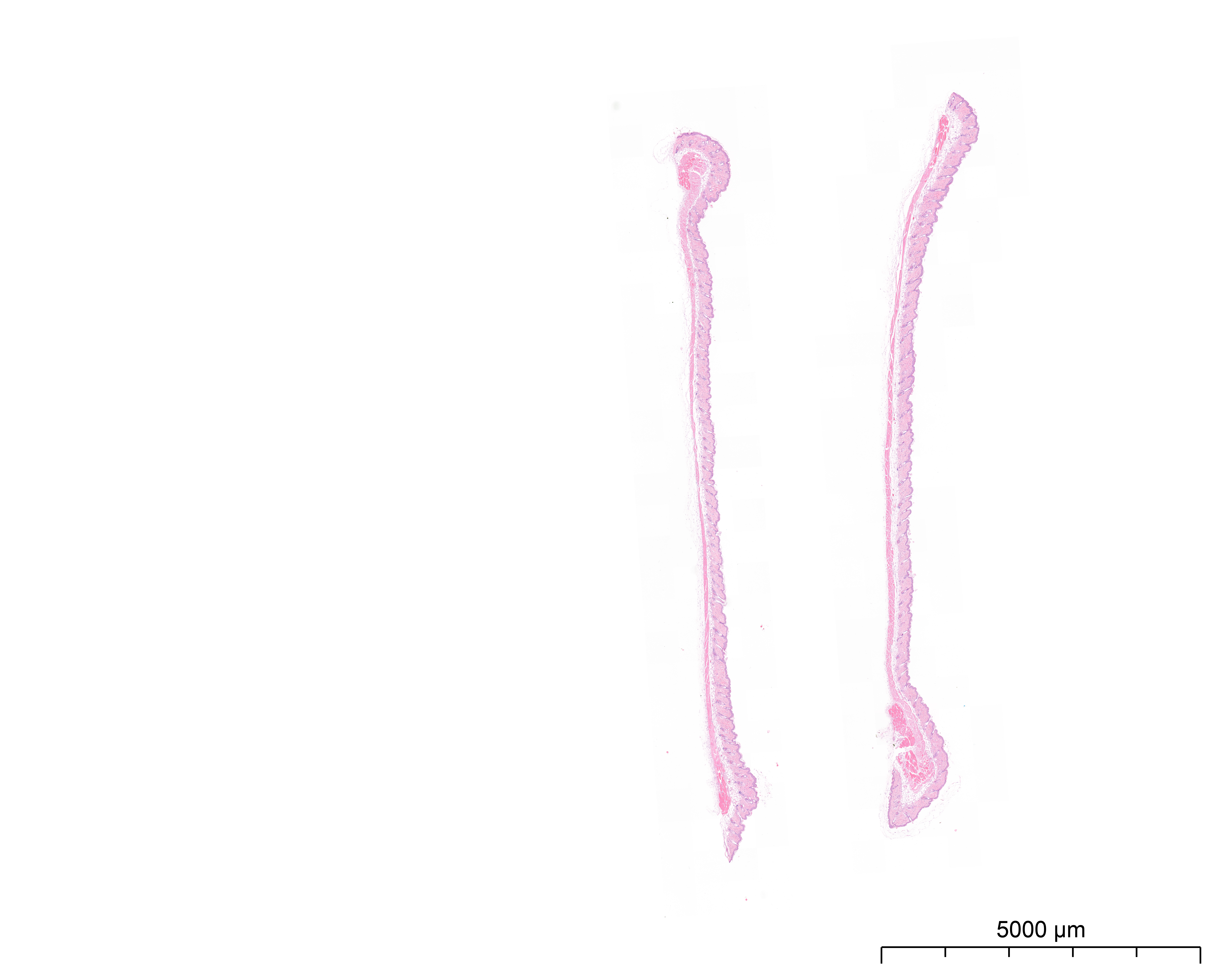

Supplement: Supplementary file 10 — Source data Fig. 8 [file 44321_2026_425_MOESM10_ESM.zip › Figure 8 Source Data/8C/8C_Normal__H&E_1.0x 600ppi.jpg]

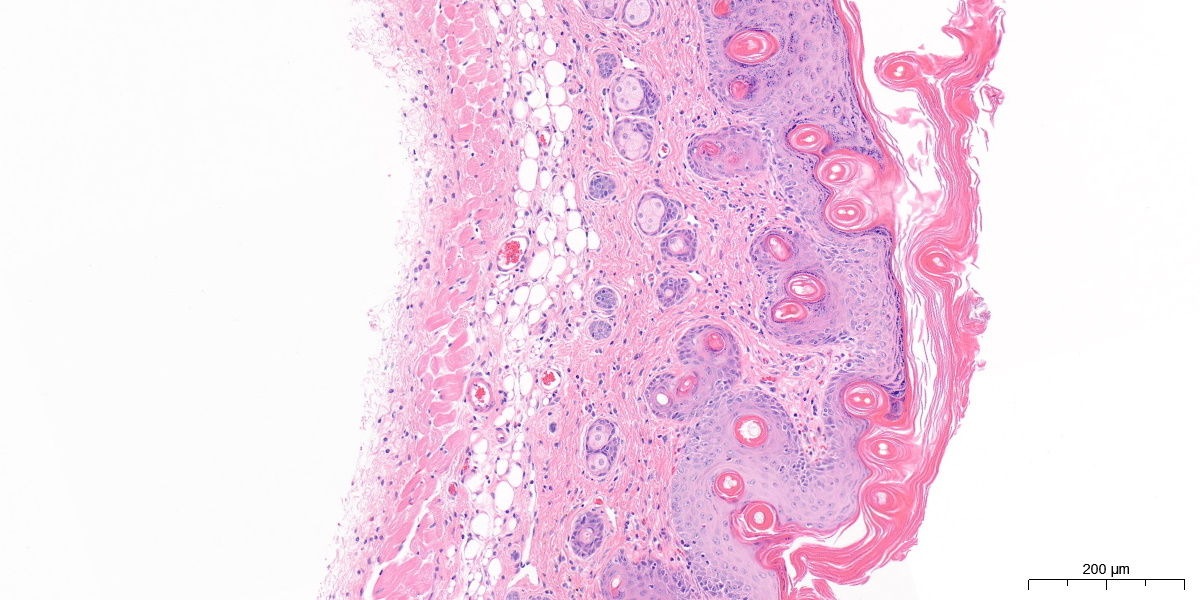

Supplement: Supplementary file 10 — Source data Fig. 8 [file 44321_2026_425_MOESM10_ESM.zip › Figure 8 Source Data/8C/8C_IMQ_12.5x.jpg]

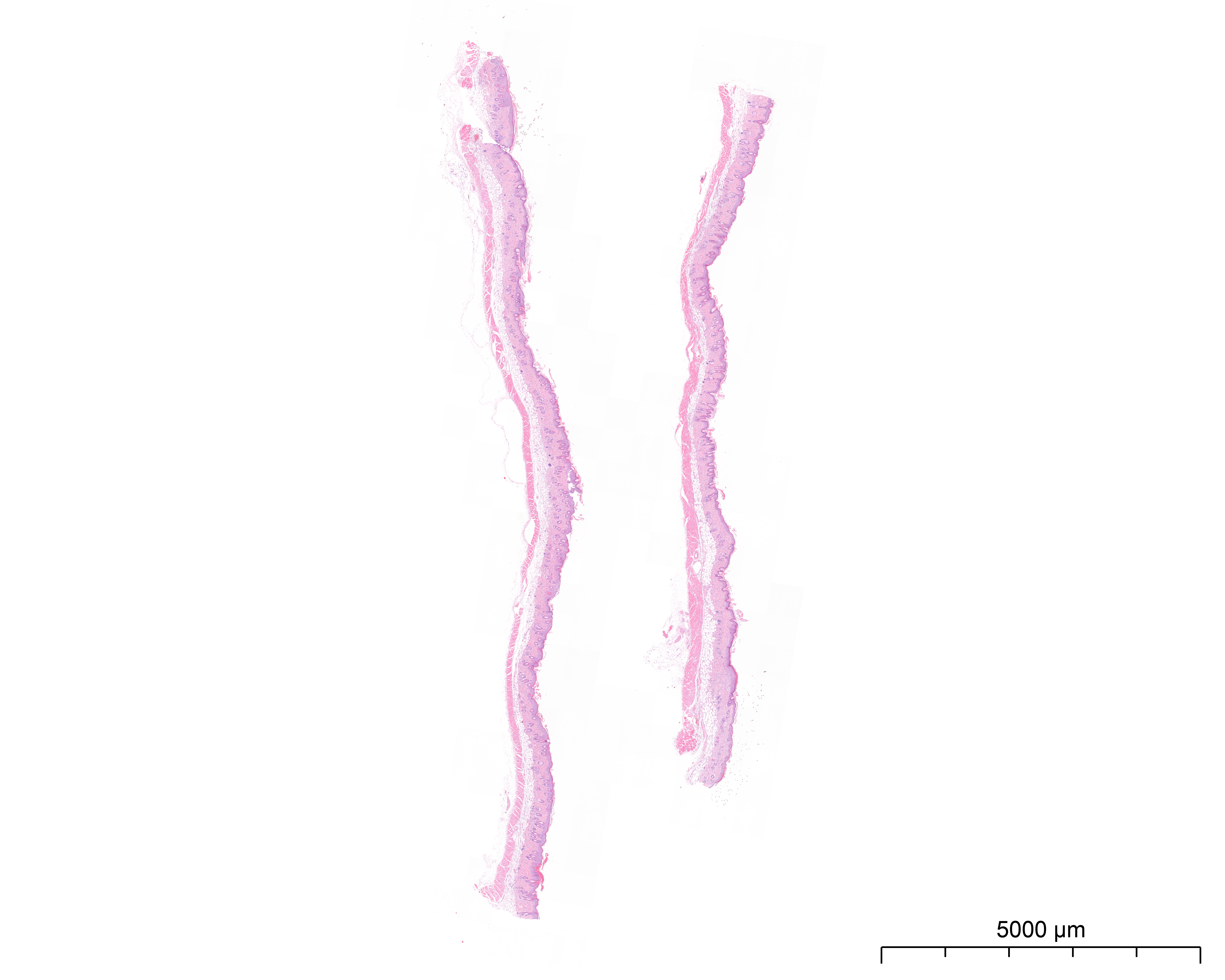

Supplement: Supplementary file 10 — Source data Fig. 8 [file 44321_2026_425_MOESM10_ESM.zip › Figure 8 Source Data/8C/8C_IMQ+LOC14__H&E_1.0x 600ppi.jpg]

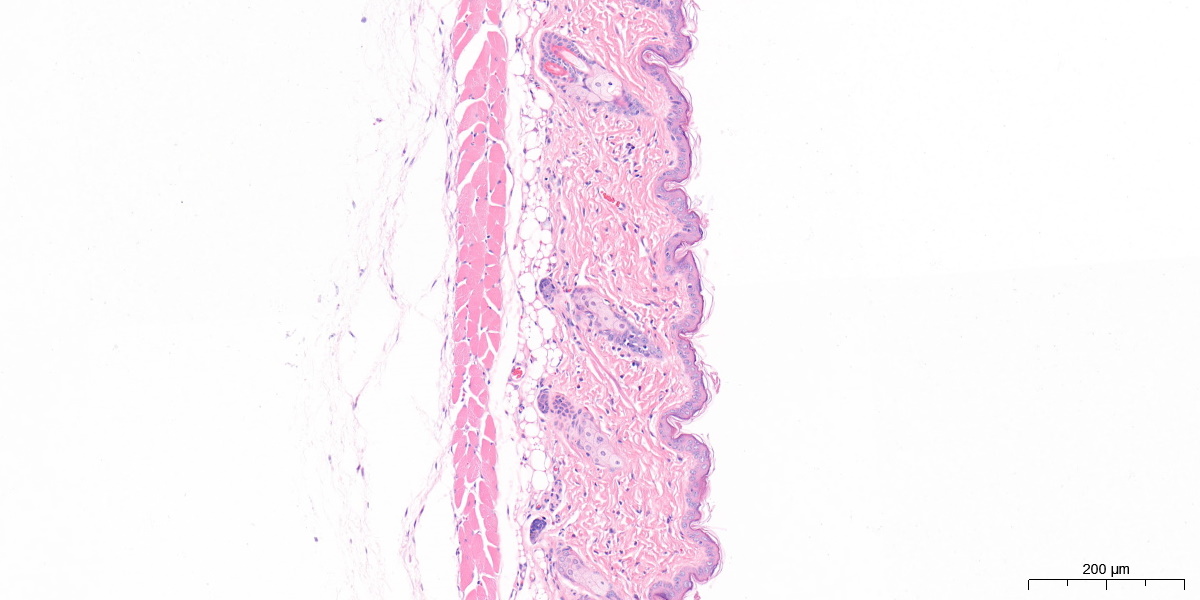

Supplement: Supplementary file 10 — Source data Fig. 8 [file 44321_2026_425_MOESM10_ESM.zip › Figure 8 Source Data/8C/8C_Normal_12.5x.jpg]

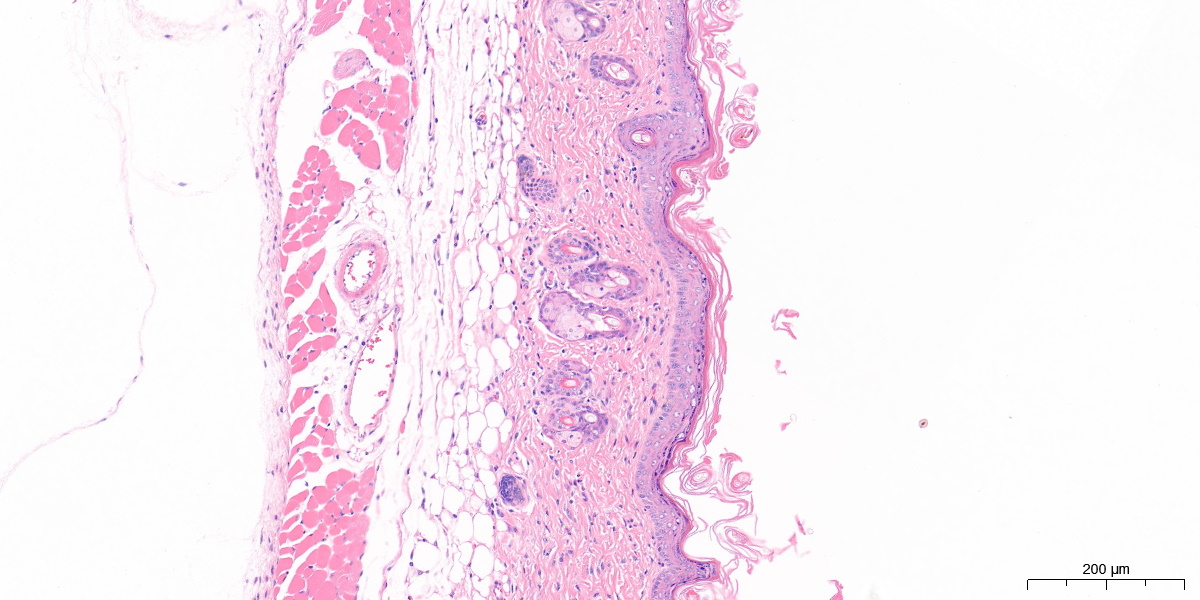

Supplement: Supplementary file 10 — Source data Fig. 8 [file 44321_2026_425_MOESM10_ESM.zip › Figure 8 Source Data/8C/8C_IMQ+LOC14_12.5x.jpg]

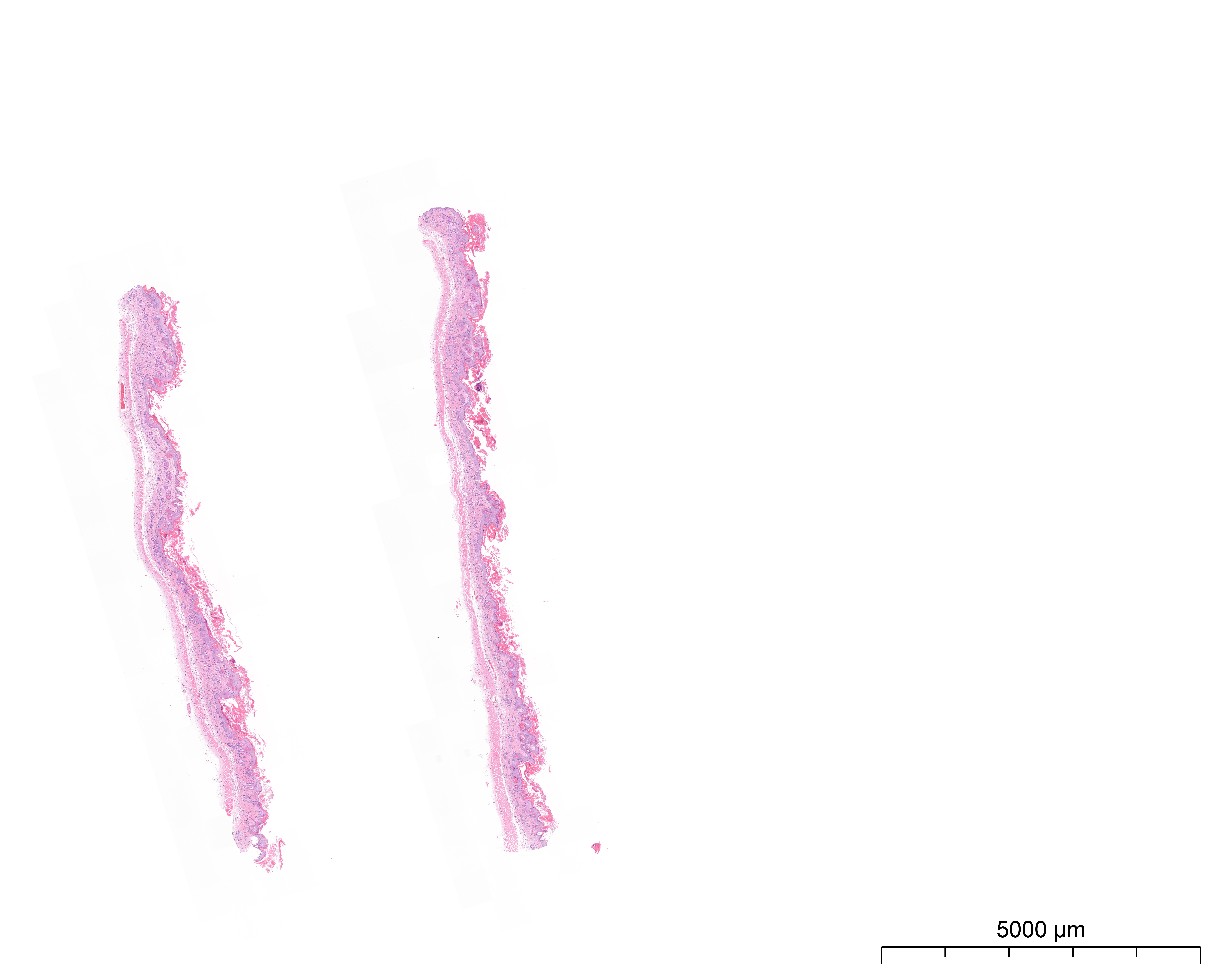

Supplement: Supplementary file 10 — Source data Fig. 8 [file 44321_2026_425_MOESM10_ESM.zip › Figure 8 Source Data/8C/8C_IMQ__H&E_1.0x 600ppi.jpg]

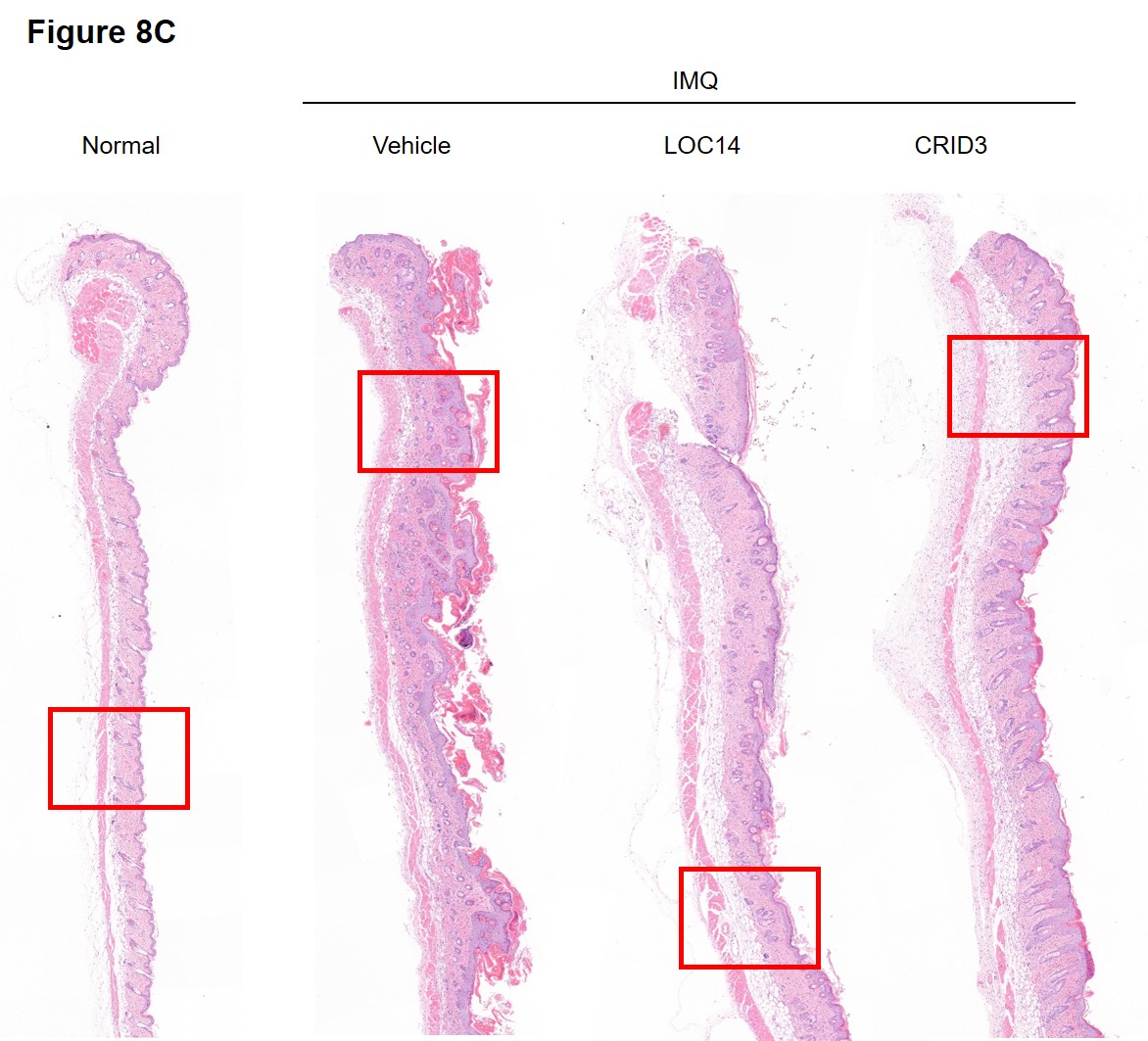

Supplement: Supplementary file 10 — Source data Fig. 8 [file 44321_2026_425_MOESM10_ESM.zip › Figure 8 Source Data/8C/8C.jpg]

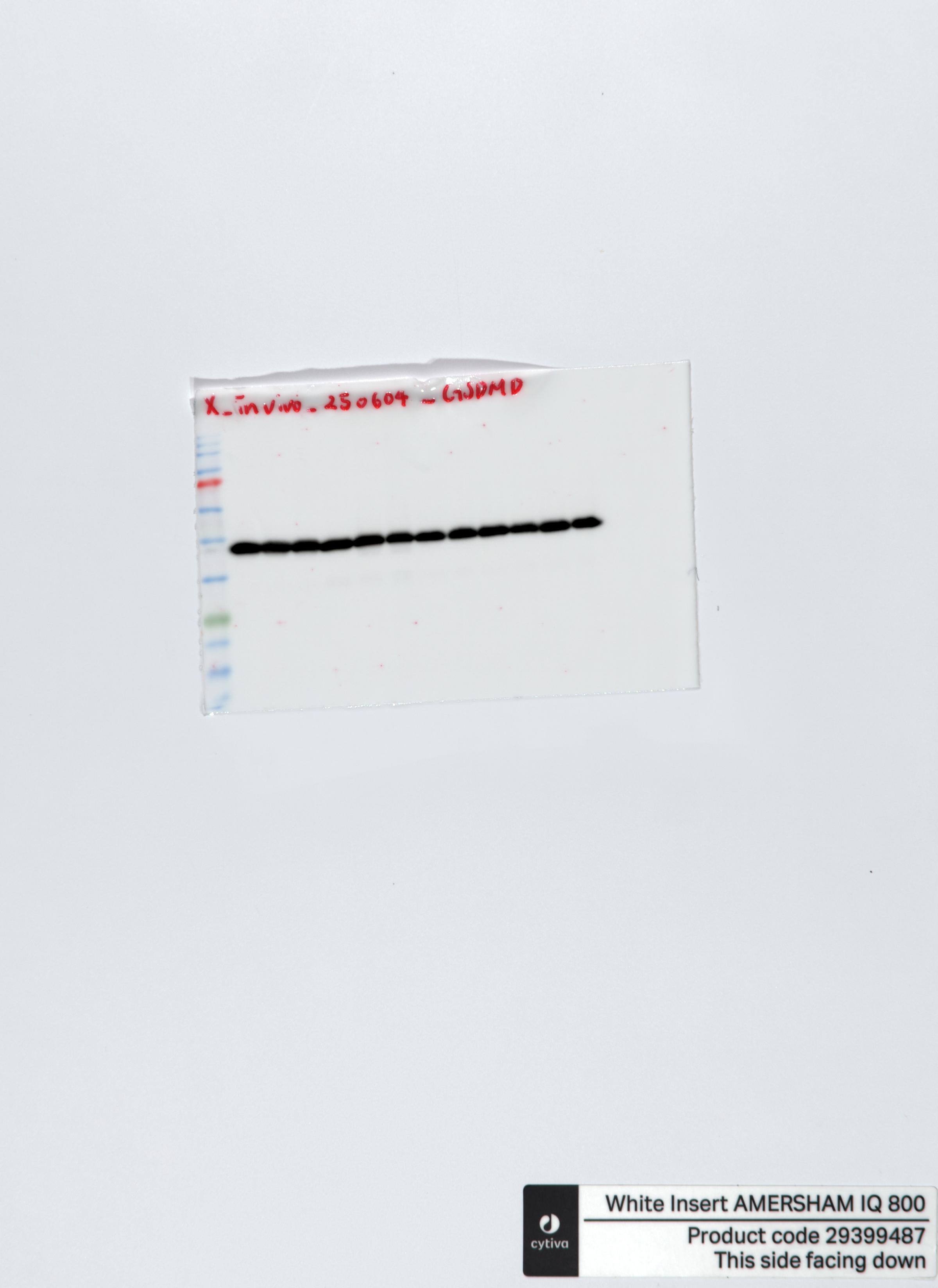

Supplement: Supplementary file 10 — Source data Fig. 8 [file 44321_2026_425_MOESM10_ESM.zip › Figure 8 Source Data/8E/8E_b-actin.jpg]

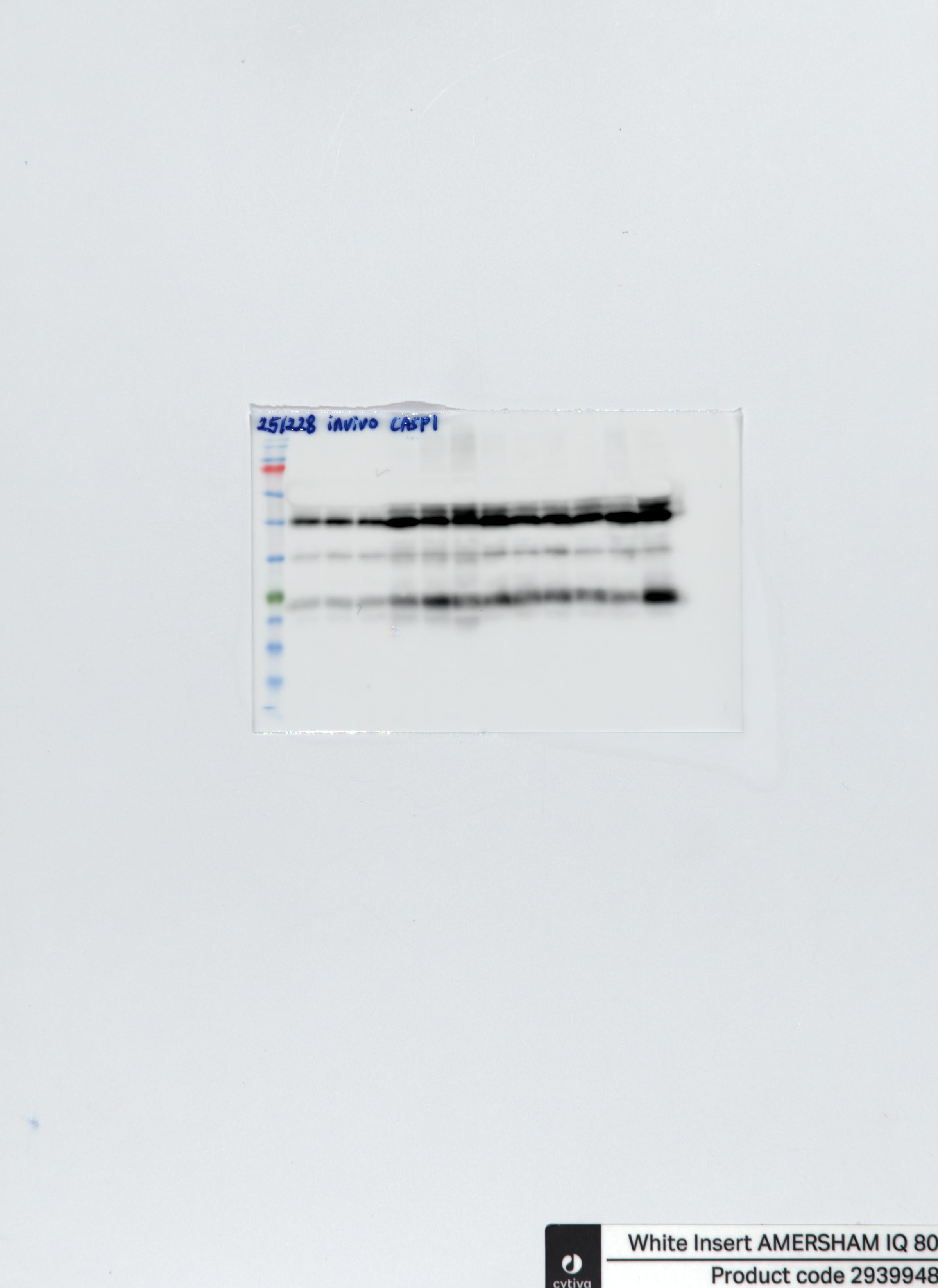

Supplement: Supplementary file 10 — Source data Fig. 8 [file 44321_2026_425_MOESM10_ESM.zip › Figure 8 Source Data/8E/8E_CASP1.jpg]

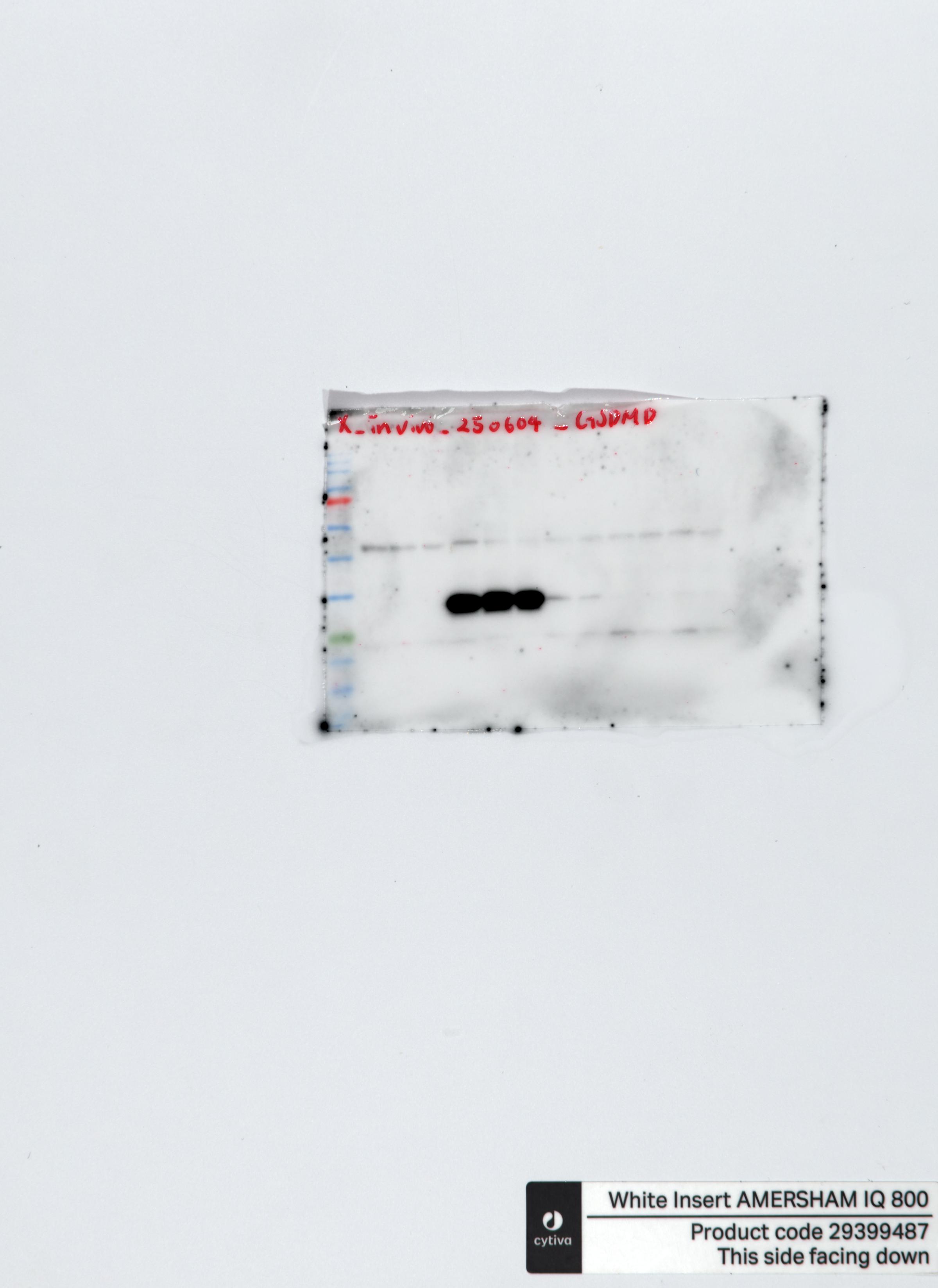

Supplement: Supplementary file 10 — Source data Fig. 8 [file 44321_2026_425_MOESM10_ESM.zip › Figure 8 Source Data/8E/8E_GSDMD.jpg]

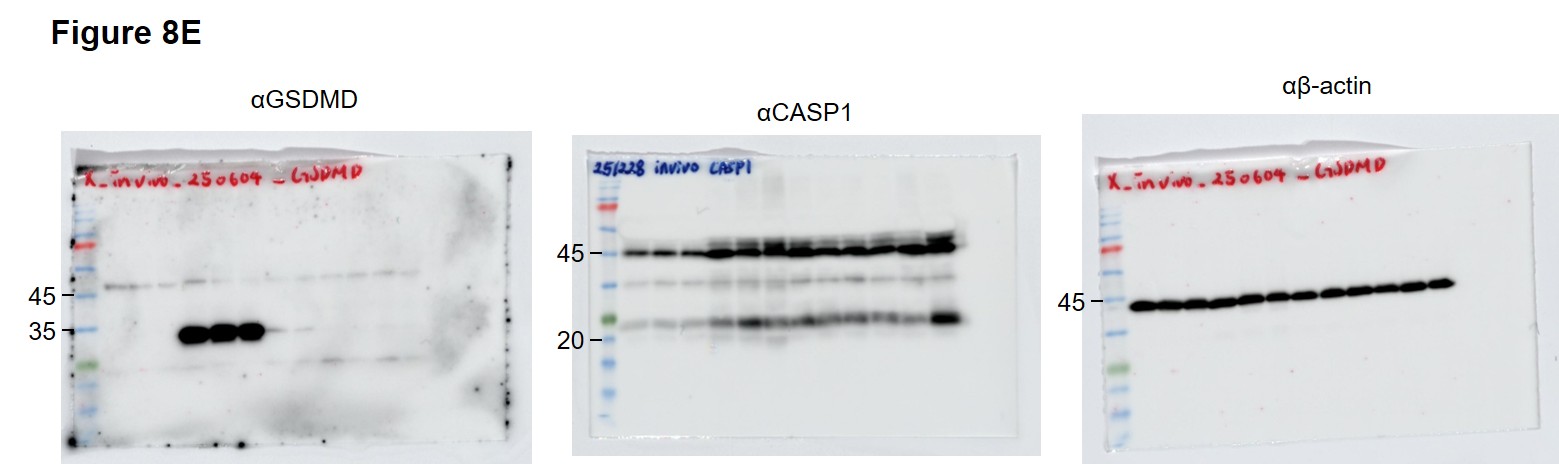

Supplement: Supplementary file 10 — Source data Fig. 8 [file 44321_2026_425_MOESM10_ESM.zip › Figure 8 Source Data/8E/8E.jpg]
