# Supplementary material for: Pharmacological targeting of the NLRP3 LRR domain with isothiazolinones overcomes CRID3-resistant inflammation
Source: EMBO Mol Med. 2026 Apr 17;18(6):2124–51. doi: 10.1038/s44321-026-00425-5 (PMC13269794; doi:10.1038/s44321-026-00425-5)
Supplement: Supplementary file 11 — Expanded View Figures [file 44321_2026_425_MOESM11_ESM.pdf]

Expanded View Figures

Figure EV1. LOC14 suppresses NLRP3 inflammasome activation.

(**A**) Immunoblot analysis of pro- (P45) and cleaved caspase-1 (CASP1; P20), and pro- (P53) and cleaved gasdermin D (GSDMD; P30) in wild-type (WT) and *Nlrp3*$^{-/-}$ bone-marrow-derived macrophages (BMDMs) primed with lipopolysaccharide (LPS) for 4 h and subsequently stimulated with imiquimod, with or without LOC14 or CRID3. (**B**) Immunoblot analysis of cleaved CASP1 and GSDMD from supernatants or full-length CASP1 and GSDMD from cell lysates of THP-1 cells primed with LPS and treated with nigericin plus LOC14 or CRID3 for 1 h. (**C–F**) Immunoblot analysis of pro- (P45) and cleaved CASP1 (P20), pro- (P53) and cleaved GSDMD (P30), and LDH (**C**), IL-1β release (**D**), real-time analysis (**E**) and representative images of cell death at 0 h and 6 h (**F**) in LPS-transfected WT BMDMs treated with or without LOC14 or CRID3. (**G**) Real-time analysis of cell death in influenza A virus (IAV)-infected WT BMDMs treated with vehicle or LOC14. (**H**) Immunoblot analysis of pro- (P45) and cleaved CASP1 (P20), pro- (P55) and cleaved caspase-8 (CASP8; P18), pro- (P35) and cleaved caspase-7 (CASP7; P20), and pro- (P35) and cleaved caspase-3 (CASP3; P19 and P17) in IAV-infected WT BMDMs treated with LOC14, as well as in *Nlrp3*$^{-/-}$ and *Zbp1*$^{-/-}$ BMDMs. Scale bar, 100 µm (**F**). GAPDH (**A**, **C**) and β-actin (**B**, **H**) were used as internal controls. Data are representative of at least three independent experiments. Data are shown as mean ± SEM (**D**, **E**, **G**).

© The Author(s)

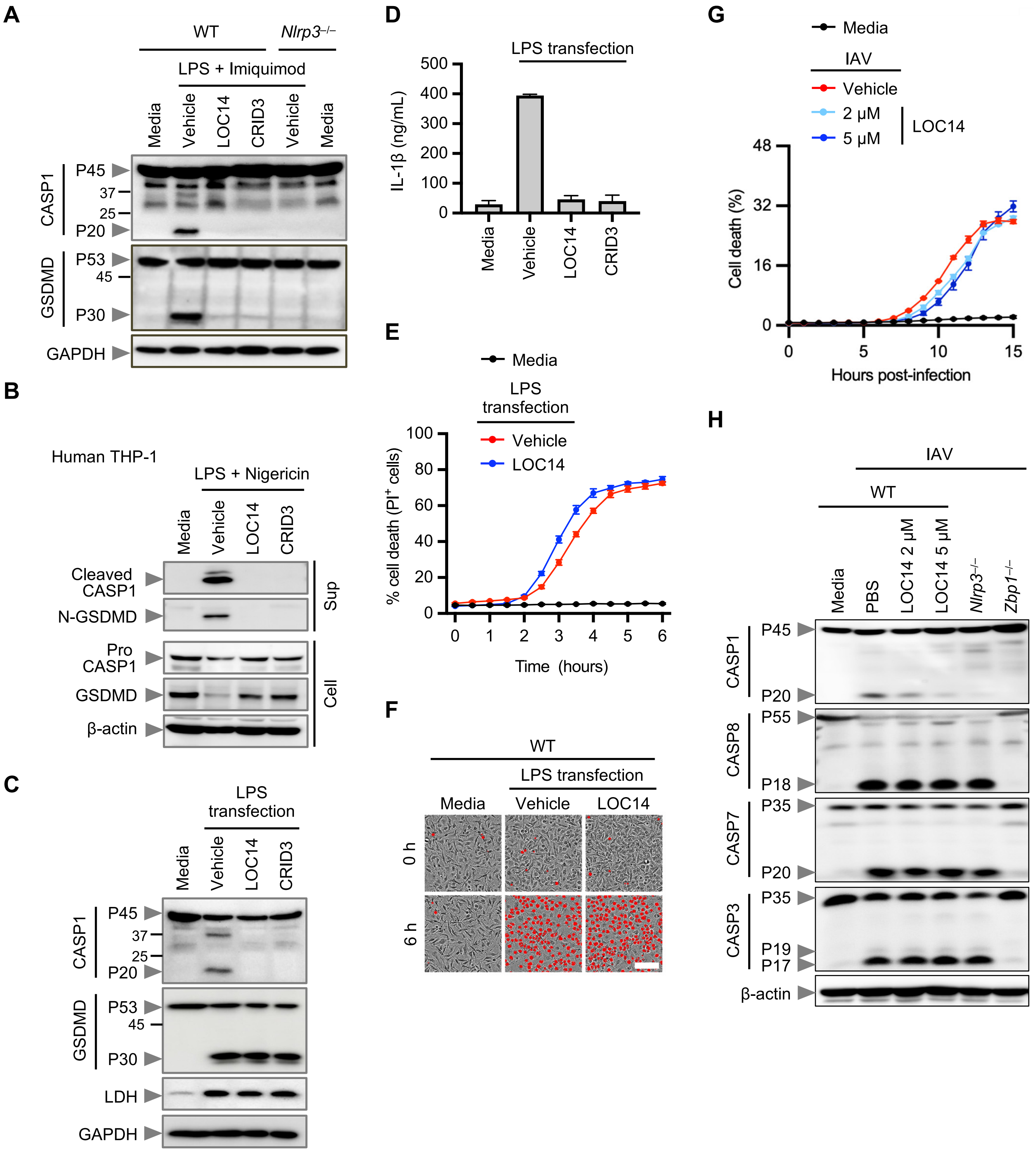
A
WT
Nlrp3−/−
LPS + Imiquimod
Media
Vehicle
LOC14
CRID3
Vehicle
Media
CASP1
P45
37
25
P20
GSDMD
P53
45
P30
GAPDH
B
Human THP-1
LPS + Nigericin
Media
Vehicle
LOC14
CRID3
Cleaved CASP1
N-GSDMD
Sup
Pro CASP1
GSDMD
β-actin
Cell
C
LPS transfection
Media
Vehicle
LOC14
CRID3
CASP1
P45
37
25
P20
GSDMD
P53
45
P30
LDH
GAPDH
D
LPS transfection
IL-1β (ng/mL)
500
400
300
200
100
0
Media
Vehicle
LOC14
CRID3
E
Media
LPS transfection
Vehicle
LOC14
% cell death (PI+ cells)
100
80
60
40
20
0
Time (hours)
F
WT
LPS transfection
Media
Vehicle
LOC14
0 h
6 h
G
Media
IAV
Vehicle
2 μM
5 μM
LOC14
Cell death (%)
48
32
16
0
Hours post-infection
H
IAV
WT
Media
PBS
LOC14 2 μM
LOC14 5 μM
Nlrp3−/−
Zbp1−/−
CASP1
P45
P20
CASP8
P55
P18
CASP7
P35
P20
CASP3
P35
P19
P17
β-actin

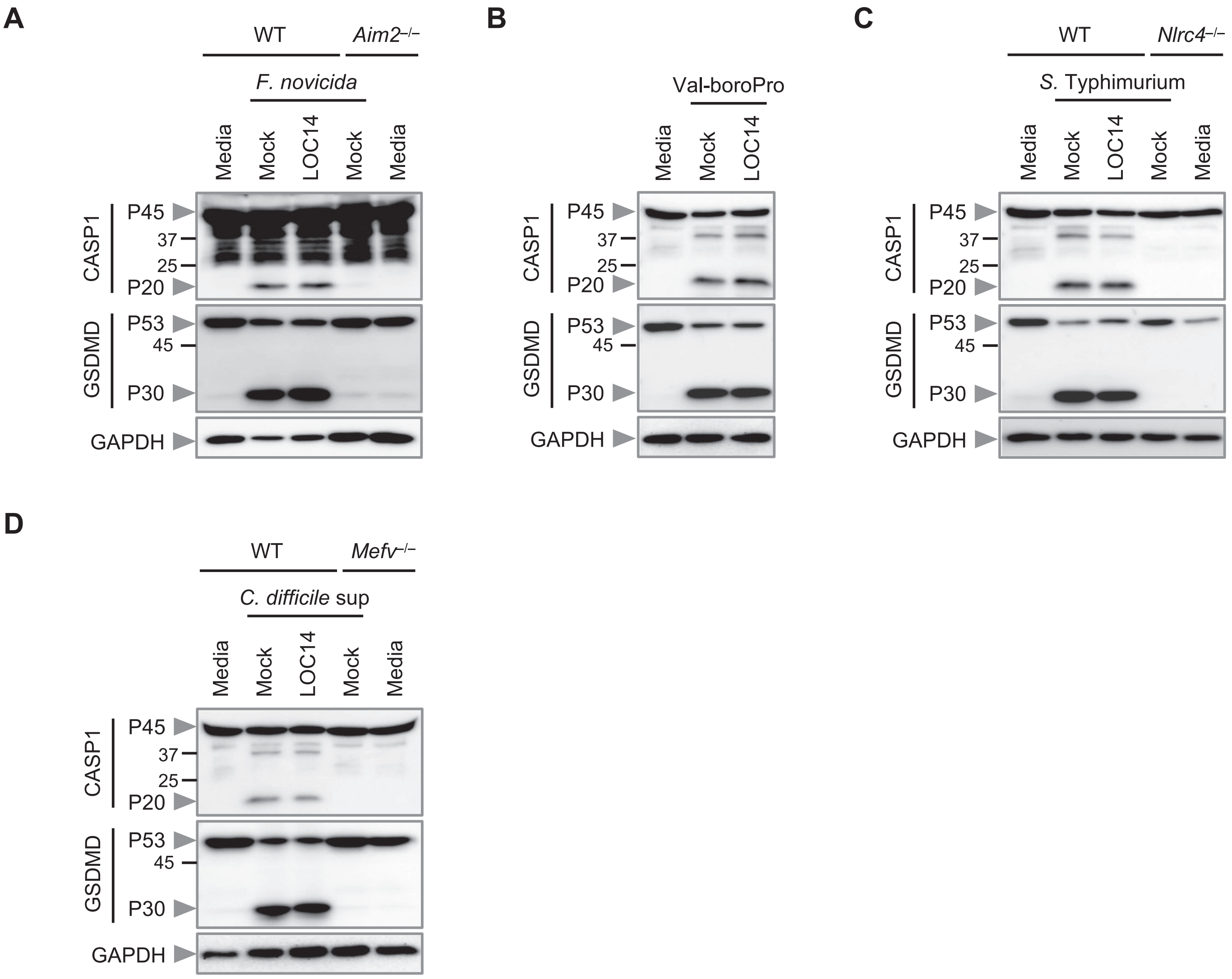

Figure EV2. LOC14 does not block caspase-1 activation or GSDMD cleavage downstream of AIM2, NLRP1b, NLRC4, or Pyrin inflammasomes.

(**A–D**) Immunoblot analysis of pro- (P45) and cleaved caspase-1 (CASP1; P20), and pro- (P53) and cleaved gasdermin D (GSDMD; P30) in bone marrow-derived macrophages (BMDMs) treated with or without LOC14 under the indicated inflammasome-activating conditions: wild-type (WT) and *Aim2*$^{-/-}$ BMDMs infected with *Francisella novicida* (50 MOI) for 16 h (**A**); WT BMDMs treated with Val-boroPro for 16 h (**B**); WT and *Nlrc4*$^{-/-}$ BMDMs infected with *Salmonella enterica* subspecies *enterica* serovar Typhimurium (1 MOI) for 6 h (**C**); and WT and *Mefv*$^{-/-}$ BMDMs incubated with *Clostridioides difficile* supernatant for 20 h (**D**). GAPDH was used as an internal control (**A–D**). Data are representative of at least three independent experiments.

 © The Author(s)

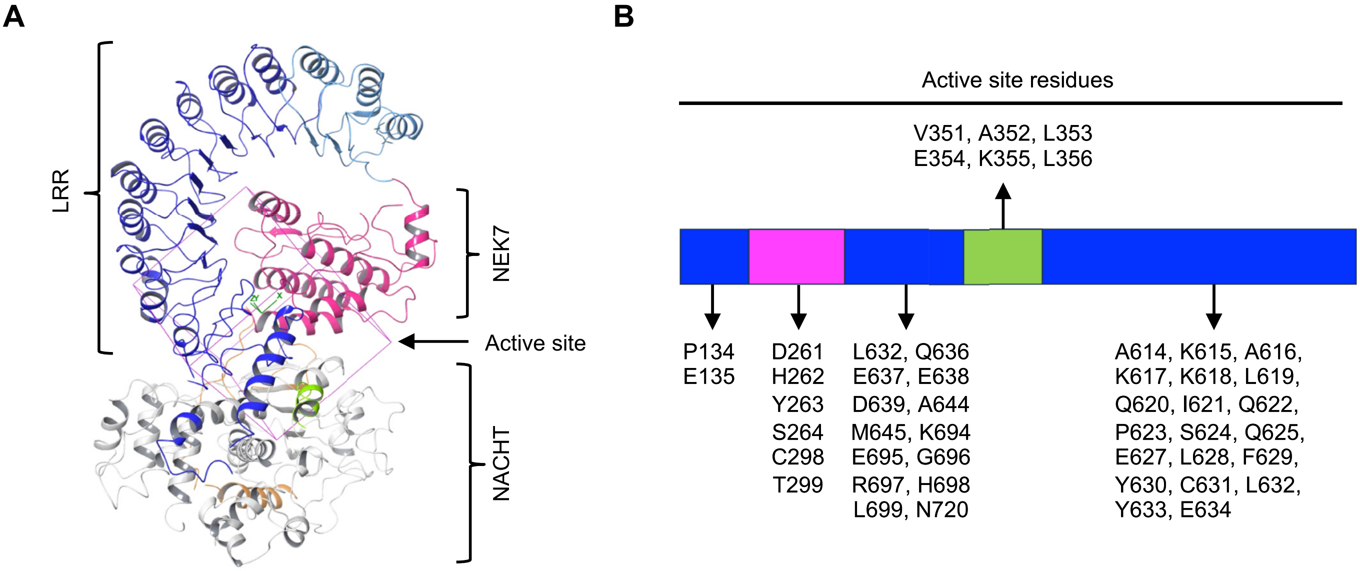

C

Time (ns)	ΔG bind (kcal/mol)	ΔG bind Coulomb (kcal/mol)	ΔG bind covalent (kcal/mol)	ΔG bind Van der (kcal/mol)	ΔG bind H Bond (kcal/mol)	ΔG bind Lipophilic (kcal/mol)	ΔG bind SolvGB (kcal/mol)
0	– 49.38	– 25.51	2.47	– 0.85	– 14.20	40.59	– 49.25
100	– 60.16	– 31.74	3.87	– 0.87	– 16.19	46.38	– 58.74
200	– 60.88	– 32.21	3.91	– 0.89	– 16.32	46.83	– 59.84
300	– 59.31	– 28.85	4.12	– 1.04	– 16.57	44.74	– 58.65
400	– 42.32	– 22.29	3.14	– 0.84	– 10.62	36.27	– 45.05
500	– 29.18	– 25.06	2.09	– 0.64	– 9.93	49.33	– 44.05
600	– 43.46	– 31.30	2.37	– 1.45	– 10.31	49.33	– 49.63
700	– 50.94	– 34.61	2.81	– 1.37	– 12.77	46.66	– 48.64
800	– 49.06	– 29.14	3.66	– 1.43	– 12.42	44.22	– 52.29
900	– 49.96	– 43.03	3.67	– 1.46	– 11.10	51.66	– 48.17
1000	– 43.52	– 35.79	3.33	– 1.37	– 10.31	48.18	– 45.99
Average	– 48.92	– 30.87	3.22	– 1.11	– 12.79	45.84	– 50.94

Figure EV3. LOC14 exhibits a higher degree of spontaneity in its interaction with NLRP3-NEK7.

(**A**, **B**) Structural analysis of NLRP3-NEK7 active site. (**C**) MM/GBSA profiles of LOC14 in interaction with NLRP3 bound to NEK7.

© The Author(s)

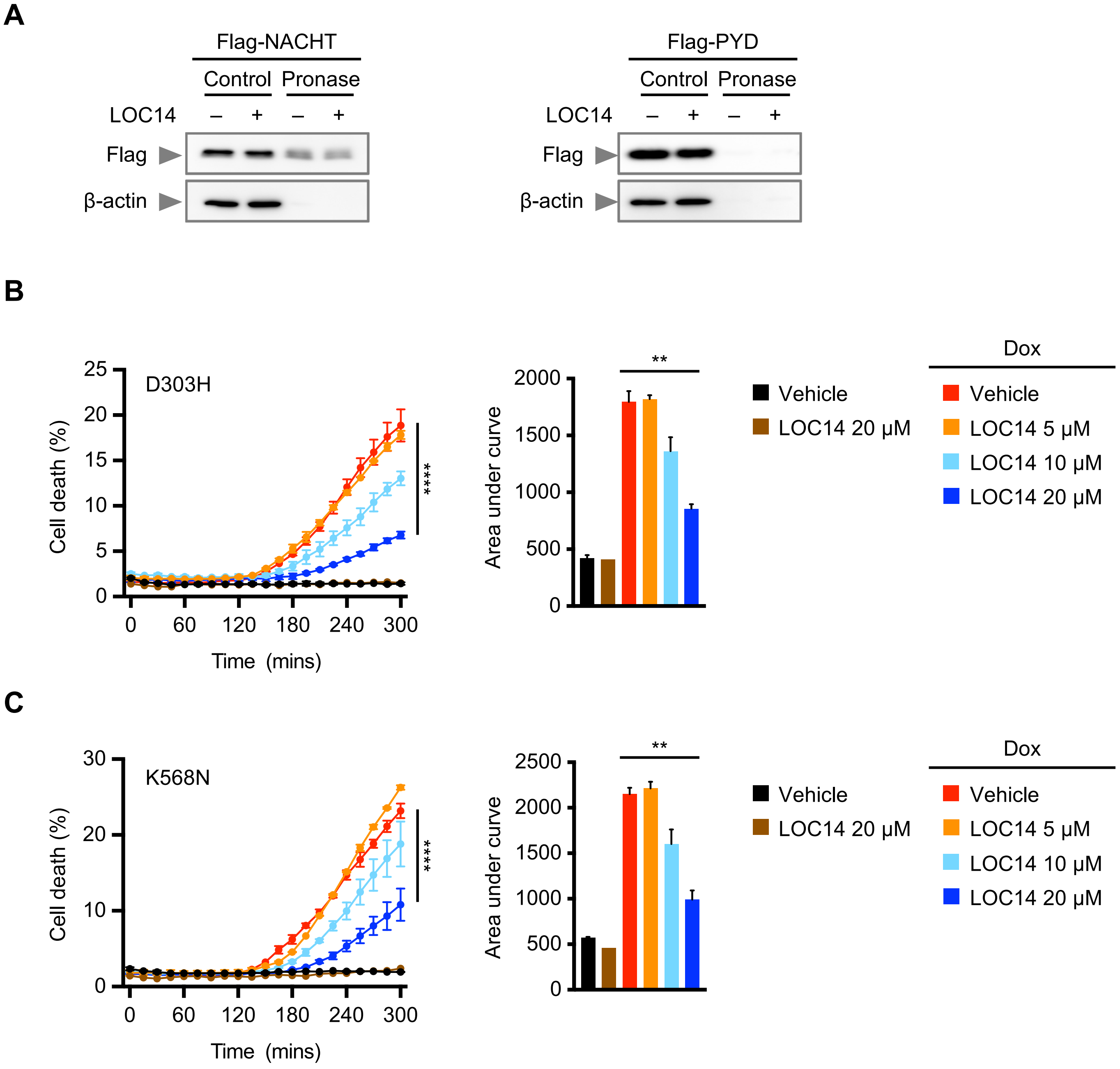

Figure EV4. LOC14 suppresses both MCC950-sensitive and -resistant inflammation.

(**A**) Immunoblot analysis of Flag in HEK293T cells expressing either NACHT or PYD domains of NLRP3, treated with or without LOC14 and pronase. (**B**, **C**) Human monocytic cell line U937 expressing doxycycline-inducible NLRP3 gain-of-function mutants associated with cryopyrin-associated periodic syndrome, D303H (**B**) and K568N (**C**), were treated with the vehicle control DMSO or LOC14. For the time-course data, Significance was evaluated using two-way ANOVA followed by Tukey's multiple comparisons test. ****$P < 0.0001$. Vehicle + Dox vs LOC14 20 μM + Dox, $P = 4E\text{-}15$. For the AUC quantification, Significance was evaluated using one-way ANOVA followed by Tukey's multiple comparisons test. **$P < 0.01$. Vehicle + Dox vs LOC14 20 μM + Dox, $P = 0.0014$ (**B**, **C**). β-actin was used as an internal control (**A**). Data are representative of at least three independent experiments (**A**). Data are from two independent experiments ($n = 2$) (**B**, **C**).

 © The Author(s)
