# Supplementary material for: Pharmacological targeting of the NLRP3 LRR domain with isothiazolinones overcomes CRID3-resistant inflammation
Source: EMBO Mol Med. 2026 Apr 17;18(6):2124–51. doi: 10.1038/s44321-026-00425-5 (PMC13269794; doi:10.1038/s44321-026-00425-5)
Supplement: Supplementary file 2 — Peer Review File [file 44321_2026_425_MOESM2_ESM.pdf]

EMBO Molecular Medicine

Pharmacological Targeting of NLRP3 with Isothiazolinones Overcomes CRID3-Resistant Inflammation

Hawon Woo, Yeonseo Jang, Soyeon Kim, Wonyoung Kim, Fenfen Zhang, Raghvendra Mall, Chirag Patel, Melan Kurera, Chinh Ngo, Simon Jiang, Asia Nicotra, Benedicte Py, Min Zheng, Si Ming Man, and Rajendra Karki

Corresponding authors: Si Ming Man (siming.man@anu.edu.au) , Min Zheng (min.zheng@szbl.ac.cn)

Review Timeline:

Submission Date:	6th Nov 25
Editorial Decision:	18th Dec 25
Revision Received:	14th Feb 26
Editorial Decision:	3rd Mar 26
Revision Received:	20th Mar 26
Accepted:	25th Mar 26

Editor: Zeljko Durdevic

Transaction Report:

(Note: With the exception of the correction of typographical or spelling errors that could be a source of ambiguity, letters and reports are not edited. Depending on transfer agreements, referee reports obtained elsewhere may or may not be included in this compilation. Referee reports are anonymous unless the Referee chooses to sign their reports.)

18th Dec 2025

Dear Prof. Man,

Thank you for the submission of your manuscript to EMBO Molecular Medicine, and please accept my apologies for the unusual delay in getting back to you. We have now received feedback from two of the three reviewers who agreed to evaluate your manuscript. As the referee #2 will unfortunately not be able to return his/her report in a timely manner, we prefer to make a decision now in order to avoid further delay in the process. Should referee #2 provide a report, we will send it to you, with the understanding that we will not ask for an additional revision.

As you will see from their reports pasted below, both referees recognize potential interest of the manuscript but also raise important concerns that should be addressed in a major revision. If you would like to discuss further the points raised by the referees, I am available to do so via email or video. Let me know if you are interested in this option.

Further consideration of a revision that addresses reviewers' concerns in full will entail a second round of review. EMBO Molecular Medicine encourages a single round of revision only and therefore, acceptance or rejection of the manuscript will depend on the completeness of your responses included in the next, final version of the manuscript. For this reason, and to save you from any frustrations in the end, I would strongly advise against returning an incomplete revision.

We would welcome the submission of a revised version within three months for further consideration. Please let us know if you require longer to complete the revision.

I look forward to receiving your revised manuscript.

Yours sincerely,

Zeljko Durdevic

Zeljko Durdevic
Senior Editor
EMBO Molecular Medicine

*****
When preparing your revised manuscript, please refer to our guidelines: https://link.springer.com/journal/44321/submission-guidelines#cms-Revised-submissions. We perform an initial quality control of all revised manuscripts before re-review; failure to include requested items will delay the evaluation of your revision.

We require:

1) A .docx formatted version of the manuscript text (including legends for main figures, EV figures and tables). Please make sure that the changes are highlighted to be clearly visible.

2) Individual production quality figure files as .eps, .tif, .jpg (one file per figure). For guidance, download the 'Figure Guide PDF': https://media.springernature.com/original/springer-cms/rest/v1/content/27825798/data/v1.

3) A .docx formatted letter INCLUDING the reviewers' reports and your detailed point-by-point responses to their comments. As part of the EMBO Press transparent editorial process, the point-by-point response is part of the Review Process File (RPF), which will be published alongside your paper.

4) A complete author checklist, which you can download from our author guidelines. Please insert information in the checklist that is also reflected in the manuscript. The completed author checklist will also be part of the RPF.

5) Please note that all corresponding authors are required to supply an ORCID ID for their name upon submission of a revised

manuscript.

6) It is mandatory to include a 'Data Availability' section after the Materials and Methods. Before submitting your revision, primary datasets produced in this study need to be deposited in an appropriate public database, and the accession numbers and database listed under 'Data Availability'. Please remember to provide a reviewer password if the datasets are not yet public.

In case you have no data that requires deposition in a public database, please state so in this section. Note that the Data Availability Section is restricted to new primary data that are part of this study.

7) For data quantification: please specify the name of the statistical test used to generate error bars and P values, the number (n) of independent experiments (specify technical or biological replicates) underlying each data point and the test used to calculate p-values in each figure legend. The figure legends should contain a basic description of n, P and the test applied. Graphs must include a description of the bars and the error bars (s.d., s.e.m.).

8) At EMBO Press we ask authors to provide source data for the main manuscript figures. You will receive a separate email with instructions for providing source data with your revised manuscript, including how to upload and organize the files.

9) Our journal encourages inclusion of *data citations in the reference list* to directly cite datasets that were re-used and obtained from public databases. Data citations in the article text are distinct from normal bibliographical citations and should directly link to the database records from which the data can be accessed. In the main text, data citations are formatted as follows: "Data ref: Smith et al, 2001" or "Data ref: NCBI Sequence Read Archive PRJNA342805, 2017". In the Reference list, data citations must be labeled with "[DATASET]". A data reference must provide the database name, accession number/identifiers and a resolvable link to the landing page from which the data can be accessed at the end of the reference.

10) We replaced Supplementary Information with Expanded View (EV) Figures and Tables that are collapsible/expandable online. A maximum of 5 EV Figures can be typeset. EV Figures should be cited as 'Figure EV1, Figure EV2" etc... in the text and their respective legends should be included in the main text after the legends of regular figures.

- For the figures that you do NOT wish to display as Expanded View figures, they should be bundled together with their legends in a single PDF file called *Appendix*, which should start with a short Table of Content. Appendix figures should be referred to in the main text as: "Appendix Figure S1, Appendix Figure S2" etc.

- Additional Tables/Datasets should be labeled and referred to as Table EV1, Dataset EV1, etc. Legends have to be provided in a separate tab in case of .xls files. Alternatively, the legend can be supplied as a separate text file (README) and zipped together with the Table/Dataset file.

11) The paper explained: EMBO Molecular Medicine articles are accompanied by a summary of the articles to emphasize the major findings in the paper and their medical implications for the non-specialist reader. Please provide a draft summary of your article highlighting

- the medical issue you are addressing,

- the results obtained and

- their clinical impact.

This may be edited to ensure that readers understand the significance and context of the research. Please refer to any of our published articles for an example.

12) Author contributions: You will be asked to provide CRediT (Contributor Role Taxonomy) terms in the submission system. These replace a narrative author contribution section in the manuscript.

13) A Conflict of Interest statement should be provided in the main text.

14) Every published paper includes a 'Synopsis' to further enhance discoverability. Synopses are displayed on the journal webpage and are freely accessible to all readers. They include a short stand first (maximum of 300 characters, including space) as well as 2-5 one-sentences bullet points that summarizes the paper. Please write the bullet points to summarize the key NEW findings. They should be designed to be complementary to the abstract - i.e. not repeat the same text. We encourage inclusion of key acronyms and quantitative information (maximum of 30 words / bullet point). Please use the passive voice. Please attach these in a separate file or send them by email, we will incorporate them accordingly.

Please also suggest a striking image or visual abstract to illustrate your article as a PNG file 550 px wide x 300-600 px high.

15) Include a Reagents and Tools Table as part of the Methods section, which can be downloaded from our author guidelines.

EMBO Molecular Medicine has a "scooping protection" policy, whereby similar findings that are published by others during review or revision are not a criterion for rejection. Should you decide to submit a revised version, I do ask that you get in touch after three months if you have not completed it, to update us on the status.

***** Reviewer's comments *****

Referee #1 (Comments on Novelty/Model System for Author):

The proposed NLRP3 targeting small molecule is novel and targets distinct domain of NLRP3 than the previously reported NLRP3 inhibitors.
The study is technically sound and considers state-of-the-art inflammasome assays.

Referee #1 (Remarks for Author):

The authors in the manuscript have addressed a critical gap in the development of anti-inflammatory therapeutics by identifying and characterizing LOC14, a selective inhibitor of NLRP3. NLRP3 inflammasome is a crucial inducer of cell death and inflammation in multiple infections and inflammatory diseases. Given the increasing importance of NLRP3 in various disease contexts, it is necessary to identify and develop NLRP3-targeting molecules with better efficiency. The authors have shown that LOC14 binds to or near the LRR domain and not only targets NLRP3 but also MCC950-resistant gain-of-function variants of NLRP3. The mechanism of inhibition has been investigated to find that LOC14 targets NLRP3-NEK7 interaction, further inhibiting oligomerization and inflammasome activation. The efficacy of LOC14 has been tested in different mouse models, and the data show promising effects of LOC14. Overall, the work is interesting and provides an intriguing lead molecule targeting NLRP3 inflammasome. I have a few comments that need to be addressed:

1. The data indicate that LOC14 disrupts NLRP3-NEK7 complex formation. Does LOC14 act by directly competing at the NLRP3-NEK7 interface or indirectly through altered NEK7 expression or stability. Assessing NEK7 mRNA and protein levels following LOC14 treatment, as well as evaluating NEK7 turnover dynamics, would help determine whether LOC14's inhibitory activity is mediated through NEK7 depletion or destabilization.

2. The study suggests that LOC14 inhibits IAV-induced NLRP3 inflammasome activation, but the role of NLRP3 during IAV infection is complex. NLRP3 is not required for cell death during IAV infection; instead, ZBP1 is essential for both cell death and NLRP3 inflammasome activation in this context. It is therefore important to clarify whether LOC14 inhibits only NLRP3 inflammasome activation or also affects ZBP1-dependent cell death pathways during IAV infection or other ZBP1-activating stimuli. Measuring cytokine production (e.g., IL-1β, IL-18) in LOC14-treated, IAV-infected cells would help address this.

3. The IC_{50} value of LOC14 has been determined only for cell death. It would be informative to also determine IC_{50} values for cytokines such as IL-1β or IL-18 to provide a more complete profile of LOC14's inhibitory potency.

4. It is not established whether LOC14 binds NLRP3 reversibly or irreversibly. Time-course or washout experiments would help determine LOC14's binding kinetics and define its mode of inhibition.

5. The docking analysis suggests that LOC14 forms hydrogen bonds with G696 and R697 within the transitional LRR domain of NLRP3, mediated by its carbonyl oxygen groups. However, functional evidence supporting the importance of these residues is lacking. Site-directed mutagenesis (e.g., G696A or R697A) followed by LOC14 binding assays or inflammasome inhibition assays would provide strong support for these residues as critical contact points in the LOC14-NLRP3 interaction.

6. LOC14 is a known allosteric inhibitor of PDIA3, raising the possibility of indirect effects on NLRP3 activation via altered ER redox balance. To rule out PDIA3-dependent mechanisms, a redox-modulation assay could be performed: under LPS + nigericin stimulation, treat cells with reducing (DTT) or oxidizing (Diamide) conditions in the presence or absence of LOC14. If LOC14's inhibitory effect remains unchanged across redox conditions, this would support a PDIA3-independent mechanism of action.

7. Although the trLRR domain is highlighted in the title, it is not mentioned anywhere in the text. Authors are suggested to revise the text to include trLRR domain in the text.

Referee #3 (Remarks for Author):

The authors in this study report the identification of LOC14 as a first in class NLRP3 inhibitor which is capable of inhibiting both MCC950-responsive and -resistant gain of function NLRP3 variants. The study is improtant considering that novel NLRP3 inhibitors are much needed for the treatment of a wide range of inflammatory, autoinflammatory and metabolic diseases.

1. Fig. 1: The authors should provide a clear timeline for LOC14 treatment. Was the compound added before LPS, together with LPS, or only at the time of ATP addition? This needs to be explicitly stated to allow proper interpretation of the priming versus activation effects.
2. In relation to Fig. 3D, did the authors test any of the other inflammasomes examined in Fig. 2 for resistance to pronase treatment in primary BMDMs?
3. Supplementary Fig. 10: NLRP3 expression should be included in these immunoblots to verify protein levels under each condition.
4. Line 263: IL-1β mRNA expression is mentioned but not shown in the corresponding figures. These data should either be included or the text revised.
5. Fig. 8: Are caspase-1 and IL-1β protein levels reduced in these animals as well? Including these measurements would provide a more complete assessment of inflammasome inhibition in vivo.
6. Line 68: The references cited for gout and atherosclerosis appear to be swapped and should be corrected.

We thank the reviewers for their careful evaluation of our manuscript and for the insightful feedback provided throughout the review process. Their comments prompted us to re-examine key aspects of our study and to strengthen both the experimental support and the presentation of our findings. We are grateful to Reviewer 1 for the thoughtful and critical assessment of our work, which helped us refine the mechanistic interpretation of our results. We also thank Reviewer 3 for the positive evaluation of the study and for constructive suggestions that improved the clarity and presentation of the data.

In response to all comments, we have carefully addressed all points raised and thoroughly revised the manuscript. We believe that these revisions have strengthened the study and improved its overall clarity. A summary of the new data and changes introduced in the revised manuscript is provided below.

Reviewer: 1
The authors in the manuscript have addressed a critical gap in the development of anti-inflammatory therapeutics by identifying and characterizing LOC14, a selective inhibitor of NLRP3. NLRP3 inflammasome is a crucial inducer of cell death and inflammation in multiple infections and inflammatory diseases. Given the increasing importance of NLRP3 in various disease contexts, it is necessary to identify and develop NLRP3-targeting molecules with better efficiency. The authors have shown that LOC14 binds to or near the LRR domain and not only targets NLRP3 but also MCC950-resistant gain-of-function variants of NLRP3. The mechanism of inhibition has been investigated to find that LOC14 targets NLRP3-NEK7 interaction, further inhibiting oligomerization and inflammasome activation. The efficacy of LOC14 has been tested in different mouse models, and the data show promising effects of LOC14. Overall, the work is interesting and provides an intriguing lead molecule targeting NLRP3 inflammasome. I have a few comments that need to be addressed:

We sincerely thank the reviewer for recognizing the significance of our work. We appreciate the reviewer’s positive assessment of the mechanistic insights, including interaction with the LRR domain, inhibition of the NLRP3–NEK7 interaction, and activity against MCC950-resistant gain-of-function NLRP3 variants. We have carefully considered all of the comments and have revised the manuscript accordingly.

1. The data indicate that LOC14 disrupts NLRP3-NEK7 complex formation. Does LOC14 act by directly competing at the NLRP3-NEK7 interface or indirectly through altered NEK7 expression or stability. Assessing NEK7 mRNA and protein levels following LOC14 treatment, as well as evaluating NEK7 turnover dynamics, would help determine whether LOC14's inhibitory activity is mediated through NEK7 depletion or destabilization.

To determine whether LOC14 affects NEK7 expression or stability, we treated primary BMDMs with LPS in the presence or absence of LOC14 and assessed NEK7 mRNA and protein levels at 0, 1, and 3 h after stimulation. LOC14 treatment did not result in any appreciable changes in NEK7 mRNA or protein abundance over this time course. These results indicate that LOC14 does not disrupt NLRP3–NEK7 complex formation by inducing NEK7 depletion or destabilization, supporting a mechanism that is independent of altered NEK7 expression or turnover. We also note that NEK7 protein levels in the input controls shown in Fig. 3F were unaffected by LOC14 treatment, which is consistent with these observations. Because this information is already reflected in the existing data, we have not added additional figures to the revised manuscript but have clarified this point in the text.

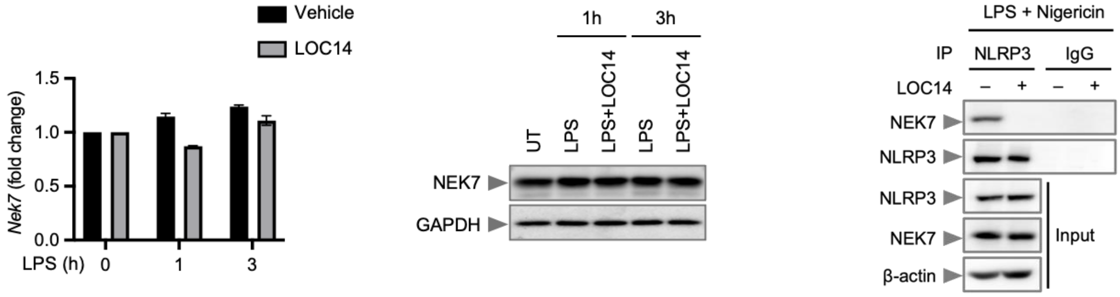

2. The study suggests that LOC14 inhibits IAV-induced NLRP3 inflammasome activation, but the role of NLRP3 during IAV infection is complex. NLRP3 is not required for cell death during IAV infection; instead, ZBP1 is essential for both cell death and NLRP3 inflammasome activation in this context. It is therefore important to clarify whether LOC14 inhibits only NLRP3 inflammasome activation or also affects ZBP1-dependent cell death pathways during IAV infection or other ZBP1-activating stimuli. Measuring cytokine production (e.g., IL-1β, IL-18) in LOC14-treated, IAV-infected cells would help address this.

We thank the reviewer for highlighting the complexity of inflammasome and cell death signaling during IAV infection and for emphasizing the need to distinguish effects on NLRP3 inflammasome activation from ZBP1-dependent cell death pathways.

To address this, we evaluated the impact of LOC14 on IAV-induced cell death and downstream signaling events associated with ZBP1 activation. LOC14 did not reduce overall IAV-induced cell death or ZBP1-associated caspase activation. In contrast, caspase-1 activation downstream of NLRP3 inflammasome assembly was selectively and dose-dependently reduced by LOC14 treatment (Fig. EV1G,H). These findings indicate that LOC14 does not broadly suppress IAV-triggered cell death pathways, including those mediated by ZBP1, but instead specifically inhibits NLRP3 inflammasome activation downstream of ZBP1 signaling. We have included this data in the revised manuscript (Fig. EV1G, H).

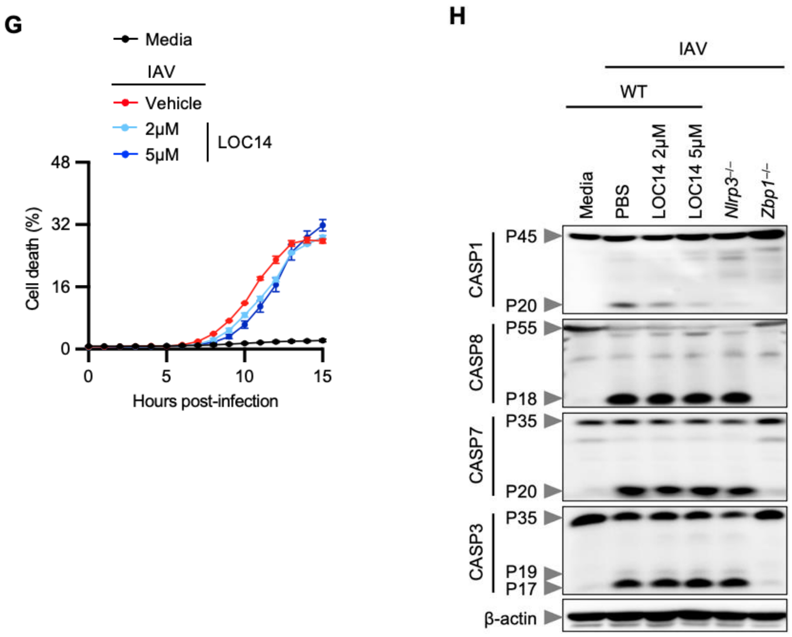

3. The IC_{50} value of LOC14 has been determined only for cell death. It would be informative to also determine IC_{50} values for cytokines such as IL-1β or IL-18 to provide a more complete profile of LOC14's inhibitory potency.

We performed dose–response analyses to determine its IC_{50} value for IL-1β release. Under LPS plus nigericin stimulation, we measured IL-1β release and found that LOC14 exhibited an IC_{50} of 0.98 μM, whereas the reference NLRP3 inhibitor CRID3 showed an IC_{50} of 0.35 μM (Appendix Fig. S1E).

IC50 (μM)	LPS + Nigericin	
	Cell death	IL-1β release
LOC14	1.551	0.9808
CRID3	0.6307	0.3538

4. It is not established whether LOC14 binds NLRP3 reversibly or irreversibly. Time-course or washout experiments would help determine LOC14's binding kinetics and define its mode of inhibition.

We thank the reviewer for this suggestion. As suggested by the reviewer, we performed washout experiments in primary BMDMs. Cells were primed with LPS for 4 h, treated with LOC14 or CRID3 for 30 min, and subsequently washed three times at 5-min intervals prior to nigericin stimulation. Following removal of the compounds, cell death was partially restored in both treatment groups, with a tendency toward greater recovery in CRID3-treated cells compared with LOC14-treated cells. These findings suggest that LOC14 exhibits more sustained target engagement with NLRP3 than CRID3. We have included this data in the revised manuscript (Appendix Fig. S6).

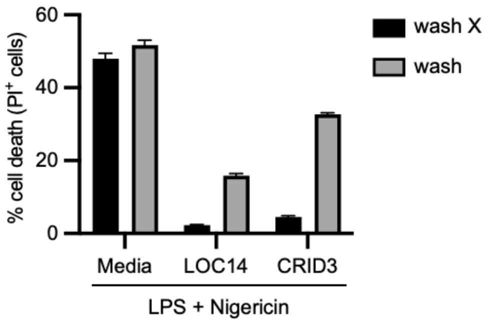

5. The docking analysis suggests that LOC14 forms hydrogen bonds with G696 and R697 within the transitional LRR domain of NLRP3, mediated by its carbonyl oxygen groups. However, functional evidence supporting the importance of these residues is lacking. Site-directed mutagenesis (e.g., G696A or R697A) followed by LOC14 binding assays or inflammasome inhibition assays would provide strong support for these residues as critical contact points in the LOC14-NLRP3 interaction.

We appreciate the reviewer for this insightful suggestion. To experimentally assess the contribution of G696 and R697 to LOC14 binding, as predicted by our docking analysis, we generated alanine substitution mutants of NLRP3 (G696A and R697A). Wild-type (WT) NLRP3, NLRP3-G696A, and NLRP3-R697A were overexpressed in HEK293T cells, and compound engagement was evaluated using DARTS assays. Compared with WT NLRP3, both mutants still exhibited partial protection from

protease-induced degradation in the presence of LOC14. These findings suggest that substitution of either G696 or R697 alone is insufficient to abolish LOC14 binding. Rather than relying on a single critical contact residue, LOC14 likely engages NLRP3 through a broader interaction surface or multivalent contacts within the LRR domain. We have included these data in the revised manuscript (Fig. 4E).

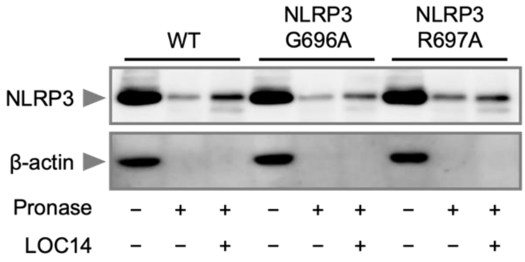

6. LOC14 is a known allosteric inhibitor of PDIA3, raising the possibility of indirect effects on NLRP3 activation via altered ER redox balance. To rule out PDIA3-dependent mechanisms, a redox-modulation assay could be performed: under LPS + nigericin stimulation, treat cells with reducing (DTT) or oxidizing (Diamide) conditions in the presence or absence of LOC14. If LOC14's inhibitory effect remains unchanged across redox conditions, this would support a PDIA3-independent mechanism of action.

We thank the reviewer for raising this important point regarding a potential PDIA3-dependent mechanism. Because LOC14 has been described as an allosteric inhibitor of PDIA3, we initially sought to directly determine whether its inhibitory effect on the NLRP3 inflammasome depends on PDIA3. First, we performed siRNA-mediated knockdown of PDIA3 in primary BMDMs and assessed NLRP3 inflammasome activation. PDIA3 silencing did not impair inflammasome activation, and LOC14 retained its inhibitory effect under these conditions (Appendix Fig. S2A–F), indicating

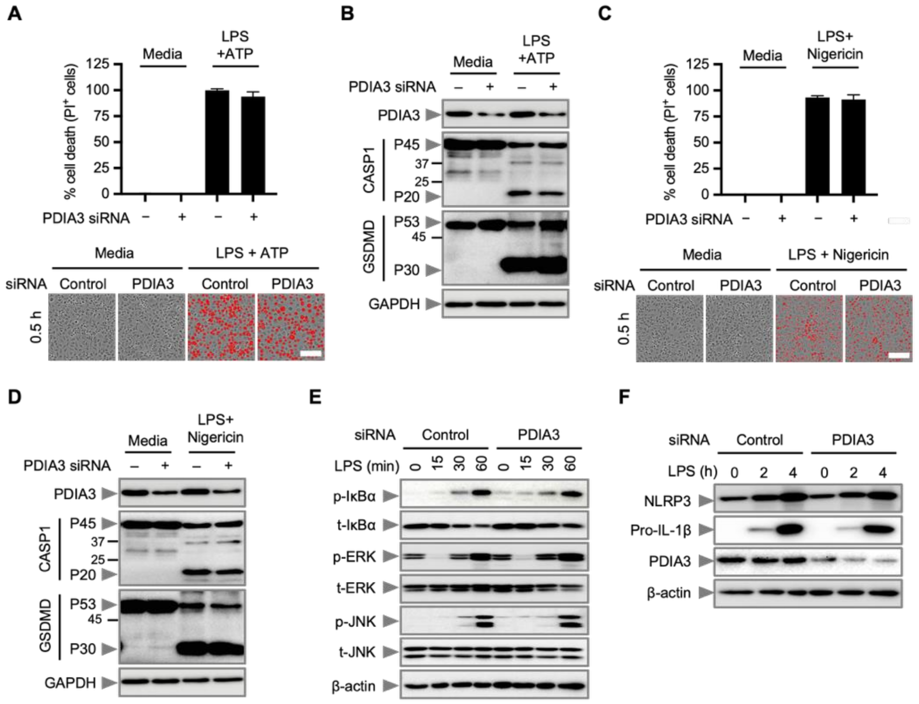

that PDIA3 is not required for NLRP3 activation or for LOC14-mediated inhibition.

In addition, as suggested by the reviewer, we examined whether LOC14 activity is sensitive to changes in the cellular redox environment. Treatment with the reducing agent dithiothreitol (DTT) did not diminish the ability of LOC14 to inhibit NLRP3 inflammasome activation. Together, these findings support a PDIA3-independent mechanism for LOC14 inhibition of the NLRP3 inflammasome.

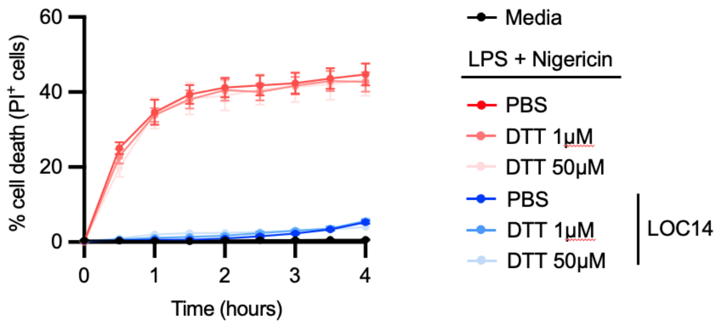

7. Although the trLRR domain is highlighted in the title, it is not mentioned anywhere in the text. Authors are suggested to revise the text to include trLRR domain in the text.

We have revised the main text to describe the transitional LRR (trLRR) domain of NLRP3 and its relevance to LOC14 binding. The trLRR domain is now clearly introduced and discussed in the context of our structural and functional analyses.

Reviewer: 3
The authors in this study report the identification of LOC14 as a first in class NLRP3 inhibitor which is capable of inhibiting both MCC950-responsive and -resistant gain of function NLRP3 variants. The study is important considering that novel NLRP3 inhibitors are much needed for the treatment of a wide range of inflammatory, autoinflammatory and metabolic diseases.

We thank the reviewer for highlighting the therapeutic potential of LOC14 as a novel NLRP3 inhibitor.

1. Fig. 1: The authors should provide a clear timeline for LOC14 treatment. Was the compound added before LPS, together with LPS, or only at the time of ATP addition? This needs to be explicitly stated to allow proper interpretation of the priming versus activation effects.

We appreciate the reviewer for clarifying the timing of LOC14 treatment. In Fig. 1, LOC14 was added after LPS priming, together with the inflammasome activation triggers (ATP or nigericin). This has now been explicitly stated in the revised figure legend to clearly distinguish effects on the activation step from those on priming. Specifically, we have clarified the experimental design as follows:

- **To validate the role of the new candidate LOC14 in suppressing NLRP3 inflammasome activation, we stimulated wild-type (WT) and *Nlrp3*$^{-/-}$ BMDMs with the NLRP3 activators LPS plus nigericin or LPS plus ATP, in the presence or absence of LOC14 or CRID3.**

- **To elucidate whether LOC14 also affects priming, BMDMs were treated with LOC14 during the LPS priming phase, or during the activation phase in the presence of nigericin or ATP (Appendix Fig. S9A).**

2. In relation to Fig. 3D, did the authors test any of the other inflammasomes examined in Fig. 2 for resistance to pronase treatment in primary BMDMs?

To assess whether LOC14 confers pronase resistance to other inflammasomes, we performed DARTS analysis. In contrast to NLRP3, LOC14 did not protect Pyrin from pronase-mediated proteolysis. Unfortunately, due to a lack of suitable and validated antibodies, DARTS analysis could not be performed for AIM2, NLRP1, or NLRC4.
However, our functional assays indicate that LOC14 does not affect the activation of these inflammasomes, with the exception of NLRP3. These new data have been included in the revised manuscript (Appendix Fig. S5A).
Together, these results demonstrate that LOC14 does not interact with Pyrin or other inflammasome components, supporting its specificity for NLRP3.

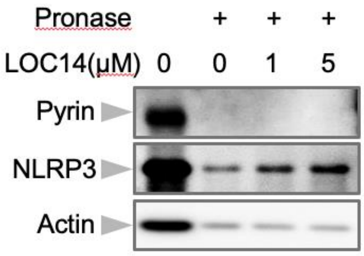

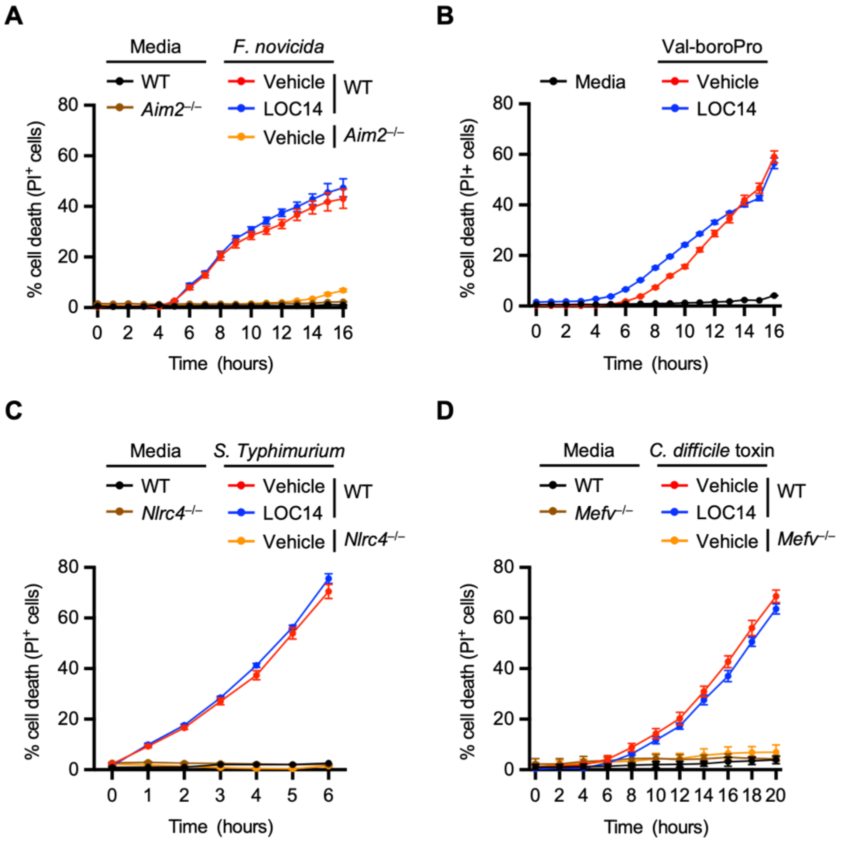

3. Supplementary Fig. 10: NLRP3 expression should be included in these immunoblots to verify protein levels under each condition.

As requested, NLRP3 expression has now been included in the immunoblots to verify protein levels under each experimental condition (Appendix Fig. S9D,G).

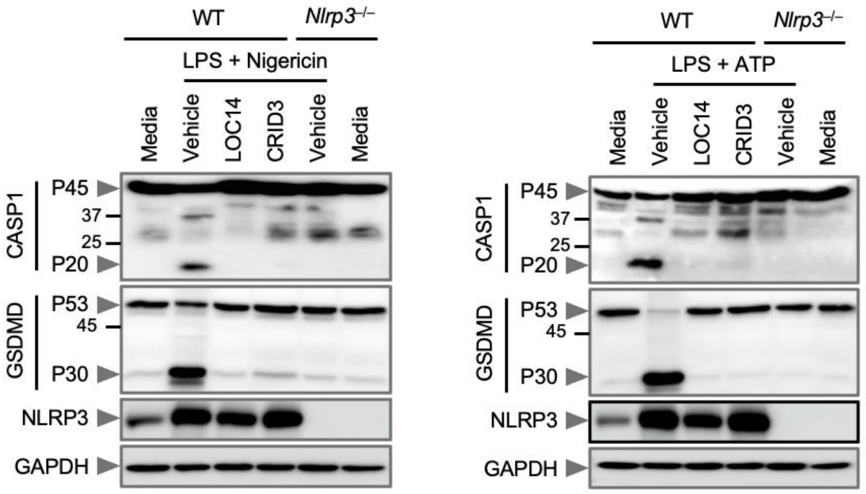

4. Line 263: IL-1β mRNA expression is mentioned but not shown in the corresponding figures. These data should either be included or the text revised.

We thank the reviewer for pointing this out. As suggested by the reviewer, the statement referring to IL-1β mRNA expression has been removed from the text.

5. Fig. 8: Are caspase-1 and IL-1β protein levels reduced in these animals as well? Including these measurements would provide a more complete assessment of inflammasome inhibition *in vivo*.

We thank the reviewer for this important suggestion. To further assess inflammasome inhibition *in vivo*, we examined caspase-1 cleavage by immunoblotting. Consistent with the GSDMD cleavage pattern, cleaved caspase-1 was markedly reduced in samples from LOC14- or CRID3-treated groups compared with vehicle controls (Fig. 8E).

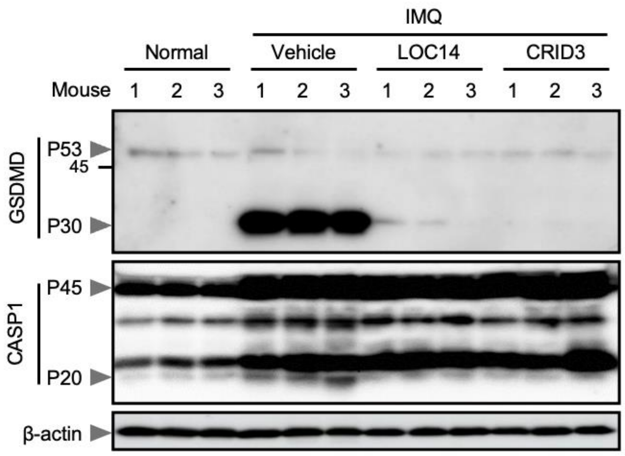

6. Line 68: The references cited for gout and atherosclerosis appear to be swapped and should be corrected.

We thank the reviewer for pointing this out. The references for gout and atherosclerosis at line 68 have been corrected accordingly.

3rd Mar 2026

Dear Prof. Man,

Thank you for submitting your revised manuscript to EMBO Molecular Medicine. Your manuscript will be ready for acceptance pending the following final amendments:

1) In the main manuscript file, please do the following:
- Please address all comments suggested by our data editors listed below:
o Data availability statement:
1. Please note that the exact p values are not provided in the legends of figures 1G, 4F, 6A, D; 7A, C, D; 8B, D.
2. Please note that n=2 in figures EV4 B, C.
- In the Abstract, please replace "first-in-class" with "selective".
- In Methods, statistical paragraph should reflect all information that you have filled in the Authors Checklist, especially regarding randomization, blinding, replication.
- Indicate in legends number and nature of replicates and exact p= values, not a range, along with the statistical test used. To keep the figures "clear" some authors found providing an Appendix table Sx with all exact p-values preferable. You are welcome to do this if you want to.
- Author contributions: Please remove it from the manuscript and specify author contributions in our submission system. CRediT has replaced the traditional author contributions section because it offers a systematic machine-readable author contributions format that allows for more effective research assessment. You are encouraged to use the free text boxes beneath each contributing author's name to add specific details on the author's contribution. More information is available in our guide to authors:
https://www.embopress.org/page/journal/17574684/authorguide#authorshipguidelines
- In data availability statement please replace the current text with "This study includes no data deposited in external repositories." Data availability statement should contain information only about raw data from datasets produced in this study. Datasets obtained from public resources should be cited at the appropriate place in the text (e.g. Methods section) together with the original article and included in the References in form of data citations. Please check our Author Guidelines for more information:
https://link.springer.com/journal/44321/submission-guidelines#cms-Reference-guidelines
2) Funding: Please add the funding mentioned in the comments box in our system to the line-by-line list of funders above.
3) The Paper Explained: Please provide "The Paper Explained" and add it to the main manuscript text. Please check "Author Guidelines" for more information. https://www.embopress.org/page/journal/17574684/authorguide#researcharticleguide
4) Synopsis: Every published paper now includes a 'Synopsis' to further enhance discoverability. Synopses are displayed on the journal webpage and are freely accessible to all readers. They include separate synopsis image and synopsis text.
- Synopsis image: Please provide a visual abstract as a high-resolution jpeg file 550 px-wide x 300-600 pixels high to illustrate your article.
- Synopsis text: Please provide a short standfirst (maximum of 300 characters, including space) as well as 2-5 one sentence bullet points that summarise the paper as a .doc file. Please write the bullet points to summarise the key NEW findings. They should be designed to be complementary to the abstract - i.e. not repeat the same text. We encourage inclusion of key acronyms and quantitative information (maximum of 30 words / bullet point). Please use the passive voice.
- Please check your synopsis text and image before submission with your revised manuscript. Please be aware that in the proof stage minor corrections only are allowed (e.g., typos).
5) As part of the EMBO Publications transparent editorial process (see our Editorial at http://embomolmed.embopress.org/content/2/9/329), EMBO Molecular Medicine will publish online a Review Process File (RPF) to accompany accepted manuscripts. This file will be published in conjunction with your paper and will include the anonymous referee reports, your point-by-point response and all pertinent correspondence relating to the manuscript. Let us know if you want to remove or not any figures from it prior to publication. Please note that the Authors checklist will be published at the end of the RPF.
6) Please provide a point-by-point letter INCLUDING my comments as well as the reviewer's reports and your detailed responses (as Word file).

I look forward to reading a new revised version of your manuscript as soon as possible.

Yours sincerely,

Zeljko Durdevic

Zeljko Durdevic
Senior Editor

EMBO Molecular Medicine

*** Instructions to submit your revised manuscript ***

*** PLEASE NOTE *** As part of the EMBO Publications transparent editorial process initiative (see our Editorial at https://www.embopress.org/doi/pdf/10.1002/emmm.201000094), EMBO Molecular Medicine will publish online a Review Process File to accompany accepted manuscripts.

In the event of acceptance, this file will be published in conjunction with your paper and will include the anonymous referee reports, your point-by-point response and all pertinent correspondence relating to the manuscript. If you do NOT want this file to be published, please inform the editorial office at contact@embomolmed.org.

*****

When preparing your revised manuscript, please refer to our guidelines: https://link.springer.com/journal/44321/submission-guidelines#cms-Revised-submissions. We perform an initial quality control of all revised manuscripts before re-review; failure to include requested items will delay the evaluation of your revision.

We require:

1) A .docx formatted version of the manuscript text (including legends for main figures, EV figures and tables). Please make sure that the changes are highlighted to be clearly visible.

2) Individual production quality figure files as .eps, .tif, .jpg (one file per figure). For guidance, download the 'Figure Guide PDF': https://media.springernature.com/original/springer-cms/rest/v1/content/27825798/data/v1.

3) A .docx formatted letter INCLUDING the reviewers' reports and your detailed point-by-point responses to their comments. As part of the EMBO Press transparent editorial process, the point-by-point response is part of the Review Process File (RPF), which will be published alongside your paper.

4) A complete author checklist, which you can download from our author guidelines. Please insert information in the checklist that is also reflected in the manuscript. The completed author checklist will also be part of the RPF.

5) Please note that all corresponding authors are required to supply an ORCID ID for their name upon submission of a revised manuscript.

6) It is mandatory to include a 'Data Availability' section after the Materials and Methods. Before submitting your revision, primary datasets produced in this study need to be deposited in an appropriate public database, and the accession numbers and database listed under 'Data Availability'. Please remember to provide a reviewer password if the datasets are not yet public.

In case you have no data that requires deposition in a public database, please state so in this section. Note that the Data Availability Section is restricted to new primary data that are part of this study.

7) For data quantification: please specify the name of the statistical test used to generate error bars and P values, the number (n) of independent experiments (specify technical or biological replicates) underlying each data point and the test used to calculate p-values in each figure legend. The figure legends should contain a basic description of n, P and the test applied. Graphs must include a description of the bars and the error bars (s.d., s.e.m.).

8) At EMBO Press we ask authors to provide source data for the main manuscript figures. You will receive a separate email with instructions for providing source data with your revised manuscript, including how to upload and organize the files.

9) Our journal encourages inclusion of *data citations in the reference list* to directly cite datasets that were re-used and obtained from public databases. Data citations in the article text are distinct from normal bibliographical citations and should directly link to the database records from which the data can be accessed. In the main text, data citations are formatted as follows: "Data ref: Smith et al, 2001" or "Data ref: NCBI Sequence Read Archive PRJNA342805, 2017". In the Reference list, data citations must be labeled with "[DATASET]". A data reference must provide the database name, accession

number/identifiers and a resolvable link to the landing page from which the data can be accessed at the end of the reference.

10) We replaced Supplementary Information with Expanded View (EV) Figures and Tables that are collapsible/expandable online. A maximum of 5 EV Figures can be typeset. EV Figures should be cited as 'Figure EV1, Figure EV2" etc... in the text and their respective legends should be included in the main text after the legends of regular figures.

- For the figures that you do NOT wish to display as Expanded View figures, they should be bundled together with their legends in a single PDF file called *Appendix*, which should start with a short Table of Content. Appendix figures should be referred to in the main text as: "Appendix Figure S1, Appendix Figure S2" etc.

- Additional Tables/Datasets should be labeled and referred to as Table EV1, Dataset EV1, etc. Legends have to be provided in a separate tab in case of .xls files. Alternatively, the legend can be supplied as a separate text file (README) and zipped together with the Table/Dataset file.

11) The paper explained: EMBO Molecular Medicine articles are accompanied by a summary of the articles to emphasize the major findings in the paper and their medical implications for the non-specialist reader. Please provide a draft summary of your article highlighting

- the medical issue you are addressing,

- the results obtained and

- their clinical impact.

This may be edited to ensure that readers understand the significance and context of the research. Please refer to any of our published articles for an example.

12) Author contributions: You will be asked to provide CRediT (Contributor Role Taxonomy) terms in the submission system. These replace a narrative author contribution section in the manuscript.

13) A Conflict of Interest statement should be provided in the main text.

14) Every published paper includes a 'Synopsis' to further enhance discoverability. Synopses are displayed on the journal webpage and are freely accessible to all readers. They include a short stand first (maximum of 300 characters, including space) as well as 2-5 one-sentences bullet points that summarizes the paper. Please write the bullet points to summarize the key NEW findings. They should be designed to be complementary to the abstract - i.e. not repeat the same text. We encourage inclusion of key acronyms and quantitative information (maximum of 30 words / bullet point). Please use the passive voice. Please attach these in a separate file or send them by email, we will incorporate them accordingly.

Please also suggest a striking image or visual abstract to illustrate your article as a PNG file 550 px wide x 300-600 px high.

15) Include a Reagents and Tools Table as part of the Methods section, which can be downloaded from our author guidelines.

EMBO Molecular Medicine has a "scooping protection" policy, whereby similar findings that are published by others during review or revision are not a criterion for rejection. Should you decide to submit a revised version, I do ask that you get in touch after three months if you have not completed it, to update us on the status.

*Additional important information regarding Figures

Each figure should be given in a separate file and should have the following resolution:
Graphs 800-1,200 DPI
Photos 400-800 DPI
Colour (only CMYK) 300-400 DPI"

Figures are not edited by the production team. All lettering should be the same size and style; figure panels should be indicated by capital letters (A, B, C etc). Gridlines are not allowed except for log plots. Figures should be numbered in the order of their appearance in the text with Arabic numerals. Each Figure must have a separate legend and a caption is needed for each panel.

*Additional important information regarding figures and illustrations can be found at
https://media.springernature.com/original/springer-cms/rest/v1/content/27825798/data/v1

***** Reviewer's comments *****

Referee #1 (Remarks for Author):

In this revised manuscript, Woo et al. have made substantial efforts to refine the quality of the study and strengthen the conceptual basis of their findings. The authors have addressed the comments raised and incorporated the suggested changes. Through the revision experiments, the authors have addressed a few critical points concerning the mechanistic details, potency, specificity and in vivo efficacy of LOC14, validating their initial findings suggesting the molecule as a promising therapeutic candidate. While multiple attempts are in progress for the identification and development of a potent and efficient NLRP3 inflammasome modulator, this study stands out as impactful due to its comprehensive approach. The outcomes of this study have significantly advanced the development of anti-inflammatory therapeutics, making it highly suitable for publishing in this journal.

The search for an effective NLRP3 modulator has accelerated over the past few years and multiple molecules have been identified as promising candidates. However, the absence of a clinically approved NLRP3 inhibitor has made it imperative for researchers to continue looking for suitable lead molecules. This study has identified LOC14 as a highly selective NLRP3 inhibitor that demonstrated to be having specific advantages compared to candidates in various aspects, including its activity against MCC950-resistant NLRP3 variants. Interestingly, the authors have highlighted the importance of the isothiazolinone core structure of LOC14 in its inhibitory function, opening the possibility of developing additional inhibitors with this functional group.

Referee #3 (Remarks for Author):

Is suitable for publication.

The authors addressed the remaining editorial issues.

25th Mar 2026

Dear Prof. Man,

We are pleased to inform you that your manuscript is accepted for publication and is now being sent to our publisher to be included in the next available issue of EMBO Molecular Medicine.

Your manuscript will be processed for publication by EMBO Press. It will be copy edited and you will receive page proofs prior to publication. Please note that you will be contacted by Springer Nature Author Services to complete licensing and payment information.

You may qualify for financial assistance for your publication charges - either via a Springer Nature fully open access agreement or an EMBO initiative. Check your eligibility: https://link.springer.com/journal/44321/how-to-publish-with-us

Should you be planning a Press Release on your article, please get in contact with embo_production@springernature.com as early as possible in order to coordinate publication and release dates.

If you have any questions, please do not hesitate to contact the Editorial Office. Thank you for your contribution to EMBO Molecular Medicine.

Yours sincerely,
Zeljko Durdevic

Zeljko Durdevic
Senior Editor
EMBO Molecular Medicine

>>> Please note that it is EMBO Molecular Medicine policy for the transcript of the editorial process (containing referee reports and your response letter) to be published as an online supplement to each paper. If you do NOT want this, you will need to inform the Editorial Office via email immediately. More information is available here: https://link.springer.com/partners/embo-press/editorial-policies#Peer%20review

EMBO Press Author Checklist

Corresponding Author Name: Min Zheng, Si Ming Man, Rajendra Karki	
Journal Submitted to: EMBO Molecular Medicine	
Manuscript Number: EMM-2025-22934-T	

USEFUL LINKS FOR COMPLETING THIS FORM
The EMBO Journal - Author Guidelines
EMBO Reports - Author Guidelines
Molecular Systems Biology - Author Guidelines
EMBO Molecular Medicine - Author Guidelines

Reporting Checklist for Life Science Articles (updated January
This checklist is adapted from Materials Design Analysis Reporting (MDAR) Checklist for Authors. MDAR establishes a minimum set of requirements in transparent reporting in the life sciences (see Statement of Task: 10.31222/osf.io/9sm4x). Please follow the journal's guidelines in preparing your
Please note that a copy of this checklist will be published alongside your article.

Abridged guidelines for figures
1. Data
The data shown in figures should satisfy the following conditions:
- the data were obtained and processed according to the field's best practice and are presented to reflect the results of the experiments in an accurate and unbiased manner.
- ideally, figure panels should include only measurements that are directly comparable to each other and obtained with the same assay.
- plots include clearly labeled error bars for independent experiments and sample sizes. Unless justified, error bars should not be shown for technical
- if n<5, the individual data points from each experiment should be plotted. Any statistical test employed should be justified.
- Source Data should be included to report the data underlying figures according to the guidelines set out in the authorship guidelines on Data

2. Captions
Each figure caption should contain the following information, for each panel where they are relevant:
- a specification of the experimental system investigated (eg cell line, species name).
- the assay(s) and method(s) used to carry out the reported observations and measurements.
- an explicit mention of the biological and chemical entity(ies) that are being measured.
- an explicit mention of the biological and chemical entity(ies) that are altered/varied/perturbed in a controlled manner.
- the exact sample size (n) for each experimental group/condition, given as a number, not a range;
- a description of the sample collection allowing the reader to understand whether the samples represent technical or biological replicates (including how many animals, litters, cultures, etc.).
- a statement of how many times the experiment shown was independently replicated in the laboratory.
- definitions of statistical methods and measures:
  - common tests, such as t-test (please specify whether paired vs. unpaired), simple χ2 tests, Wilcoxon and Mann-Whitney tests, can be unambiguously identified by name only, but more complex techniques should be described in the methods section;
  - are tests one-sided or two-sided?
  - are there adjustments for multiple comparisons?
  - exact statistical test results, e.g., P values = x but not P values < x;
  - definition of 'center values' as median or average;
  - definition of error bars as s.d. or s.e.m.

Please complete ALL of the questions below.
Select "Not Applicable" only when the requested information is not relevant for your study.

Materials

Newly Created Materials	Information included in the manuscript?	In which section is the information available? (Reagents and Tools Table, Materials and Methods, Figures, Data Availability Section)
New materials and reagents need to be available; do any restrictions apply?	Not Applicable	

Antibodies	Information included in the manuscript?	In which section is the information available? (Reagents and Tools Table, Materials and Methods, Figures, Data Availability Section)
For **antibodies** provide the following information:<br>- Commercial antibodies: RRID (if possible) or supplier name, catalogue number and or/clone number<br>- Non-commercial: RRID or citation	Yes	Reagents and Tools Table and Methods

DNA and RNA sequences	Information included in the manuscript?	In which section is the information available? (Reagents and Tools Table, Materials and Methods, Figures, Data Availability Section)
**Short novel DNA or RNA including primers, probes**: provide the sequences.	Yes	Reagents and Tools Table and Methods

Cell materials	Information included in the manuscript?	In which section is the information available? (Reagents and Tools Table, Materials and Methods, Figures, Data Availability Section)
**Cell lines:** Provide species information, strain. Provide accession number in repository **OR** supplier name, catalog number, clone number, and/**OR** RRID.	Yes	Reagents and Tools Table and Methods
**Primary cultures:** Provide species, strain, sex of origin, genetic modification status.	Yes	Reagents and Tools Table and Methods
Report if the cell lines were recently **authenticated** (e.g., by STR profiling) and tested for mycoplasma contamination.	Yes	Methods

Experimental animals	Information included in the manuscript?	In which section is the information available? (Reagents and Tools Table, Materials and Methods, Figures, Data Availability Section)
**Laboratory animals or Model organisms:** Provide species, strain, sex, age, genetic modification status. Provide accession number in repository **OR** supplier name, catalog number, clone number, **OR** RRID.	Yes	Reagents and Tools Table and Methods
**Animal observed in or captured from the field:** Provide species, sex, and age where possible.	Not Applicable	
Please detail **housing and husbandry conditions**.	Yes	Methods

Plants and microbes	Information included in the manuscript?	In which section is the information available? (Reagents and Tools Table, Materials and Methods, Figures, Data Availability Section)
**Plants:** provide species and strain, ecotype and cultivar where relevant, unique accession number if available, and source (including location for collected wild specimens).	Not Applicable	
**Microbes:** provide species and strain, unique accession number if available, and source.	Yes	Reagents and Tools Table and Methods

Human research participants	Information included in the manuscript?	In which section is the information available? (Reagents and Tools Table, Materials and Methods, Figures, Data Availability Section)
If collected and within the bounds of privacy constraints report on age, sex and gender or ethnicity for all study participants.	Yes	Methods

Core facilities	Information included in the manuscript?	In which section is the information available? (Reagents and Tools Table, Materials and Methods, Figures, Data Availability Section)
If your work benefited from core facilities, was their service mentioned in the acknowledgments section?	Yes	Acknowledgements

Design

Study protocol	Information included in the manuscript?	In which section is the information available? (Reagents and Tools Table, Materials and Methods, Figures, Data Availability Section)
If study protocol has been **pre-registered, provide DOI in the manuscript**. For clinical trials, provide the trial registration number **OR** cite DOI.	Not Applicable	
Report the **clinical trial registration number** (at ClinicalTrials.gov or equivalent), where applicable.	Not Applicable	

Laboratory protocol	Information included in the manuscript?	In which section is the information available? (Reagents and Tools Table, Materials and Methods, Figures, Data Availability Section)
Provide DOI OR other citation details if **external detailed step-by-step protocols** are available.	Not Applicable	

Experimental study design and statistics	Information included in the manuscript?	In which section is the information available? (Reagents and Tools Table, Materials and Methods, Figures, Data Availability Section)
Include a statement about **sample size** estimate even if no statistical methods were used.	Yes	Methods and Figure legends
Were any steps taken to minimize the effects of subjective bias when allocating animals/samples to treatment (e.g. **randomization procedure**)? If yes, have they been described?	Not Applicable	
Include a statement about **blinding** even if no blinding was done.	Not Applicable	
Describe **inclusion/exclusion criteria** if samples or animals were excluded from the analysis. Were the criteria pre-established? If sample or data points were omitted from analysis, report if this was due to attrition or intentional exclusion and provide justification.	Not Applicable	
For every figure, are **statistical tests** justified as appropriate? Do the data meet the assumptions of the tests (e.g., normal distribution)? Describe any methods used to assess it. Is there an estimate of variation within each group of data? Is the variance similar between the groups that are being statistically compared?	Yes	Methods and Figure legends

Sample definition and in-laboratory replication	Information included in the manuscript?	In which section is the information available? (Reagents and Tools Table, Materials and Methods, Figures, Data Availability Section)
In the figure legends: state number of times the experiment was **replicated** in laboratory.	Yes	Figure legends
In the figure legends: define whether data describe **technical or biological replicates**.	Yes	Figure legends

Ethics

Ethics	Information included in the manuscript?	In which section is the information available? (Reagents and Tools Table, Materials and Methods, Figures, Data Availability Section)
Studies involving **human participants**: State details of **authority granting ethics approval** (IRB or equivalent committee(s), provide reference number for approval.	Yes	Methods
Studies involving **human participants**: Include a statement confirming that **informed consent** was obtained from all subjects and that the experiments conformed to the principles set out in the WMA Declaration of Helsinki and the Department of Health and Human Services Belmont Report.	Yes	Methods
Studies involving **human participants: For publication of patient photos**, include a statement confirming that consent to publish was obtained.	Not Applicable	
Studies involving experimental **animals**: State details of **authority granting ethics approval** (IRB or equivalent committee(s), provide reference number for approval. Include a statement of compliance with ethical regulations.	Yes	Methods
Studies involving **specimen and field samples:** State if relevant **permits** obtained, provide details of authority approving study; if none were required, explain why.	Not Applicable	

Dual Use Research of Concern (DURC)	Information included in the manuscript?	In which section is the information available? (Reagents and Tools Table, Materials and Methods, Figures, Data Availability Section)
Could your study fall under dual use research restrictions? Please check biosecurity documents and list of **select agents and toxins** (CDC): https://www.selectagents.gov/sat/list.htm	Not Applicable	
If you used a select agent, is the security level of the lab appropriate and reported in the manuscript?	Not Applicable	
If a study is subject to dual use research of concern regulations, is the name of the **authority granting approval and reference number** for the regulatory approval provided in the manuscript?	Not Applicable	

Reporting

The MDAR framework recommends adoption of discipline-specific guidelines, established and endorsed through community initiatives. Journals have their own policy about requiring specific guidelines and recommendations to complement MDAR.

Adherence to community standards	Information included in the manuscript?	In which section is the information available? (Reagents and Tools Table, Materials and Methods, Figures, Data Availability Section)
State if relevant guidelines or checklists (e.g., **ICMJE, MIBBI, ARRIVE, PRISMA**) have been followed or provided.	Not Applicable	
For **tumor marker prognostic studies,** we recommend that you follow the **REMARK** reporting guidelines (see link list at top right). See author guidelines, under 'Reporting Guidelines'. Please confirm you have followed these guidelines.	Not Applicable	
For **phase II and III randomized controlled trials**, please refer to the **CONSORT** flow diagram (see link list at top right) and submit the CONSORT checklist (see link list at top right) with your submission. See author guidelines, under 'Reporting Guidelines'. Please confirm you have submitted this list.	Not Applicable	

Data Availability

Data availability	Information included in the manuscript?	In which section is the information available? (Reagents and Tools Table, Materials and Methods, Figures, Data Availability Section)
Have **primary datasets** been deposited according to the journal's guidelines (see 'Data Deposition' section) and the respective accession numbers provided in the Data Availability Section?	Not Applicable	
Were **human clinical and genomic datasets** deposited in a public access-controlled repository in accordance to ethical obligations to the patients and to the applicable consent agreement?	Not Applicable	
Are **computational models** that are central and integral to a study available without restrictions in a machine-readable form? Were the relevant accession numbers or links provided?	Not Applicable	
If publicly available data were reused, provide the respective **data citations in the reference list**.	Yes	Methods
